# Four-coordinate triarylborane synthesis *via* cascade B–Cl/C–B cross-metathesis and C–H bond borylation†
†Electronic supplementary information (ESI) available. CCDC 1814302 and 1817506. For ESI and crystallographic data in CIF or other electronic format see DOI: 10.1039/c8sc02281j


**DOI:** 10.1039/c8sc02281j

**Published:** 2018-08-13

**Authors:** Kai Yang, Guan Zhang, Qiuling Song

**Affiliations:** a Institute of Next Generation Matter Transformation , College of Chemical Engineering , College of Material Sciences Engineering at Huaqiao University , 668 Jimei Boulevard , Xiamen , Fujian 361021 , P. R. China . Email: qsong@hqu.edu.cn; b State Key Laboratroy of Organometallic Chemistry , Shanghai Institute of Organic Chemistry , Chinese Academy of Sciences , Shanghai 200032 , P. R. China

## Abstract

To develop a simple and efficient synthetic method for four-coordinate triarylboranes, we herein describe a tandem highly selective B–Cl/C–B cross-metathesis of two of the same or different arylboranes and C–H bond borylation to synthesize four-coordinate triarylboranes with a broad substrate scope. By switching substituent groups of the target molecules, different emission wavelengths can be achieved from 467 nm to 583 nm with aggregation-induced emission (AIE) properties.

## Abstract 2

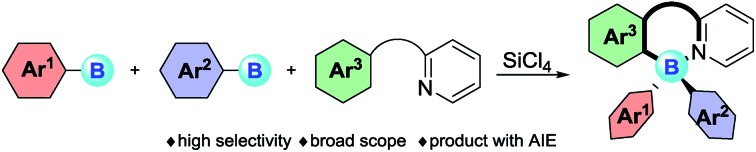
We herein describe a tandem highly selective B–Cl/C–B cross-metathesis of two same or different arylboranes and C–H bond borylation to synthesize four-coordinate triarylboranes with broad substrate scope.

## 1. Introduction

**Fig. 1:**
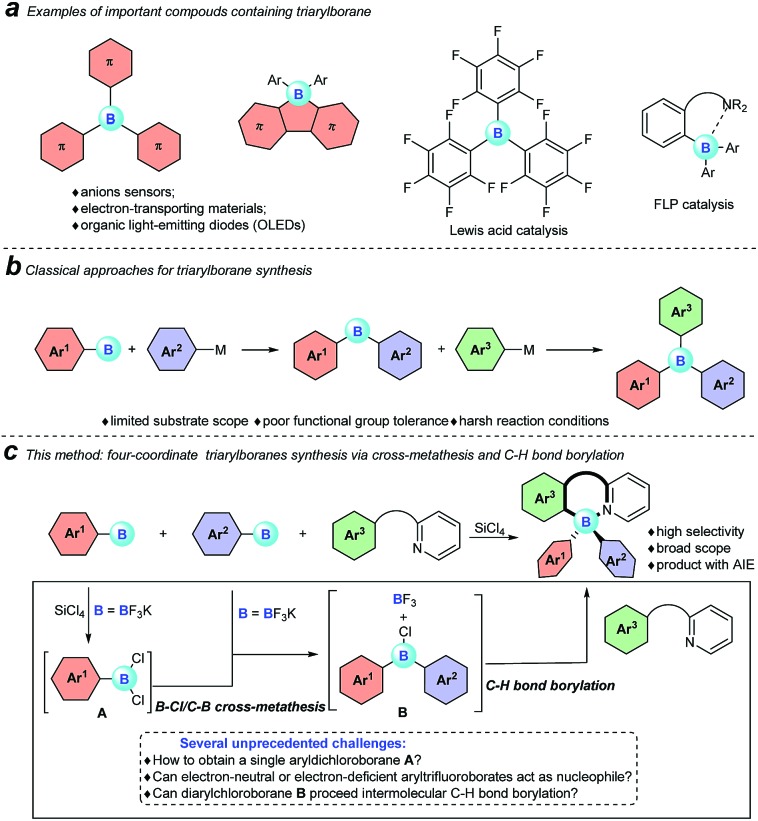
Our motivation and a comparison of classical approaches with our cross-metathesis and C–H bond borylation reaction for triarylborane synthesis are described here.

Triarylboranes and four-coordinate triarylboranes have had an unshakeable position among organic photoelectronic materials because of the unique electron-accepting character of boron atoms and their Lewis acidity, therefore, they have been extensively used as anion sensors,^1^ electron-transporting materials,^2^ and imaging materials^3^ as well as organic light emitting devices (OLEDs).^4^ Meanwhile, triarylboranes can serve as significant catalysts, for instance as direct Lewis acid catalysts (B(C_6_F_5_)_3_), part of intermolecular frustrated Lewis pair (FLP) catalysts,^5^ and intramolecular FLP catalysts^6^ (Fig. 1a).

However, despite the great significance of triarylboranes and four-coordinate triarylboranes, synthetic methods for them are astonishingly rare, hardly matching the rapidly growing demand for them, especially when three-different-substituted triarylboranes are needed.^7^ In the past decade, the majority of triarylborane compounds have been synthesized through the double nucleophilic addition of organometallic reagents, or organosilanes, to arylborons (Fig. 1b).^8^ Unfortunately, the diversity of substrates has been highly restricted by the strong nucleophilicity of these organometallic reagents and the harsh reaction conditions. Therefore, the development of a concise and efficient synthetic approach to synthesize triarylboranes and four-coordinate triarylboranes has become extremely attractive yet challenging as well.

With this background, to further develop synthetic methods for four-coordinate triarylboranes, we envision that a process involving B–Cl/C–B cross-metathesis would occur between *in situ* generated aryldichloroborane **A** by the addition of SiCl_4_ to aryltrifluoroborate^9^ and another aryltrifluoroborate as a nucleophile to afford diarylchloroborane **B**, which then undergoes pyridine directed intermolecular sp^2^ C–H bond borylation,^10^ and a four-coordinate triarylborane might be the resultant product (Fig. 1c). Recently, directed C–H bond functionalization has emerged as a straightforward effective method for the synthesis of organic photoelectronic materials.^11^ Cross-metathesis reactions^12^ have had a transformative impact on chemistry with exciting synthetic value. However, despite being fundamentally fascinating and synthetically useful, cross-metathesis in organic chemistry is relatively rare and has not been well investigated so far. The most famous one is olefin metathesis.10a,b Recently, P, S,12*g* and Si12*h* atom-involved cross metathesis has started to catch the eye of chemists, and in terms of the B atom this transformation has rarely been reported yet,^13^ let alone the cross-metathesis of two different arylboranes, due to the synthetic challenges and poor chemoselectivity. There are several unprecedented challenges in this hypothesis: (1) two different aryldichloroboranes are generated, leading to multiple product mixtures; (2) electron-neutral or -deficient aryltrifluoroborates as pure nucleophiles have rarely been investigated;^14^ (3) it is well known that boron trihalides can participate in pyridine directed sp^2^ C–H bond borylation to construct four-coordinate organoboron fluorophores, but no other forms of boron have ever been reported under metal-free conditions yet.^15^ Herein, we report an extraordinary step-economic strategy which solves all the above questions to construct four-coordinate triarylboranes in one step through a combination of B–Cl/C–B cross-metathesis of two arylboranes and a sequential pyridine directed C–H bond borylation. The reaction proceeds under relatively simple conditions, featuring high efficiency, excellent selectivity, a broad substrate scope and new types of target molecule with AIE properties.

## 2. Results and discussion

### 2.1 Optimization study

As they are readily available, *N*-phenylpyridin-2-amine (**1**) and potassium phenyltrifluoroborate (**2**) were chosen as test substrates for the optimization study (Table 1). To our delight, exposure of **1** to SiCl_4_ and Et_3_N triggered both B–Cl/C–B cross-metathesis and C–H bond borylation to afford product **3** in 47% yield along with 31% yield of by-product **4** (entry 1). Further solvent screening suggested that toluene was best compared to xylene, *o*-xylene, mesitylene and chlorobenzene (entries 1–5). Next, the reaction temperature was investigated (entries 6 and 7). Interestingly, a temperature decrease from 140 °C to 135 °C improved the yield of **3** and suppressed the yield of **4** significantly (entry 7). The yield of **3** was further increased when the loading of **2** and the base was increased (entry 8). Pleasingly, changing the identity of the base from Et_3_N to *i*Pr_2_NEt improved the yield of four-coordinate triarylborane product **3** to 75% (entry 9). The structures of **3** and **4** were unambiguously confirmed by X-ray crystallographic analysis (CCDC ; 1814302 and ; 1817506).†

**Table 1:** The development of optimized conditions for four-coordinate triarylborane formation^*a*^

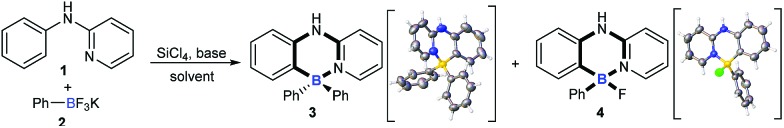						
Entry	Solvent	Base	*T* (°C)	Time (h)	3 yield^*b*^ (%)	4 yield^*b*^ (%)
1	Xylene	Et_3_N	140	5	47	31
2	Toluene	Et_3_N	140	5	63	20
3	*o*-Xylene	Et_3_N	140	5	42	27
4	Mesitylene	Et_3_N	140	5	12	15
5	Chlorobenzene	Et_3_N	140	5	21	23
6	Toluene	Et_3_N	135	5	67	7
7	Toluene	Et_3_N	145	5	61	10
8^*c*^	Toluene	Et_3_N	135	3	69	5
9^*d*^	Toluene	*i*Pr_2_NEt	135	3	75	Trace

^*a*^Reaction conditions: **1** (0.2 mmol), **2** (0.4 mmol), SiCl_4_ (0.2 mmol) and Et_3_N (0.6 mmol) in solvent (1 mL) under an N_2_ atmosphere unless otherwise specified.

^*b*^Isolated yield.

^*c*^ **1** (0.2 mmol), **2** (0.48 mmol), SiCl_4_ (0.2 mmol) and Et_3_N (0.72 mmol) in toluene (1 mL).

^*d*^ **1** (0.2 mmol), **2** (0.48 mmol), SiCl_4_ (0.2 mmol) and *i*Pr_2_NEt (0.72 mmol) in toluene (1 mL).

### 2.2 Scope of the investigation

**Scheme 1:**
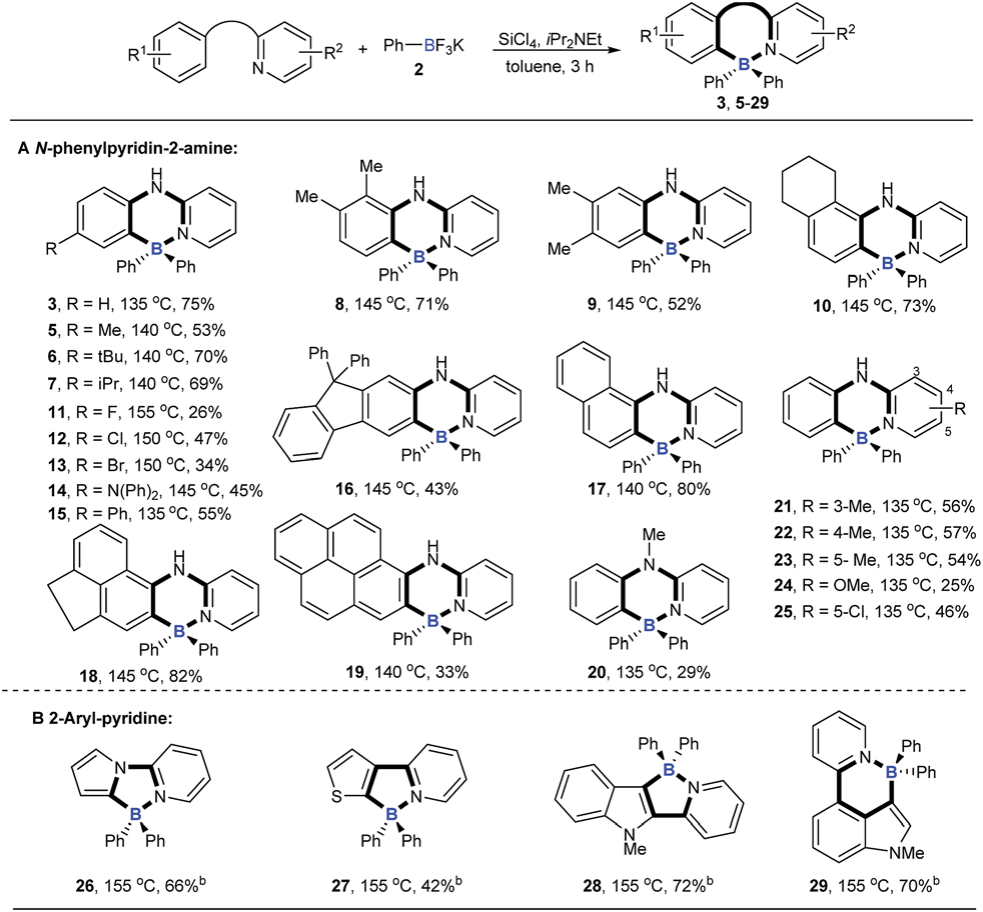
The substrate scope of amines. ^a^Reaction conditions: [a] *N*-arylpyridin-2-amine (0.2 mmol), **2** (0.48 mmol), SiCl_4_ (0.2 mmol), *i*Pr_2_NEt (0.72 mmol), toluene (1 mL), 3 h; [b] 2-aryl-pyridine (0.2 mmol), **2** (0.72 mmol), SiCl_4_ (0.2 mmol), *i*Pr_2_NEt (1 mmol), toluene (1 mL).

With the optimized conditions available, the substrate scope of amines in this tandem transformation was investigated (Scheme 1). Firstly, R^1^-groups with methyl, ethyl, *tert*-butyl, isopropyl and other disubstituted alkyls gave corresponding products **5–10** in 49–73% yields, and remarkably, *meta*-substitution exhibited excellent regioselectivity and only one major regioisomer was obtained probably owing to steric hindrance (**9**). Pleasingly, halogen groups were well-tolerated (**11–13**), providing feasibility for further structural elaborations. Electron-rich substituents, like *N*,*N*-diphenyl which usually emerge in organic photoelectronic materials,^16^ were also compatible under the standard conditions and the desired product was obtained in a moderate yield (**14**). Notably, biphenyl and polycyclic aromatic substrates, such as fluorene, naphthalene, acenaphthene and pyrene all afforded the corresponding target molecules in moderate to good yields (**15–19**). A tertiary amine was also smoothly transformed under the reaction conditions to give product **20** in 29% yield. The diversity of the reaction was also shown by R^2^-groups on a pyridine moiety (*e.g.* methyl, methoxy and chloro groups) and the desired products were afforded in decent yields (**21–25**). 2-aryl-pyridines, which are attractive building blocks for organic photoelectronic materials,^17^ were good substrates for this transformation as well, and the corresponding four-coordinate triarylborane products were obtained with moderate to good yields (**26–29**).

**Scheme 2:**
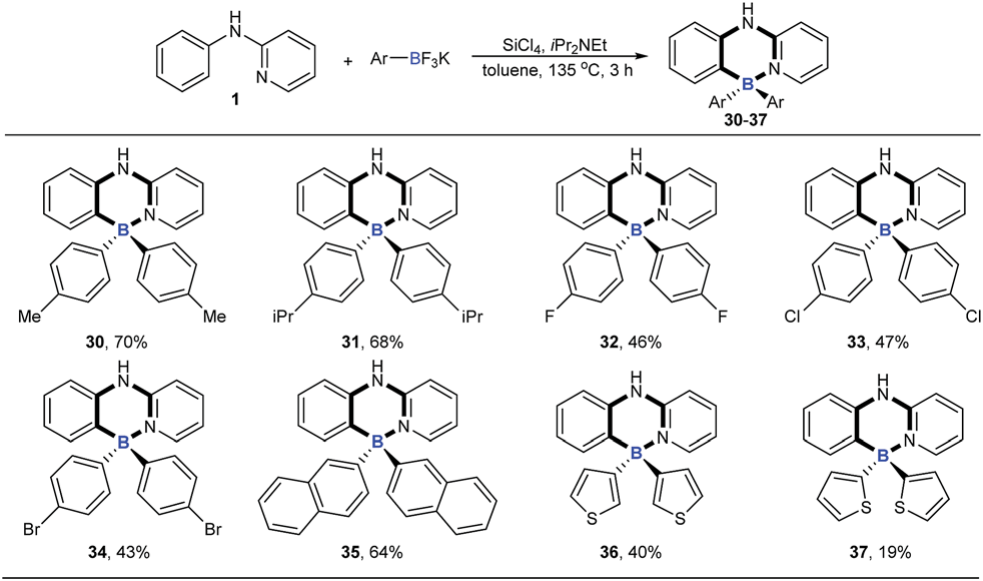
The substrate scope of potassium aryltrifluoroborates. Reaction conditions: **1** (0.2 mmol), potassium aryltrifluoroborates (0.48 mmol), SiCl_4_ (0.2 mmol), *i*Pr_2_NEt (0.72 mmol), toluene (1 mL), 135 °C, 3 h.

We then explored the scope of potassium aryltrifluoroborates (Scheme 2). To the best of our knowledge, the substrate scope of potassium aryltrifluoroborates as pure nucleophiles was limited to the electron-rich aryl and vinyltrifluoroborates.^14^ To our delight, various substituents such as electron-neutral alkyl groups (methyl, isopropyl) and weak electron-deficient halogens (F, Cl, Br) were all compatible under the standard conditions (**30–34**). Moreover, potassium 2-naphthalenyltrifluoroborate and electron-rich potassium thiophenyltrifluoroborate were also good candidates in the reaction to afford the corresponding target molecules (**35–37**) albeit with a lower yield of **37**.

**Scheme 3:**
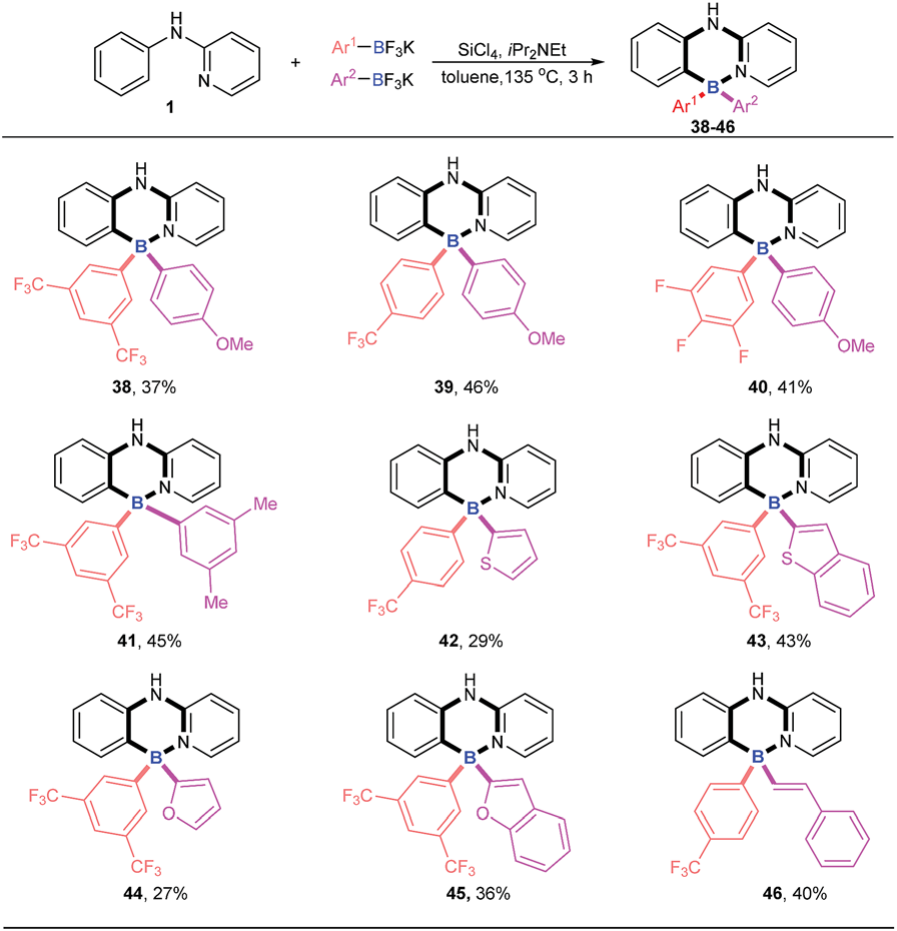
Examination of the reaction versatility with two different potassium aryltrifluoroborates. Reaction conditions: amine **1** (0.2 mmol), Ar^1^BF_3_K (0.24 mmol), Ar^2^BF_3_K (0.24 mmol), SiCl_4_ (0.2 mmol), *i*Pr_2_NEt (0.72 mmol), 135 °C, toluene (1 mL), 3 h.

Four-coordinate triarylboranes with three different substituents are interesting molecules,^7^,^18^ yet their syntheses are very rare due to the great challenge of selectivity. How to realize their construction in a one pot strategy has become a very attractive challenge. With our new strategy in hand, we examined the versatility of this reaction with two different potassium aryltrifluoroborates (Scheme 3). In order to suppress the formation of the homo-linking by-products and increase the formation of cross-over desired products, we hypothesized that the combination of electron-rich potassium aryltrifluoroborates with aryldichloroborane, which was generated *in situ* from electron-deficient potassium aryltrifluoroborate and SiCl_4_, will favor the cross-over product since the former is strongly nucleophilic. Therefore, we chose one electron-poor potassium aryltrifluoroborate (pink), such as 3,5-diCF_3_, 4-CF_3_ and 3,4,5-trifluoro potassium aryltrifluoroborate, and one electron-rich potassium aryltrifluoroborate with 4-MeO, 3,5-diMe, or other electron-rich heterocyclics (purple) (thiophene, benzothiophene, furan, and benzofuran) as substrates to proceed *via* this B–Cl/C–B cross-metathesis and C–H bond borylation tactic. To our delight, these substrates afforded targeted products with high selectivities (**38–45**) without homo-linking molecules (BAr_2_^1^ and BAr_2_^2^) detected and only a trace amount of by-product **4** was ever obtained, which stemmed from electron-poor potassium aryltrifluoroborates (for details, see ESI Scheme 1†). Activated potassium vinyltrifluoroborate also furnished products **46** in 40% yield with excellent selectivity.

**Scheme 4:**
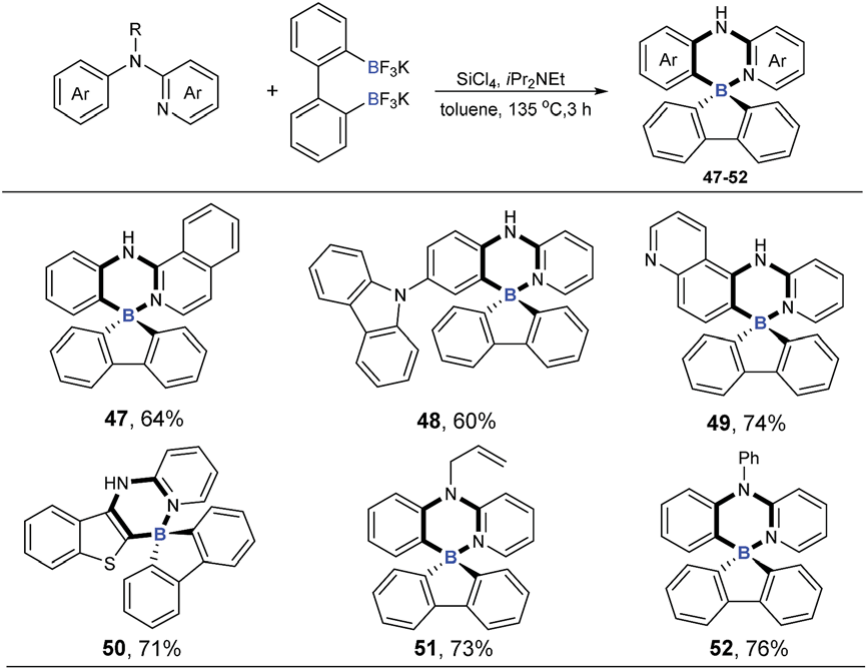
Cascade ring-closing B–Cl/C–B cross-metathesis and C–H bond borylation. Reaction conditions: amine (0.2 mmol), Ar(BF_3_K)_2_ (0.24 mmol), SiCl_4_ (0.2 mmol), *i*Pr_2_NEt (0.72 mmol), 135 °C, toluene (1 mL), 3 h.

Four-coordinate spiro-triarylboranes are very peculiar molecules,^19^ since both six-membered and five-membered rings are connected on a shared boron atom, whose special structures might lead to special properties. Therefore, we next became interested in applying this tandem cross-metathesis and C–H activation reaction to the preparation of four-coordinate spiro-triarylborane (Scheme 4). These substrates including isoquinoline and pyridine with a carbazole moiety on the benzene ring showed a good reactivity to obtain the corresponding spiro four-coordinate triarylboranes in satisfactory yields (**47** and **48**). Diheterocyclic organoboron structures are widely found in organic dyes and materials,4a,^20^ so nitrogen or sulphur-containing heterocyclic amines were chosen as substrates, and to our delight, the reaction allowed the formation of corresponding diheterocyclic spiro ones in good yields (**49** and **50**). Finally, tertiary amines were also successfully employed, affording the ring-closing products in good yields (**51** and **52**), respectively.

### 2.3 Mechanistic study

**Scheme 5:**
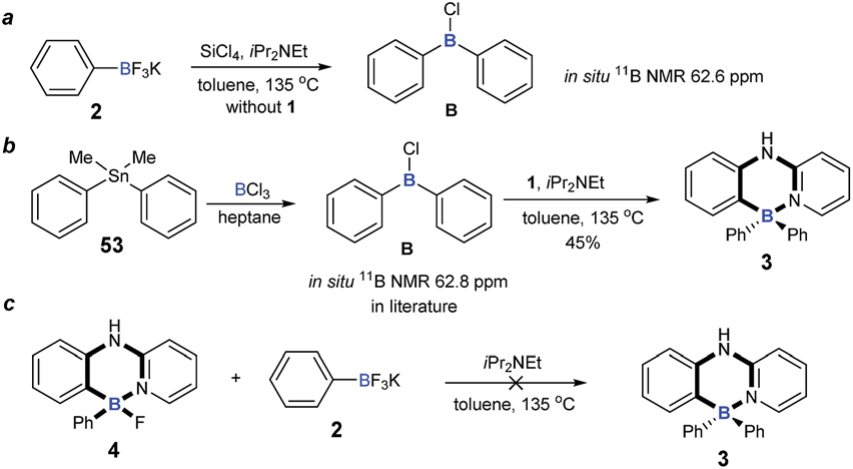
Experimental mechanistic studies. (a) Control experiment for probable intermediate; (b) synthesis of four-coordinate triarylborane from probable intermediate. (c) Control experiment of by-product.

To understand the mechanism of the cascade B–Cl/C–B cross-metathesis and C–H bond borylation reaction, a control experiment was first carried out in the absence of amine **1**. In Scheme 5a, compound **B** was obtained whose structure was verified by *in situ* NMR spectroscopy (^11^B NMR 62.6 ppm *vs.* 62.8 ppm in the literature^21^), but compound **B** was not detected without iPrNEt_2_ which probably served to quench the BF_3_ from B–Cl/C–B cross-metathesis (for details, see ESI Fig. 1†). In order to prove that compound **B** is the key intermediate for this cascade process, the *in situ* formed diphenylchloroborane **B** that was prepared according to the method in the literature^21^ was exposed to amine **1**, and target molecule **3** was obtained under standard conditions in 45% yield (Scheme 5b). This result undeniably convinced us that compound **B** is the key intermediate for our transformation. By-product **4** can’t be transformed into the four-coordinate triarylborane product **3** in our reaction system (in Scheme 5c), so it excluded the approach of directly converting **4** to **3**, because the nucleophilic substitution reaction of the B–F bond in four-coordinate organoborane compounds was limited by strong nucleophilic reagents such as Grignard reagents and organolithium reagents.^22^

**Scheme 6:**
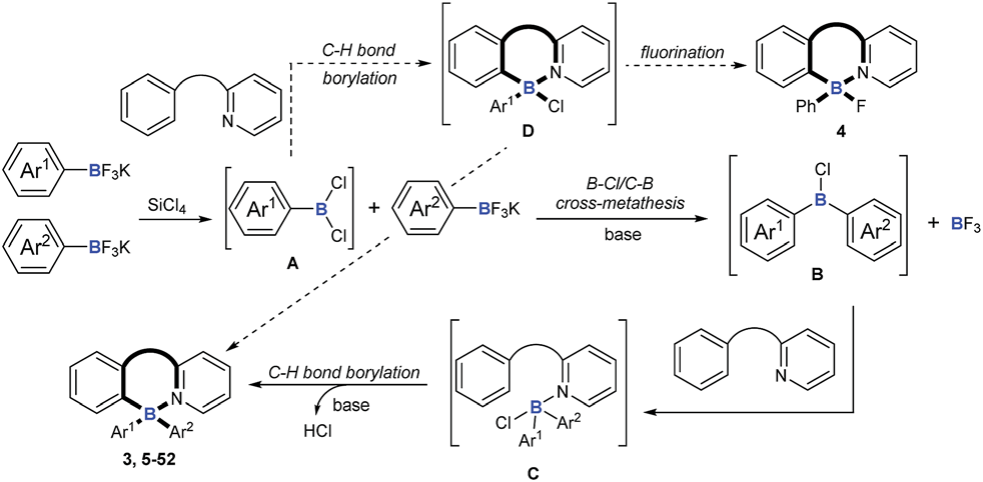
Plausible reaction mechanisms.

On the basis of the above results and previous reports,^9^,^10^,^22^,^23^ we proposed a mechanism for this cascade B–Cl/C–B cross-metathesis and C–H borylation reaction (Scheme 6). An active aryldichloroborane **A** intermediate could be highly selectively obtained which was generated *in situ* by the addition of SiCl_4_ to aryltrifluoroborates^9^ when Ar^1^ is an electron-deficient aryltrifluoroborate and Ar^2^ is an electron-rich aryltrifluoroborate. Intermediate **A** reacts with another aryltrifluoroborate (Ar^2^BF_3_K) *via* B–Cl/C–B cross-metathesis to obtain diphenylchloroborane **B**, and subsequent pyridine directed electrophilic aromatic borylation^10^ of amine **1** eventually leads to four-coordinate triarylborane products **3**, **5–52**. In addition, intermediate **A** could also proceed *via* C–H bond borylation9b,^23^ with amine **1**, and subsequent fluorination^24^ leads to by-product **4**. We can’t rule out the possibility of forming the four-coordinate triarylborane products *via* B–Cl/C–B cross-metathesis between intermediate **D** and another aryltrifluoroborate (Ar^2^BF_3_K).

### 2.4 Fluorescence properties

**Fig. 2:**
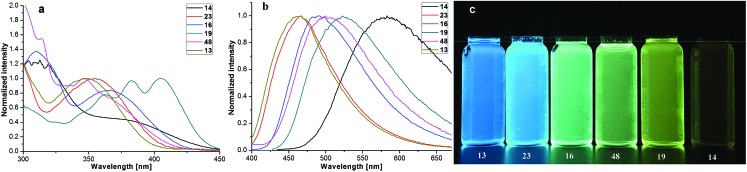
(a) Absorption spectra of some four-coordinate triarylborane products in DCM; (b) emission spectra of some four-coordinate triarylborane products in DCM; (c) picture of some four-coordinate triarylborane products under UV light irradiation (365 nm).

**Fig. 3:**
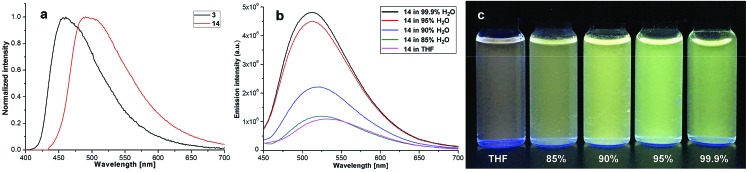
(a) Emission spectra of solid samples of **3** and **14**; (b) emission spectra of **17** in H_2_O/THF; (c) picture of **14** in H_2_O/THF mixtures under UV light irradiation (365 nm).

Our four-coordinate triarylborane compounds are structurally similar to the known organoboron compounds4a,20a,^25^ that have found promising applications in light emitting materials. Accordingly, we found that our products fluoresce under light irradiation, and the absorption and emission spectra have been collected. In the absorption spectra (Fig. 2a), our products show absorption maxima from 310 nm to 410 nm. By switching substituent groups, fluorescent molecules with different emission wavelengths can be achieved ranging from 467 nm to 583 nm as shown in Fig. 2b and c. To our utmost delight, when the quantum yields of some of the products were inspected, we found that the quantum yields of the solid-state products are higher than those of their counterparts in solution, for example, compound **3** has a quantum yield of 42% in the solid-state, yet its quantum yield reduces to 29% in solvent; the quantum yield of **14** is 0.5% in solvent but increases to 29% in the solid-state (for details, see ESI Table 1†). We then collected solid emission spectra of **3** and **14** (Fig. 3a). We envisioned that the four-coordinate triarylborane compounds synthesized with our strategy would possess photophysical properties with aggregation-induced emission (AIE) phenomena,^26^ and our conjecture was well proven by subsequent experiments: product **14** completely dissolved in THF and showed very weak fluorescence after UV irradiation, but the fluorescence intensity enhanced significantly with increasing amounts of water fraction up to 99.9% (Fig. 3b and c). These experimental data and phenomena suggested that our four-coordinate triarylboranes might be a new type of fluorescent organic material showing AIE phenomena and their efficient construction might add extra value for this type of compound in organic photoelectronic material applications.

## 3. Conclusions

We have shown that four-coordinate triarylboranes were synthesized with high selectivity and a broad substrate scope *via* tandem B–Cl/C–B cross-metathesis of two different arylboranes and C–H bond borylation. Our data suggest that the target molecules obtained from our strategy possess different emission wavelengths by switching substituent groups, and a potential new fluorescent organic material with AIE properties can be achieved. Our future experiments are aimed at further investigating the characteristics of these products as well as extending this new reactivity and expanding the substrate scope.

## Conflicts of interest

There are no conflicts to declare.

## Supplementary Material

Supplementary information

Crystal structure data
