# Supplementary material for: Four-coordinate triarylborane synthesis via cascade B–Cl/C–B cross-metathesis and C–H bond borylation
Source: Chem Sci. 2018 Aug 13;9(39):7666–72. doi: 10.1039/c8sc02281j (PMC6182419; doi:10.1039/c8sc02281j)
Supplement: Supplementary file 1 [file SC-009-C8SC02281J-s001.pdf]

Electronic Supplementary Material (ESI) for Chemical Science.
This journal is © The Royal Society of Chemistry 2018

Four-Coordinate Triarylborane Synthesis via Cascade B-Cl/C-B Cross-Metathesis and C-H Bond Borylation

Kai Yang,[a] Guan Zhang,[a] Qiuling Song*[a,b]

[a] Institute of Next Generation Matter Transformation, College of Chemical Engineering, College of Material Sciences Engineering at Huaqiao University, 668 Jimei Boulevard, Xiamen, Fujian 361021, P. R. China

[b] State Key Laboratroy of Organometallic Chemistry, Shanghai Institute of Organic Chemistry, Chinese Academy of Sciences, Shanghai 200032, P. R. China.

General. All experiments were conducted with a schlenk tube. Flash column chromatography was performed over silica gel (200-300 mesh). ^{1}H NMR and ^{13}C NMR spectra were recorded at ambient temperature using Bruker AVIII-500M spectrometers, chemical shifts (in ppm) were referenced to $CDCl_3$ (δ = 7.26 ppm), DMSO-d_6 (δ = 2.50 ppm) and acetone-d_6 (δ = 2.05 ppm) as internal standards. ^{13}C NMR spectra were obtained by using the same NMR spectrometers and were calibrated with $CDCl_3$ (δ = 77.0 ppm), DMSO-d_6 (δ = 39.5 ppm) and acetone-d_6 (δ = 206.68 ppm). The amines was prepared from the corresponding 2-bromopyridine with arylamines according to previous literature.[1] The potassium aryltrifluoroborates were prepared from the corresponding arylboronic acids and KF_2H. Unless otherwise noted, materials obtained from commercial suppliers were used without further purification.

[1] Y., Onoda, M., Suzuki and T., Kanbara, JP 2009286716

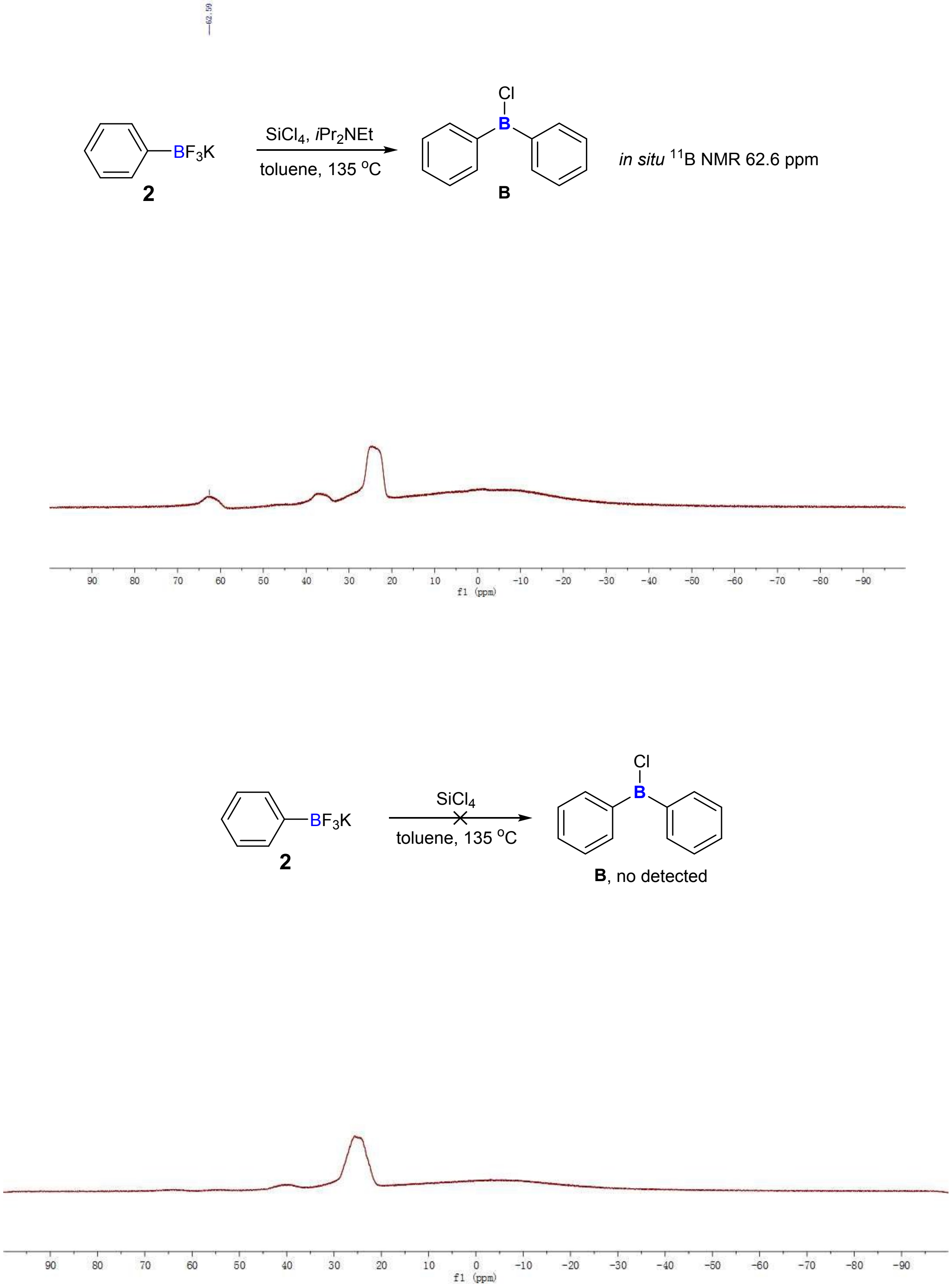

Supplementary Figure 1. Control experiment for probable intermediate. **a** 2 (0.48 mmol), $SiCl_4$ (0.2 mmol), *i*Pr_2NEt (0.72 mmol), 135 °C, toluene (1 mL), 4.5 h; **b** 2 (0.48 mmol), $SiCl_4$ (0.2 mmol), 135 °C, toluene (1 mL), 4.5 h.

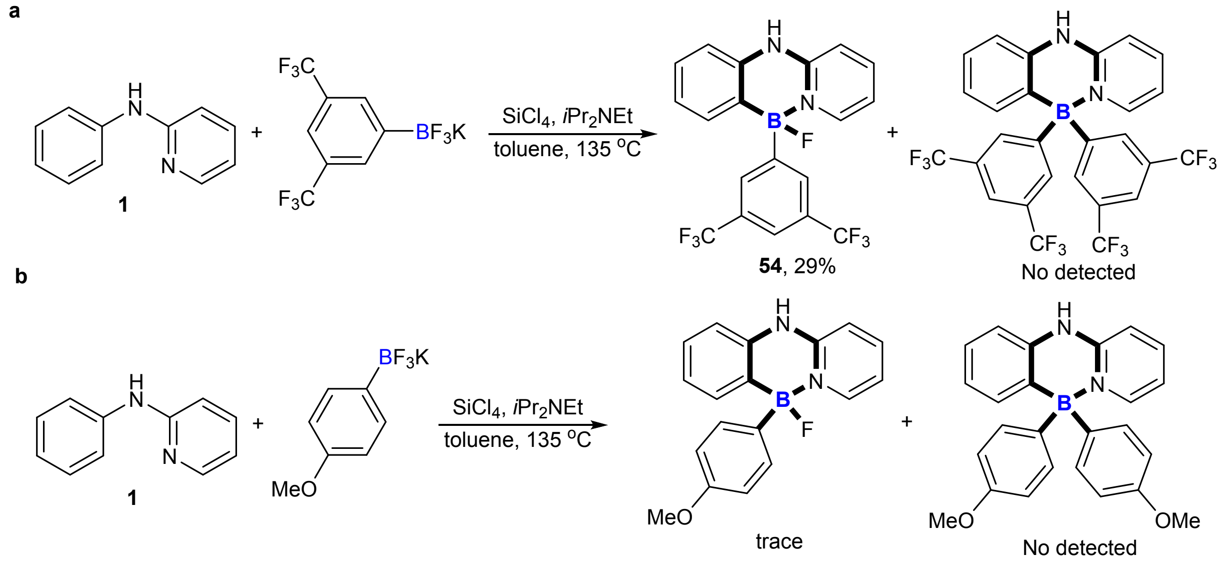

Supplementary Scheme 1. Symmetric molecule (BAr^12 or BAr^22) was not detected in our reaction when only added one electron-rich potassium aryltrifluoroborate or electron- deficient potassium aryltrifluoroborate.

Supplementary Table 1. quantum yields of some four-coordinate triarylborane products in DCM and solid state

number	quantum yields in DCM (%)	quantum yields in solid state (%)
**3**	28.87	42.07
**7**	17.63	22.75
**17**	0.52	28.94
**29**	23.96	39.14
**32**	33.28	48.24
**47**	17.65	20.33
**49**	1.49	19.66

General procedure A for the preparation of four-coordinate triarylborane products from amines and potassium aryltrifluoroborates.

In air, a 25 mL schlenk tube was charged with amine (0.2 mmol, 1 equiv) and potassium aryltrifluoroborates (0.48 mmol, 2.4 equiv). The tube was evacuated and filled with nitrogen for three cycles. Then, toluene (1 mL), $SiCl_4$ (0.2 mmol, 24 µl, 1 equiv) and *i*Pr_2NEt (0.72 mmol, 119 µl, 3.6 equiv) was added at room temperature. The reaction was allowed to stir at corresponding temperature for 3 hours. Upon completion, the reaction was cooled to room temperature and proper amount of silica gel was added. After removal of the solvent, the crude reaction mixture was purified

on silica gel (petroleum ether: ethyl acetate = 5:1-1:1) to afford the desired product.

General procedure B for the preparation of four-coordinate triarylborane products from 2-aryl-pyridines and potassium aryltrifluoroborates.

In air, a 25 mL schlenk tube was charged with 2-aryl-pyridine (0.2 mmol, 1 equiv) and potassium aryltrifluoroborates (0.72 mmol, 3.6 equiv). The tube was evacuated and filled with nitrogen for three cycles. Then, toluene (1 mL), $SiCl_4$ (0.2 mmol, 24 µl, 1 equiv) and *i*Pr_2NEt (1 mmol, 165 µl, 5 equiv) was added at room temperature. The reaction was allowed to stir at corresponding temperature for 3 hours. Upon completion, the reaction was cooled to room temperature and proper amount of silica gel was added. After removal of the solvent, the crude reaction mixture was purified on silica gel (petroleum ether: ethyl acetate = 5:1-1:1) to afford the desired product.

General procedure C for the preparation of four-coordinate triarylborane products from amines and two different potassium aryltrifluoroborates.

In air, a 25 mL schlenk tube was charged with amine (0.2 mmol, 1 equiv), potassium aryltrifluoroborates **2** (0.24 mmol, 1.2 equiv) and potassium aryltrifluoroborates **4** (0.24 mmol, 1.2 equiv). The tube was evacuated and filled with nitrogen for three cycles. Then, toluene (1 mL), $SiCl_4$ (0.2 mmol, 24 µl, 1 equiv) and *i*Pr_2NEt (0.72 mmol, 119 µl, 3.6 equiv) was added at room temperature. The reaction was allowed to stir at corresponding temperature for 3 hours. Upon completion, the reaction was cooled to room temperature and proper amount of silica gel was added. After removal of the solvent, the crude reaction mixture was purified on silica gel (petroleum ether: ethyl acetate = 5:1-1:1) to afford the desired product.

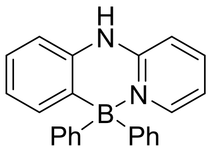

6,6-diphenyl-6,11-dihydro-5l4,6l4-benzo[c]pyrido[2,1-f][1,5,2]diazaborinine, 3. The General procedure A was followed by using *N*-phenylpyridin-2-amine (34 mg, 0.2 mmol, 1 equiv), potassium phenyltrifluoroborate (89 mg 0.48 mmol, 2.4 equiv), $SiCl_4$ (0.2 mmol, 24 µl, 1 equiv), *i*Pr_2NEt (0.72 mmol, 119 µl, 3.6 equiv) and toluene (1 mL) to afford 51 mg of the product as a white solid (75%). mp 240-241 °C. ^{1}H NMR (500 MHz, acetone) δ 9.56 (s, 1H), 7.83-7.81 (m, 1H), 7.74 (d, J = 6.3 Hz, 1H), 7.19 – 6.98 (m, 13H), 6.94 – 6.86 (m, 2H), 6.83 (d, J = 7.9 Hz, 1H). ^{13}C NMR (126 MHz, acetone) δ 152.7, 144.7, 141.4, 139.6, 135.2, 128.2, 127.0, 126.2, 124.4, 115.5,

114.8, 114.4. ^{11}B NMR (160 MHz, Acetone) δ -1.21. HRMS (ESI) m/z calcd for C23H20BN2 $[M+H]^+$: 335.1714, found 335.1716.

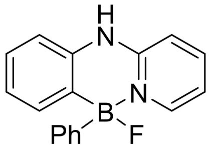

6-fluoro-6-phenyl-6,11-dihydro-5l4,6l4-benzo[c]pyrido[2,1-f][1,5,2]diazaborinine, 4. white solid. mp 249-251 °C. ^{1}H NMR (500 MHz, Acetone) δ 9.89 (s, 1H), 7.99 (d, *J* = 6.1 Hz, 1H), 7.88 – 7.72 (m, 1H), 7.33-7.28 (m, 3H), 7.20 (d, *J* = 8.7 Hz, 1H), 7.18 – 7.10 (m, 3H), 7.09-7.04 (m, 1H), 7.00-6.95 (m, 2H), 6.90 (t, *J* = 6.6 Hz, 1H). ^{13}C NMR (126 MHz, acetone) δ 150.7, 142.4, 141.6, 138.8, 138.7, 134.4, 132.5, 132.5, 128.3, 118.2, 126.9, 124.3, 115.5, 115.1, 114.9. ^{19}F NMR (471 MHz, Acetone) δ -168.7. ^{11}B NMR (160 MHz, acetone) δ 4.23. HRMS (ESI) m/z calcd for C17H14BFN2Na $[M+Na]^+$: 299.1126, found 299.1127.

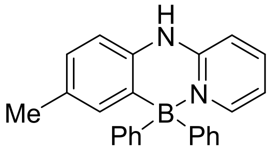

8-methyl-6,6-diphenyl-6,11-dihydro-5l4,6l4-benzo[c]pyrido[2,1-f][1,5,2]diazaborinine, 5. The general procedure A was followed by using *N*-(p-tolyl)pyridin-2-amine (36.8 mg, 0.2 mmol, 1 equiv), potassium phenyltrifluoroborate (89 mg 0.48 mmol, 2.4 equiv), $SiCl_4$ (0.2 mmol, 24 µl, 1 equiv), *i*Pr_2NEt (0.72 mmol, 119 µl, 3.6 equiv) and toluene (1 mL) to afford 37 mg of the product as a white solid (53%). mp 237-238 °C. ^{1}H NMR (500 MHz, $CDCl_3$) δ 7.78 (dd, *J* = 6.3, 1.5 Hz, 1H), 7.52-7.49 (m, 1H), 7.26 – 7.20 (m, 4H), 7.20 – 7.10 (m, 6H), 6.95 (s, 1H), 6.88 (dd, *J* = 7.9, 1.5 Hz, 1H), 6.79 (s, 1H), 6.69 – 6.66 (m, 1H), 6.49 (t, *J* = 7.9 Hz, 2H), 2.24 (s, 3H). ^{13}C NMR (126 MHz, CDC13) δ 150.5, 143.7, 139.3, 135.0, 134.6, 134.0, 132.9, 127.1, 126.5, 125.3, 114.0, 112.8, 112.6, 21.1. ^{11}B NMR (160 MHz, $CDCl_3$) δ -0.88. HRMS (ESI) m/z calcd for C24H22BN2 $[M+H]^+$: 349.1871, found 349.1870.

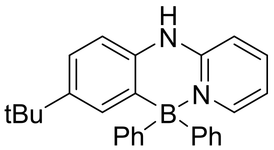

8-(tert-butyl)-6,6-diphenyl-6,11-dihydro-5l4,6l4-benzo[c]pyrido[2,1-f][1,5,2]diazaborinine, 6. The General procedure A was followed by using *N*-(*o*-tolyl)pyridin-2-amine (36.8 mg, 0.2 mmol, 1 equiv), potassium phenyltrifluoroborate (89 mg 0.48 mmol, 2.4 equiv), $SiCl_4$ (0.2 mmol, 24 µl, 1 equiv), *i*Pr_2NEt (0.72 mmol, 119 µl, 3.6 equiv) and toluene (1 mL) to afford 34 mg of the product as a white solid (49%). mp 179-180 °C. ^{1}H NMR (500 MHz, $CDCl_3$) δ 7.80 (dd, *J* = 6.3, 1.3 Hz, 1H), 7.49-7.45 (m, 1H), 7.26 – 7.21 (m, 5H), 7.19 – 7.14 (m, 6H), 7.10 (dd, *J* = 8.2, 2.3 Hz, 1H), 6.73 (s, 1H), 6.69 – 6.64 (m, 1H), 6.47 (d, *J* = 8.2 Hz, 1H), 6.42 (d, *J* = 8.6 Hz, 1H), 1.25 (s, 9H). ^{13}C NMR (126 MHz, $CDCl_3$) δ 150.6, 146.2, 143.6, 139.2, 135.1, 134.0, 131.3,

127.1, 125.2, 122.5, 114.0, 112.8, 112.2, 34.3, 31.5. ^{11}B NMR (160 MHz, $CDCl_3$) δ -0.77 (s). HRMS (ESI) m/z calcd for C27H28BN2 $[M+H]^+$: 391.2340, found 391.2347.

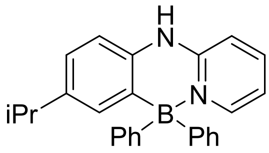

8-isopropyl-6,6-diphenyl-6,11-dihydro-5l4,6l4-benzo[c]pyrido[2,1-f][1,5,2]diazaborinine, 7. The General procedure A was followed by using *N*-(4-isopropylphenyl)pyridin-2-amine (42 mg, 0.2 mmol, 1 equiv), potassium phenyltrifluoroborate (89 mg 0.48 mmol, 2.4 equiv), $SiCl_4$ (0.2 mmol, 24 µl, 1 equiv), *i*Pr_2NEt (0.72 mmol, 119 µl, 3.6 equiv) and toluene (1 mL) to afford 52 mg of the product as a white solid (69%). mp 188-189 °C. ^{1}H NMR (500 MHz, $CDCl_3$) δ 7.77 (d, *J* = 5.4 Hz, 1H), 7.49-7.45 (m, 1H), 7.25 (dd, *J* = 11.5, 4.5 Hz, 4H), 7.21 – 7.12 (m, 6H), 7.02 (d, *J* = 1.8 Hz, 1H), 6.95 (dd, *J* = 8.0, 2.0 Hz, 1H), 6.78 (s, 1H), 6.70 – 6.63 (m, 1H), 6.48 (d, *J* = 8.0 Hz, 1H), 6.43 (d, *J* = 8.5 Hz, 1H), 2.83 (hept, *J* = 6.8 Hz, 1H), 1.19 (d, *J* = 6.9 Hz, 6H). ^{13}C NMR (126 MHz, $CDCl_3$) δ 150.6, 144.1, 143.6, 139.2, 135.4, 134.0, 132.6, 127.1, 125.3, 123.3, 114.0, 112.8, 112.7, 33.6, 24.2. ^{11}B NMR (160 MHz, $CDCl_3$) δ -0.66 (s). HRMS (ESI) m/z calcd for C26H26BN2 $[M+H]^+$: 377.2184, found 377.2186.

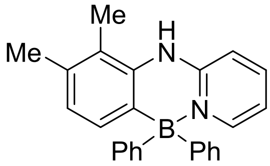

9,10-dimethyl-6,6-diphenyl-6,11-dihydro-5l4,6l4-benzo[c]pyrido[2,1-f][1,5,2]diazaborinine, 8. The General procedure A was followed by using *N*-(2,3-dimethylphenyl)pyridin-2-amine (40 mg, 0.2 mmol, 1 equiv), potassium phenyltrifluoroborate (89 mg 0.48 mmol, 2.4 equiv), $SiCl_4$ (0.2 mmol, 24 µl, 1 equiv), *i*Pr_2NEt (0.72 mmol, 119 µl, 3.6 equiv) and toluene (1 mL) to afford 51 mg of the product as a white solid (71%). mp 226-227 °C. ^{1}H NMR (500 MHz, acetone) δ 8.68 (s, 1H), 7.83-7.79 (m, 1H), 7.71 (dd, *J* = 6.3, 1.4 Hz, 1H), 7.43 (d, *J* = 8.7 Hz, 1H), 7.19 – 7.00 (m, 10H), 6.98 – 6.86 (m, 1H), 6.75 (q, *J* = 7.5 Hz, 2H), 2.23 (s, 3H), 2.22 (s, 3H). ^{13}C NMR (126 MHz, Acetone) δ 153.0, 144.4, 141.1, 137.8, 135.2, 134.6, 132.5, 128.1, 126.3, 126.1, 120.1, 115.7, 115.6, 21.0, 13.3. ^{11}B NMR (160 MHz, acetone) δ -1.30 (s). HRMS (ESI) m/z calcd for C25H24^{10}BN2 $[M+H]^+$: 362.2063, found 362.2062.

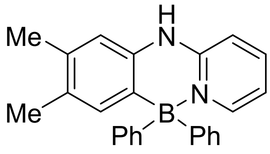

8,9-dimethyl-6,6-diphenyl-6,11-dihydro-5l4,6l4-benzo[c]pyrido[2,1-f][1,5,2]diazaborinine, 9. The General procedure A was followed by using *N*-(3,4-dimethylphenyl)pyridin-2-amine (40 mg, 0.2 mmol, 1 equiv), potassium phenyltrifluoroborate (89 mg 0.48 mmol, 2.4 equiv), $SiCl_4$ (0.2 mmol, 24 µl, 1 equiv), *i*Pr_2NEt (0.72 mmol, 119 µl, 3.6 equiv)

and toluene (1 mL) to afford 38 mg of the product as a white solid (52%). mp 247-249 °C. ^1H NMR (500 MHz, acetone) δ 9.39 (s, 1H), 7.77 – 7.67 (m, 2H), 7.18 – 7.09 (m, 9H), 7.07-7.03 (m, 2H), 6.84 – 6.75 (m, 2H), 6.62 (s, 1H), 2.14 (s, 3H), 2.08 (s, 3H). ^{13}C NMR (126 MHz, acetone) δ 152.6, 144.6, 141.0, 137.5, 136.3, 135.2, 134.7, 131.8, 128.1, 126.1, 115.6, 115.0, 114.7, 20.1, 20.0. ^{11}B NMR (160 MHz, acetone) δ -1.30 (s). HRMS (ESI) m/z calcd for $C25H24^{10}BN2$ $[M+H]^+$: 362.2063, found 362.2061.

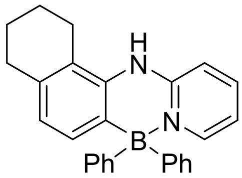

7,7-diphenyl-1,2,3,4,7,13-hexahydro-7l4,8l4-naphtho[2,1-c]pyrido[2,1-f][1,5,2]diazaborinine, 10. The General procedure A was followed by using *N-(5,6,7,8-tetrahydronaphthalen-1-yl)pyridin-2-amine* (44 mg, 0.2 mmol, 1 equiv), potassium phenyltrifluoroborate (89 mg 0.48 mmol, 2.4 equiv), $SiCl_4$ (0.2 mmol, 24 µl, 1 equiv), *i*Pr_2NEt (0.72 mmol, 119 µl, 3.6 equiv) and toluene (1 mL) to afford 57 mg of the product as a white solid (73%). mp 229-230 °C. ^1H NMR (500 MHz, acetone) δ 8.55 (s, 1H), 7.82-7.79 (m, 1H), 7.71 (dd, J = 6.3, 1.6 Hz, 1H), 7.49 – 7.42 (m, 1H), 7.14 – 7.07 (m, 8H), 7.07 – 7.02 (m, 2H), 6.89 (td, J = 7.0, 1.2 Hz, 1H), 6.76 (d, J = 7.5 Hz, 1H), 6.66 (d, J = 7.5 Hz, 1H), 2.68 (t, J = 6.5 Hz, 4H), 1.86-1.81 (m, 2H), 1.74-1.69 (m, 2H). ^{13}C NMR (126 MHz, acetone) δ 152.9, 144.4, 141.1, 137.2, 135.4, 135.2, 132.4, 128.1, 126.1, 125.4, 120.9, 115.7, 115.6, 30.9, 25.1, 24.5, 24.0. ^{11}B NMR (160 MHz, acetone) δ -1.32. HRMS (ESI) m/z calcd for $C27H26^{10}BN2$ $[M+H]^+$: 388.2220, found 388.2227.

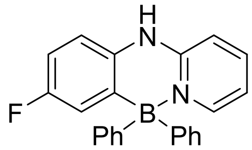

8-fluoro-6,6-diphenyl-6,11-dihydro-5l4,6l4-benzo[c]pyrido[2,1-f][1,5,2]diazaborinine, 11. The General procedure A was followed by using *N*-(4-fluorophenyl)pyridin-2-amine (37 mg, 0.2 mmol, 1 equiv), potassium phenyltrifluoroborate (89 mg 0.48 mmol, 2.4 equiv), $SiCl_4$ (0.2 mmol, 24 µl, 1 equiv), *i*Pr_2NEt (0.72 mmol, 119 µl, 3.6 equiv) and toluene (1 mL) to afford 20 mg of the product as a white solid (26%). mp 230-231 °C. ^1H NMR (500 MHz, acetone) δ 9.62 (s, 1H), 7.84-7.81 (m, 1H), 7.73 (d, J = 6.4 Hz, 1H), 7.19 – 7.12 (m, 5H), 7.13 – 7.06 (m, 6H), 6.91 – 6.85 (m, 2H), 6.80-6.76 (m, 1H), 6.67 (dd, J = 9.4, 2.9 Hz, 1H). ^{13}C NMR (126 MHz, acetone) δ 161.1 (d, J = 237.5 Hz), 152.6, 144.5, 141.6, 135.9, 135.1, 128.4, 126.5, 120.5 (d, J = 19.9 Hz), 115.8 (d, J = 7.5 Hz), 115.7, 114.8, 113.5 (d, J = 23.8 Hz). ^{11}B NMR (160 MHz, acetone) δ -1.34 (s). HRMS (ESI) m/z calcd for C23H18BFN2Na $[M+Na]^+$: 375.1439, found 375.1441.

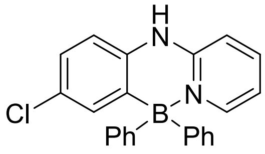 **8-chloro-6,6-diphenyl-6,11-dihydro-5l4,6l4-benzo[c]pyrido[2,1-f][1,5,2]diazaborinine, 12.** The General procedure A was followed by using *N*-(4-chlorophenyl)pyridin-2-amine (40 mg, 0.2 mmol, 1 equiv), potassium phenyltrifluoroborate (89 mg 0.48 mmol, 2.4 equiv), $SiCl_4$ (0.2 mmol, 24 µl, 1 equiv), *i*Pr_2NEt (0.72 mmol, 119 µl, 3.6 equiv) and toluene (1 mL) to afford 35 mg of the product as a white solid (47%). mp 249-250 °C. ^{1}H NMR (500 MHz, DMSO) δ 10.59 (s, 1H), 7.85-7.81 (m, 1H), 7.59 (d, *J* = 6.2 Hz, 1H), 7.18 – 7.10 (m, 5H), 7.10 – 7.04 (m, 3H), 7.02 – 6.95 (m, 4H), 6.91 – 6.83 (m, 2H), 6.74 (d, *J* = 2.4 Hz, 1H). ^{13}C NMR (126 MHz, DMSO) δ 150.4, 142.7, 140.6, 136.8, 133.3, 132.2, 127.1, 126.7, 125.5, 125.2, 115.0, 114.7, 113.5. ^{11}B NMR (160 MHz, DMSO) δ -2.23. HRMS (ESI) m/z calcd for C23H19BClN2 $[M+H]^+$: 369.1324, found 369.1322.

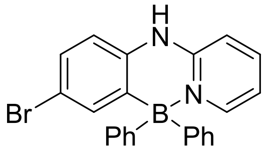

8-bromo-6,6-diphenyl-6,11-dihydro-5l4,6l4-benzo[c]pyrido[2,1-f][1,5,2]diazaborinine, 13. The General procedure A was followed by using *N*-(4-bromophenyl)pyridin-2-amine (49.6 mg, 0.2 mmol, 1 equiv), potassium phenyltrifluoroborate (89 mg 0.48 mmol, 2.4 equiv), $SiCl_4$ (0.2 mmol, 24 µl, 1 equiv), *i*Pr_2NEt (0.72 mmol, 119 µl, 3.6 equiv) and toluene (1 mL) to afford 28 mg of the product as a white solid (34%). mp 257-258 °C. ^{1}H NMR (500 MHz, acetone) δ 9.69 (s, 1H), 7.87-7.84 (m, 1H), 7.75 (dd, *J* = 6.4, 1.5 Hz, 1H), 7.21 – 7.13 (m, 6H), 7.13 – 7.03 (m, 7H), 6.97 – 6.89 (m, 1H), 6.82 (d, *J* = 8.4 Hz, 1H). ^{13}C NMR (126 MHz, acetone) δ 152.5, 144.6, 141.8, 138.8, 137.2, 135.1, 129.8, 128.4, 126.5, 117.2, 116.6, 116.1, 114.9. ^{11}B NMR (160 MHz, acetone) δ -1.39. HRMS (ESI) m/z calcd for C23H19BBrN2 $[M+H]^+$: 413.0819, found 413.0813.

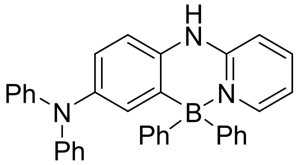 **N,N,6,6-tetraphenyl-6,11-dihydro-5l4,6l4-benzo[c]pyrido[2,1-f][1,5,2]diazaborinin-8-amine, 14.** The General procedure A was followed by using N^1,N^1-diphenyl-N^4-(pyridin-2-yl)benzene-1,4-diamine (67, 0.2 mmol, 1 equiv), potassium phenyltrifluoroborate (89 mg 0.48 mmol, 2.4 equiv), $SiCl_4$ (0.2 mmol, 24 µl, 1 equiv), *i*Pr_2NEt (0.72 mmol, 119 µl, 3.6 equiv) and toluene (1 mL) to afford 45 mg of the product as a yellow solid (45%).

mp 236-237 °C. ^{1}H NMR (500 MHz, acetone) δ 9.62 (s, 1H), 7.84 – 7.80 (m, 1H), 7.64 (d, J = 5.8 Hz, 1H), 7.21-7.17 (m, 5H), 7.13-7.09 (m, 4H), 7.08 – 7.02 (m, 6H), 6.98-6.96 (m, 5H), 6.91-6.81 (m, 4H), 6.73 (dd, J = 8.3, 2.4 Hz, 1H). ^{13}C NMR (126 MHz, acetone) δ 152.6, 149.6, 144.6, 144.2, 141.4, 136.2, 135.1, 132.7, 130.3, 128.3, 126.3, 124.9, 124.0, 122.9, 115.6, 115.5, 114.8. ^{11}B NMR (160 MHz, acetone) δ -1.34. HRMS (ESI) m/z calcd for C35H28BN3 $[M+H]^+$: 502.2449, found 502.2449.

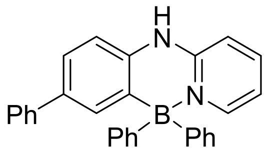

6,6,8-triphenyl-6,11-dihydro-5l4,6l4-benzo[c]pyrido[2,1-f][1,5,2]diazaborinine, 15. The General procedure A was followed by using *N*-([1,1'-biphenyl]-4-yl)pyridin-2-amine (49 mg, 0.2 mmol, 1 equiv), potassium phenyltrifluoroborate (89 mg 0.48 mmol, 2.4 equiv), $SiCl_4$ (0.2 mmol, 24 µl, 1 equiv), *i*Pr_2NEt (0.72 mmol, 119 µl, 3.6 equiv) and toluene (1 mL) to afford 45 mg of the product as a white solid (55%). mp 269-270 °C. ^{1}H NMR (500 MHz, acetone, CS_2) δ 9.74 (s, 1H), 7.84-7.80 (m, 1H), 7.75 (dd, J = 6.3, 1.4 Hz, 1H), 7.50 – 7.41 (m, 2H), 7.37 – 7.27 (m, 4H), 7.26 – 7.11 (m, 10H), 7.10 – 7.03 (m, 2H), 6.96 – 6.91 (m, 1H), 6.91 – 6.85 (m, 1H). ^{13}C NMR (126 MHz, acetone, CS_2) δ 152.6, 144.7, 143.3, 141.4, 139.3 136.9, 135.3, 133.7, 130.0, 128.3, 127.8, 127.7, 126.4, 125.8, 115.7, 115.0, 114.9. ^{11}B NMR (160 MHz, acetone, CS_2) δ -1.05. HRMS (ESI) m/z calcd for C29H24BN2 $[M+H]^+$: 411.2027, found 411.2026.

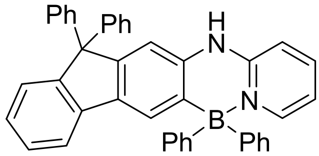

6,6,12,12-tetraphenyl-12,14-dihydro-6H-5l4,6l4-fluoreno[3,2-c]pyrido[2,1-f][1,5,2]diazaborinine, 16. The General procedure A was followed by using *N*-(9,9-diphenyl-9H-fluoren-2-yl)pyridin-2-amine (82 mg, 0.2 mmol, 1 equiv), potassium phenyltrifluoroborate (89 mg 0.48 mmol, 2.4 equiv), $SiCl_4$ (0.2 mmol, 24 µl, 1 equiv), *i*Pr_2NEt (0.72 mmol, 119 µl, 3.6 equiv) and toluene (1 mL) to afford 34 mg of the product as a light yellow solid (49%). mp >295 °C. ^{1}H NMR (500 MHz, acetone, CS_2) δ 9.48 (s, 1H), 7.70-7.65 (m, 2H), 7.50 (d, J = 7.6 Hz, 1H), 7.43 (s, 1H), 7.30 – 6.96 (m, 24H), 6.78 – 6.70 (m, 2H). ^{13}C NMR (126 MHz, acetone, CS_2) δ 153.7, 153.2, 151.9, 149.3, 146.3, 144.0, 142.0, 140.9, 138.1, 136.9, 131.0, 130.9, 130.3, 130.0, 129.3, 128.9, 128.7, 128.5, 128.0, 122.4, 116.7, 116.2, 113.8, 67.8. ^{11}B NMR (160 MHz, acetone, CS_2) δ -1.18. HRMS (ESI) m/z calcd for C42H32BN2 $[M+H]^+$: 575.2653, found 575.2655.

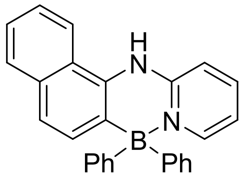

7,7-diphenyl-7,13-dihydro-7l4,8l4-naphtho[2,1-c]pyrido[2,1-f][1,5,2]diazaborinine, 17. The General procedure A was followed by using *N*-(naphthalen-1-yl)pyridin-2-amine (44 mg, 0.2 mmol, 1 equiv), potassium phenyltrifluoroborate (89 mg 0.48 mmol, 2.4 equiv), $SiCl_4$ (0.2 mmol, 24 µl, 1 equiv), *i*Pr_2NEt (0.72 mmol, 119 µl, 3.6 equiv) and toluene (1 mL) to afford 61 mg of the product as a light yellow solid (80%). mp 229-230 °C. ^{1}H NMR (500 MHz, $CDCl_3$) δ 7.92 (dd, *J* = 6.2, 1.2 Hz, 1H), 7.82 (d, *J* = 7.7 Hz, 1H), 7.70 – 7.62 (m, 2H), 7.58 (t, *J* = 7.8 Hz, 1H), 7.53 (d, *J* = 8.2 Hz, 1H), 7.50 – 7.44 (m, 1H), 7.41 (dd, *J* = 11.0, 3.9 Hz, 1H), 7.32 (d, *J* = 8.1 Hz, 1H), 7.26 – 7.13 (m, 10H), 6.84 (d, *J* = 8.5 Hz, 1H), 6.75 (t, *J* = 6.7 Hz, 1H). ^{13}C NMR (126 MHz, $CDCl_3$) δ 150.4, 143.9, 139.6, 134.0, 132.7, 132.3, 130.6, 129.0, 127.1, 125.5, 125.3, 124.6, 123.2, 120.7, 117.5, 114.9, 113.3. ^{11}B NMR (160 MHz, $CDCl_3$) δ -0.74. HRMS (ESI) m/z calcd for C27H22BN2 $[M+H]^+$: 385.1871, found 385.1875.

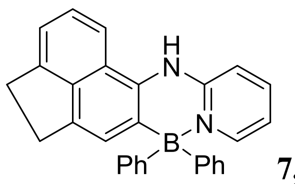

7,7-diphenyl-4,5,7,13-tetrahydro-7l4,8l4-acenaphtho[4,5-c]pyrido[2,1-f][1,5,2]diazaborinine, 18. The General procedure A was followed by using *N*-(1,2-dihydroacenaphthylen-5-yl)pyridin-2-amine (49 mg, 0.2 mmol, 1 equiv), potassium phenyltrifluoroborate (89 mg 0.48 mmol, 2.4 equiv), $SiCl_4$ (0.2 mmol, 24 µl, 1 equiv), *i*Pr_2NEt (0.72 mmol, 119 µl, 3.6 equiv) and toluene (1 mL) to afford 67 mg of the product as a yellow solid (82%). mp 169-171 °C. ^{1}H NMR (500 MHz, acetone, CS_2) δ 9.49 (s, 1H), 7.85 (d, *J* = 8.4 Hz, 1H), 7.81-7.78 (m, 2H), 7.47 (d, *J* = 8.9 Hz, 1H), 7.42 – 7.34 (m, 1H), 7.18-7.10 (m, 9H), 7.05-7.02 (m, 2H), 6.98 (s, 1H), 6.86 (t, *J* = 6.5 Hz, 1H), 3.37 – 3.30 (m, 2H), 3.26 – 3.20 (m, 2H). ^{13}C NMR (126 MHz, acetone, CS_2) δ 153.0, 147.9, 145.1, 141.3, 141.2, 140.4, 135.9, 130.3, 128.9, 128.8, 127.2, 126.7, 122.0, 120.0, 117.1, 116.1, 115.7, 32.4, 31.5. ^{11}B NMR (160 MHz, acetone, CS_2) δ -0.96. HRMS (ESI) m/z calcd for C C29H24^{10}BN2 $[M+H]^+$: 410.2063, found 410.2061.

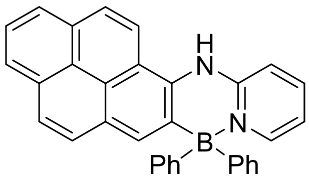
7,7-diphenyl-7,13-dihydro-7l4,8l4-pyreno[2,1-c]pyrido[2,1-f][1,5,2]diazaborinine, 19. The General procedure A was followed by using *N*-(pyren-1-yl)pyridin-2-amine (59 mg, 0.2 mmol, 1 equiv), potassium phenyltrifluoroborate (89 mg 0.48 mmol, 2.4 equiv), $SiCl_4$ (0.2 mmol, 24 µl, 1 equiv), *i*Pr_2NEt (0.72 mmol, 119 µl, 3.6 equiv) and toluene (1 mL) to afford 30 mg of the product as a yellow solid (33%). mp 270-272 °C. ^{1}H NMR (500 MHz, acetone, CS_2) δ 9.84 (s, 1H), 8.38 (d, *J* = 9.3 Hz, 1H), 8.05 – 7.94 (m, 3H), 7.91 – 7.75 (m, 6H), 7.62 (d, *J* = 8.6 Hz, 1H), 7.15 – 6.97 (m, 10H), 6.95 – 6.87 (m, 1H). ^{13}C NMR (126 MHz, acetone, CS_2) δ 154.3, 146.6, 142.5, 137.3, 135.3, 135.0, 134.7, 133.9, 131.3, 130.4, 130.2, 129.6, 128.6, 128.5, 128.3, 127.7, 127.5, 127.1, 126.7, 122.7, 118.5, 117.9, 117.4. ^{11}B NMR (160 MHz, acetone, CS_2) δ -0.66. HRMS (ESI) m/z calcd for C33H24BN2 $[M+H]^+$: 459.2027, found 459.2028.

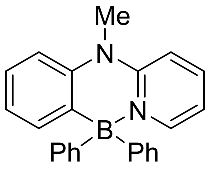
11-methyl-6,6-diphenyl-6,11-dihydro-5l4,6l4-benzo[c]pyrido[2,1-f][1,5,2]diazaborinine, 20. The General procedure A was followed by using *N*-methyl-*N*-phenylpyridin-2-amine (37 mg, 0.2 mmol, 1 equiv), potassium phenyltrifluoroborate (89 mg 0.48 mmol, 2.4 equiv), $SiCl_4$ (0.2 mmol, 24 µl, 1 equiv), *i*Pr_2NEt (0.72 mmol, 119 µl, 3.6 equiv) and toluene (1 mL) to afford 20 mg of the product as a white solid (29%). mp 227-228 °C. ^{1}H NMR (500 MHz, acetone, CS_2) δ 8.04-8.00 (M, 1H), 7.87 (dd, *J* = 6.2, 1.6 Hz, 1H), 7.38 (d, *J* = 8.8 Hz, 1H), 7.22 – 7.15 (m, 1H), 7.12 – 7.07 (m, 4H), 7.07 – 7.02 (m, 4H), 7.01 – 6.98 (m, 2H), 6.97 – 6.93 (m, 4H), 3.52 (s, 3H). ^{13}C NMR (126 MHz, acetone, CS_2) δ 154.4, 145.7, 144.2, 142.6, 135.8, 135.5, 128.7, 127.7, 126.8, 124.9, 116.4, 114.5, 114.2, 37.5. ^{11}B NMR (160 MHz, acetone, CS_2) δ -1.23. HRMS (ESI) m/z calcd for C24H22BN2 $[M+H]^+$: 349.1871, found 349.1872.

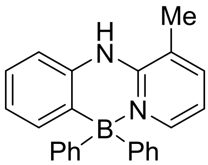

6,6,8-triphenyl-6,11-dihydro-5l4,6l4-benzo[c]pyrido[2,1-f][1,5,2]diazaborinine, 21. The General procedure A was followed by using 3-methyl-*N*-phenylpyridin-2-amine (37 mg, 0.2 mmol, 1 equiv), potassium phenyltrifluoroborate (89 mg 0.48 mmol, 2.4 equiv), $SiCl_4$ (0.2 mmol, 24 µl, 1 equiv), *i*Pr_2NEt (0.72 mmol, 119 µl, 3.6 equiv) and toluene (1 mL) to afford 39 mg of the product as a white

solid (56%). mp 260-262 °C. ^{1}H NMR (500 MHz, acetone) δ 8.54 (s, 1H), 7.70 (dd, *J* = 11.7, 6.7 Hz, 2H), 7.14 – 7.03 (m, 12H), 7.00 (d, *J* = 7.1 Hz, 1H), 6.96 – 6.90 (m, 1H), 6.83 (t, *J* = 6.7 Hz, 1H), 2.44 (s, 3H). ^{13}C NMR (126 MHz, acetone) δ 151.4, 142.7, 141.2, 139.7, 135.2, 135.0, 128.1, 126.9, 126.2, 124.4, 122.7, 115.2, 115.1, 17.9. ^{11}B NMR (160 MHz, acetone) δ -1.08. HRMS (ESI) m/z calcd for C24H22BN2 [M+H]$^{+}$: 349.1871, found 349.1866.

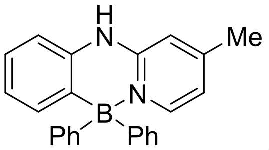

2-methyl-6,6-diphenyl-6,11-dihydro-5l4,6l4-benzo[c]pyrido[2,1-f][1,5,2]diazaborinine, 22. The General procedure A was followed by using 4-methyl-*N*-phenylpyridin-2-amine (37 mg, 0.2 mmol, 1 equiv), potassium phenyltrifluoroborate (89 mg 0.48 mmol, 2.4 equiv), $SiCl_4$ (0.2 mmol, 24 µl, 1 equiv), *i*Pr_2NEt (0.72 mmol, 119 µl, 3.6 equiv) and toluene (1 mL) to afford 40 mg of the product as a white solid (57%). mp 260-262 °C. ^{1}H NMR (500 MHz, acetone) δ 9.38 (s, 1H), 7.61 (d, *J* = 6.5 Hz, 1H), 7.16 – 7.08 (m, 8H), 7.07-7.00 (m, 4H), 6.94 (s, 1H), 6.91-6.88 (m, 1H), 6.81 (d, *J* = 7.6 Hz, 1H), 6.71 (dd, *J* = 6.5, 1.6 Hz, 1H), 2.35 (s, 3H). ^{13}C NMR (126 MHz, acetone) δ 153.3, 152.4, 144.0, 139.7, 135.2, 135.1, 128.1, 126.9, 126.1, 124.1, 117.3, 114.2, 113.7, 21.7. ^{11}B NMR (160 MHz, acetone) δ -1.55. HRMS (ESI) m/z calcd for C24H22BN2 [M+H]$^{+}$: 349.1871, found 349.1869.

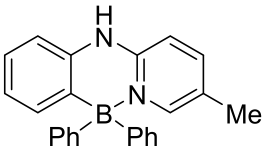

3-methyl-6,6-diphenyl-6,11-dihydro-5l4,6l4-benzo[c]pyrido[2,1-f][1,5,2]diazaborinine, 23. The General procedure A was followed by using 5-methyl-*N*-phenylpyridin-2-amine (37 mg, 0.2 mmol, 1 equiv), potassium phenyltrifluoroborate (89 mg 0.48 mmol, 2.4 equiv), $SiCl_4$ (0.2 mmol, 24 µl, 1 equiv), *i*Pr_2NEt (0.72 mmol, 119 µl, 3.6 equiv) and toluene (1 mL) to afford 38 mg of the product as a white solid (54%). mp 256-257 °C.^{1}H NMR (500 MHz, acetone) δ 9.41 (s, 1H), 7.66 (dd, *J* = 8.8, 2.1 Hz, 1H), 7.58 (s, 1H), 7.17 – 7.09 (m, 9H), 7.08 – 6.97 (m, 4H), 6.88 (td, *J* = 7.3, 1.0 Hz, 1H), 6.80 (d, *J* = 7.9 Hz, 1H), 2.11 (s, 3H). ^{13}C NMR (126 MHz, acetone) δ 151.1, 143.0, 142.8, 139.7, 135.2, 135,1, 128.1, 126.0, 126.1, 124.8, 124.0, 114.5, 114.1, 18.0. ^{11}B NMR (160 MHz, acetone) δ -1.29. HRMS (ESI) m/z calcd for C24H22BN2 [M+H]$^{+}$: 349.1871, found 349.1870.

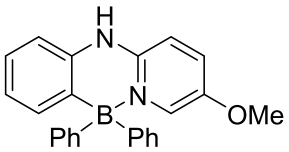

3-methoxy-6,6-diphenyl-6,11-dihydro-5l4,6l4-benzo[c]pyrido[2,1-f][1,5,2]diazaborinine, 24. The General procedure A was followed by using 5-methoxy-*N*-phenylpyridin-2-amine (40 mg, 0.2 mmol, 1 equiv), potassium phenyltrifluoroborate (89 mg 0.48 mmol, 2.4 equiv), $SiCl_4$ (0.2 mmol, 24 µl, 1 equiv), *i*Pr_2NEt (0.72 mmol, 119 µl, 3.6 equiv) and toluene (1 mL) to afford 18 mg of the product as a white solid (25%). mp 243-244 °C. ^{1}H NMR (500 MHz, acetone) δ 9.34 (s, 1H), 7.61 (dd, *J* = 9.3, 2.9 Hz, 1H), 7.36 (d, *J* = 2.9 Hz, 1H), 7.20 – 7.10 (m, 9H), 7.09 – 6.99 (m, 4H), 6.94 – 6.85 (m, 1H), 6.79 (dd, *J* = 8.3, 0.9 Hz, 1H), 3.60 (s, 3H). ^{13}C NMR (126 MHz, acetone) δ 150.4, 148.5, 140.1, 135.2, 135.1, 131.9, 128.2, 127.9, 127.0, 126.2, 123.9, 115.8, 114.1, 57.0. ^{11}B NMR (160 MHz, acetone) δ -0.80 (s). HRMS (ESI) m/z calcd for C24H22^{10}BN2O [M+H]$^+$: 364.1856, found 364.1857.

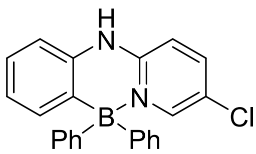

3-chloro-6,6-diphenyl-6,11-dihydro-5l4,6l4-benzo[c]pyrido[2,1-f][1,5,2]diazaborinine, 25. The General procedure A was followed by using 5-chloro-*N*-phenylpyridin-2-amine (40 mg, 0.2 mmol, 1 equiv), potassium phenyltrifluoroborate (89 mg 0.48 mmol, 2.4 equiv), $SiCl_4$ (0.2 mmol, 24 µl, 1 equiv), *i*Pr_2NEt (0.72 mmol, 119 µl, 3.6 equiv) and toluene (1 mL) to afford 34 mg of the product as a yellow solid (46%). mp 232-233 °C. ^{1}H NMR (500 MHz, acetone) δ 9.80 (s, 1H), 7.83 (dd, *J* = 9.2, 2.5 Hz, 1H), 7.70 (d, *J* = 2.5 Hz, 1H), 7.23 (d, *J* = 9.2 Hz, 1H), 7.20 – 7.14 (m, 4H), 7.14 – 7.04 (m, 7H), 7.01 (dd, *J* = 7.3, 1.4 Hz, 1H), 6.95 (td, *J* = 7.3, 1.0 Hz, 1H), 6.86 (d, *J* = 7.9 Hz, 1H). ^{13}C NMR (126 MHz, acetone) δ 151.5, 142.0, 141.5, 139.1, 135.2, 135.1, 128.4, 127.2, 126.5, 124.9, 121.6, 116.6, 114.7. ^{11}B NMR (160 MHz, acetone) δ -0.65. HRMS (ESI) m/z calcd for C23H19BClN2 [M+H]$^+$: 369.1324, found 369.1323.

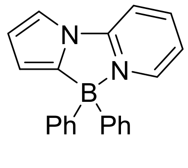

4,4-diphenyl-4H-4l4,5l4-pyrrolo[2',1':3,4][1,4,2]diazaborolo[1,5-a]pyridine, 26. The General procedure B was followed by using 2-(1H-pyrrol-1-yl)pyridine (29 mg, 0.2 mmol, 1 equiv), potassium phenyltrifluoroborate (133 mg 0.72 mmol, 3.6 equiv), $SiCl_4$ (0.2 mmol, 24 µl, 1 equiv), *i*Pr_2NEt (1 mmol, 165 µl, 5 equiv) and toluene (1 mL) to afford 40 mg of the product as a white solid (66%). mp 251-253 °C. ^{1}H NMR (500 MHz, Acetone, CS2) δ 8.48 – 8.41 (m, 1H), 8.21-8.17 (m, 1H), 7.88 – 7.78 (m, 1H), 7.45 (dd, *J* = 2.9, 0.9 Hz, 1H), 7.35-7.32 (m, 1H), 7.29-7.28 (m, 4H), 7.21 – 7.12 (m, 4H), 7.11 – 7.04 (m, 2H), 6.47 (t, *J* = 2.9 Hz, 1H), 6.19 (dd, *J* = 3.0, 0.9 Hz, 1H). ^{13}C NMR (126 MHz, Acetone, CS2) δ 151.5, 145.2, 144.9, 134.4, 128.7, 127.1, 120.3, 118.7, 113.7,

112.1, 110.9. ^{11}B NMR (160 MHz, Acetone, CS2) δ 0.7. HRMS (ESI) m/z calcd for C21H18BN2 $[M+H]^+$: 309.1558, found 309.1557.

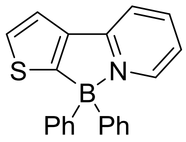

9,9-diphenyl-9H-8l4,9l4-thieno[2',3':3,4][1,2]azaborolo[1,5-a]pyridine, 27. The General procedure B was followed by using 2-(thiophen-3-yl)pyridine (32 mg, 0.2 mmol, 1 equiv), potassium phenyltrifluoroborate (133 mg 0.72 mmol, 3.6 equiv), $SiCl_4$ (0.2 mmol, 24 μl, 1 equiv), *i*Pr_2NEt (1 mmol, 165 μl, 5 equiv) and toluene (1 mL) to afford 27 mg of the product as a white solid (42%). mp 158-159 °C. ^{1}H NMR (500 MHz, Acetone) δ 8.58 (d, *J* = 5.8 Hz, 1H), 8.23 (td, *J* = 8.0, 1.4 Hz, 1H), 8.10 – 8.03 (m, 1H), 7.64-7.61 (m, 2H), 7.49-7.46 (m, 1H), 7.22-7.20 (m, 4H), 7.18 – 7.12 (m, 4H), 7.12 – 7.07 (m, 2H). ^{13}C NMR (126 MHz, Acetone) δ 155.7, 146.2, 143.4, 141.9, 134.1, 132.7, 128.7, 127.0, 121.7, 121.5, 119.5. ^{11}B NMR (160 MHz, Acetone) δ 3.06. HRMS (ESI) m/z calcd for C21H17BNS $[M+H]^+$: 326.1169, found 326.1171.

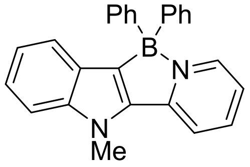

11-methyl-6,6-diphenyl-6,11-dihydro-5l4,6l4-pyrido[1',2':1,5][1,2]azaborolo[4,3-b]indole, 28. The General procedure B was followed by using 1-methyl-2-(pyridin-2-yl)-1H-indole (41 mg, 0.2 mmol, 1 equiv), potassium phenyltrifluoroborate (133 mg 0.72 mmol, 3.6 equiv), $SiCl_4$ (0.2 mmol, 24 μl, 1 equiv), *i*Pr_2NEt (1 mmol, 165 μl, 5 equiv) and toluene (1 mL) to afford 54 mg of the product as a yellow solid (72%). mp247-249 °C. ^{1}H NMR (500 MHz, Acetone, CS_2) δ 8.50 (d, *J* = 5.7 Hz, 1H), 8.08 (t, *J* = 7.8 Hz, 1H), 7.98 (d, *J* = 8.2 Hz, 1H), 7.59 (d, *J* = 7.9 Hz, 1H), 7.33-7.27 (m, 2H), 7.24 – 7.15 (m, 5H), 7.07-7.03 (m, 4H), 7.03 – 6.95 (m, 3H), 4.12 (s, 3H).^{13}C NMR (126 MHz, Acetone, CS_2) δ 153.9, 148.4, 147.0, 143.7, 140.5, 136.3, 131.7, 130.3, 128.4, 127.3, 127.2, 122.6, 121.9, 119.6, 112.8, 33.9. ^{11}B NMR (160 MHz, Acetone, CS_2) δ 2.31. HRMS (ESI) m/z calcd for C26H21BKN2 $[M+K]^+$: 411.1429, found 411.1432.

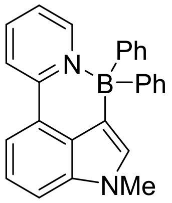

4-methyl-6,6-diphenyl-4,6-dihydro-6l4,7l4-pyrido[1',2':1,6][1,2]azaborinino[3,4,5-cd]indole, 29. The General procedure **B** was followed by using 1-methyl-4-(pyridin-2-yl)-1H-indole (41 mg, 0.2

mmol, 1 equiv), potassium phenyltrifluoroborate (133 mg 0.72 mmol, 3.6 equiv), $SiCl_4$ (0.2 mmol, 24 µl, 1 equiv), *i*Pr_2NEt (1 mmol, 165 µl, 5 equiv) and toluene (1 mL) to afford 52 mg of the product as a yellow solid (70%). mp 235-237 °C. ^{1}H NMR (500 MHz, Acetone, CS2) δ 8.60 (dd, *J* = 9.6, 4.6 Hz, 2H), 8.16-8.12 (m, 1H), 7.81 (d, *J* = 7.5 Hz, 1H), 7.49 – 7.40 (m, 2H), 7.24-7.20 (m, 5H), 7.11-7.08 (m, 4H), 7.05 – 6.97 (m, 2H), 6.81 (s, 1H), 3.80 (s, 3H). ^{13}C NMR (126 MHz, Acetone, CS2) δ 154.8, 150.9, 141.9, 138.4, 135.4, 132.5, 131.3, 128.5, 126.4, 123.9, 123.4, 123.1, 122.7, 116.5, 114.1, 33.6. ^{11}B NMR (160 MHz, Acetone, CS2) δ 2.75. HRMS (ESI) m/z calcd for C26H22BN2 $[M+H]^+$: 373.1871, found 373.1872.

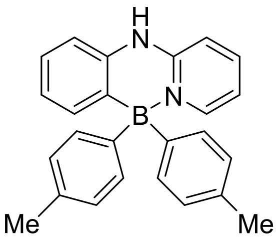

6,6-di-p-tolyl-6,11-dihydro-5l4,6l4-benzo[c]pyrido[2,1-f][1,5,2]diazaborinine, 30. The General procedure A was followed by using *N*-phenylpyridin-2-amine (34 mg, 0.2 mmol, 1 equiv), potassium *p*-tolyltrifluoroborate (95 mg 0.48 mmol, 2.4 equiv), $SiCl_4$ (0.2 mmol, 24 µl, 1 equiv), *i*Pr_2NEt (0.72 mmol, 119 µl, 3.6 equiv) and toluene (1 mL) to afford 51 mg of the product as a white solid (70%). mp 289-290 °C. ^{1}H NMR (500 MHz, acetone) δ 9.50 (s, 1H), 7.78-7.75 (m, 2H), 7.13 (dd, *J* = 9.2, 1.0 Hz, 1H), 7.05 – 6.98 (m, 6H), 6.95 (d, *J* = 7.7 Hz, 4H), 6.92 – 6.87 (m, 1H), 6.86 – 6.79 (m, 2H), 2.23 (s, 6H). ^{13}C NMR (126 MHz, acetone) δ 152.6, 144.6, 141.2, 139.5, 135.2, 135.1, 134.9, 128.9, 126.8, 124.2, 115.3, 114.6, 114.2, 21.7. ^{11}B NMR (160 MHz, acetone) δ -1.23. HRMS (ESI) m/z calcd for C25H24BN2 $[M+H]^+$: 363.2027, found 363.2028.

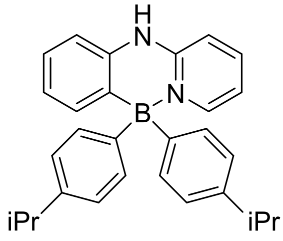

6,6-bis(4-isopropylphenyl)-6,11-dihydro-5l4,6l4-benzo[c]pyrido[2,1-f][1,5,2]diazaborinine, 31. The General procedure **A** was followed by using *N*-phenylpyridin-2-amine (34 mg, 0.2 mmol, 1 equiv), potassium 4-isopropylphenyltrifluoroborate (108 mg 0.48 mmol, 2.4 equiv), $SiCl_4$ (0.2 mmol, 24 µl, 1 equiv), *i*Pr_2NEt (0.72 mmol, 119 µl, 3.6 equiv) and toluene (1 mL) to afford 57 mg of the product as a white solid (68%). mp 257-258 °C. ^{1}H NMR (500 MHz, acetone) δ 9.49 (s, 1H), 7.83 – 7.73 (m, 2H), 7.13 (dd, *J* = 9.3, 1.2 Hz, 1H), 7.07 – 6.99 (m, 10H), 6.93 – 6.86 (m, 1H), 6.86 – 6.78 (m, 2H), 2.85-2.77 (m, 2H), 1.20 (d, *J* = 6.9 Hz, 12H). ^{13}C NMR (126 MHz, acetone) δ 152.6, 146.1, 144.8, 141.2, 139.5, 135.3, 126.8, 126.2, 124.2, 115.4, 114.6,

114.3, 35.0, 25.0, 25.0. ^{11}B NMR (160 MHz, acetone) δ -1.18. HRMS (ESI) m/z calcd for C29H32BN2 $[M+H]^+$: 419.2653, found 419.2654.

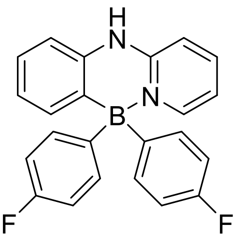

6,6-bis(4-fluorophenyl)-6,11-dihydro-5l4,6l4-benzo[c]pyrido[2,1-f][1,5,2]diazaborinine, 32. The General procedure A was followed by using *N*-phenylpyridin-2-amine (34 mg, 0.2 mmol, 1 equiv), potassium 4-fluorophenyltrifluoroborate (97 mg 0.48 mmol, 2.4 equiv), $SiCl_4$ (0.2 mmol, 24 μl, 1 equiv), *i*Pr_2NEt (0.72 mmol, 119 μl, 3.6 equiv) and toluene (1 mL) to afford 34 mg of the product as a white solid (46%). mp 213-214 °C. ^{1}H NMR (500 MHz, acetone) δ 9.62 (s, 1H), 7.86 – 7.77 (m, 1H), 7.74 (d, J = 6.3 Hz, 1H), 7.18 (d, J = 8.7 Hz, 1H), 7.09-7.05 (m, 5H), 6.98 – 6.84 (m, 8H). ^{13}C NMR (126 MHz, acetone) δ 162.7 (d, J = 240 Hz), 152.6, 144.3, 141.5, 139.5, 136.6 (d, J = 6.8 Hz), 135.0, 127.2, 124.5, 115.8, 114.9, 114.7 (d, J = 18.7 Hz), 114.5. ^{11}B NMR (160 MHz, acetone) δ -1.67. HRMS (ESI) m/z calcd for C23H18BF2N2 $[M+H]^+$: 371.1526, found 371.1526.

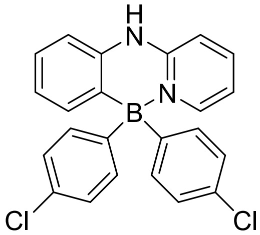

6,6-bis(4-chlorophenyl)-6,11-dihydro-5l4,6l4-benzo[c]pyrido[2,1-f][1,5,2]diazaborinine, 33. The General procedure A was followed by using *N*-phenylpyridin-2-amine (34 mg, 0.2 mmol, 1 equiv), potassium 4-chlorophenyltrifluoroborate (104 mg 0.48 mmol, 2.4 equiv), $SiCl_4$ (0.2 mmol, 24 μl, 1 equiv), *i*Pr_2NEt (0.72 mmol, 119 μl, 3.6 equiv) and toluene (1 mL) to afford 38 mg of the product as a white solid (47%). mp 280-281 °C. ^{1}H NMR (500 MHz, acetone) δ 9.66 (s, 1H), 7.84-7.80 (m, 1H), 7.77 – 7.70 (m, 1H), 7.24 – 7.14 (m, 5H), 7.13 – 7.01 (m, 5H), 7.00 – 6.84 (m, 4H). ^{13}C NMR (126 MHz, acetone) δ 152.5, 144.2, 141.6, 139.4, 136.7, 134.8, 131.9, 128.2, 127.3, 124.5, 115.8, 114.9, 114.54. ^{11}B NMR (160 MHz, acetone) δ -1.74. HRMS (ESI) m/z calcd for C23H17BCl2N2Na $[M+Na]^+$: 425.0754, found 425.0762.

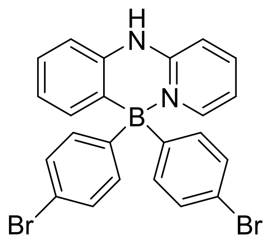

6,6-bis(4-bromophenyl)-6,11-dihydro-5l4,6l4-benzo[c]pyrido[2,1-f][1,5,2]diazaborinine, 34. The General procedure A was followed by using *N*-phenylpyridin-2-amine (34 mg, 0.2 mmol, 1 equiv), potassium 4-bromophenyltrifluoroborate (126 mg 0.48 mmol, 2.4 equiv), $SiCl_4$ (0.2 mmol, 24 µl, 1 equiv), *i*Pr_2NEt (0.72 mmol, 119 µl, 3.6 equiv) and toluene (1 mL) to afford 42 mg of the product as a white solid (43%). mp 283-284 °C. ^1H NMR (500 MHz, acetone, CS_2) δ 9.64 (s, 1H), 7.84-7.81 (m, 1H), 7.72 (d, *J* = 5.3 Hz, 1H), 7.31 (d, *J* = 8.2 Hz, 4H), 7.18 (d, *J* = 8.6 Hz, 1H), 7.10 – 7.03 (m, 1H), 7.02 (d, *J* = 8.2 Hz, 4H), 6.98 – 6.82 (m, 4H). ^{13}C NMR (126 MHz, acetone, CS_2) δ 152.6, 144.3, 141.7, 139.5, 137.3, 135.0, 131.3, 127.5, 124.7, 120.6, 116.0, 115.1, 114.7. ^{11}B NMR (160 MHz, acetone, CS_2) δ -1.76. HRMS (ESI) m/z calcd for C23H18BBr2N2 $[M+H]^+$: 490.9924, found 490.9920.

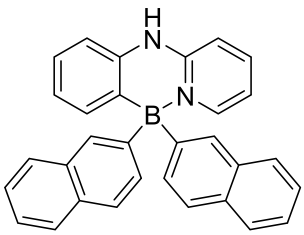

6,6-di(naphthalen-2-yl)-6,11-dihydro-5l4,6l4-benzo[c]pyrido[2,1-f][1,5,2]diazaborinine, 35. The General procedure A was followed by using *N*-phenylpyridin-2-amine (34 mg, 0.2 mmol, 1 equiv), potassium naphthalen-2-yltrifluoroborate (112 mg 0.48 mmol, 2.4 equiv), $SiCl_4$ (0.2 mmol, 24 µl, 1 equiv), *i*Pr_2NEt (0.72 mmol, 119 µl, 3.6 equiv) and toluene (1 mL) to afford 60 mg of the product as a white solid (69%). mp 164-165 °C. ^1H NMR (500 MHz, acetone) δ 9.65 (s, 1H), 7.87 (dd, *J* = 6.4, 1.4 Hz, 1H), 7.83 – 7.74 (m, 3H), 7.71 (d, *J* = 8.3 Hz, 2H), 7.65 – 7.57 (m, 2H), 7.54 – 7.46 (m, 4H), 7.41 – 7.28 (m, 4H), 7.22 (d, *J* = 8.6 Hz, 1H), 7.15 – 7.04 (m, 2H), 6.96 (td, *J* = 7.3, 1.0 Hz, 1H), 6.92 – 6.81 (m, 2H). ^{13}C NMR (126 MHz, acetone) δ 152.7, 144.7, 141.5, 139.7, 135.2, 134.8, 134.0, 133.9, 133.7, 128.9, 128.6, 127.2, 127.1, 126.2, 125.8, 124.5, 115.7, 114.8, 114.5. ^{11}B NMR (160 MHz, acetone) δ -1.03. HRMS (ESI) m/z calcd for C31H24BN2 $[M+H]^+$: 435.2027, found 435.2029.

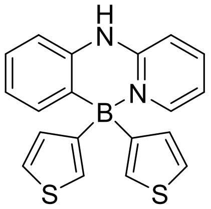

6,6-di(thiophen-3-yl)-6,11-dihydro-5l4,6l4-benzo[c]pyrido[2,1-f][1,5,2]diazaborinine, 36. The General procedure A was followed by using *N*-phenylpyridin-2-amine (34 mg, 0.2 mmol, 1 equiv), potassium thiophen-3-yltrifluoroborate (91 mg 0.48 mmol, 2.4 equiv), $SiCl_4$ (0.2 mmol, 24 μl, 1 equiv), *i*Pr_2NEt (0.72 mmol, 119 μl, 3.6 equiv) and toluene (1 mL) to afford 28 mg of the product as a white solid (40%). mp 264-265 °C. ^{1}H NMR (500 MHz, acetone) δ 9.57 (s, 1H), 7.84 (d, *J* = 6.3 Hz, 1H), 7.80-7.77 (m, 1H), 7.25-7.24 (m, 2H), 7.13 (d, *J* = 8.6 Hz, 1H), 7.07 – 7.00 (m, 2H), 6.96 – 6.81 (m, 5H), 6.76-6.73 (m, 2H). ^{13}C NMR (126 MHz, acetone) δ 152.2, 144.3, 141.4, 139.0, 134.7, 134.1, 127.3, 127.1, 125.0, 124.3, 115.6, 114.7, 114.5. ^{11}B NMR (160 MHz, acetone) δ -4.24. HRMS (ESI) m/z calcd for C19H16BN2S2 $[M+H]^+$: 347.0842, found 347.0844.

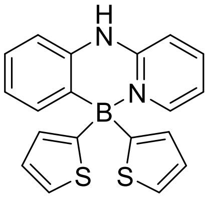

6,6-di(thiophen-2-yl)-6,11-dihydro-5l4,6l4-benzo[c]pyrido[2,1-f][1,5,2]diazaborinine, 37. The General procedure A was followed by using *N*-phenylpyridin-2-amine (34 mg, 0.2 mmol, 1 equiv), potassium thiophen-2-yltrifluoroborate (91 mg 0.48 mmol, 2.4 equiv), $SiCl_4$ (0.2 mmol, 24 μl, 1 equiv), *i*Pr_2NEt (0.72 mmol, 119 μl, 3.6 equiv) and toluene (1 mL) to afford 14 mg of the product as a white solid (19%). mp 226-227 °C. ^{1}H NMR (500 MHz, acetone) δ 9.70 (s, 1H), 8.01 – 7.93 (m, 1H), 7.81-7.78 (m, 1H), 7.34 (dd, *J* = 4.8, 0.9 Hz, 2H), 7.22 – 7.03 (m, 3H), 7.01 – 6.84 (m, 5H), 6.82 (d, *J* = 3.2 Hz, 2H). ^{13}C NMR (126 MHz, acetone) δ 151.7, 144.2, 141.7, 138.7, 135.4, 131.7, 128.1, 127.7, 127.6, 124.4, 115.7, 114.8, 114.6. ^{11}B NMR (160 MHz, acetone) δ -4.61. HRMS (ESI) m/z calcd for C19H16BN2S2 $[M+H]^+$: 347.0842, found 347.0842.

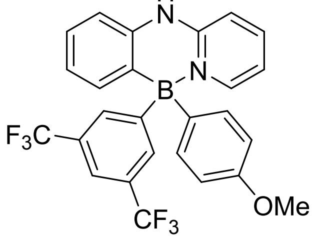

6-(3,5-bis(trifluoromethyl)phenyl)-6-(4-methoxyphenyl)-6,11-dihydro-5l4,6l4-benzo[c]pyrido[2,1-f][1,5,2]diazaborinine, 38. The General procedure **C** was followed by using *N*-phenylpyridin-2-amine (34 mg, 0.2 mmol, 1 equiv), potassium 4-

methoxyphenyltrifluoroborate (51 mg 0.24 mmol, 1.2 equiv), potassium 3,5-bis(trifluoromethyl)trifluoroborate (77 mg 0.24 mmol, 1.2 equiv), $SiCl_4$ (0.2 mmol, 24 µl, 1 equiv), *i*Pr_2NEt (0.72 mmol, 119 µl, 3.6 equiv) and toluene (1 mL) to afford 37 mg of the product as a white solid (37%). mp 221-222 °C. ^{1}H NMR (500 MHz, acetone) δ 9.76 (s, 1H), 7.90-7.86 (m, 1H), 7.79 (d, *J* = 6.4 Hz, 1H), 7.70 (s, 1H), 7.60 (s, 2H), 7.24 (d, *J* = 8.7 Hz, 1H), 7.12-7.09 (m, 1H), 7.06 – 6.93 (m, 6H), 6.90 (d, *J* = 7.8 Hz, 1H), 6.83 – 6.77 (m, 2H), 3.74 (s, 3H). ^{13}C NMR (126 MHz, acetone) δ 159.5, 152.6, 144.0, 141.9, 139.5, 136.5, 134.7, 134.3, 130.6(q, *J* = 31.3 Hz), 127.7, 125.7(q, *J* = 270.0 Hz), 124.7, 119.9 (q, *J* = 3.7 Hz), 116.1, 115.2, 114.5, 114.3, 55.6. ^{11}B NMR (160 MHz, acetone) δ -1.66. HRMS (ESI) m/z calcd for C26H19BF6N2NaO $[M+Na]^+$: 523.1387, found 523.1378.

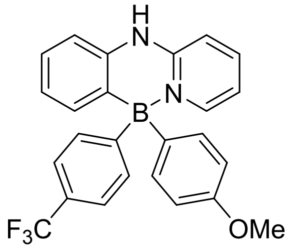

6-(4-methoxyphenyl)-6-(4-(trifluoromethyl)phenyl)-6,11-dihydro-5l4,6l4-benzo[c]pyrido[2,1-f][1,5,2]diazaborinine, 39. The General procedure **C** was followed by using *N*-phenylpyridin-2-amine (34 mg, 0.2 mmol, 1 equiv), potassium 4-methoxyphenyltrifluoroborate (51 mg 0.24 mmol, 1.2 equiv), potassium 4-(trifluoromethyl)phenyl)trifluoroborate (77 mg 0.24 mmol, 1.2 equiv), $SiCl_4$ (0.2 mmol, 24 µl, 1 equiv), *i*Pr_2NEt (0.72 mmol, 119 µl, 3.6 equiv) and toluene (1 mL) to afford 40 mg of the product as a white solid (46%) with 9% isomer. mp 248-250 °C. ^{1}H NMR (500 MHz, acetone) δ 9.63 (s, 1H), 7.84-7.80 (m, 1H), 7.75 (dd, J = 6.4, 1.5 Hz, 1H), 7.44 (d, J = 7.8 Hz, 2H), 7.24 (d, J = 7.7 Hz, 2H), 7.19 (d, J = 8.6 Hz, 1H), 7.07 – 7.03 (m, 3H), 7.01 (dd, *J* = 7.4, 1.4 Hz, 1H), 6.95 (dd, *J* = 7.3, 1.0 Hz, 1H), 6.91 (ddd, J = 13.4, 6.1, 1.1 Hz, 1H), 6.86 (dd, J = 7.8, 0.5 Hz, 1H), 6.79 – 6.76 (m, 2H), 3.73 (s, 3H). 13C NMR (126 MHz, acetone) δ 159.3, 152.6, 144.3, 141.5, 139.5, 136.6, 135.0, 134.8, 127.6 (q, *J* = 31.3 Hz), 127.2, 124.5 (q, *J* = 3.6 Hz), 124.4, 115.7, 114.8, 114.5, 114.0, 55.5. ^{11}B NMR (160 MHz, acetone) δ -1.53. HRMS (ESI) m/z calcd for C25H20BF3N2NaO $[M+Na]^+$: 455.1513, found 455.1521.

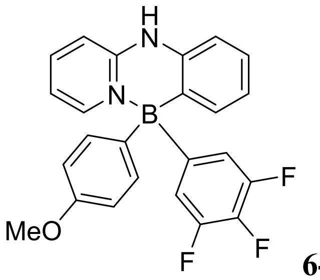

6-(4-methoxyphenyl)-6-(3,4,5-trifluorophenyl)-6,11-dihydro-5l4,6l4-benzo[c]pyrido[2,1-f][1,5,2]diazaborinine, 40. The General procedure **C** was followed by using *N*-phenylpyridin-2-amine (34 mg, 0.2 mmol, 1 equiv), potassium 4-methoxyphenyltrifluoroborate (51 mg 0.24 mmol, 1.2 equiv), potassium 3,4,5-trifluorophenyltrifluoroborate (57 mg 0.24 mmol, 1.2 equiv), $SiCl_4$ (0.2 mmol, 24 µl, 1 equiv), *i*Pr_2NEt (0.72 mmol, 119 µl, 3.6 equiv) and toluene (1 mL) to afford 34 mg of the product as a white solid (41%). mp 202-204 ^oC. ^{1}H NMR (500 MHz, acetone) δ 9.71 (s, 1H), 7.86-7.83 (m, 1H), 7.80 – 7.74 (m, 1H), 7.20 (d, *J* = 8.6 Hz, 1H), 7.09 (ddd, *J* = 7.9, 6.9, 2.1 Hz, 1H), 7.05 – 6.91 (m, 6H), 6.88 (d, *J* = 7.8 Hz, 1H), 6.81 – 6.76 (m, 2H), 6.68 (dd, *J* = 9.3, 7.4 Hz, 2H), 3.73 (s, 3H). ^{13}C NMR (126 MHz, acetone) δ 159.4, 152.5, 151.6 (ddd, *J* = 246.3, 8.7, 2.5 Hz), 144.1, 141.6, 139.4, 138.1 (dt, *J* = 276.3, 15.8 Hz), 136.3, 134.8, 127.5, 124.5, 117.3 (dd, *J* = 13.3, 3.0 Hz), 115.9, 114.9, 114.6, 114.1, 55.5. ^{11}B NMR (160 MHz, acetone) δ -1.97. HRMS (ESI) m/z calcd for C24H18BF3N2NaO $[M+Na]^+$: 441.1356, found 441.1359.

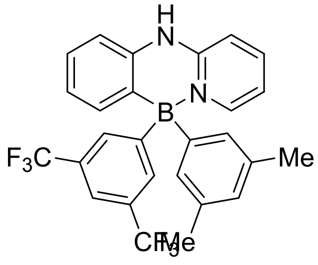

6-(3,5-bis(trifluoromethyl)phenyl)-6-(3,5-dimethylphenyl)-6,11-dihydro-5l4,6l4-benzo[c]pyrido[2,1-f][1,5,2]diazaborinine, 41. The General procedure **C** was followed by using *N*-phenylpyridin-2-amine (34 mg, 0.2 mmol, 1 equiv), potassium 3,5-dimethylphenyltrifluoroborate (51 mg 0.24 mmol, 1.2 equiv), potassium 3,5-bis(trifluoromethyl)trifluoroborate (77 mg 0.24 mmol, 1.2 equiv), $SiCl_4$ (0.2 mmol, 24 µl, 1 equiv), *i*Pr_2NEt (0.72 mmol, 119 µl, 3.6 equiv) and toluene (1 mL) to afford 45 mg of the product as a white solid (45%) with 9% isomer. mp 197-198 ^oC. ^{1}H NMR (500 MHz, acetone) δ 9.72 (s, 1H), 7.90-7.87 (m, 1H), 7.78 (d, *J* = 6.3 Hz, 1H), 7.70 (s, 1H), 7.61 (s, 2H), 7.23 (d, *J* = 8.7 Hz, 1H), 7.12 – 7.06 (m, 1H), 7.00 – 6.93 (m, 3H), 6.89 (d, *J* = 7.8 Hz, 1H), 6.79 (s, 1H), 6.74 (s, 2H), 2.16 (s, 6H). ^{13}C NMR (126 MHz, acetone) δ 152.7, 144.2, 141.9, 139.5, 137.3, 134.8, 134.6, 133.4, 133.3, 130.6 (q, *J* = 31.3 Hz), 128.7, 127.7, 125.7 (q, *J* = 270.0 Hz), 124.8, 120.0 (q, *J* = 3.6 Hz), 116.1, 115.2, 114.8, 22.2. ^{11}B NMR (160 MHz, acetone) δ -1.74. HRMS (ESI) m/z calcd for C27H20BF6N2 $[M-H^+]$: 497.1629, found 497.1637.

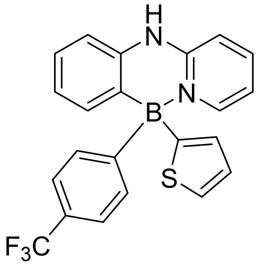

6-(thiophen-2-yl)-6-(4-(trifluoromethyl)phenyl)-6,11-dihydro-5l4,6l4-benzo[c]pyrido[2,1-f][1,5,2]diazaborinine, 42. The General procedure **C** was followed by using *N*-phenylpyridin-2-amine (34 mg, 0.2 mmol, 1 equiv), potassium thiophen-2-yltrifluoroborate (46 mg 0.24 mmol, 1.2 equiv), potassium 4-(trifluoromethyl)phenyl)trifluoroborate (77 mg 0.24 mmol, 1.2 equiv), $SiCl_4$ (0.2 mmol, 24 μl, 1 equiv), *i*Pr_2NEt (0.72 mmol, 119 μl, 3.6 equiv) and toluene (1 mL) to afford 26 mg of the product as a white solid (32%). mp 212-214 °C. ^{1}H NMR (500 MHz, DMSO) δ 10.60 (s, 1H), 7.88 – 7.79 (m, 1H), 7.68 (d, *J* = 6.3 Hz, 1H), 7.47 (d, *J* = 8.1 Hz, 2H), 7.44 (d, *J* = 4.7 Hz, 1H), 7.23 (d, *J* = 7.9 Hz, 2H), 7.14 (d, *J* = 8.7 Hz, 1H), 7.08 (td, *J* = 7.6, 1.5 Hz, 1H), 7.00 (dd, *J* = 4.7, 3.3 Hz, 1H), 6.98 – 6.94 (m, 1H), 6.92-6.85 (m, 3H), 6.66 (d, *J* = 3.2 Hz, 1H). ^{13}C NMR (126 MHz, DMSO) δ 150.2, 142.3, 140.5, 137.2, 133.2, 132.9, 130.7, 127.3, 126.2, 125.7(q, *J* = 31.3), 123.4 (q, *J* =3.7 Hz), 122.9, 114.5, 113.7, 113.4. ^{11}B NMR (160 MHz, DMSO) δ -3.37. HRMS (ESI) m/z calcd for C22H17BF3N2S $[M+H]^+$: 409.1152, found 409.1158.

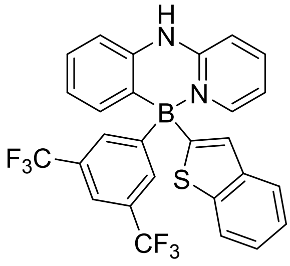

6-(benzo[b]thiophen-2-yl)-6-(3,5-bis(trifluoromethyl)phenyl)-6,11-dihydro-5l4,6l4-benzo[c]pyrido[2,1-f][1,5,2]diazaborinine, 43. The General procedure **C** was followed by using *N*-phenylpyridin-2-amine (34 mg, 0.2 mmol, 1 equiv), potassium benzo[b]thiophen-2-yltrifluoroborate (58 mg 0.24 mmol, 1.2 equiv), potassium 3,5-bis(trifluoromethyl)trifluoroborate (77 mg 0.24 mmol, 1.2 equiv), $SiCl_4$ (0.2 mmol, 24 μl, 1 equiv), *i*Pr_2NEt (0.72 mmol, 119 μl, 3.6 equiv) and toluene (1 mL) to afford 45 mg of the product as a white solid (43%). mp 192-194 °C. ^{1}H NMR (500 MHz, acetone) ^{1}H NMR (500 MHz, Acetone) δ 9.88 (s, 1H), 8.00 – 7.90 (m, 2H), 7.81-7.76 (m, 4H), 7.68 (d, *J* = 7.8 Hz, 1H), 7.30 – 7.23 (m, 2H), 7.22 – 7.18 (m, 1H), 7.16 (dd, *J* = 7.6, 1.3 Hz, 1H), 7.12 (d, *J* = 7.4 Hz, 1H), 7.06 – 6.99 (m, 2H), 6.96 – 6.90 (m, 2H). ^{13}C NMR (126 MHz, Acetone) δ 152.4, 144.4, 143.9, 143.1, 142.3, 139.1, 134.9, 134.4, 130.8(q, *J* = 31.3 Hz), 129.5, 128.3, 125.7 (q, *J* = 270.0 Hz), 125.0, 124.8, 124.30, 124.1, 123.3, 120.6 (q, *J* = 3.6 Hz), 116.5, 115.5, 115.1. ^{11}B NMR (160 MHz, scetone) δ -3.40.HRMS (ESI) m/z calcd for C27H17BF6N2NaS $[M+Na]^+$: 549.1002, found 549.1009.

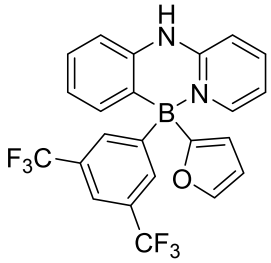

6-(3,5-bis(trifluoromethyl)phenyl)-6-(furan-2-yl)-6,11-dihydro-5l4,6l4-benzo[c]pyrido[2,1-f][1,5,2]diazaborinine, 44. The General procedure **C** was followed by using *N*-phenylpyridin-2-amine (34 mg, 0.2 mmol, 1 equiv), potassium furan-2-yltrifluoroborate (42 mg 0.24 mmol, 1.2 equiv), potassium 3,5-bis(trifluoromethyl)trifluoroborate (77 mg 0.24 mmol, 1.2 equiv), $SiCl_4$ (0.2 mmol, 24 μl, 1 equiv), *i*Pr_2NEt (0.72 mmol, 119 μl, 3.6 equiv) and toluene (1 mL) to afford 25 mg of the product as a white solid (27 %). mp 232-234 °C. ^{1}H NMR (500 MHz, acetone) δ 9.79 (s, 1H), 7.92 – 7.83 (m, 2H), 7.71 (s, 1H), 7.68 (s, 2H), 7.62 – 7.56 (m, 1H), 7.22 (d, *J* = 8.6 Hz, 1H), 7.11 (td, *J* = 7.8, 1.6 Hz, 1H), 7.07 – 7.00 (m, 1H), 6.97 (dtd, *J* = 10.7, 7.1, 1.1 Hz, 2H), 6.91 (d, *J* = 7.9 Hz, 1H), 6.31 (dd, *J* = 3.1, 1.7 Hz, 1H), 6.03 (d, *J* = 3.1 Hz, 1H). ^{13}C NMR (126 MHz, acetone) δ 152.1, 145.3, 144.2, 142.1, 138.9, 134.7, 134.0, 131.1, 130.7 (q, *J* = 31.3 Hz), 128.0, 126.7 (q, *J* = 270.0 Hz), 124.7, 120.2 (q, *J* = 3.6 Hz), 117.0, 116.1, 115.4, 115.1, 110.5. ^{11}B NMR (160 MHz, acetone) δ -4.61. HRMS (ESI) m/z calcd for C23H15BF6N2NaO [M+ Na]+: 483.1074, found 483.1077.

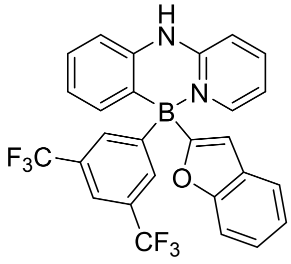

6-(benzofuran-2-yl)-6-(3,5-bis(trifluoromethyl)phenyl)-6,11-dihydro-5l4,6l4-benzo[c]pyrido[2,1-f][1,5,2]diazaborinine, 45. The General procedure **C** was followed by using *N*-phenylpyridin-2-amine (34 mg, 0.2 mmol, 1 equiv), potassium benzofuran-2-yltrifluoroborate (54 mg 0.24 mmol, 1.2 equiv), potassium 3,5-bis(trifluoromethyl)trifluoroborate (77 mg 0.24 mmol, 1.2 equiv), $SiCl_4$ (0.2 mmol, 24 μl, 1 equiv), *i*Pr_2NEt (0.72 mmol, 119 μl, 3.6 equiv) and toluene (1 mL) to afford 37 mg of the product as a white solid (36%). mp 141-142 °C. ^{1}H NMR (500 MHz, acetone) δ 9.89 (s, 1H), 7.99 (d, *J* = 6.3 Hz, 1H), 7.92-7.89 (m, 1H), 7.78 (s, 2H), 7.77 (s, 1H), 7.56 – 7.46 (m, 1H), 7.36 (d, *J* = 8.0 Hz, 1H), 7.26 (d, *J* = 8.7 Hz, 1H), 7.20 – 7.06 (m, 4H), 7.05 – 6.91 (m, 3H), 6.41 (s, 1H). ^{13}C NMR (126 MHz, acetone) δ 158.9, 152.2, 144.3, 142.3, 138.9, 134.7, 134.2, 131.3, 130.9 (q, *J* = 31.3 Hz), 130.7, 128.3, 125.7 (q, *J* = 270.0 Hz), 124.9, 124.2, 123.1, 121.7, 120.5 (q, *J* = 3.6 Hz), 116.3, 115.5, 115.2, 114.0, 112.0. ^{11}B NMR (160 MHz, acetone) δ -4.66. HRMS (ESI) m/z calcd for C27H16BF6N2O [M-H]$^-$: 509.1265, found 509.1266.

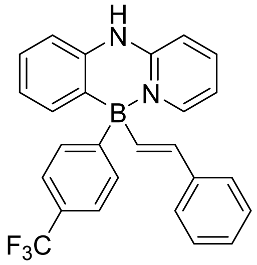

(*E*)-6-styryl-6-(4-(trifluoromethyl)phenyl)-6,11-dihydro-5l4,6l4-benzo[c]pyrido[2,1-f][1,5,2]diazaborinine, 46. The General procedure **C** was followed by using *N*-phenylpyridin-2-amine (34 mg, 0.2 mmol, 1 equiv), potassium *E*-styryltrifluoroborate (51 mg 0.24 mmol, 1.2 equiv), potassium 4-(trifluoromethyl)phenyl)trifluoroborate (77 mg 0.24 mmol, 1.2 equiv), $SiCl_4$ (0.2 mmol, 24 µl, 1 equiv), *i*Pr_2NEt (0.72 mmol, 119 µl, 3.6 equiv) and toluene (1 mL) to afford 34 mg of the product as a white solid (40%). mp 201-203 °C. ^{1}H NMR (500 MHz, acetone) δ 9.62 (s, 1H), 7.92 (d, *J* = 6.3 Hz, 1H), 7.86-7.83 (m, 1H), 7.47 (d, *J* = 8.1 Hz, 2H), 7.43 (d, *J* = 8.0 Hz, 2H), 7.36 (d, *J* = 7.3 Hz, 2H), 7.24 (t, *J* = 7.7 Hz, 2H), 7.18 (d, *J* = 8.7 Hz, 1H), 7.15 – 7.01 (m, 4H), 6.97 – 6.89 (m, 2H), 6.86 (d, *J* = 7.9 Hz, 1H), 6.14 (d, *J* = 17.9 Hz, 1H). ^{13}C NMR (126 MHz, acetone) δ 152.3, 144.5, 141.6, 141.5, 138.9, 137.0, 134.9, 134.7, 129.6, 127.9(d, *J* = 31.3 Hz), 127.5, 127.3, 127.2, 124.74 (q, *J* = 3.7 Hz), 124.5, 115.8, 114.9, 114.8. ^{11}B NMR (160 MHz, acetone) δ -3.23 (s). HRMS (ESI) m/z calcd for C26H21BF3N2 $[M+H]^+$: 429.1744, found 429.1747.

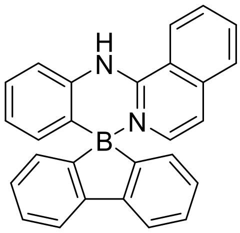

13H-7l4,8l4-spiro[benzo[3,4][1,5,2]diazaborinino[6,1-a]isoquinoline-8,5'-dibenzo[b,d]borole], 47. The general procedure **A** was followed by using *N*-phenylisoquinolin-1-amine (44 mg, 0.2 mmol, 1 equiv), 2,2'-bis(trifluoro-l4-boranyl)-1,1'-biphenyl, dipotassium salt (88 mg 0.24 mmol, 1.2 equiv), $SiCl_4$ (0.2 mmol, 24 µl, 1 equiv), *i*Pr_2NEt (0.72 mmol, 119 µl, 3.6 equiv) and toluene (1 mL) to afford 49 mg of the product as a white solid (64%). mp 286-288 °C. ^{1}H NMR (500 MHz, acetone, CS_2) δ 9.89 (s, 1H), 8.73 (d, *J* = 8.5 Hz, 1H), 7.87 – 7.70 (m, 3H), 7.70 – 7.60 (m, 2H), 7.24 (t, *J* = 8.0 Hz, 3H), 7.16 (td, *J* = 7.5, 1.1 Hz, 2H), 7.06 – 6.93 (m, 4H), 6.90 (d, *J* = 7.0 Hz, 1H), 6.75 (td, *J* = 7.3, 0.9 Hz, 1H), 6.57 (dd, *J* = 7.3, 1.2 Hz, 1H). ^{13}C NMR (126 MHz, acetone, CS_2) δ 151.1, 150.3, 140.0, 138.6, 137.3, 134.9, 134.7, 132.9, 130.1, 129.6, 128.6, 128.5, 127.8, 126.1, 125.5, 121.0, 117.0, 114.8. ^{11}B NMR (160 MHz, acetone, CS_2) δ -1.87. HRMS (ESI) m/z calcd for C27H20BN2 $[M+H]^+$: 383.1714, found 383.1712.

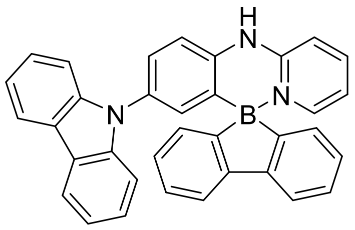

8-(9H-carbazol-9-yl)-11H-5l4,6l4-spiro[benzo[c]pyrido[2,1-f][1,5,2]diazaborinine-6,5'-dibenzo[b,d]borole], 48. The general procedure **A** was followed by using *N*-(4-(9H-carbazol-9-yl)phenyl)pyridin-2-amine (67 mg, 0.2 mmol, 1 equiv), 2,2'-bis(trifluoro-l4-boranyl)-1,1'-biphenyl, dipotassium salt (88 mg 0.24 mmol, 1.2 equiv), $SiCl_4$ (0.2 mmol, 24 µl, 1 equiv), *i*Pr_2NEt (0.72 mmol, 119 µl, 3.6 equiv) and toluene (1 mL) to afford 60 mg of the product as a white solid (60%). mp 109-110 °C. ^{1}H NMR (500 MHz, acetone, CS_2) δ 9.96 (s, 1H), 8.08 – 7.97 (m, 2H), 7.83 – 7.69 (m, 1H), 7.58 (d, J = 7.5 Hz, 2H), 7.37 (d, J = 7.0 Hz, 2H), 7.33 – 7.28 (m, 1H), 7.28 – 7.18 (m, 5H), 7.18 – 7.10 (m, 6H), 7.03 (t, J = 7.2 Hz, 2H), 6.76 (d, J = 1.7 Hz, 1H), 6.67 (t, J = 6.7 Hz, 1H). ^{13}C NMR (126 MHz, acetone, CS_2) δ 152.9, 150.1, 143.8, 142.5, 142.1, 139.5, 134.0, 132.5, 132.4, 128.6, 128.3, 127.5, 125.8, 124.8, 121.8, 121.3, 120.9, 116.8, 116.6, 115.8, 111.6. ^{11}B NMR (160 MHz, acetone, CS_2) δ -1.83. HRMS (ESI) m/z calcd for C35H25BN3 $[M+H]^+$: 498.2136, found 498.2137.

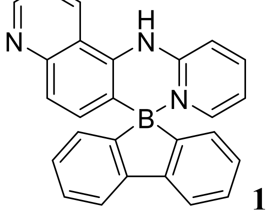

13'H-5l4,8'l4-spiro[dibenzo[b,d]borole-5,7'-pyrido[1',2':1,6][1,5,2]diazaborinino[4,3-f]quinoline], 49. The general procedure **A** was followed by using *N*-(pyridin-2-yl)quinolin-5-amine (44 mg, 0.2 mmol, 1 equiv), 2,2'-bis(trifluoro-l4-boranyl)-1,1'-biphenyl, dipotassium salt (88 mg 0.24 mmol, 1.2 equiv), $SiCl_4$ (0.2 mmol, 24 µl, 1 equiv), *i*Pr_2NEt (0.72 mmol, 119 µl, 3.6 equiv) and toluene (1 mL) to afford 57 mg of the product as a white solid (74%). mp >295 °C. ^{1}H NMR (500 MHz, DMSO) δ 10.41 (s, 1H), 8.96 (d, J = 8.6 Hz, 1H), 8.81 (d, J = 4.0 Hz, 1H), 7.84 (t, J = 7.8 Hz, 1H), 7.73 (d, J = 7.8 Hz, 2H), 7.65 (s, 1H), 7.58 (dd, J = 8.6, 4.1 Hz, 1H), 7.37 (d, J = 8.3 Hz, 1H), 7.24 – 7.13 (m, 5H), 6.98 (t, J = 7.2 Hz, 2H), 6.80 – 6.68 (m, 2H). ^{13}C NMR (126 MHz, DMSO) δ 160.6, 150.8, 148.9, 147.7, 147.4, 141.1, 140.5, 134.0, 132.5, 130.4, 128.7, 126.8, 126.5, 122.9, 120.2, 119.0, 116.8, 115.6, 114.9. ^{11}B NMR (160 MHz, DMSO) δ -1.82. HRMS (ESI) m/z calcd for C26H19BN3 $[M+H]^+$: 384.1667, found 384.1669.

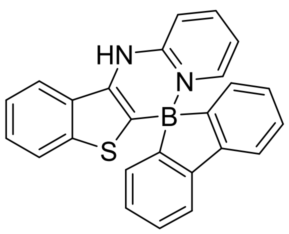

12H-6l4,7l4-spiro[benzo[4,5]thieno[2,3-c]pyrido[2,1-f][1,5,2]diazaborinine-6,5'-dibenzo[b,d]borole], 50. The general procedure **A** was followed by using *N*-(benzo[b]thiophen-3-yl)pyridin-2-amine (45 mg, 0.2 mmol, 1 equiv), 2,2'-bis(trifluoro-l4-boranyl)-1,1'-biphenyl, dipotassium salt (88 mg 0.24 mmol, 1.2 equiv), $SiCl_4$ (0.2 mmol, 24 µl, 1 equiv), *i*Pr_2NEt (0.72 mmol, 119 µl, 3.6 equiv) and toluene (1 mL) to afford 55 mg of the product as a yellow solid (71%). mp 159-160 °C. ^1H NMR (500 MHz, acetone, CS_2) δ 10.08 (s, 1H), 7.94 – 7.88 (m, 1H), 7.76-7.72 (m, 1H), 7.72 – 7.65 (m, 3H), 7.38 – 7.31 (m, 2H), 7.26 (d, *J* = 7.0 Hz, 2H), 7.24 – 7.14 (m, 4H), 7.00 (td, *J* = 7.2, 1.0 Hz, 2H), 6.67 (td, *J* = 6.7, 1.2 Hz, 1H). ^{13}C NMR (126 MHz, acetone, CS_2) δ 152.4, 149.9, 143.5, 143.3, 141.4, 133.8, 132.6, 130.2, 128.8, 128.3, 125.1, 124.7, 124.3, 120.8, 119.9, 116.5, 116.3. ^{11}B NMR (160 MHz, acetone, CS_2) δ -1.43. HRMS (ESI) m/z calcd for C25H18BN2S $[M+H]^+$: 389.1278, found 389.1278.

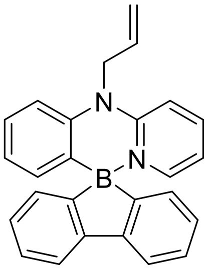

11-allyl-11H-5l4,6l4-spiro[benzo[c]pyrido[2,1-f][1,5,2]diazaborinine-6,5'-dibenzo[b,d]borole], 51. The general procedure **A** was followed by using *N*-allyl-*N*-phenylpyridin-2-amine (42 mg, 0.2 mmol, 1 equiv), 2,2'-bis(trifluoro-l4-boranyl)-1,1'-biphenyl, dipotassium salt (88 mg 0.24 mmol, 1.2 equiv), $SiCl_4$ (0.2 mmol, 24 µl, 1 equiv), *i*Pr_2NEt (0.72 mmol, 119 µl, 3.6 equiv) and toluene (1 mL) to afford 54 mg of the product as a white solid (73%). mp 191-192 °C. ^1H NMR (500 MHz, acetone, CS_2) δ 7.84-7.78 (m, 1H), 7.66 (d, *J* = 7.5 Hz, 2H), 7.63 (dd, *J* = 6.2, 1.7 Hz, 1H), 7.30-7.26 (m, 3H), 7.17 (td, *J* = 7.5, 1.1 Hz, 2H), 7.13-7.06 (m, 2H), 7.03-6.97 (dd, *J* = 10.4, 4.0 Hz, 2H), 6.83 – 6.76 (m, 1H), 6.75 – 6.67 (m, 2H), 6.30 – 6.20 (m, 1H), 5.45 (dd, *J* = 10.7, 0.8 Hz, 1H), 5.33 (dd, *J* = 17.4, 0.8 Hz, 1H), 4.90-4.86 (m, 2H). ^{13}C NMR (126 MHz, acetone, CS_2) δ 154.0, 150.4, 143.8, 143.7, 142.7, 133.8, 133.7, 132.4, 128.6, 128.3, 128.0, 125.3, 120.9, 118.6, 117.2, 115.8, 115.4, 53.7. ^{11}B NMR (160 MHz, acetone, CS_2) δ -1.88. HRMS (ESI) m/z calcd for C26H22BN2 $[M+H]^+$: 373.1871, found 373.1871.

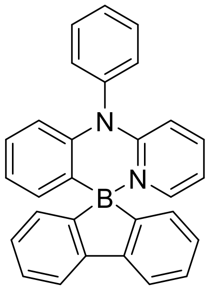

11-phenyl-11H-5l4,6l4-spiro[benzo[c]pyrido[2,1-f][1,5,2]diazaborinine-6,5'-dibenzo[b,d]borole], 52. The general procedure **A** was followed by using *N,N*-diphenylpyridin-2-amine (49 mg, 0.2 mmol, 1 equiv), 2,2'-bis(trifluoro-l4-boranyl)-1,1'-biphenyl, dipotassium salt (88 mg 0.24 mmol, 1.2 equiv), $SiCl_4$ (0.2 mmol, 24 µl, 1 equiv), *i*Pr_2NEt (0.72 mmol, 119 µl, 3.6 equiv) and toluene (1 mL) to afford 62 mg of the product as a white solid (76%). mp 279-280 °C. ^1H NMR (500 MHz, acetone, CS_2) δ 7.85 (dd, *J* = 10.6, 4.9 Hz, 2H), 7.73 (t, *J* = 7.5 Hz, 1H), 7.69 – 7.59 (m, 5H), 7.50 (dd, *J* = 6.3, 1.5 Hz, 1H), 7.36 (d, *J* = 7.0 Hz, 2H), 7.18 (td, *J* = 7.4, 1.1 Hz, 2H), 7.03 (t, *J* = 7.2 Hz, 2H), 6.88 – 6.81 (m, 1H), 6.75-6.69 (m, 2H), 6.64 (dd, *J* = 7.3, 1.5 Hz, 1H), 6.58 (d, *J* = 9.0 Hz, 1H), 6.23 (d, *J* = 8.3 Hz, 1H). ^{13}C NMR (126 MHz, acetone, CS_2) δ 154.1, 150.4, 144.5, 144.4, 142.2, 141.9, 134.5, 133.7, 132.7, 132.4, 131.6, 128.8, 128.6), 127.7, 125.5, 121.1, 117.1, 116.9, 116.1. ^{11}B NMR (160 MHz, acetone, CS_2) δ -1.80. HRMS (ESI) m/z calcd for C29H22BN2 $[M+ H]^+$: 409.1871, found 409.1873.

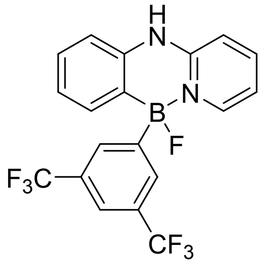

6-(3,5-bis(trifluoromethyl)phenyl)-6-fluoro-6,11-dihydro-5l4,6l4-benzo[c]pyrido[2,1-f][1,5,2]diazaborinine, **54.** White solid. mp 211-213 °C. ^1H NMR (500 MHz, Acetone) δ 10.18 (s, 1H), 7.98 (d, *J* = 6.4 Hz, 1H), 7.95 – 7.85 (m, 3H), 7.75 (s, 1H), 7.29 (d, *J* = 8.8 Hz, 1H), 7.26 – 7.19 (m, 2H), 7.06 (d, *J* = 7.9 Hz, 1H), 7.02-6.96 (m, 2H). ^{13}C NMR (126 MHz, Acetone) δ 150.7, 142.1, 141.7 (d, *J* = 2.2 Hz), 138.6 (d, *J* = 3.4 Hz), 134.0, 132.4, 131.08 (q, *J* = 31.7 Hz), 129.0, 125.5 (q, *J* = 270.3Hz), 124.6, 120.8 (m), 116.0, 115.5, 115.4. ^{19}F NMR (471 MHz, Acetone) δ -63.1, -169.0. ^{11}B NMR (160 MHz, Acetone) δ 3.51. HRMS (ESI) m/z calcd for C19H12BF7N2Na $[M+Na^+]$: 435.0874, found 435.0875.

Crystal structure of compound 3

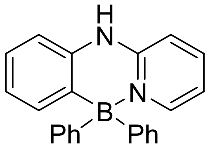

(CCDC 1814302)

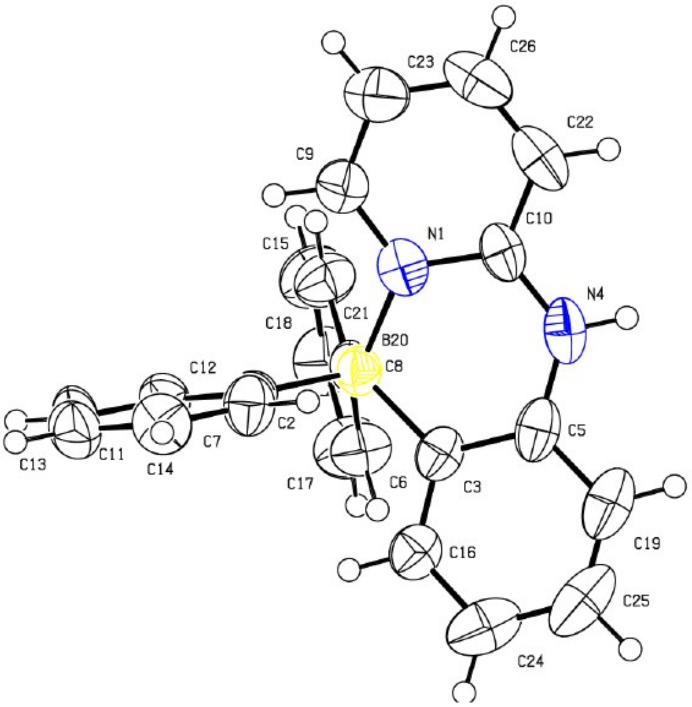

Bond precision: C-C = 0.0047 A Wavelength=0.71073

Cell: a=9.5765(11) b=10.1909(12) c=10.8774(16)

alpha=115.915(13) beta=106.476(11) gamma=90.377(10)

Temperature: 294 K

	Calculated	Reported
**Volume**	905.1(2)	905.1(2)
**Space group**	P -1	P -1
**Hall group**	-P 1	-P 1
**Moiety formula**	C23 H19 B N2	C23 H19 B N2
**Sum formula**	C23 H19 B N2	C23 H19 B N2
**Mr**	334.21	334.24
**Dx,g cm-3**	1.226	1.226
**Z**	2	2
**Mu (mm-1)**	0.071	0.071
**F000**	352.0	352.1
**F000'**	352.12	
**h,k,lmax**	11,12,12	11,12,12
**Nref**	3183	3178
**Tmin,Tmax**		0.630, 1.000
**Tmin'**		

Correction method= # Reported T Limits: Tmin=0.630 Tmax=1.000

AbsCorr = MULTI-SCAN

Data completeness= 0.998 Theta(max)= 25.000

R(reflections)= 0.0612(1860) wR2(reflections)= 0.1903(3178)

S = 1.057 Npar= 234

Crystal structure of compound 4

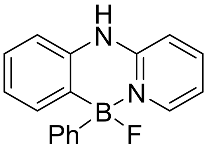

(CCDC 1817506)

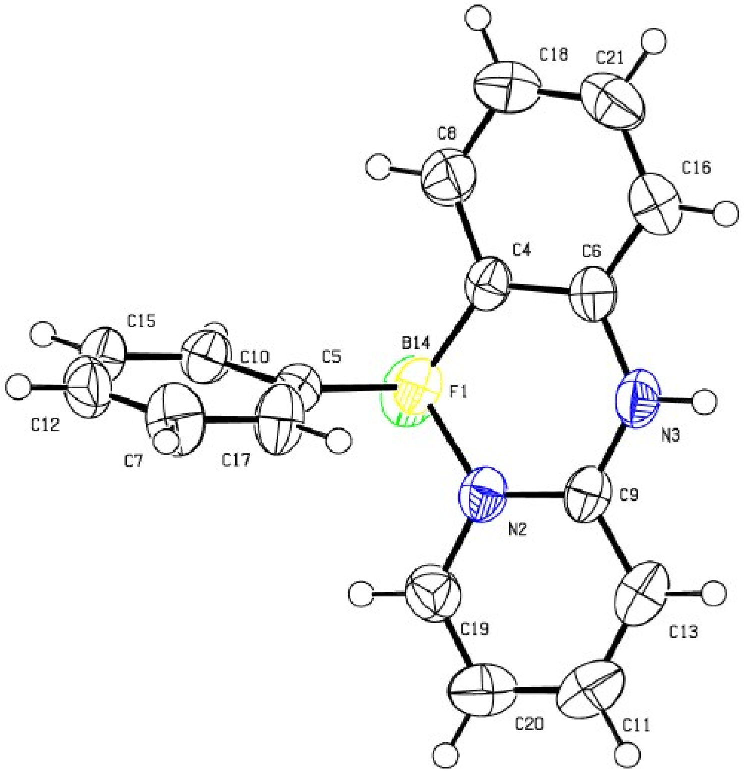

Bond precision: C-C = 0.0036A Wavelength=0.71073

Cell: a=9.1839(14) b=11.2820(14) c=14.148(3)

alpha=90 beta=101.510(17) gamma=90

Temperature: 296 K

	Calculated	Reported
**Volume**	1436.4(4)	1436.5(4)
**Space group**	P 21/n	P 1 21/n 1

**Hall group**	-P 2yn	-P 2yn
**Moiety formula**	C17 H14 B F N2	C17 H14 B F N2
**Sum formula**	C17 H14 B F N2	C17 H14 B F N2
**Mr**	276.11	276.14
**Dx,g cm-3**	1.277	1.277
**Z**	4	4
**Mu (mm-1)**	0.084	0.084
**F000**	576.0	576.3
**F000'**	576.24	
**h,k,lmax**	10,13,16	10,13,16
**Nref**	2531	2524
**Tmin,Tmax**		0.585,1.000
**Tmin'**		

Correction method= # Reported T Limits: Tmin=0.585 Tmax=1.000

AbsCorr = MULTI-SCAN

Data completeness= 0.997 Theta(max)= 25.000

R(reflections)= 0.0502(1605) wR2(reflections)= 0.1224(2524)

S = 1.022 Npar= 189

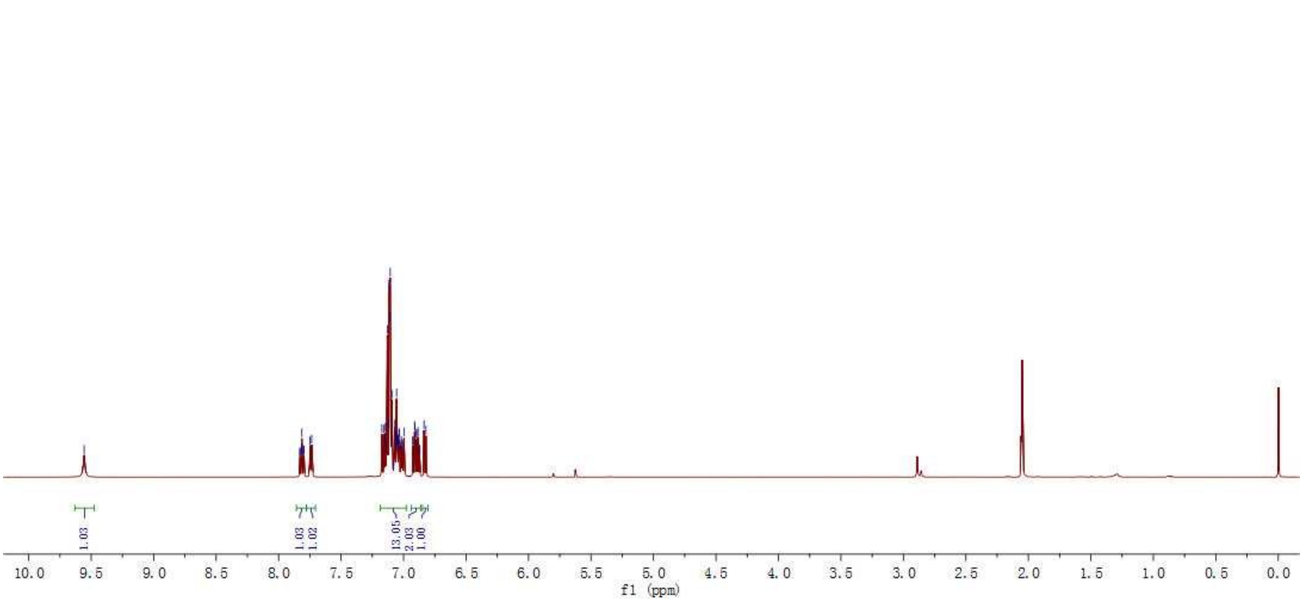

9.56
7.83 7.83 7.82 7.81 7.80 7.80 7.75 7.74 7.17 7.16 7.15 7.14 7.14 7.13 7.12 7.12 7.11 7.11 7.09 7.08 7.07 7.07 7.06 7.06 7.05 7.05 7.05 7.05 7.04 7.03 7.02 7.02 7.01 7.00 6.93 6.92 6.91 6.91 6.90 6.90 6.88 6.87 6.84 6.82
1.03 1.03 1.02 13.05 2.03 1.00
10.0 9.5 9.0 8.5 8.0 7.5 7.0 6.5 6.0 5.5 5.0 4.5 4.0 3.5 3.0 2.5 2.0 1.5 1.0 0.5 0.0
f1 (ppm)

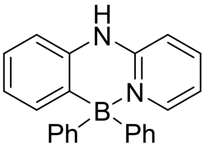

H
N
N
B
Ph
Ph

3

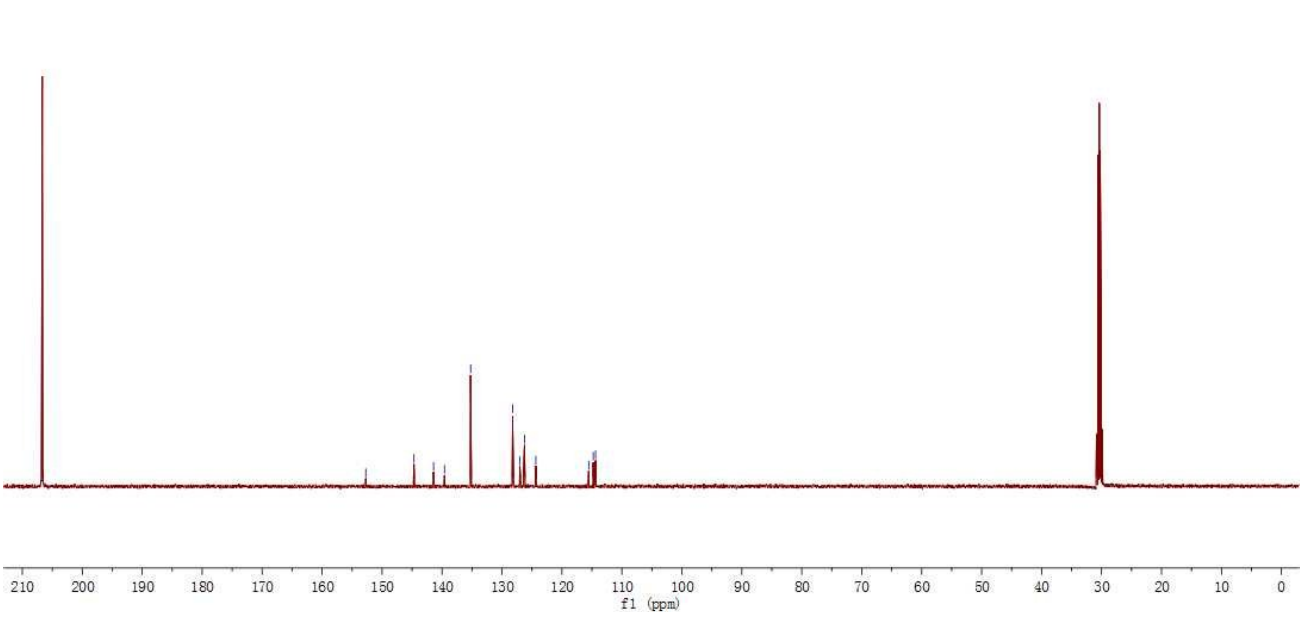

152.72
144.68 141.41 139.59
135.23
128.23 127.02 126.24 124.35
115.53 114.78 114.39
210 200 190 180 170 160 150 140 130 120 110 100 90 80 70 60 50 40 30 20 10 0
f1 (ppm)

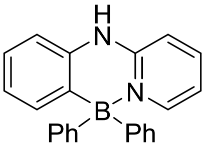

H
N
N
B
Ph
Ph

3

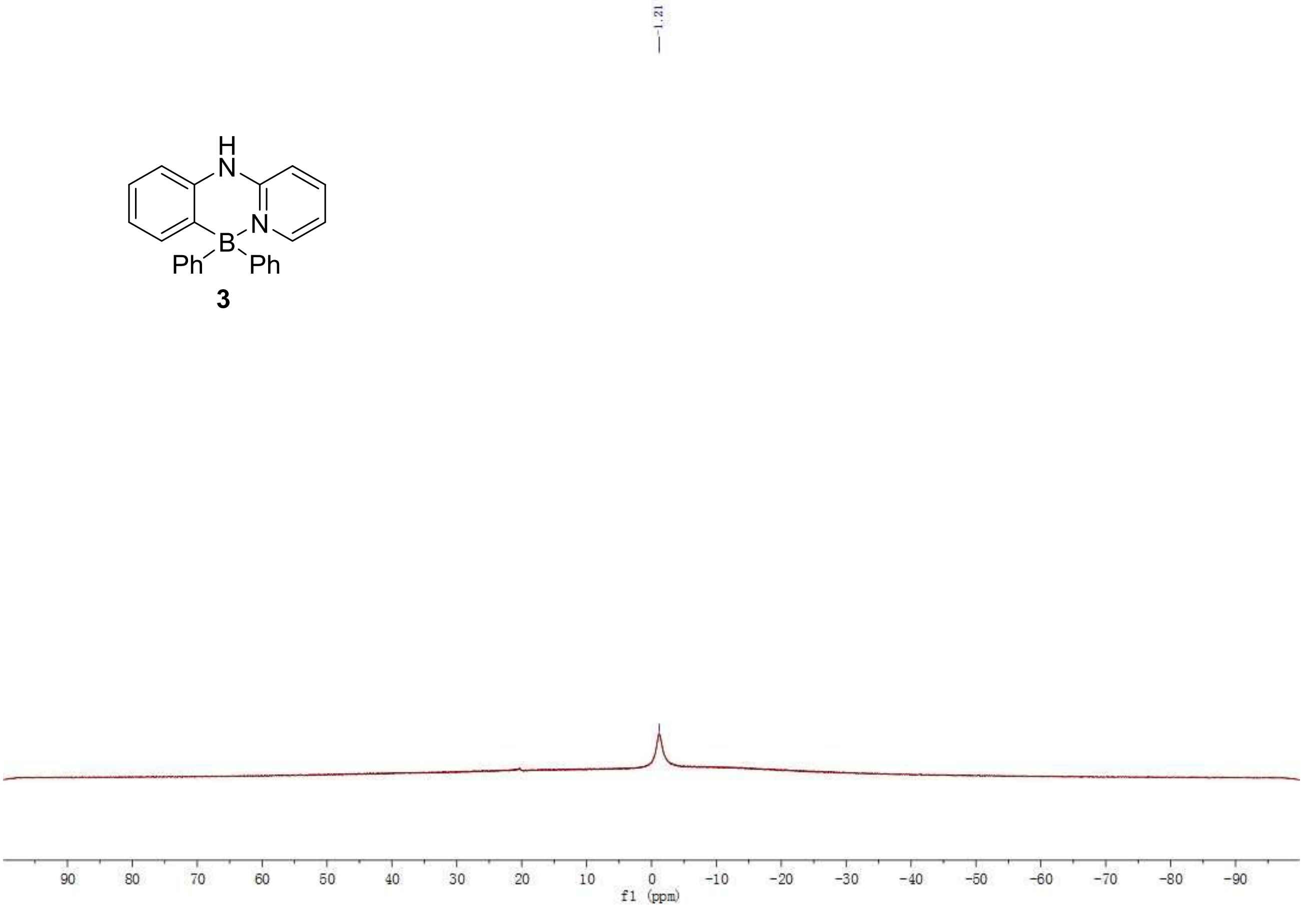

1.21
H
N
N
B
Ph
Ph
3
90 80 70 60 50 40 30 20 10 0 -10 -20 -30 -40 -50 -60 -70 -80 -90
f1 (ppm)

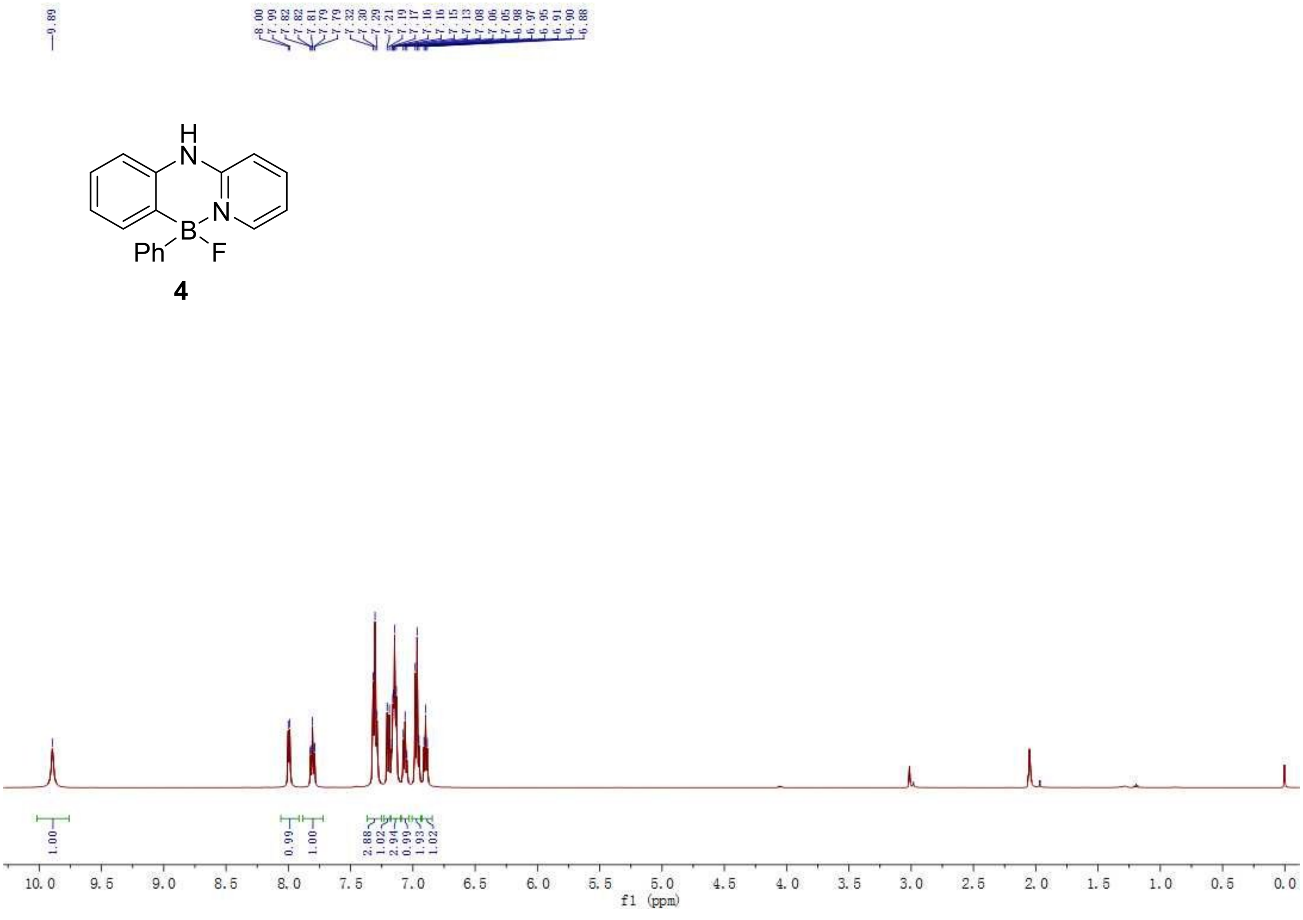

9.89
8.00 7.99 7.82 7.82 7.81 7.79 7.32 7.30 7.29 7.21 7.19 7.17 7.16 7.16 7.15 7.13 7.08 7.06 7.05 6.98 6.97 6.95 6.91 6.90 6.88
H
N
N
B
Ph
F
4
1.00
0.99
1.00
2.88
1.02
2.94
0.99
1.93
1.02
10.0 9.5 9.0 8.5 8.0 7.5 7.0 6.5 6.0 5.5 5.0 4.5 4.0 3.5 3.0 2.5 2.0 1.5 1.0 0.5 0.0
f1 (ppm)

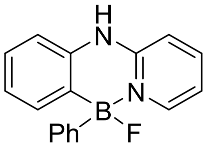
H
N
B
N
Ph
F

4

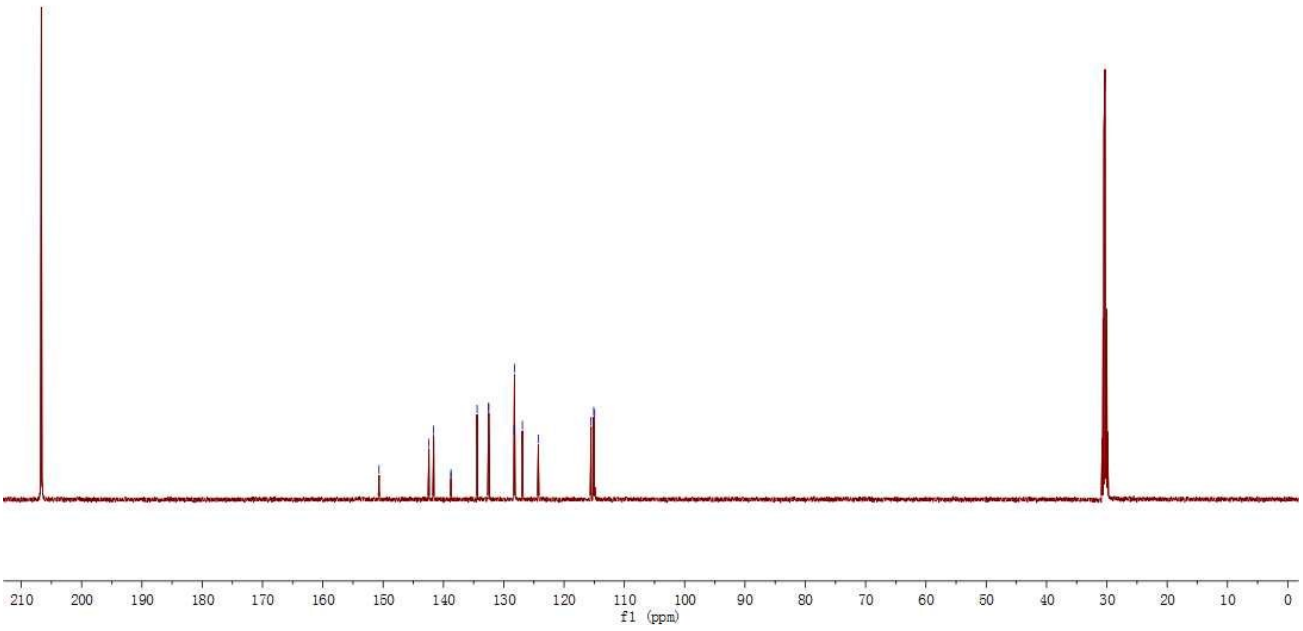
150.69
142.41
141.62
138.78
138.75
134.42
132.52
132.49
128.28
128.23
126.91
124.27
115.53
115.08
114.93
210 200 190 180 170 160 150 140 130 120 110 100 90 80 70 60 50 40 30 20 10 0
f1 (ppm)

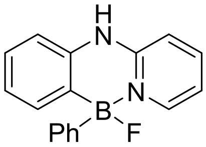
H
N
B
N
Ph
F

4

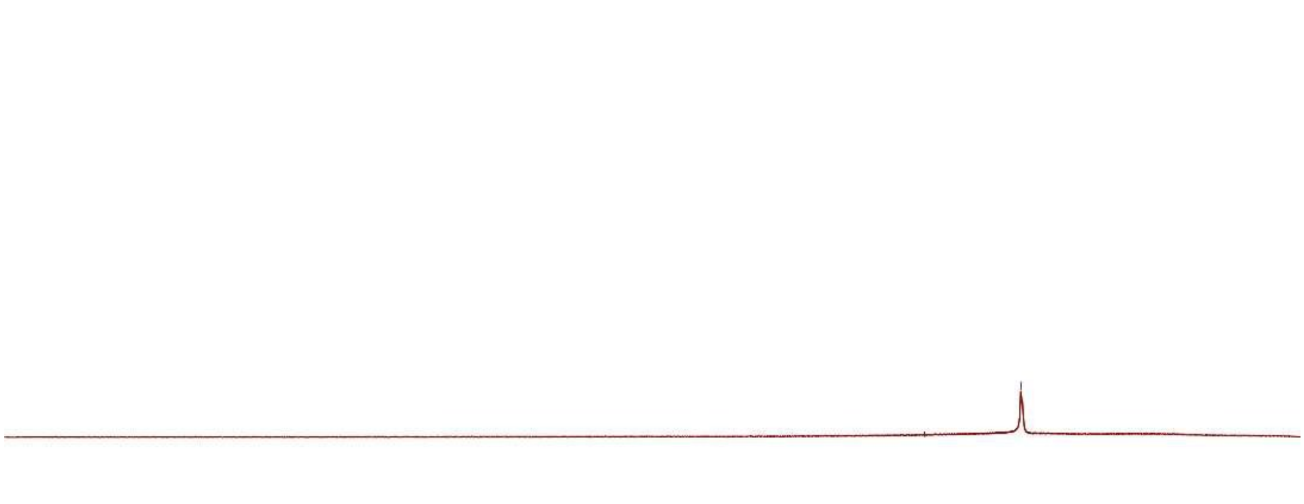
-168.72
10 0 -10 -20 -30 -40 -50 -60 -70 -80 -90 -100 -110 -120 -130 -140 -150 -160 -170 -180 -190 -200 -210
f1 (ppm)

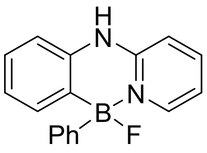

H
N
N
B
Ph
F

4

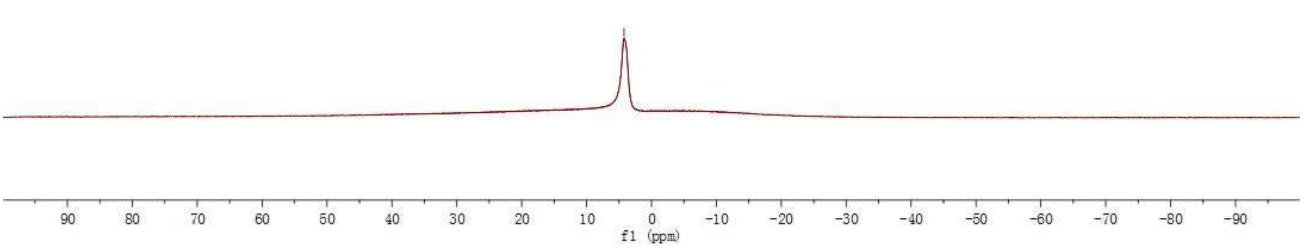

4.23
f1 (ppm)

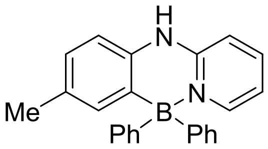

H
N
N
Me
B
Ph
Ph

5

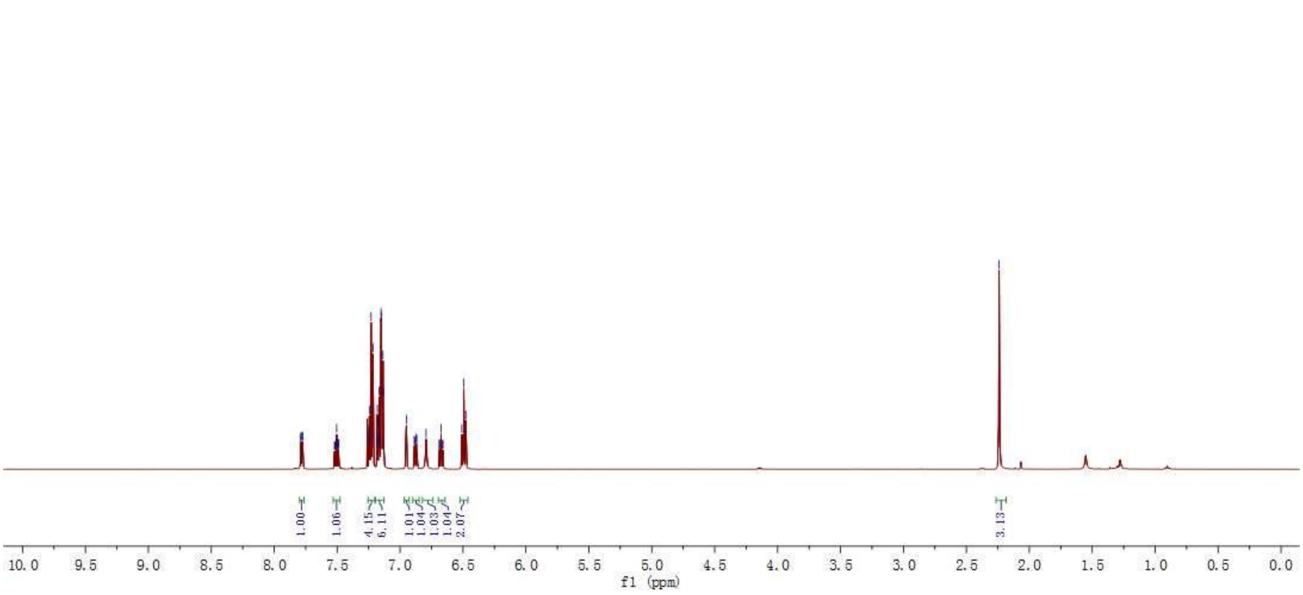

2.24
1.00
1.06
4.15
6.11
1.01
1.04
1.03
1.04
2.07
3.13
f1 (ppm)

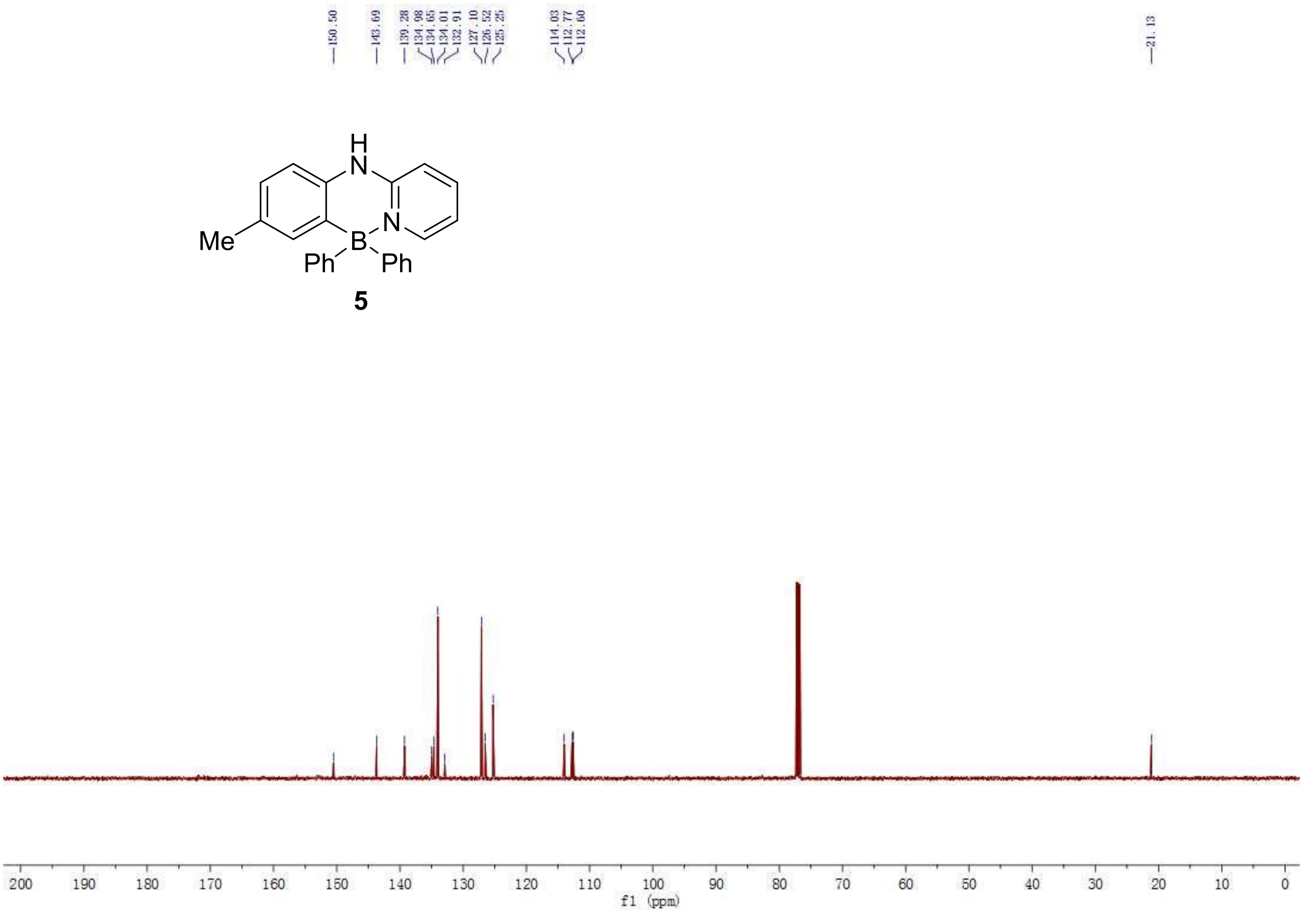

150.50
143.69
139.28
134.98
134.65
134.01
132.91
127.10
126.52
125.25
114.03
112.77
112.60
21.13
H
N
N
B
Me
Ph
Ph
5
200 190 180 170 160 150 140 130 120 110 100 90 80 70 60 50 40 30 20 10 0
f1 (ppm)

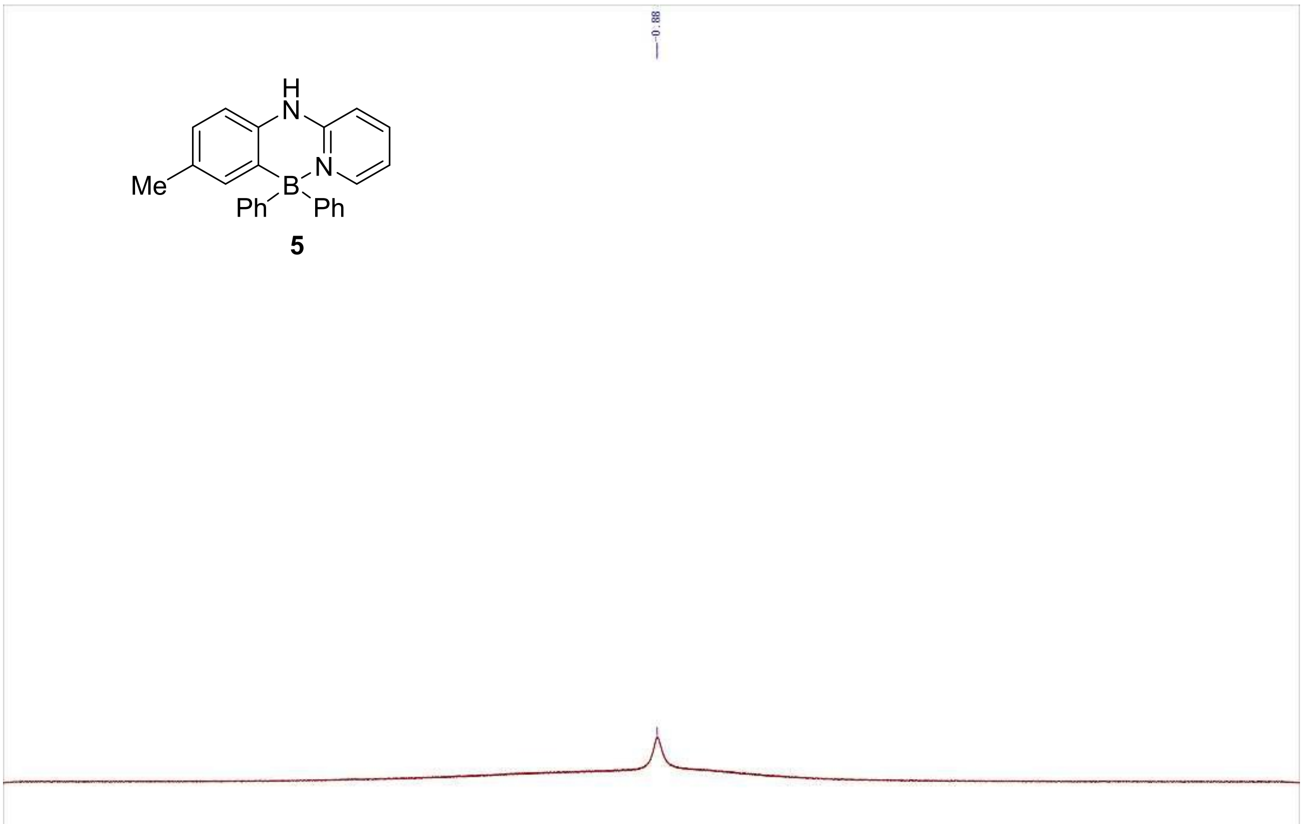

-0.88
H
N
N
B
Me
Ph
Ph
5
90 80 70 60 50 40 30 20 10 0 -10 -20 -30 -40 -50 -60 -70 -80 -90
f1 (ppm)

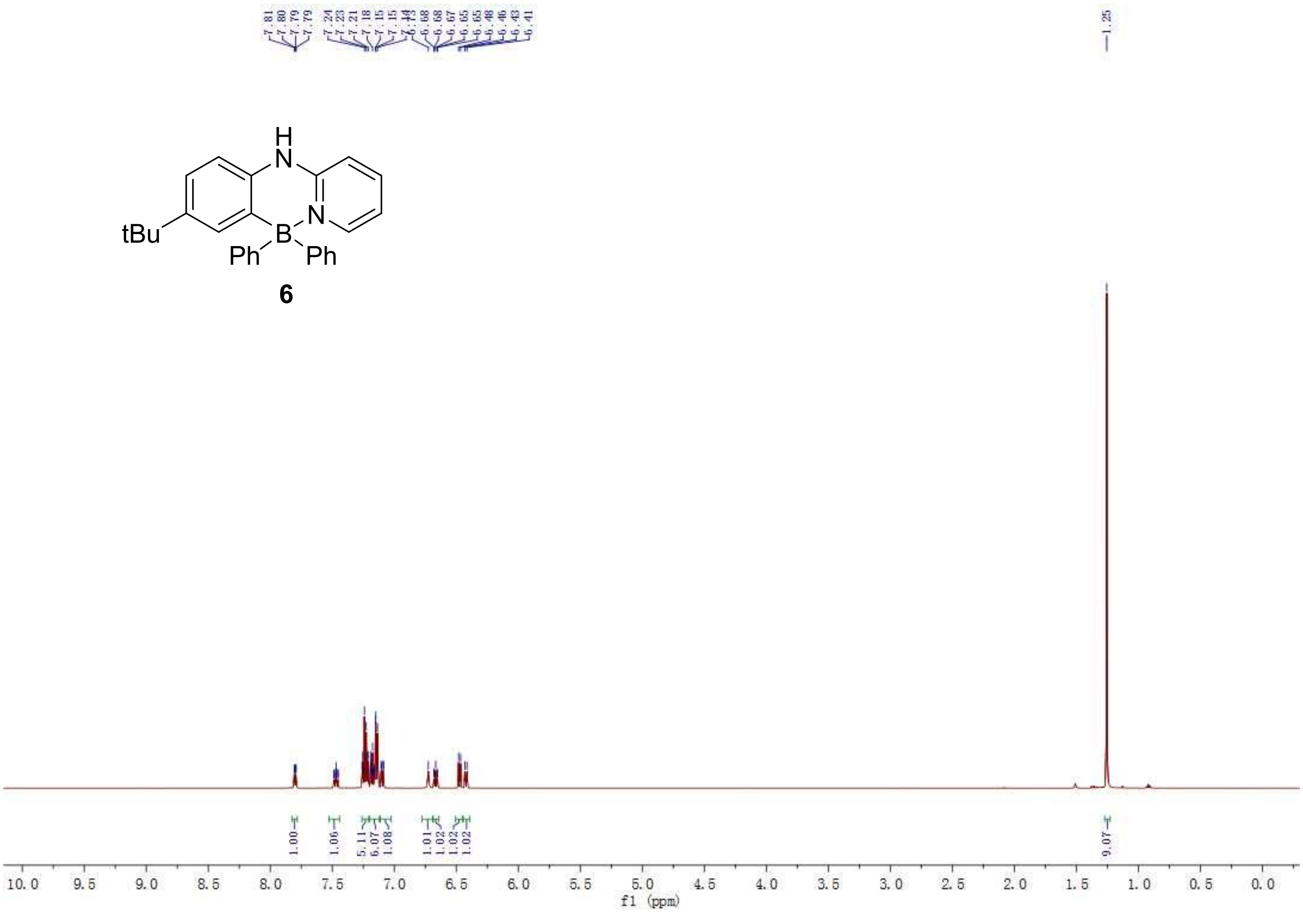
1.25
tBu
Ph
Ph
6
1.00
1.06
5.11
6.07
1.08
1.01
1.02
1.02
1.02
9.07
10.0 9.5 9.0 8.5 8.0 7.5 7.0 6.5 6.0 5.5 5.0 4.5 4.0 3.5 3.0 2.5 2.0 1.5 1.0 0.5 0.0
f1 (ppm)

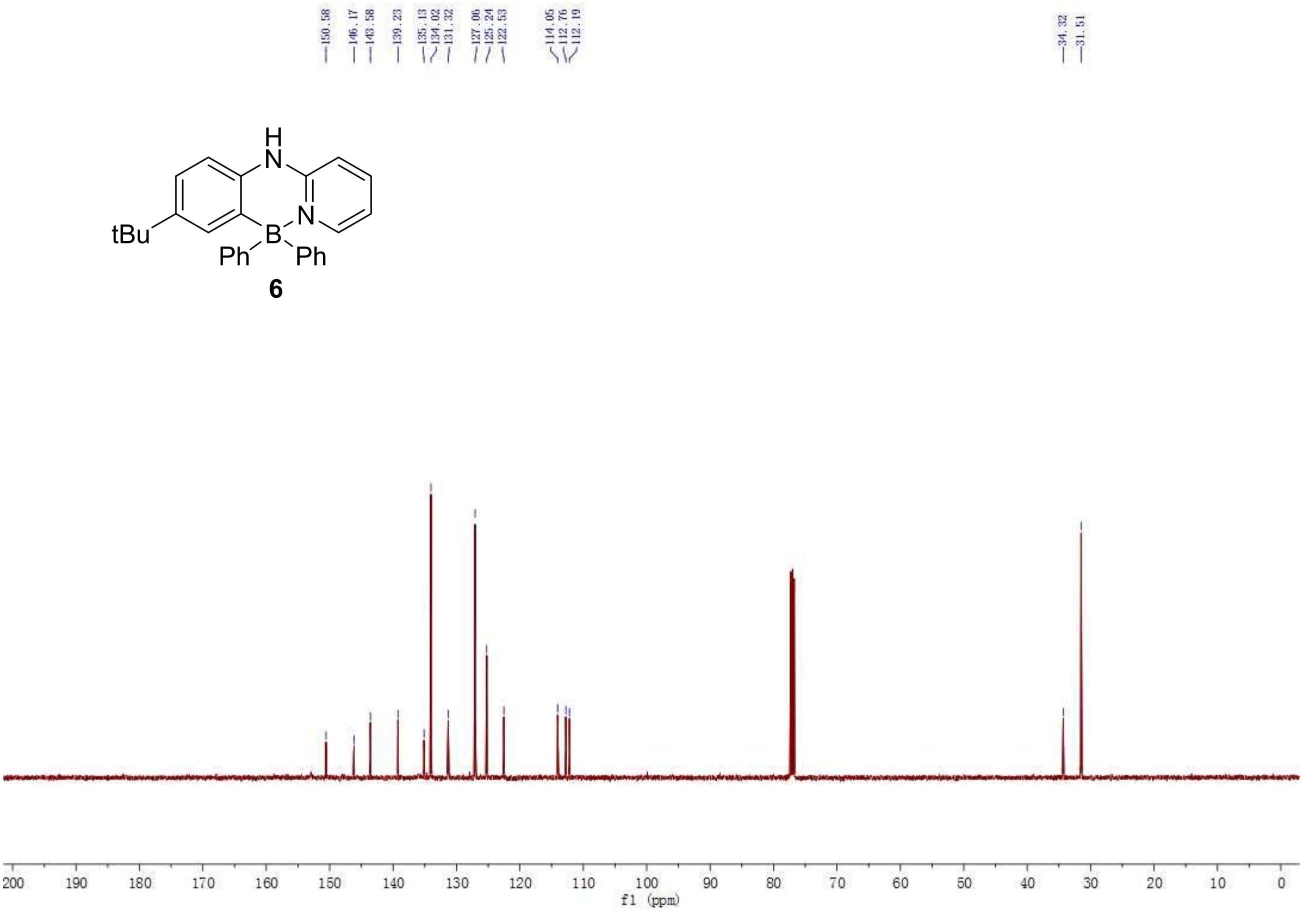
150.58
146.17
143.58
139.23
135.13
134.02
131.32
127.06
125.24
122.53
114.05
112.76
112.19
34.32
31.51
tBu
Ph
Ph
6
200 190 180 170 160 150 140 130 120 110 100 90 80 70 60 50 40 30 20 10 0
f1 (ppm)

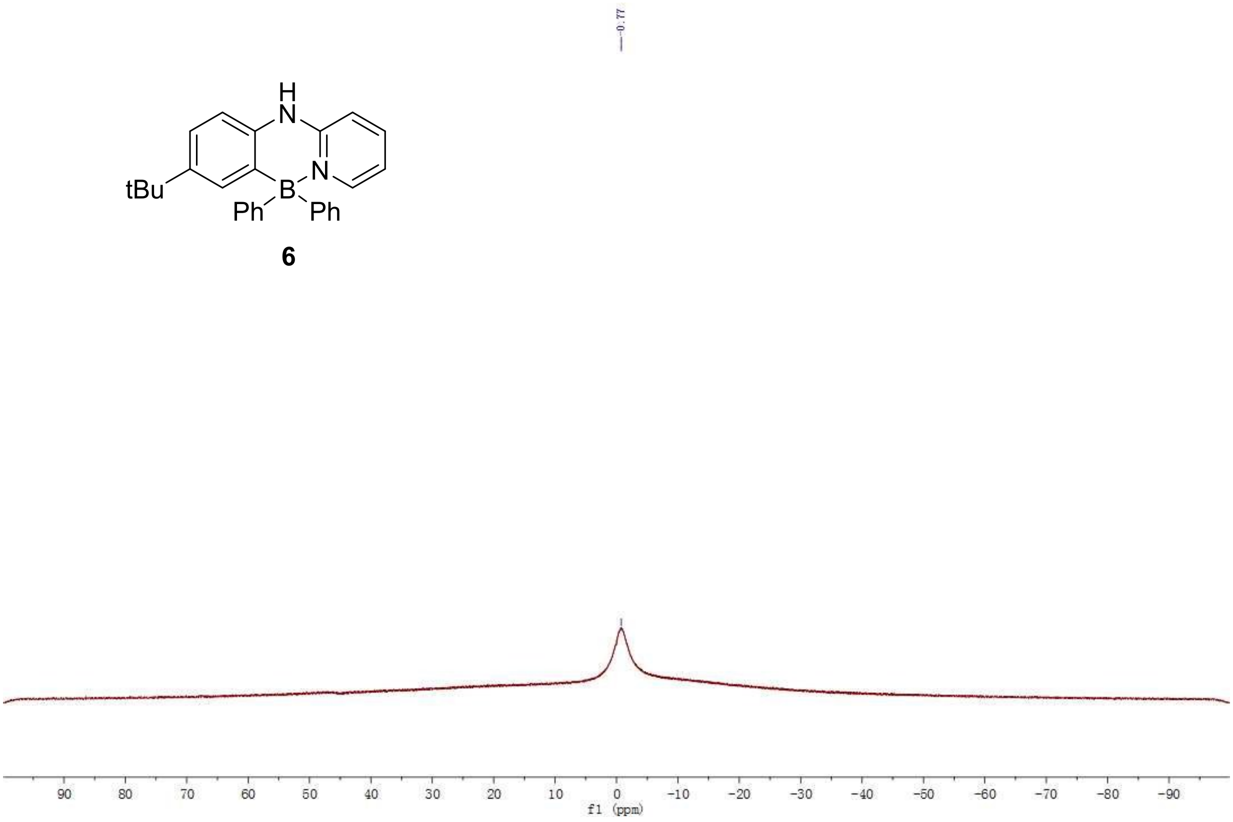

6

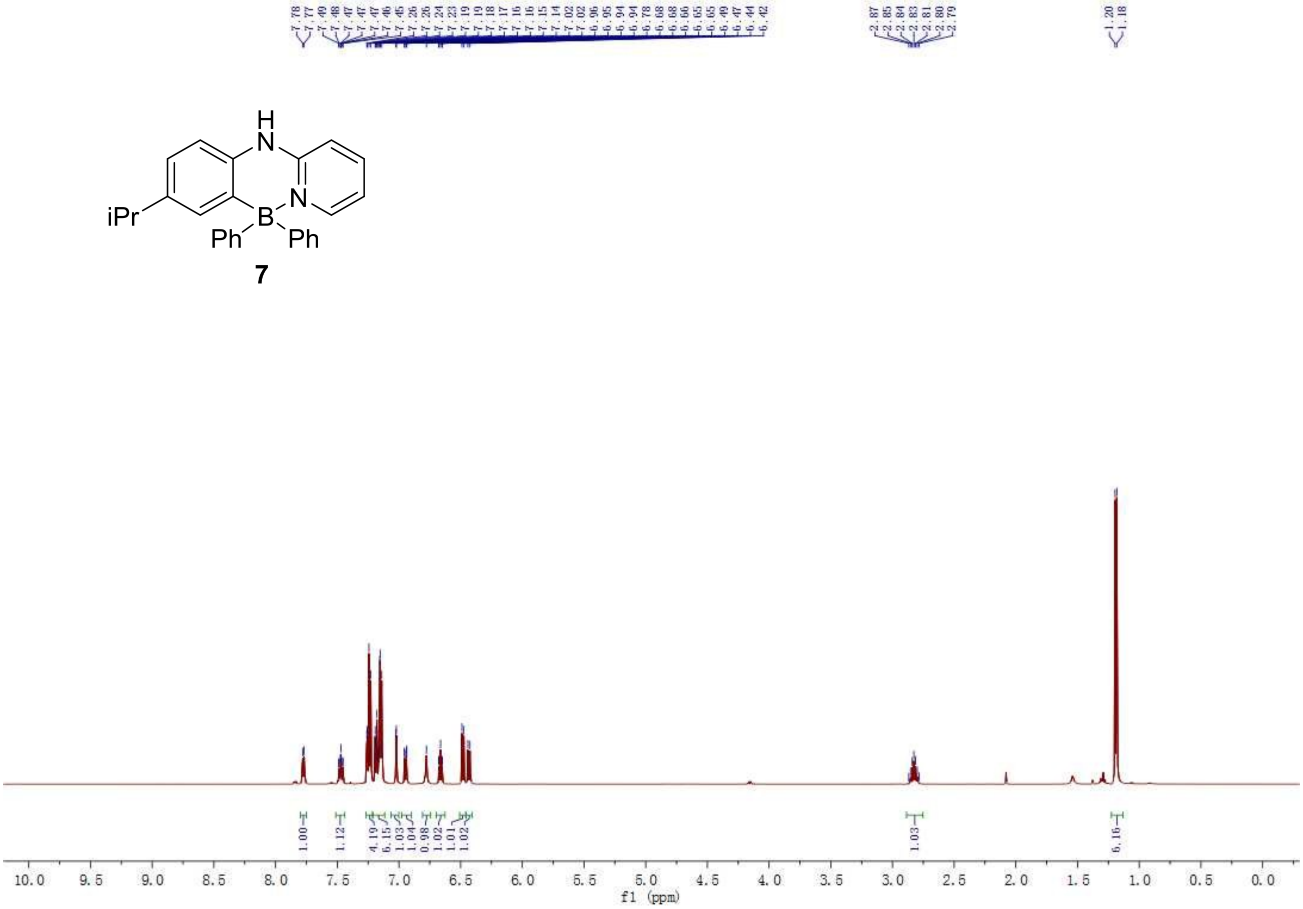

7

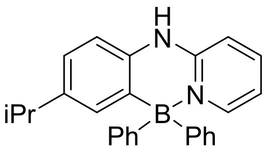
iPr
H
N
N
B
Ph
Ph

7

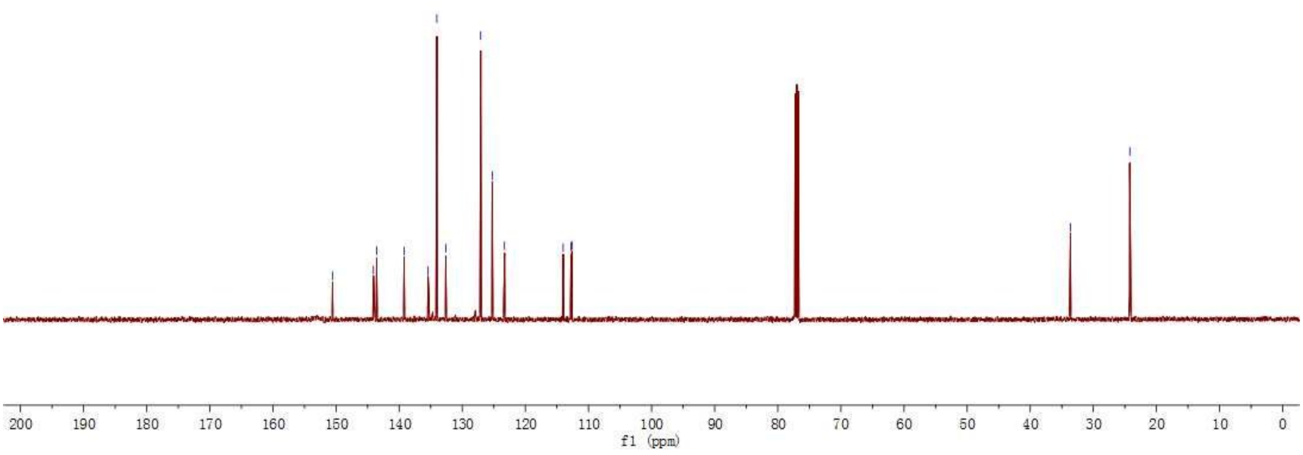
150.56
144.08
143.56
139.22
135.41
134.03
132.61
127.08
125.25
123.30
114.01
112.77
112.62
33.63
24.22
200 190 180 170 160 150 140 130 120 110 100 90 80 70 60 50 40 30 20 10 0
f1 (ppm)

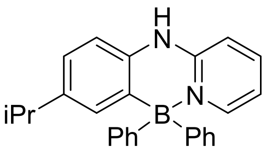
iPr
H
N
N
B
Ph
Ph

7

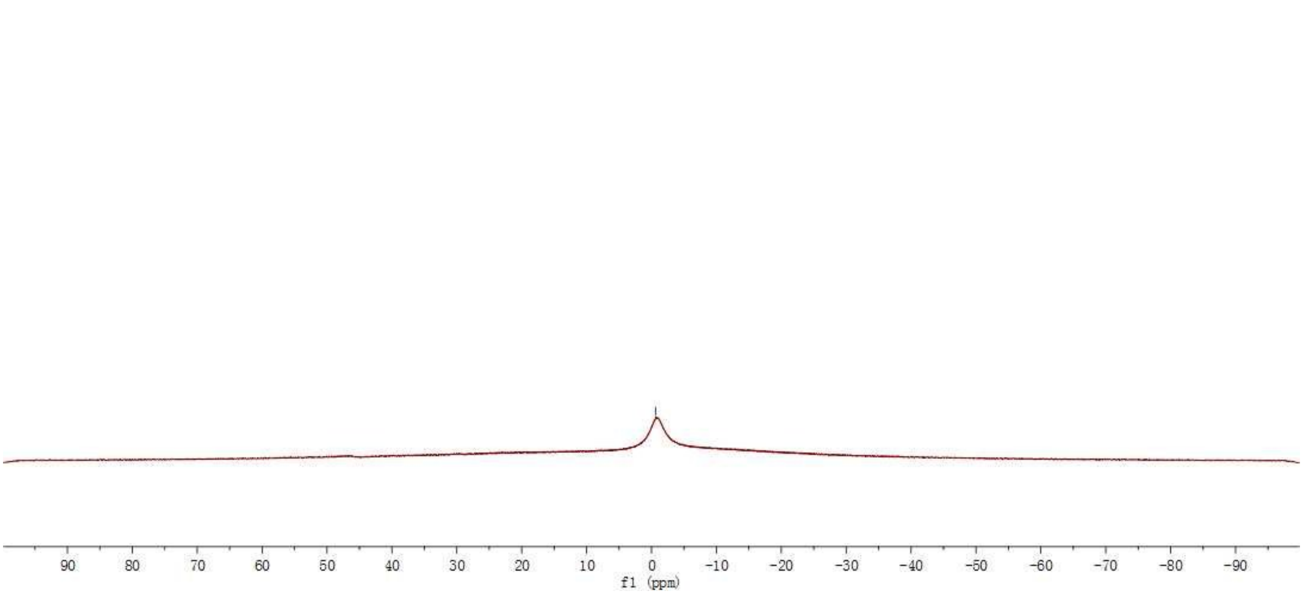
-0.66
90 80 70 60 50 40 30 20 10 0 -10 -20 -30 -40 -50 -60 -70 -80 -90
f1 (ppm)

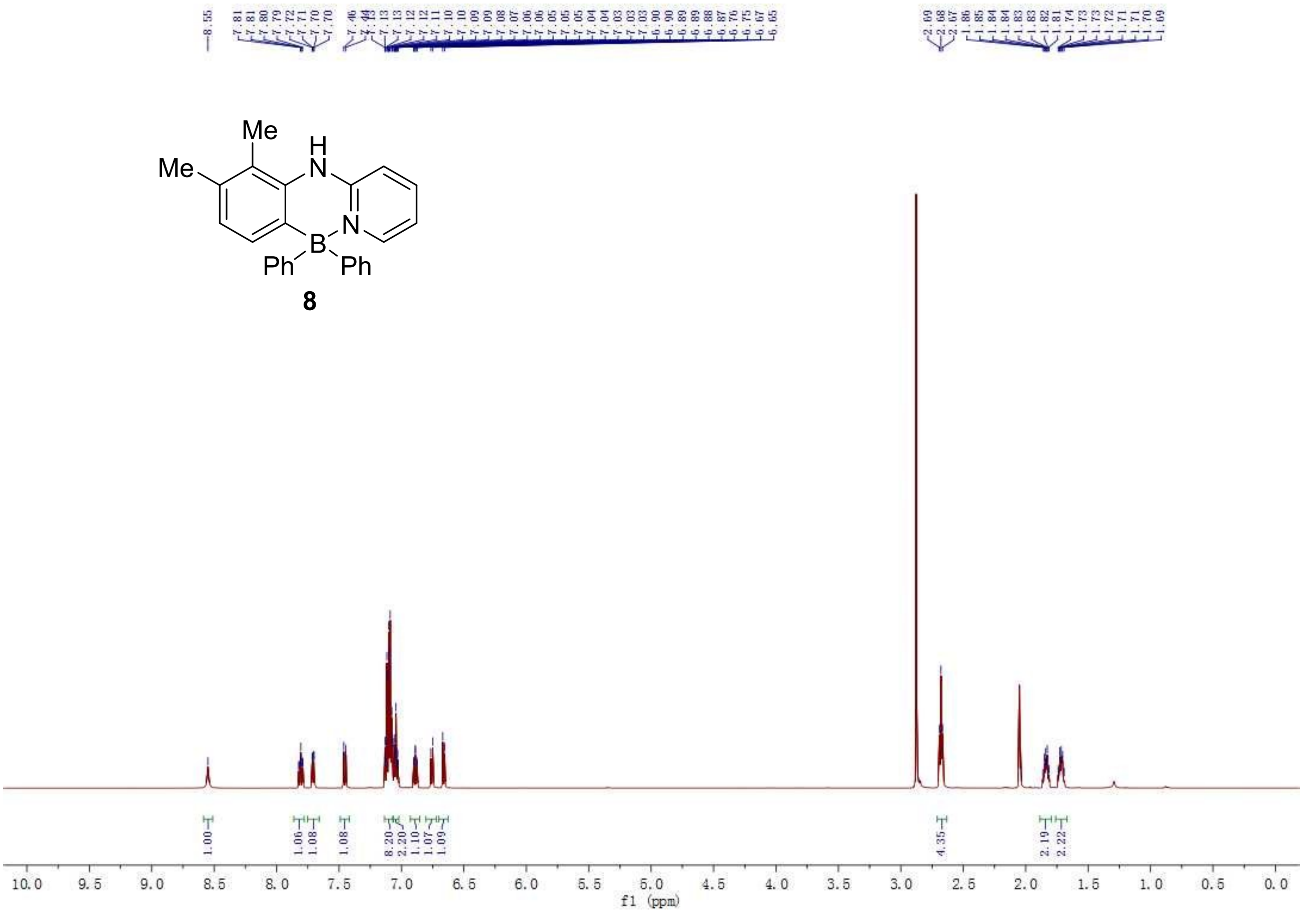

8.55
7.81 7.81 7.80 7.79 7.72 7.71 7.70 7.70
7.46 7.44 7.43 7.13 7.13 7.12 7.12 7.11 7.10 7.10 7.09 7.09 7.08 7.07 7.06 7.06 7.05 7.05 7.04 7.04 7.03 7.03 6.90 6.90 6.89 6.89 6.88 6.87 6.76 6.75 6.67 6.65
2.69 2.68 2.67 1.86 1.85 1.84 1.84 1.83 1.83 1.82 1.81 1.74 1.73 1.73 1.72 1.71 1.70 1.69
Me
Me
H
N
N
B
Ph
Ph
8
1.00
1.06 1.08
1.08
8.20 2.20 1.10 1.07 1.09
4.35
2.19 2.22
10.0 9.5 9.0 8.5 8.0 7.5 7.0 6.5 6.0 5.5 5.0 4.5 4.0 3.5 3.0 2.5 2.0 1.5 1.0 0.5 0.0
f1 (ppm)

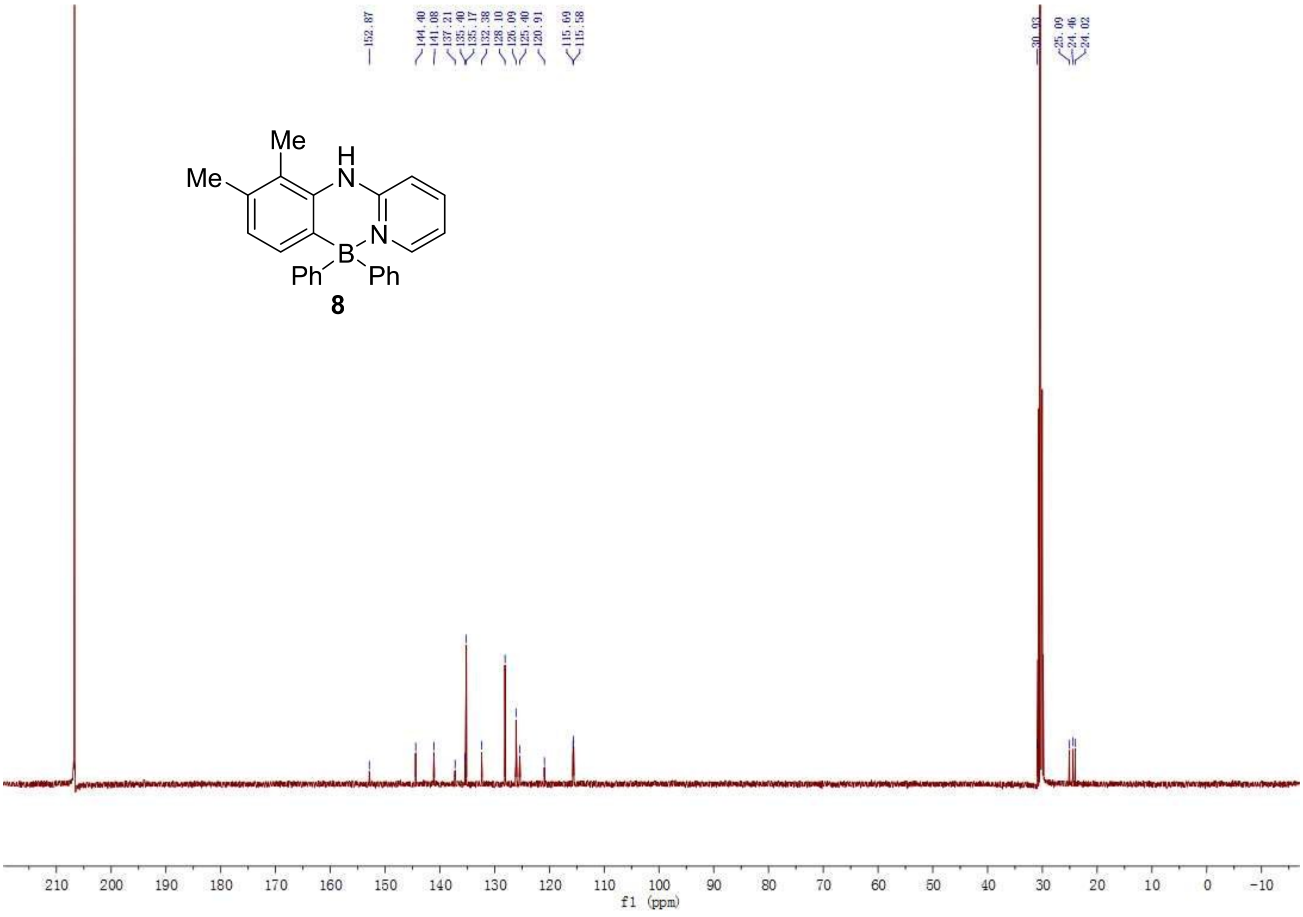

152.87
144.40 141.08 137.21 135.40 135.17 132.38 128.10 126.09 125.40 120.91 115.69 115.58
30.93
25.09 24.46 24.02
Me
Me
H
N
N
B
Ph
Ph
8
210 200 190 180 170 160 150 140 130 120 110 100 90 80 70 60 50 40 30 20 10 0 -10
f1 (ppm)

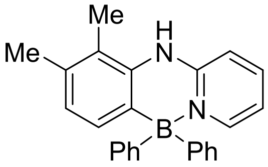
Me
Me
H
N
N
B
Ph
Ph

8

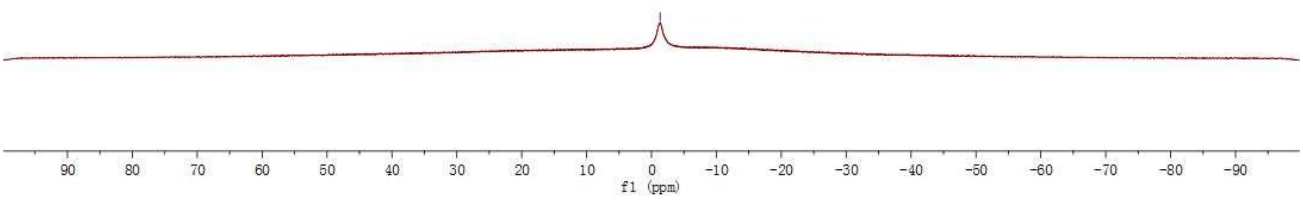
1.32
f1 (ppm)

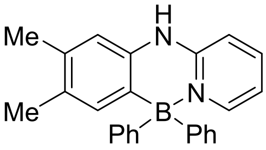
Me
Me
H
N
N
B
Ph
Ph

9

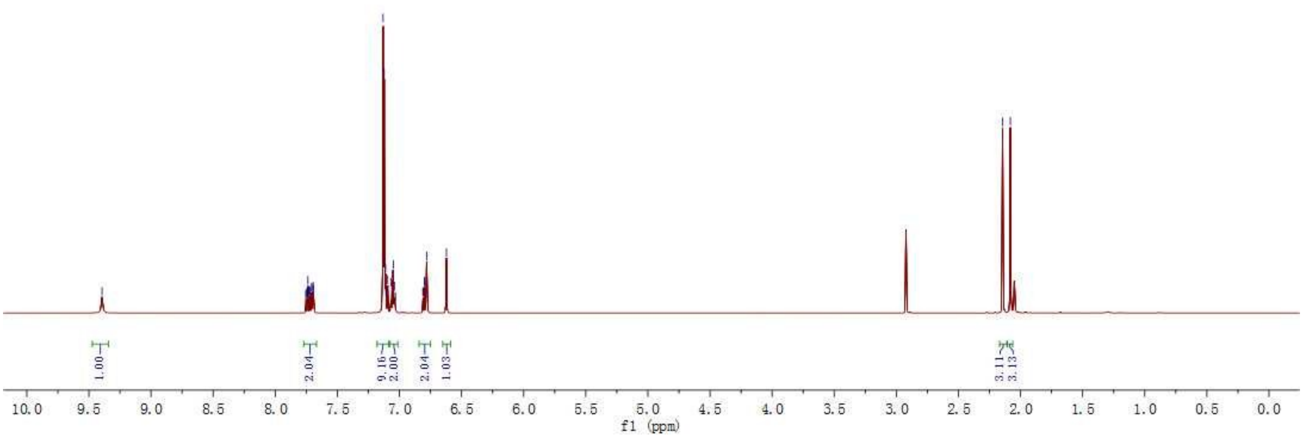
9.39
7.75 7.75 7.74 7.74 7.73 7.72 7.72 7.71 7.70 7.69 7.69
7.13 7.12 7.11 7.05 6.81 6.80 6.80 6.78 6.62
2.14 2.08
1.00
2.04
9.16 2.00
2.04
1.03
3.11 3.13
f1 (ppm)

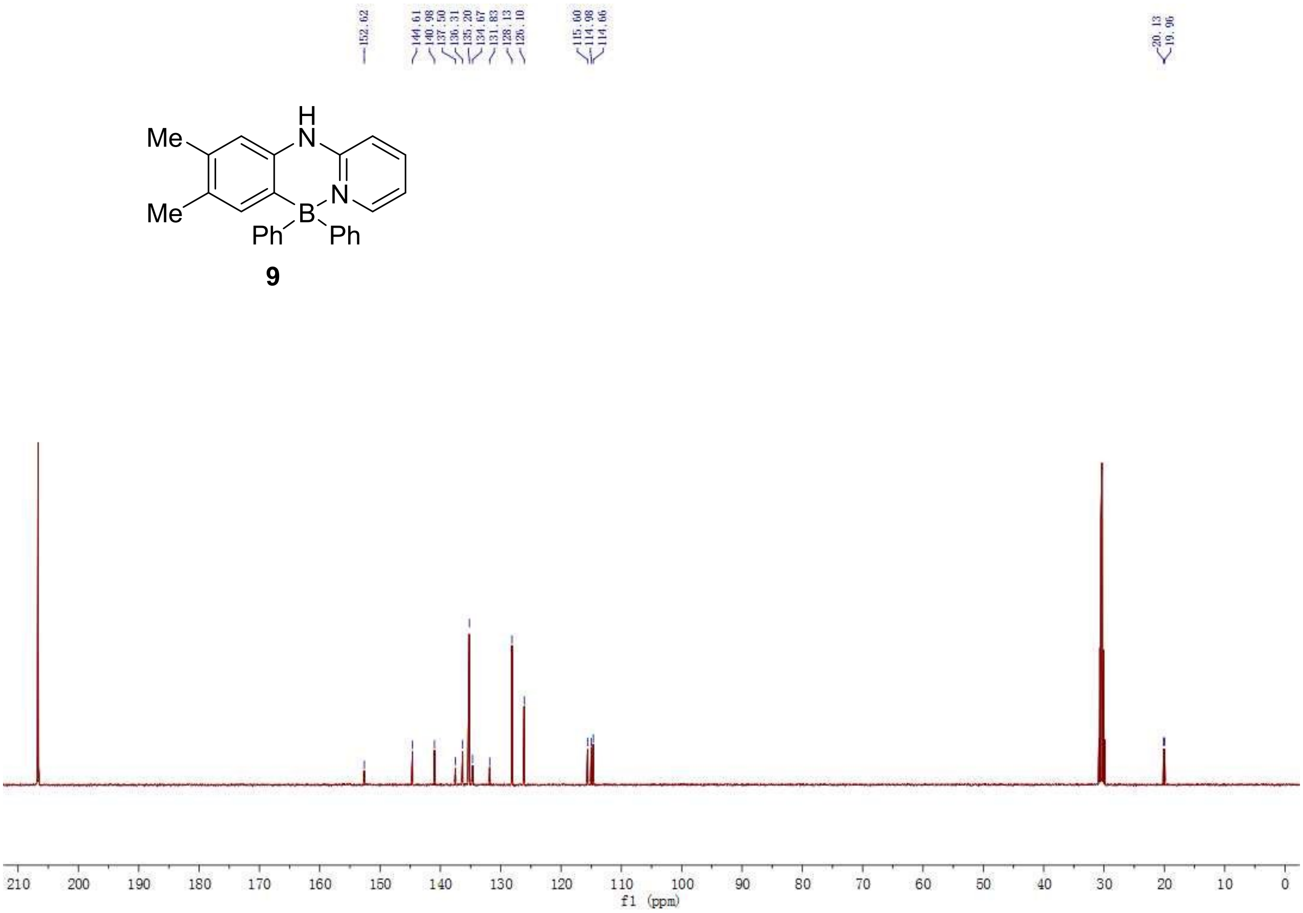
152.62
144.61
140.98
137.50
136.31
135.20
134.67
131.83
128.13
126.10
115.60
114.98
114.66
20.13
19.96
Me
Me
H
N
N
B
Ph
Ph
9
210 200 190 180 170 160 150 140 130 120 110 100 90 80 70 60 50 40 30 20 10 0
f1 (ppm)

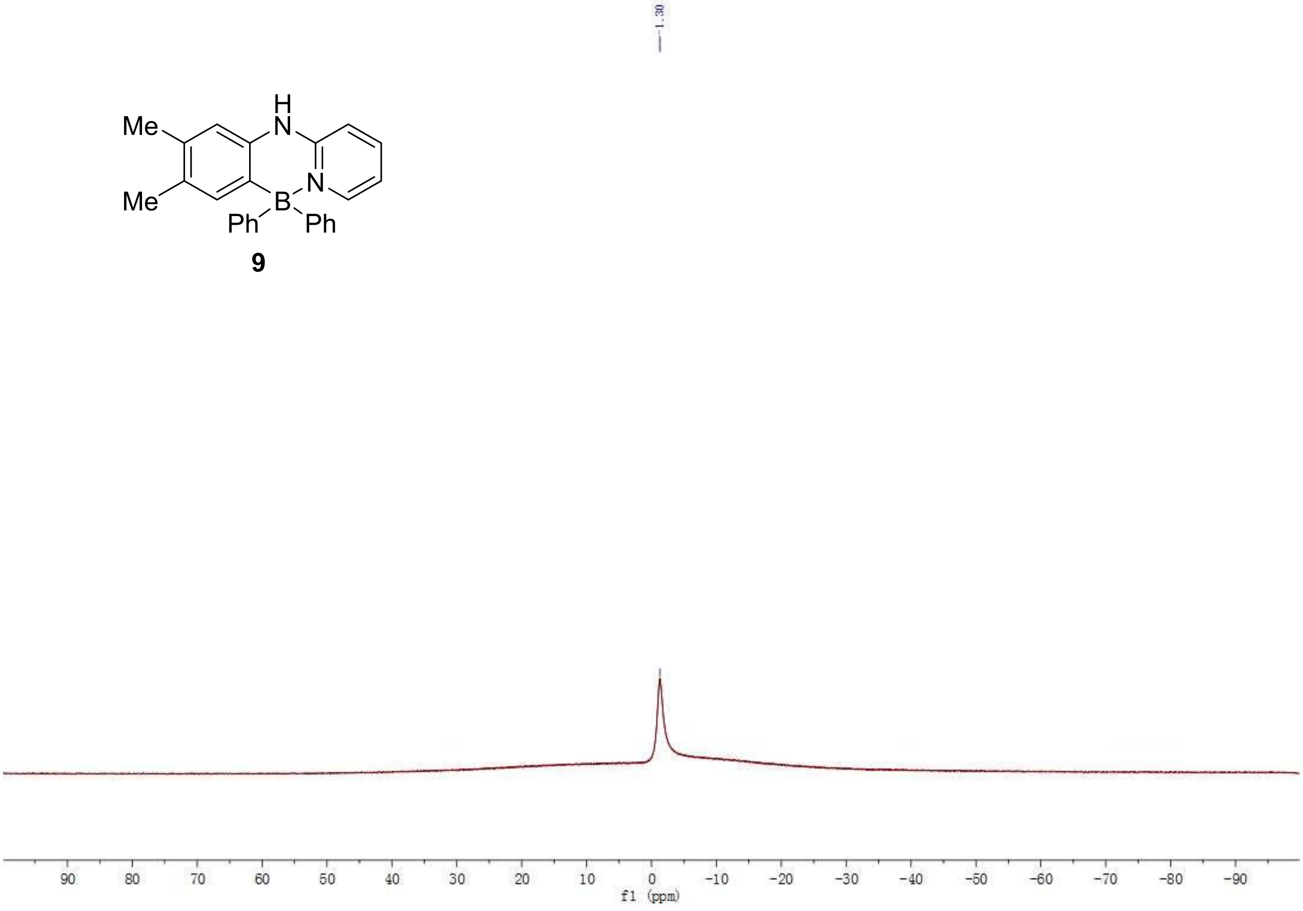
1.30
Me
Me
H
N
N
B
Ph
Ph
9
90 80 70 60 50 40 30 20 10 0 -10 -20 -30 -40 -50 -60 -70 -80 -90
f1 (ppm)

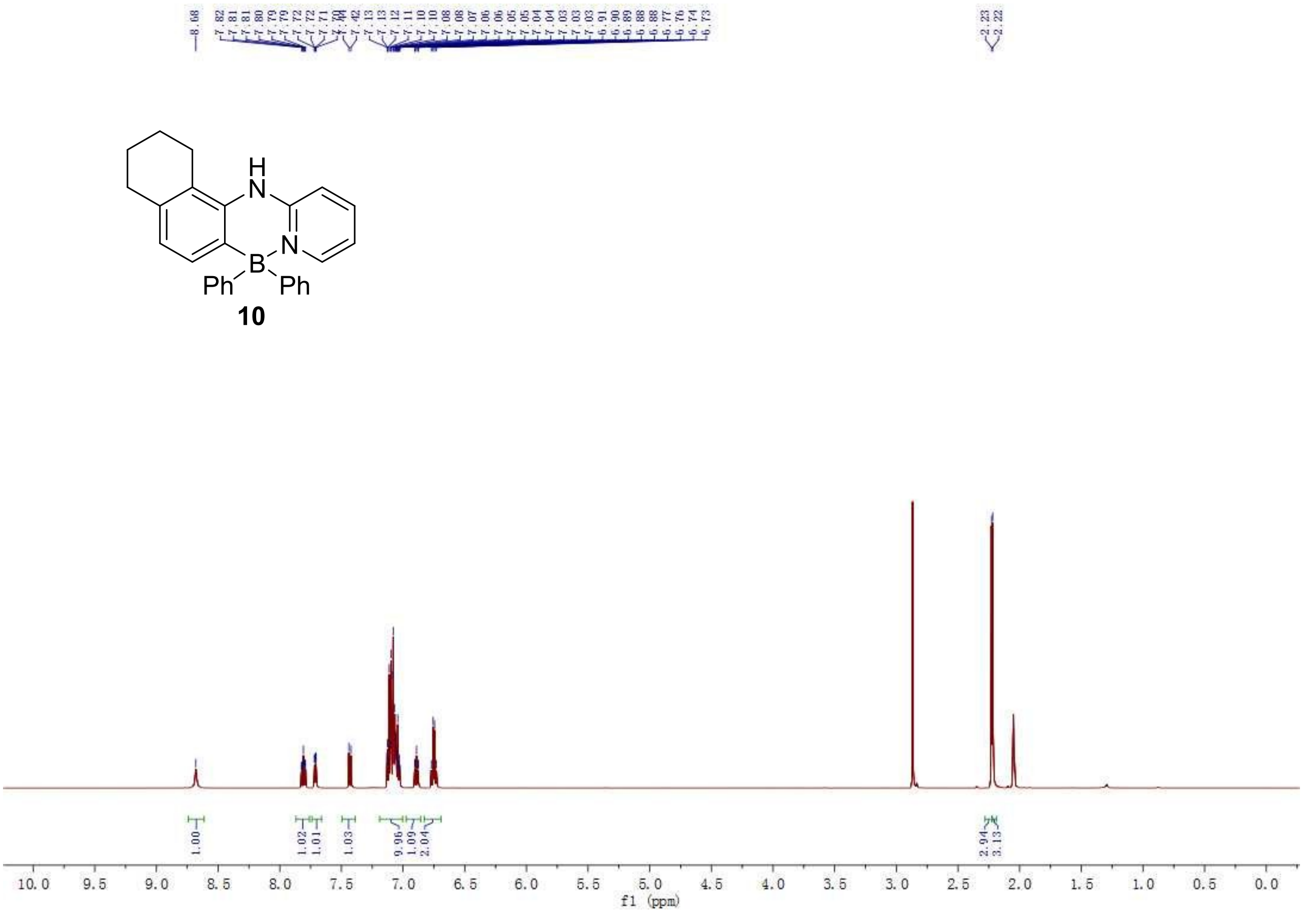
8.68
7.82 7.81 7.81 7.80 7.79 7.79 7.72 7.72 7.71 7.70 7.44 7.42 7.13 7.12 7.11 7.10 7.08 7.08 7.07 7.06 7.06 7.05 7.05 7.04 7.04 7.03 7.03 7.03 6.91 6.90 6.89 6.88 6.77 6.76 6.74 6.73
2.23 2.22
H
N
N
B
Ph
Ph
10
1.00
1.02 1.01
1.03
9.96 1.09 2.04
2.94 3.13
f1 (ppm)

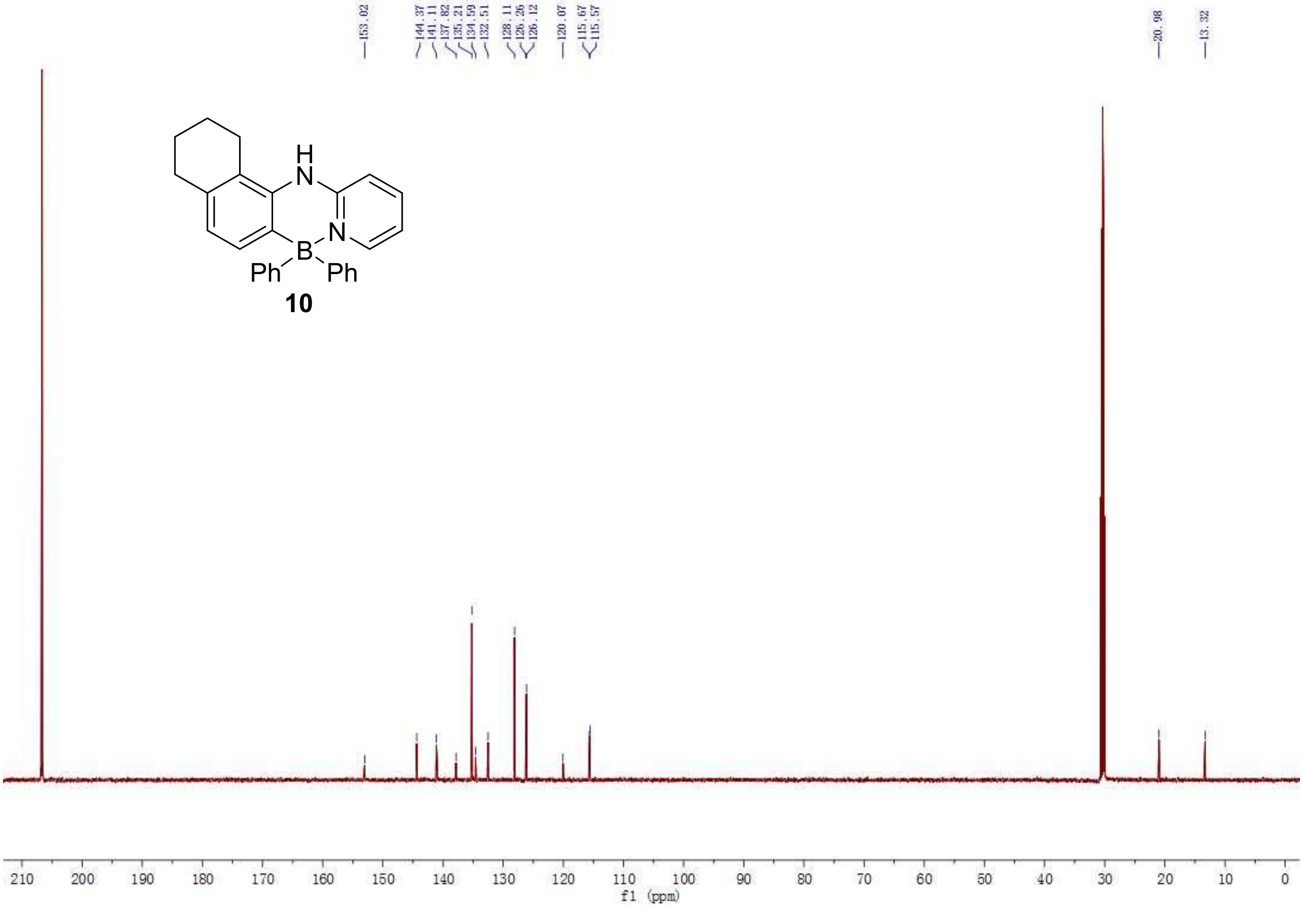
153.02
144.37 141.11 137.82 135.21 134.59 132.51
128.11 126.26 126.12
120.07
115.67 115.57
20.98
13.32
H
N
N
B
Ph
Ph
10
f1 (ppm)

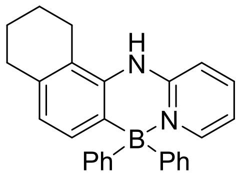

10

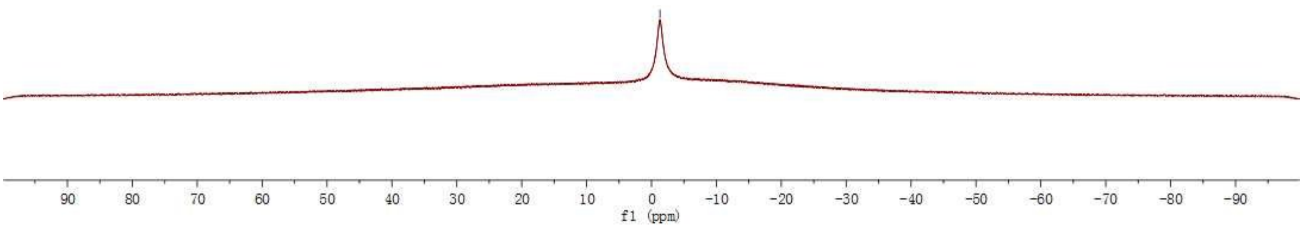

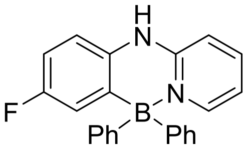

11

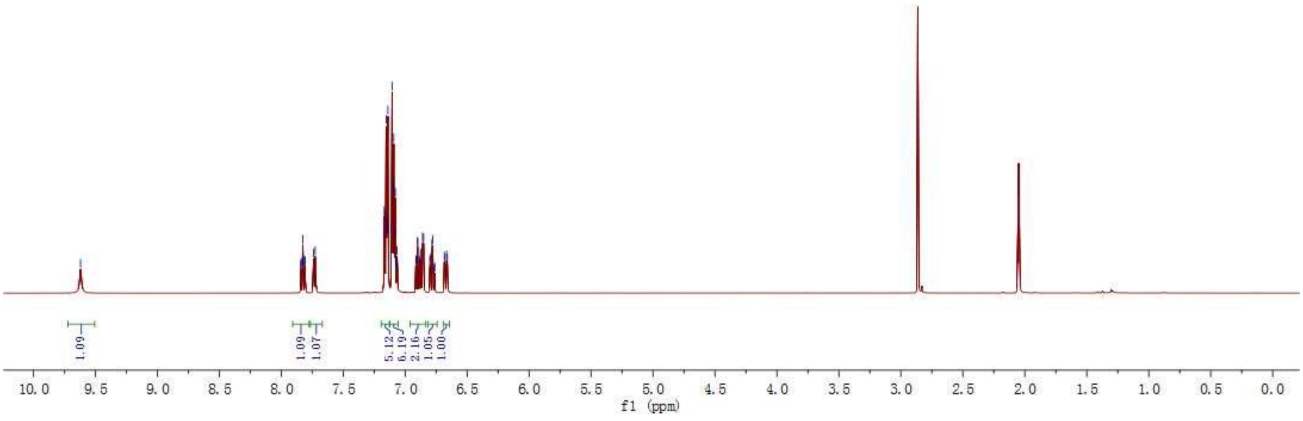

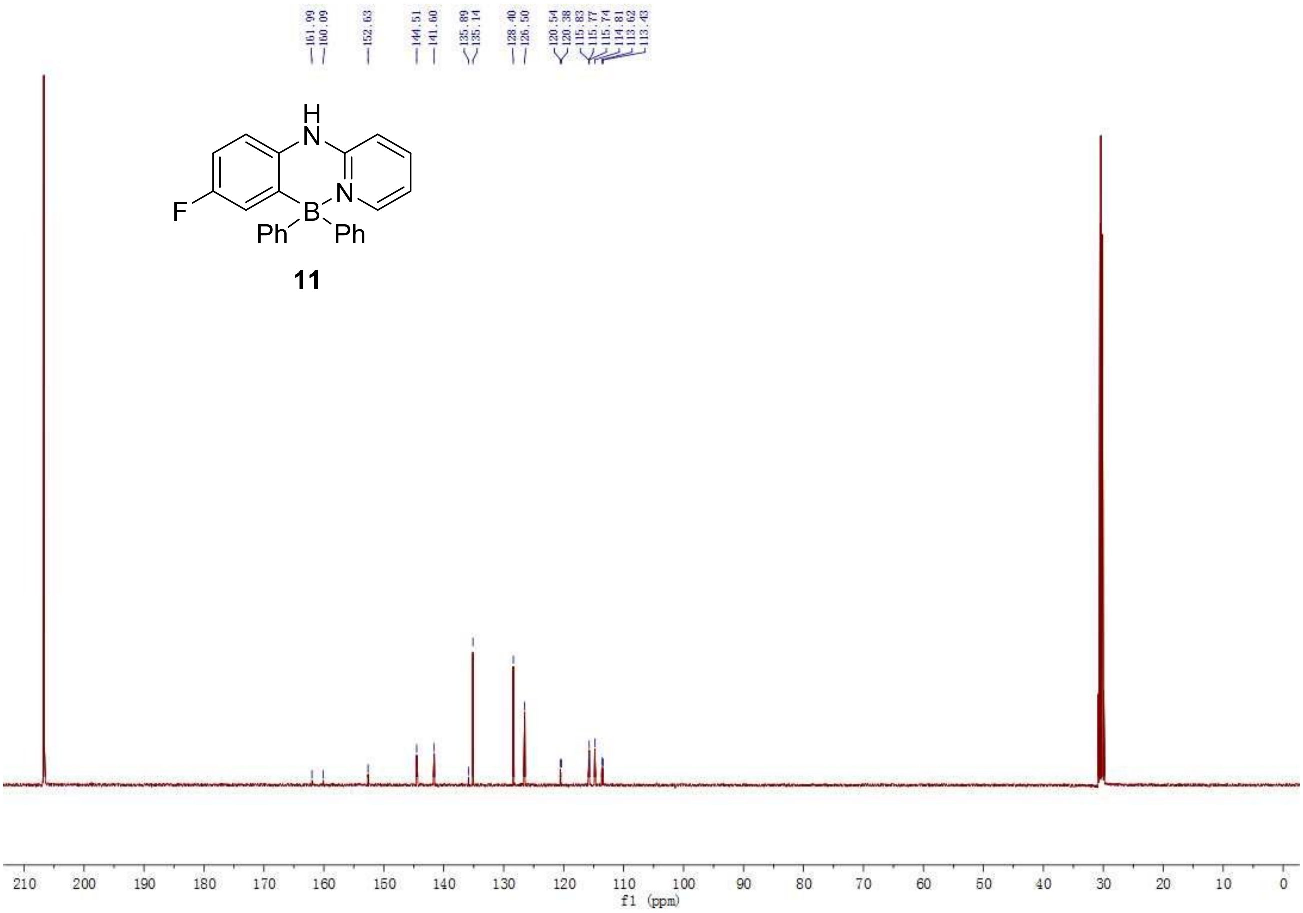

161.99
160.09
152.63
144.51
141.60
135.89
135.14
128.40
126.50
120.54
120.38
115.83
115.77
115.74
114.81
113.62
113.43
H
N
N
F
B
Ph
Ph
11
210 200 190 180 170 160 150 140 130 120 110 100 90 80 70 60 50 40 30 20 10 0
f1 (ppm)

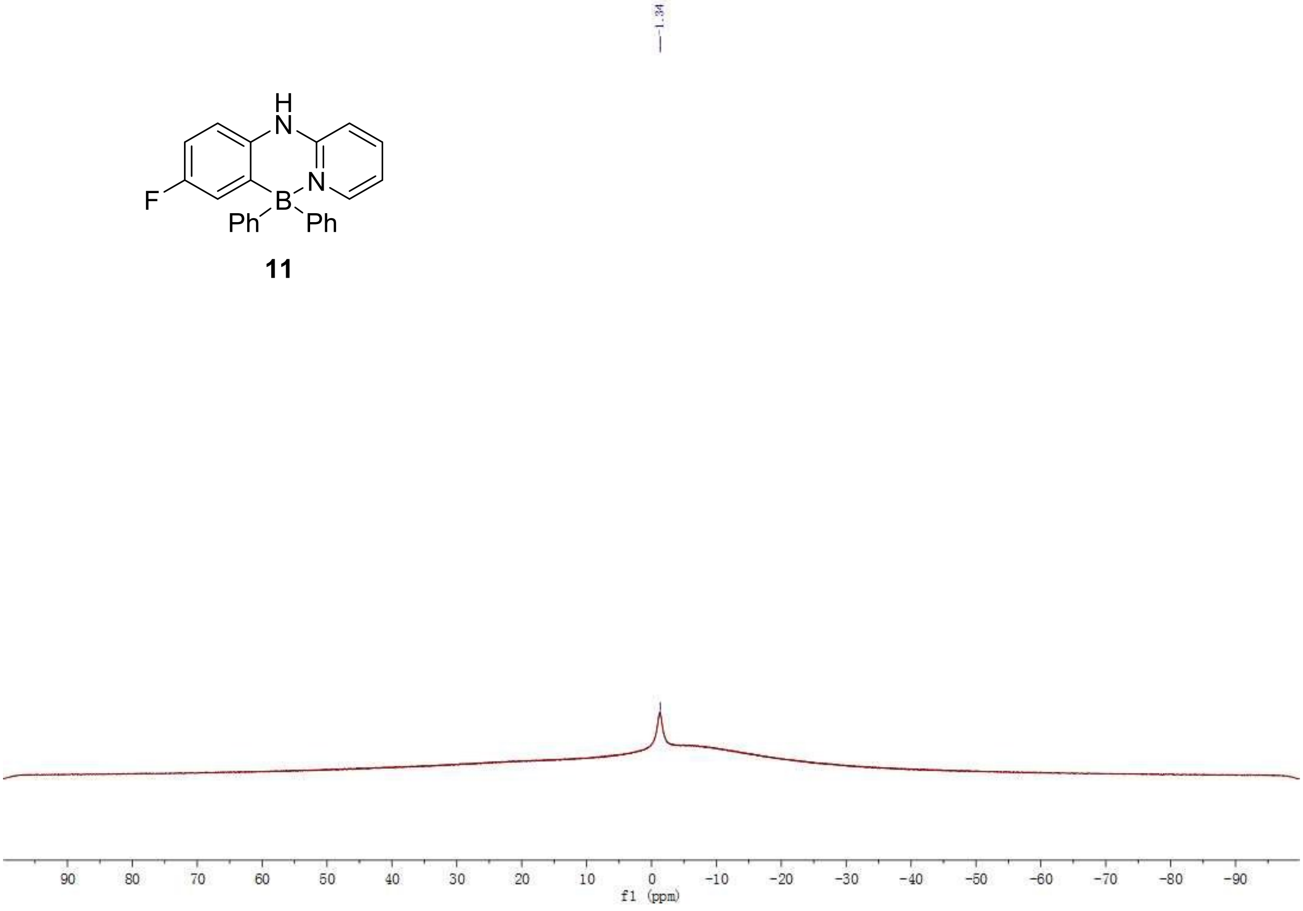

1.34
H
N
N
F
B
Ph
Ph
11
90 80 70 60 50 40 30 20 10 0 -10 -20 -30 -40 -50 -60 -70 -80 -90
f1 (ppm)

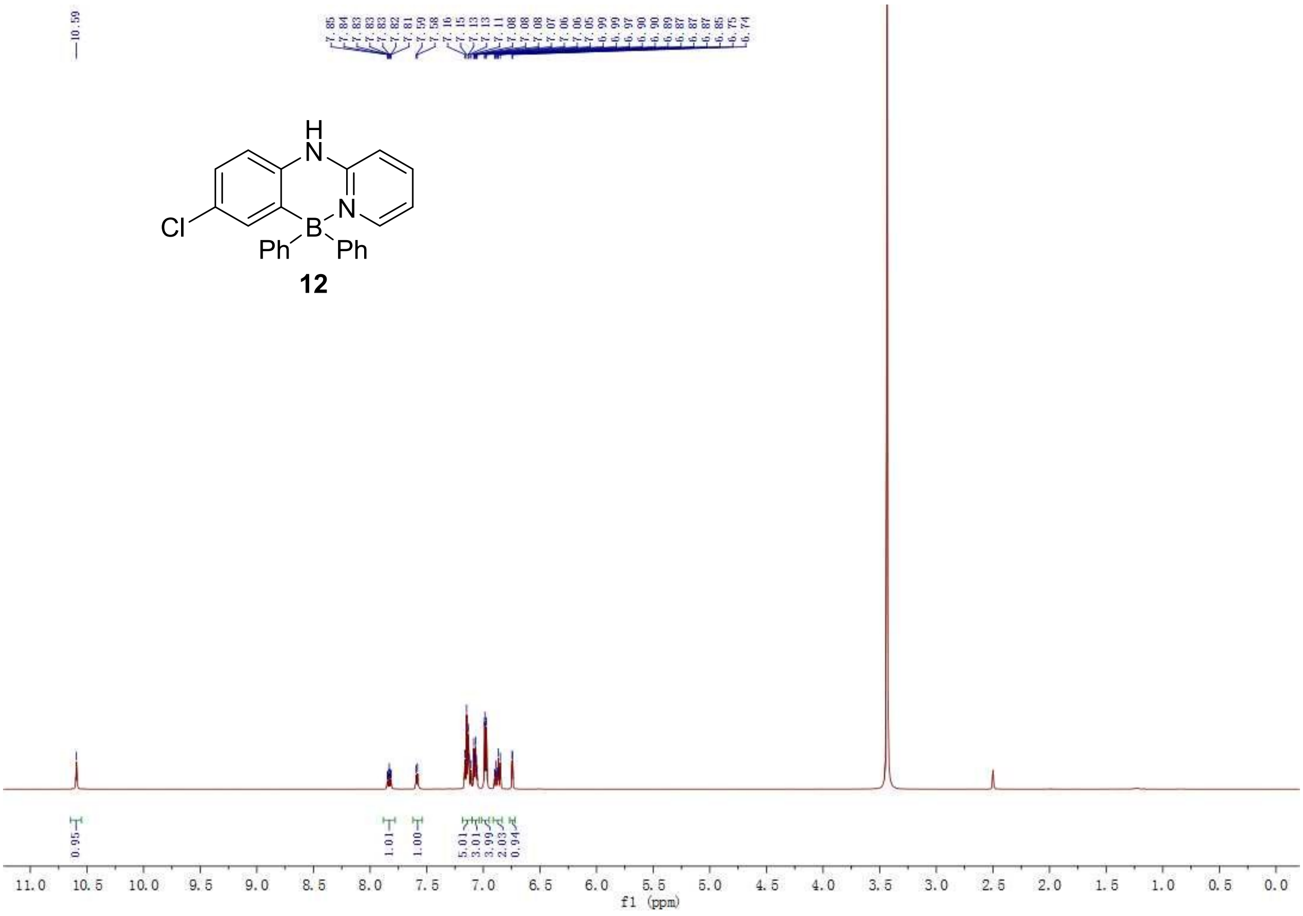
10.59
7.85 7.84 7.83 7.83 7.82 7.81 7.59 7.58 7.16 7.15 7.13 7.13 7.11 7.08 7.08 7.07 7.06 7.05 6.99 6.99 6.97 6.90 6.89 6.87 6.87 6.85 6.75 6.74
H
N
Cl
B
Ph
Ph
12
0.95
1.01
1.00
5.01
3.01
3.99
2.03
0.94
11.0 10.5 10.0 9.5 9.0 8.5 8.0 7.5 7.0 6.5 6.0 5.5 5.0 4.5 4.0 3.5 3.0 2.5 2.0 1.5 1.0 0.5 0.0
f1 (ppm)

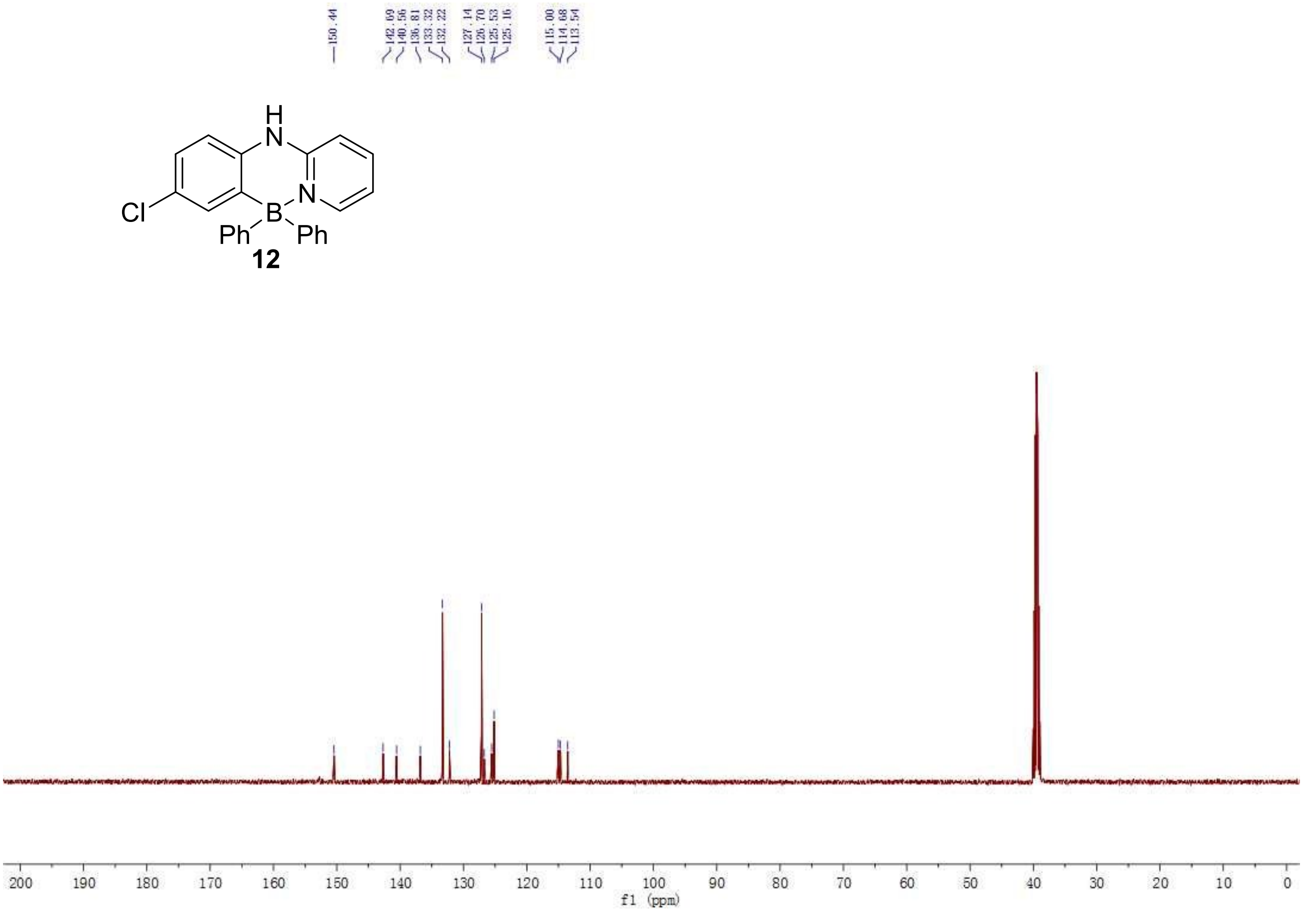
150.44
142.69 140.56 136.81 133.32 132.22
127.14 126.70 125.53 125.16
115.00 114.68 113.54
H
N
Cl
B
Ph
Ph
12
200 190 180 170 160 150 140 130 120 110 100 90 80 70 60 50 40 30 20 10 0
f1 (ppm)

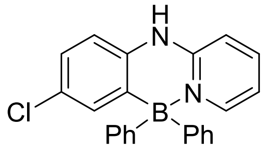

12

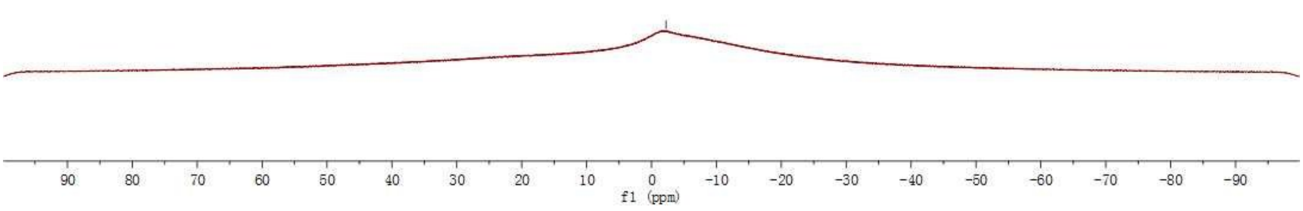

-2.23
f1 (ppm)

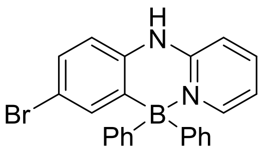

13

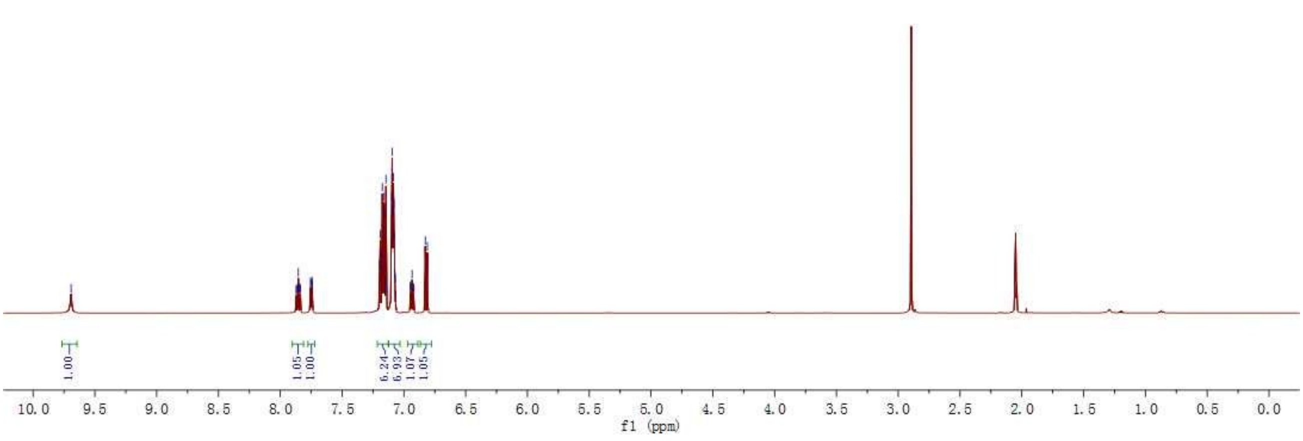

9.69
7.87 7.87 7.86 7.85 7.85 7.84 7.84 7.76 7.75 7.74 7.74 7.20 7.19 7.18 7.17 7.16 7.16 7.16 7.15 7.15 7.14 7.10 7.10 7.09 7.09 7.08 7.08 7.07 7.07 7.07 6.95 6.93 6.92 6.92 6.83 6.81
1.00
1.05
1.00
6.24
6.93
1.07
1.05
f1 (ppm)

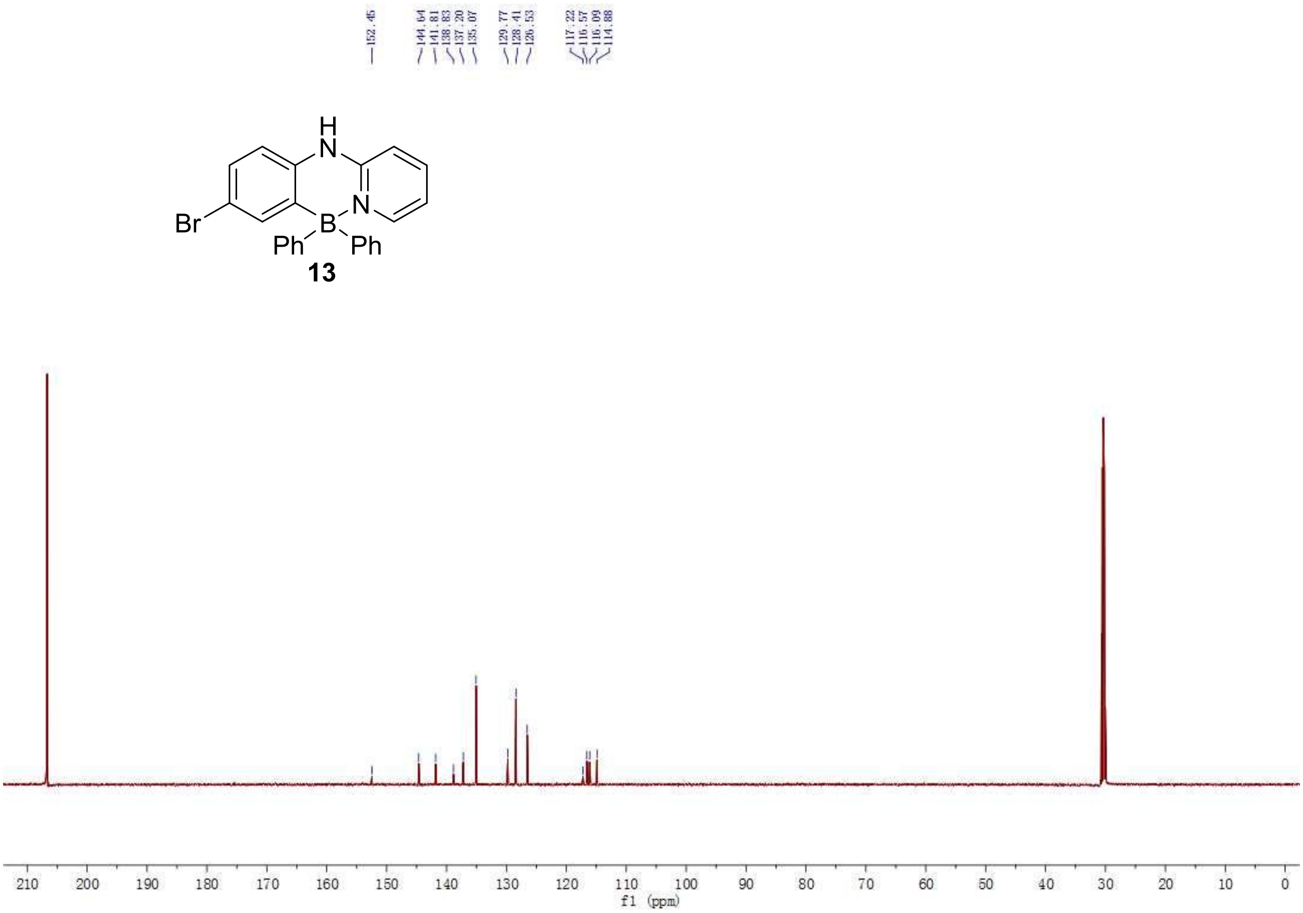

152.45
144.64
141.81
138.83
137.20
135.07
129.77
128.41
126.53
117.22
116.57
116.09
114.88
H
N
N
Br
B
Ph
Ph
13
210 200 190 180 170 160 150 140 130 120 110 100 90 80 70 60 50 40 30 20 10 0
f1 (ppm)

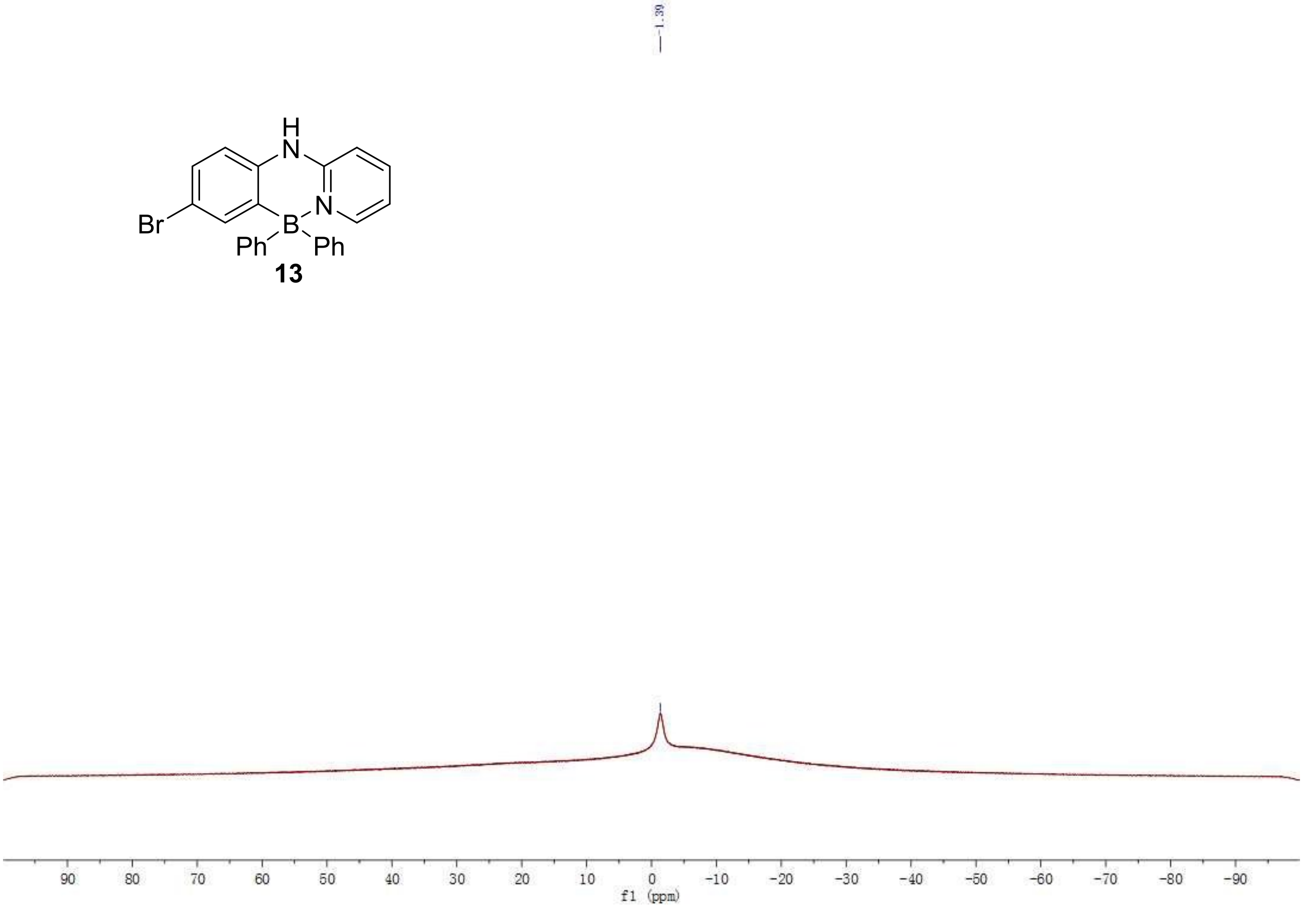

1.39
H
N
N
Br
B
Ph
Ph
13
90 80 70 60 50 40 30 20 10 0 -10 -20 -30 -40 -50 -60 -70 -80 -90
f1 (ppm)

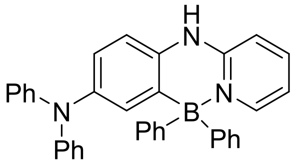
H
N
Ph
B

14

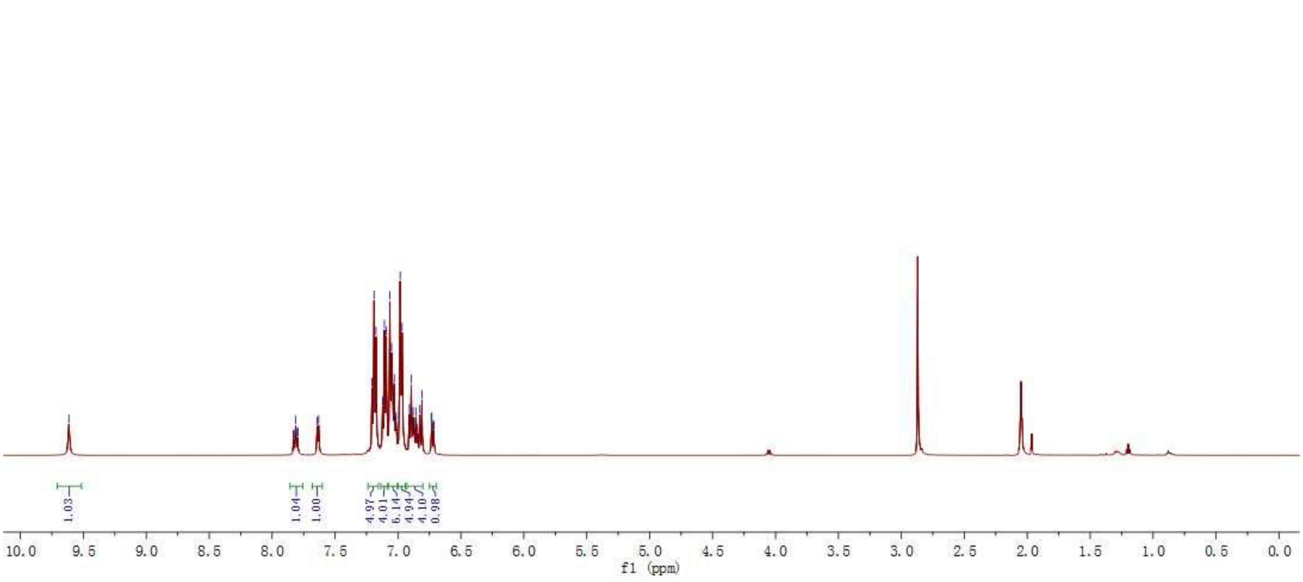
9.62
7.83 7.83 7.81 7.80 7.80 7.64 7.63 7.21 7.19 7.17 7.12 7.11 7.09 7.06 7.05 7.03 7.02 6.98 6.97 6.91 6.89 6.88 6.87 6.86 6.85 6.83 6.81 6.74 6.73 6.72 6.71
1.03
1.04
1.00
4.97
4.01
6.14
4.94
4.10
0.98
f1 (ppm)

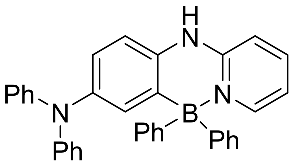
H
N
Ph
B

14

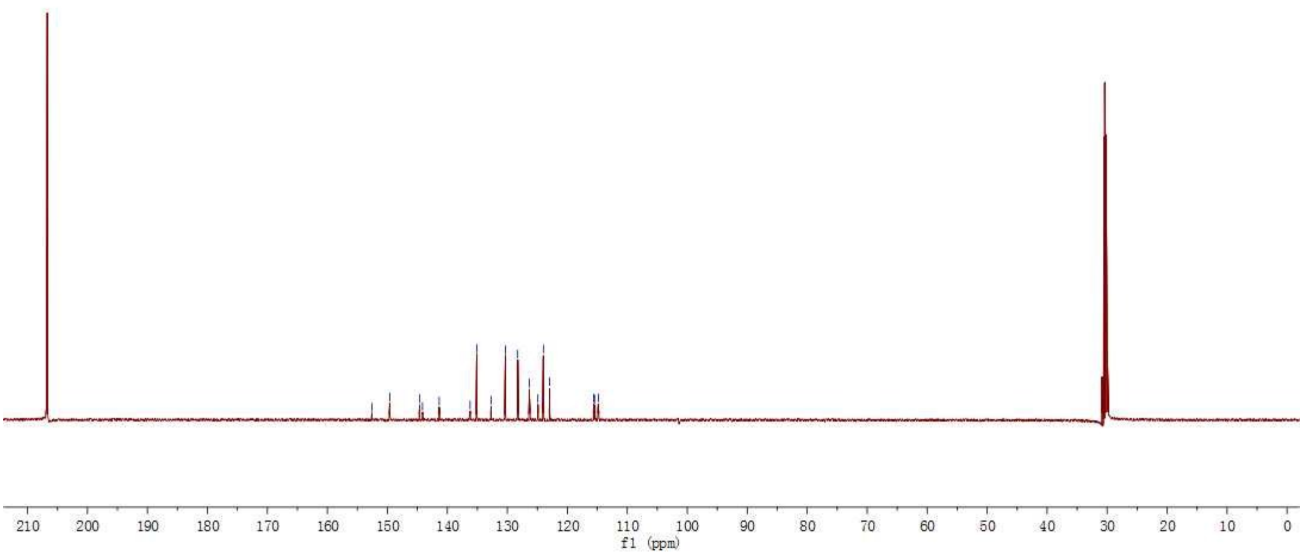
152.57
149.58
144.62 144.15 141.36
136.21 135.09 132.71 130.32 128.27 126.32 124.91 123.99 122.95
115.90 115.45 114.84
f1 (ppm)

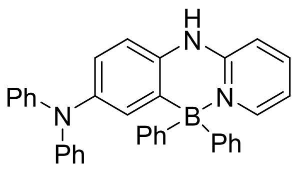

H
N
Ph
N
Ph
B
N
Ph
Ph

14

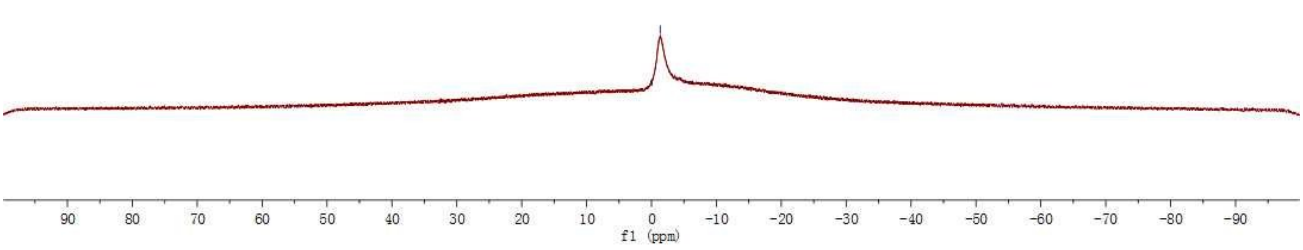

90 80 70 60 50 40 30 20 10 0 -10 -20 -30 -40 -50 -60 -70 -80 -90
f1 (ppm)

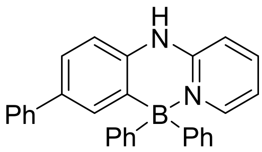

H
N
Ph
B
N
Ph
Ph

15

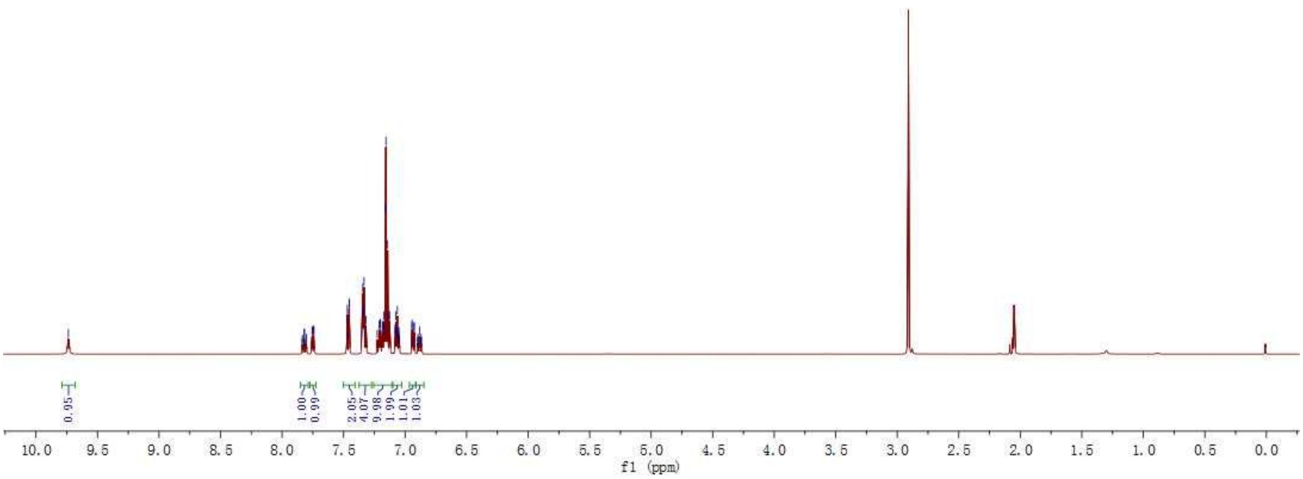

0.95
1.00
0.99
2.05
4.07
9.98
1.99
1.01
1.03
10.0 9.5 9.0 8.5 8.0 7.5 7.0 6.5 6.0 5.5 5.0 4.5 4.0 3.5 3.0 2.5 2.0 1.5 1.0 0.5 0.0
f1 (ppm)

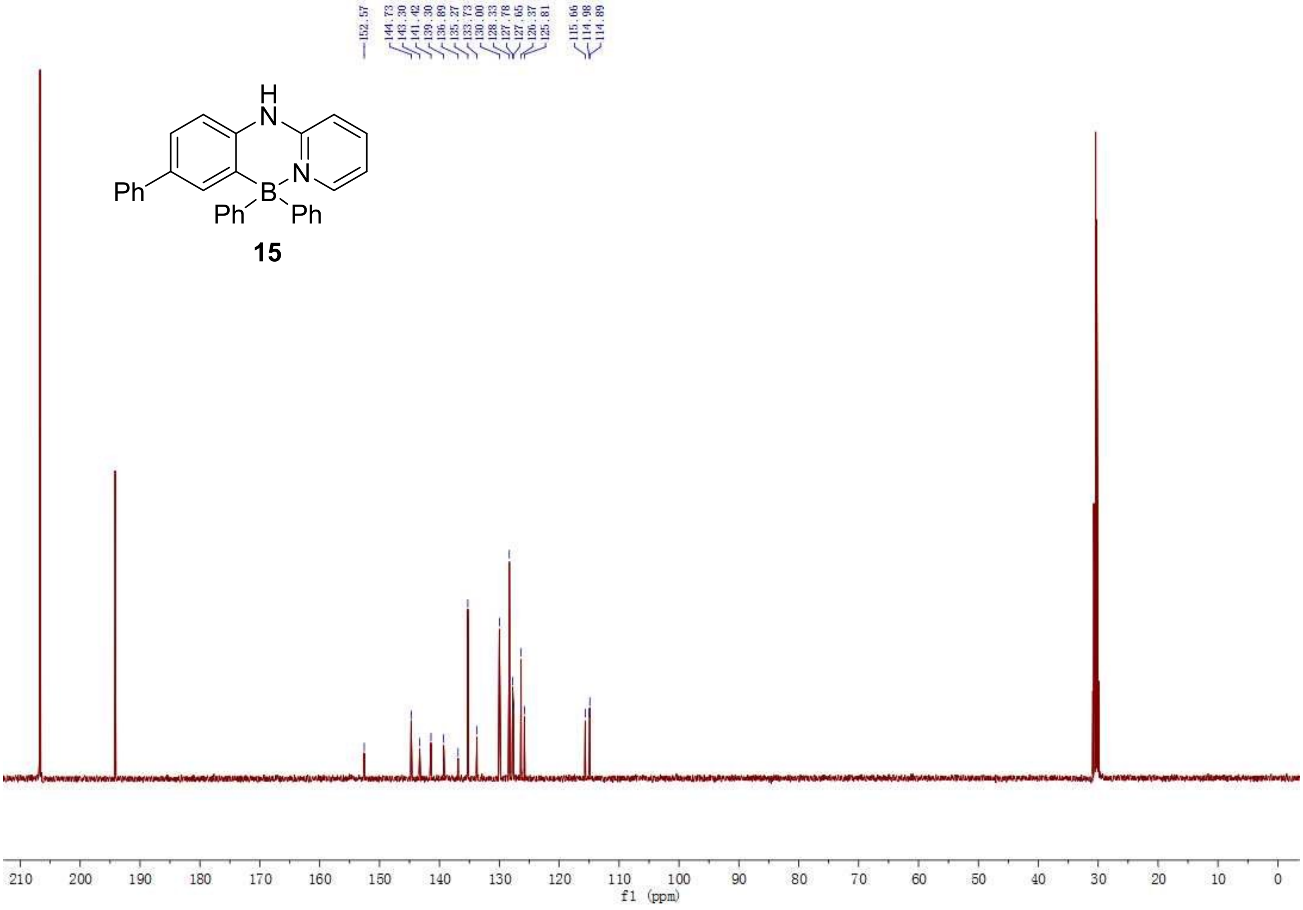

152.57
144.73
143.30
141.42
139.30
136.89
135.27
133.73
130.00
128.33
127.78
127.65
126.37
125.81
115.66
114.98
114.89
H
N
N
B
Ph
Ph
Ph
210 200 190 180 170 160 150 140 130 120 110 100 90 80 70 60 50 40 30 20 10 0
f1 (ppm)

15

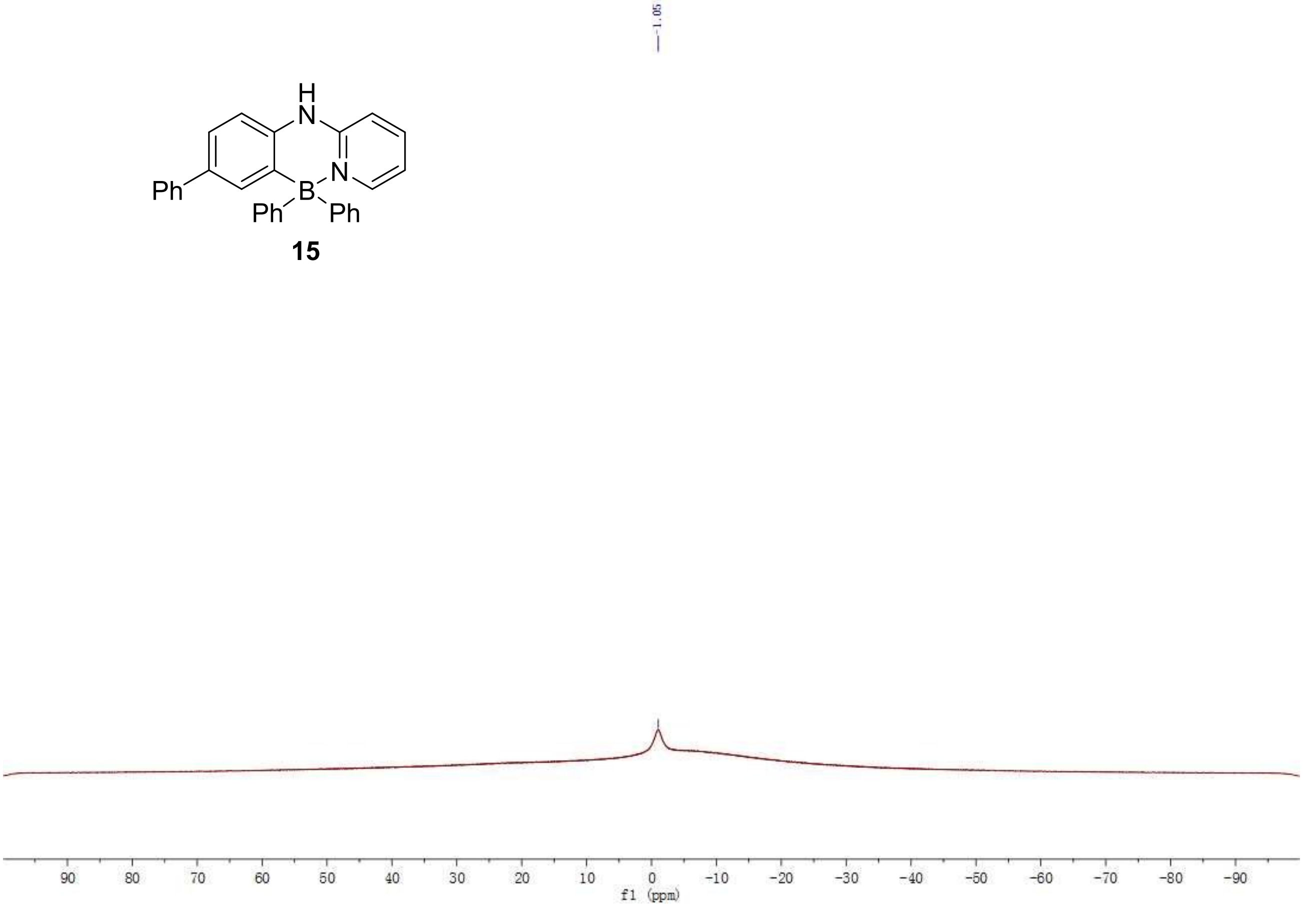

1.05
H
N
N
B
Ph
Ph
Ph
90 80 70 60 50 40 30 20 10 0 -10 -20 -30 -40 -50 -60 -70 -80 -90
f1 (ppm)

15

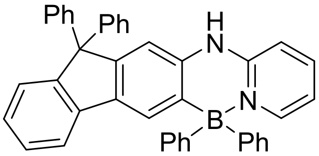

16

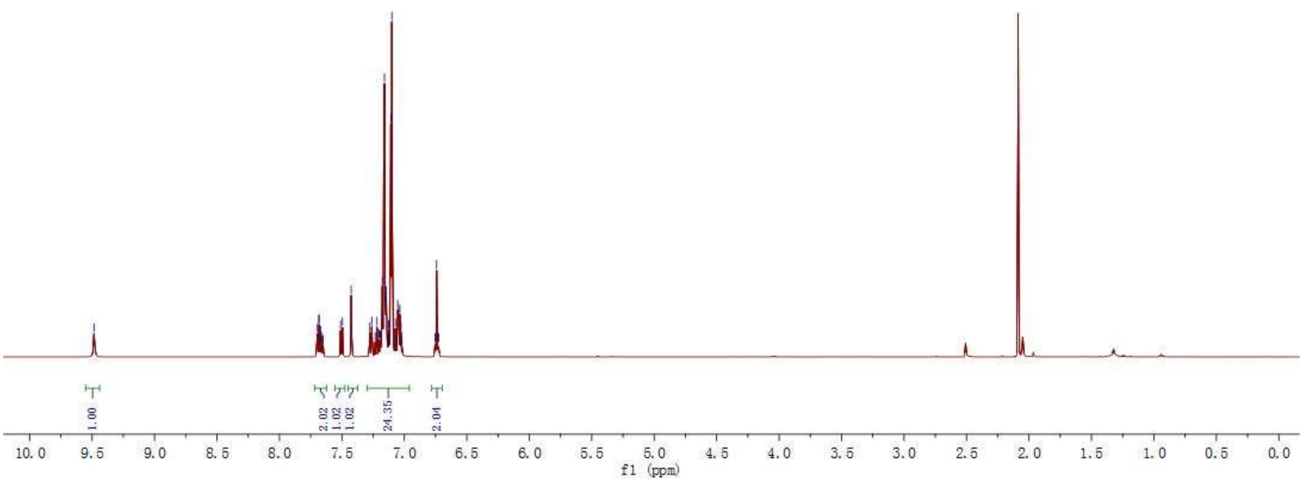

1.00
2.02
1.02
1.02
24.35
2.04
f1 (ppm)

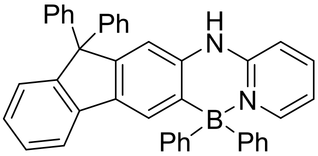

16

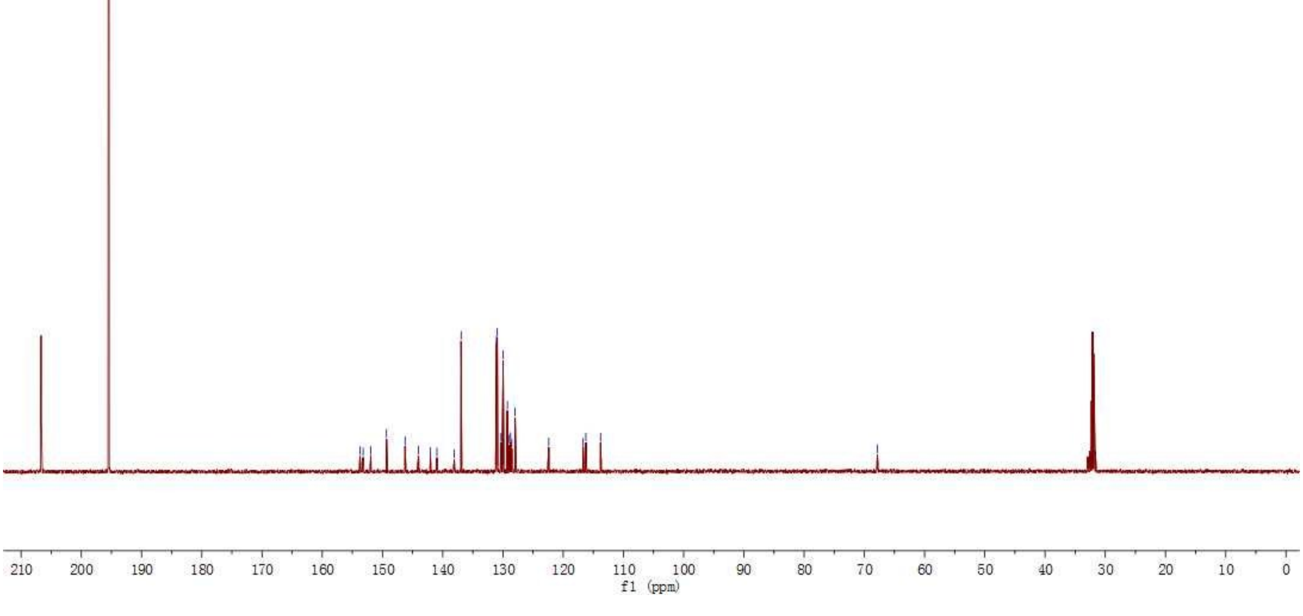

153.74
153.22
151.93
149.34
146.25
144.01
142.05
140.96
138.09
136.94
131.08
130.94
129.97
129.26
116.69
116.23
113.79
67.83
f1 (ppm)

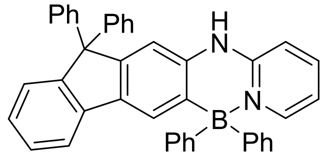

16

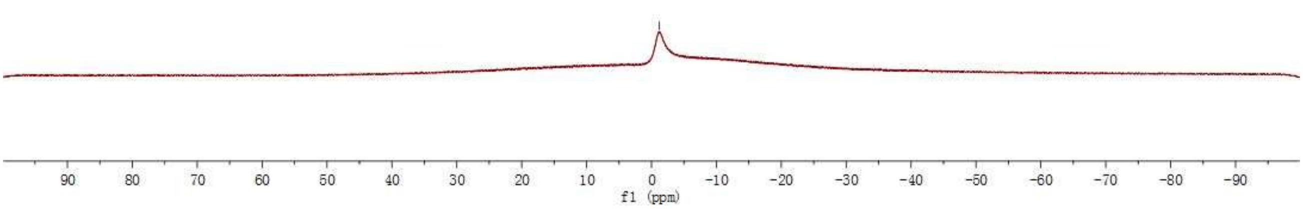

1.18
90
80
70
60
50
40
30
20
10
0
-10
-20
-30
-40
-50
-60
-70
-80
-90
f1 (ppm)

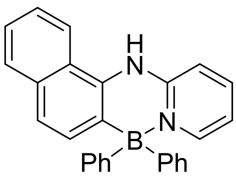

17

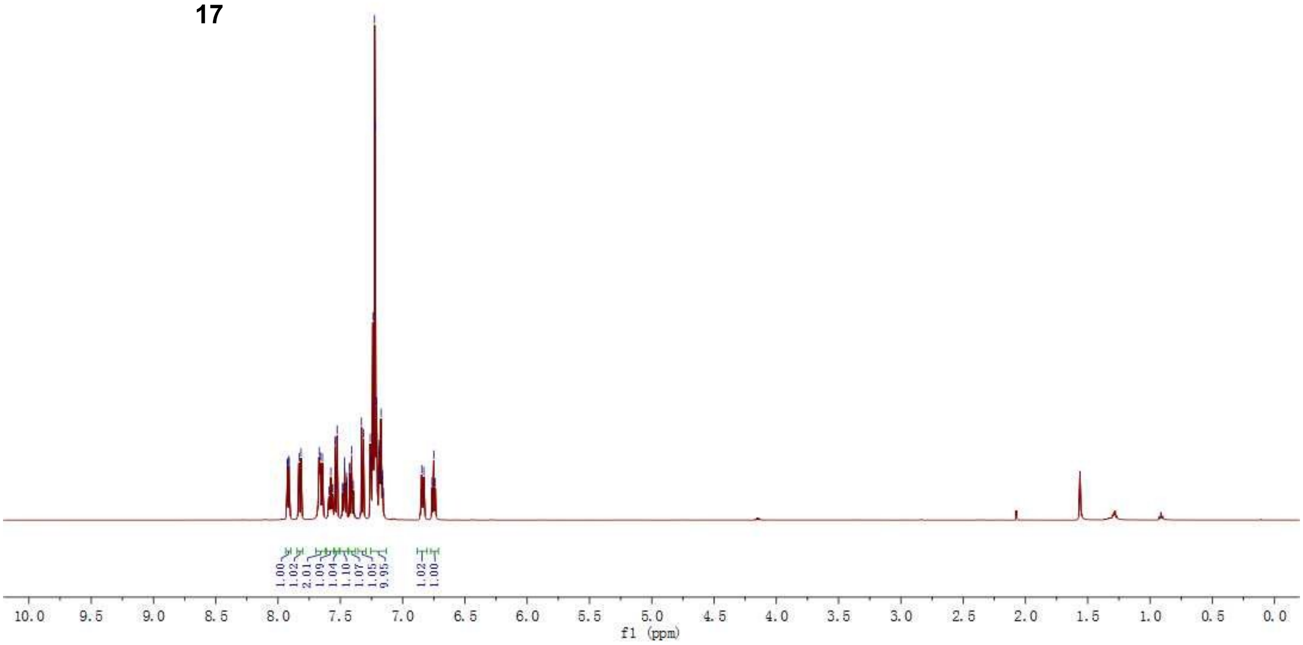

7.93
7.92
7.91
7.83
7.82
7.67
7.66
7.64
7.58
7.54
7.53
7.47
7.43
7.42
7.41
7.33
7.31
7.26
7.25
7.24
7.22
7.22
7.21
7.19
7.19
7.18
7.17
6.83
6.76
6.75
6.74
1.00
1.02
2.01
1.09
1.04
1.10
1.07
1.05
9.95
1.02
1.00
10.0
9.5
9.0
8.5
8.0
7.5
7.0
6.5
6.0
5.5
5.0
4.5
4.0
3.5
3.0
2.5
2.0
1.5
1.0
0.5
0.0
f1 (ppm)

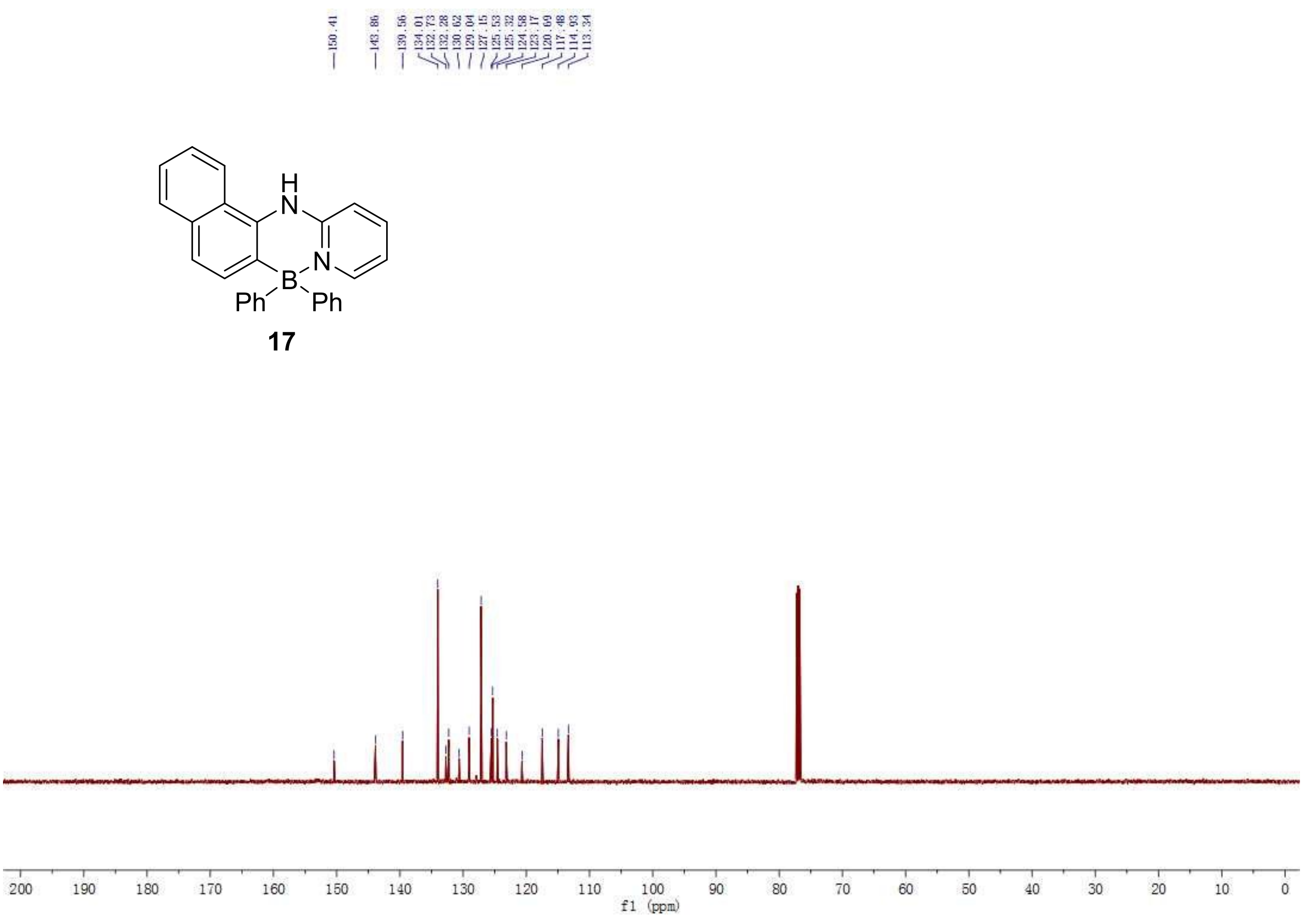
150.41
143.86
139.56
134.01
132.73
132.28
130.62
129.04
127.15
125.53
125.32
124.58
123.17
120.69
117.48
114.93
113.34
H
N
N
B
Ph
Ph
17
200 190 180 170 160 150 140 130 120 110 100 90 80 70 60 50 40 30 20 10 0
f1 (ppm)

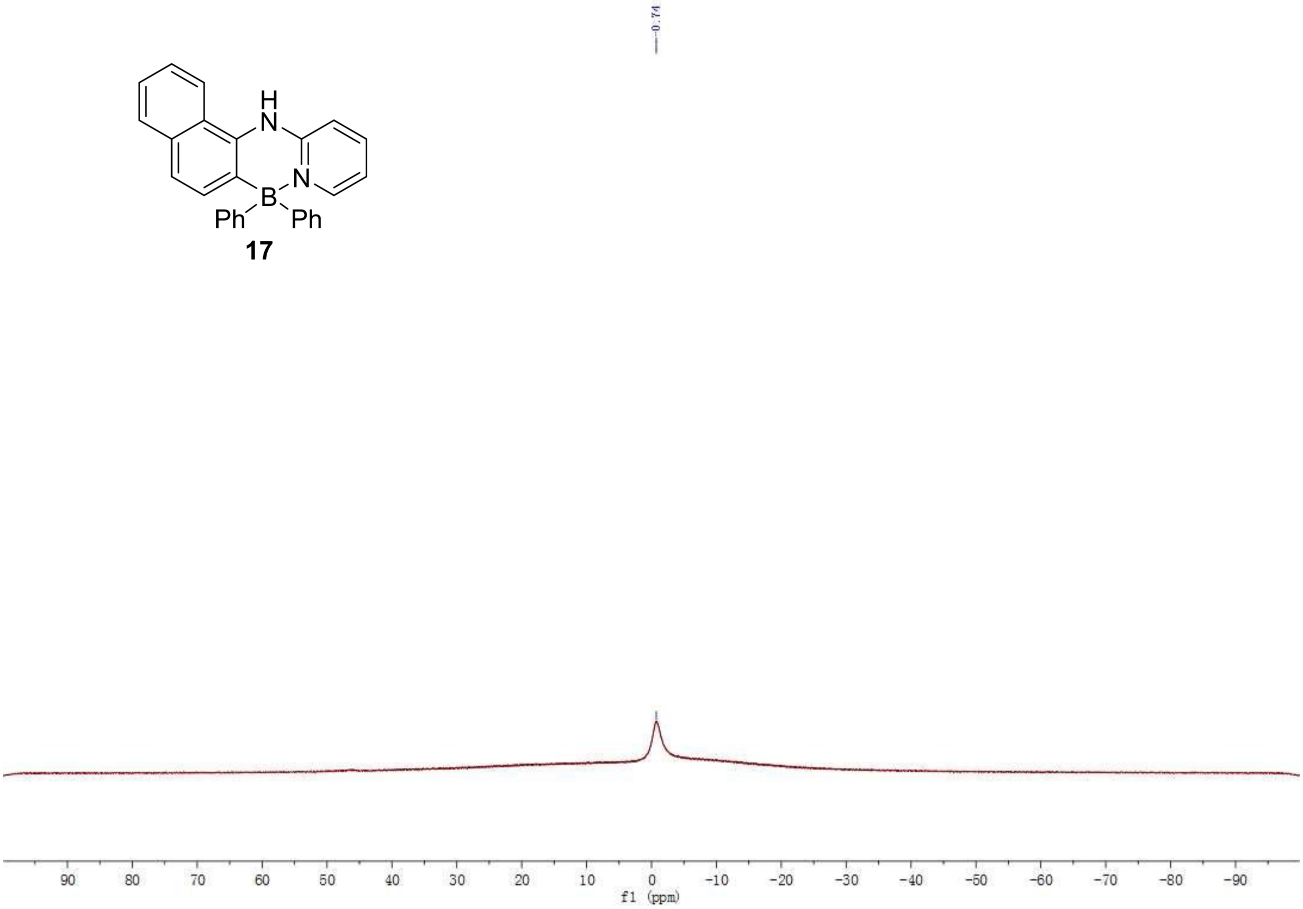
0.74
H
N
N
B
Ph
Ph
17
90 80 70 60 50 40 30 20 10 0 -10 -20 -30 -40 -50 -60 -70 -80 -90
f1 (ppm)

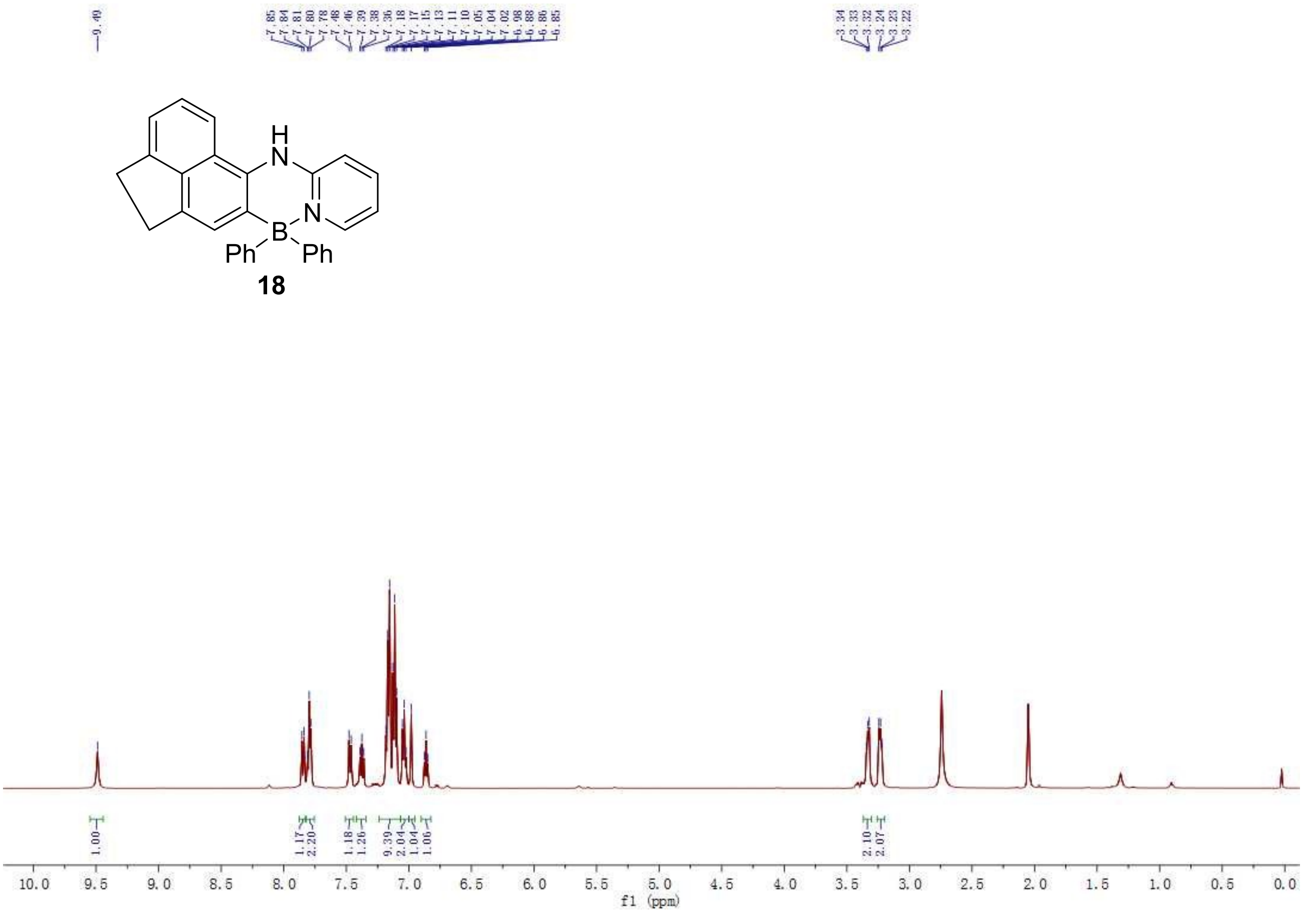
9.49
7.85 7.84 7.81 7.80 7.78 7.48 7.46 7.39 7.38 7.36 7.18 7.17 7.15 7.13 7.11 7.10 7.05 7.04 7.02 6.98 6.88 6.86 6.85
3.34 3.33 3.32 3.24 3.23 3.22
H
N
N
B
Ph
Ph
18
1.00
1.17
2.20
1.18
1.26
9.39
2.04
1.04
1.06
2.10
2.07
10.0 9.5 9.0 8.5 8.0 7.5 7.0 6.5 6.0 5.5 5.0 4.5 4.0 3.5 3.0 2.5 2.0 1.5 1.0 0.5 0.0
f1 (ppm)

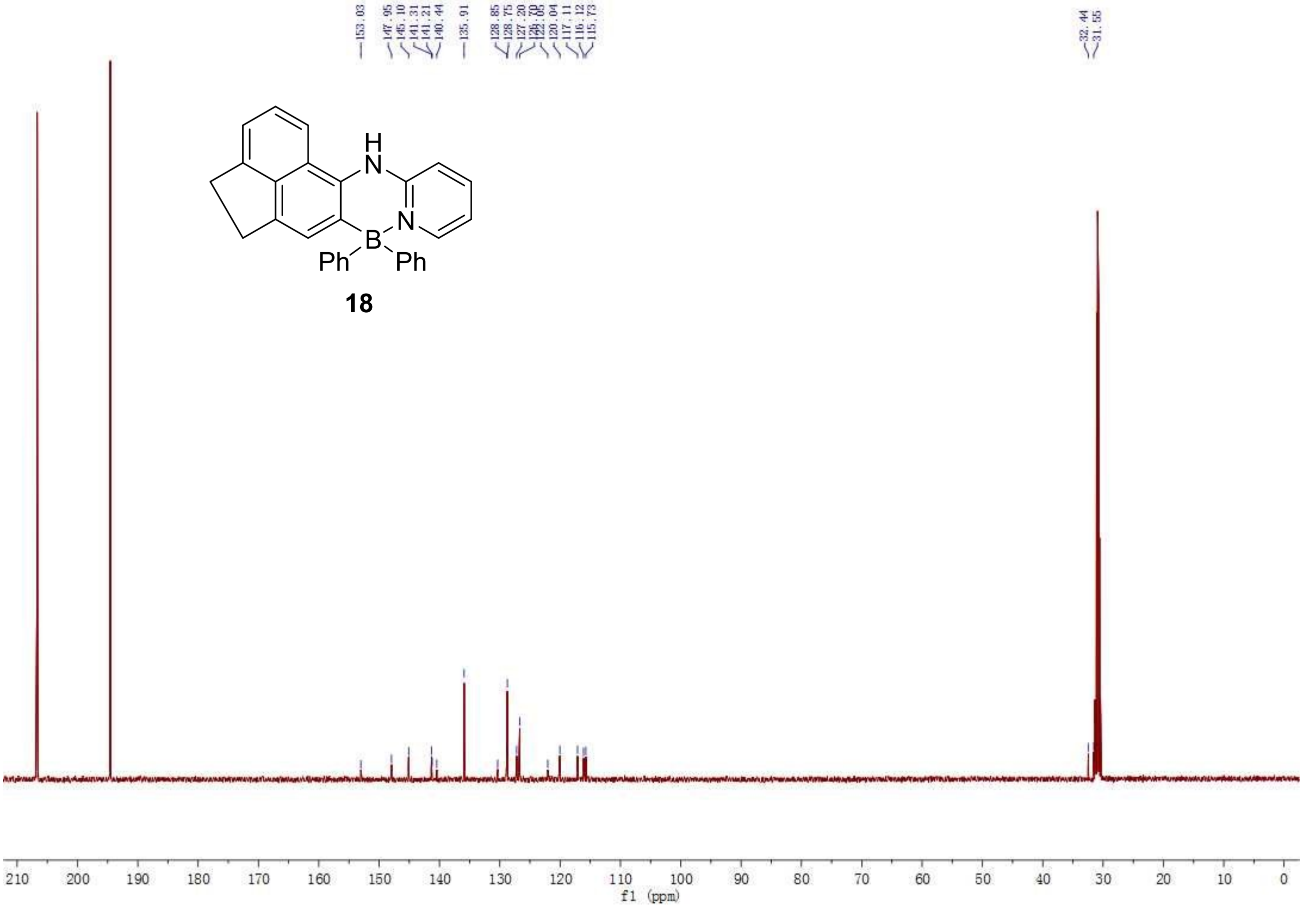
153.03
147.95 145.10 141.31 141.21 140.44
135.91
128.85 128.75 127.20 126.70 122.05 120.04 117.11 116.12 115.73
32.44 31.55
H
N
N
B
Ph
Ph
18
210 200 190 180 170 160 150 140 130 120 110 100 90 80 70 60 50 40 30 20 10 0
f1 (ppm)

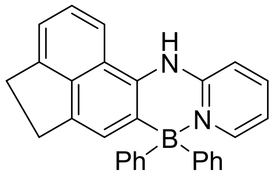

18

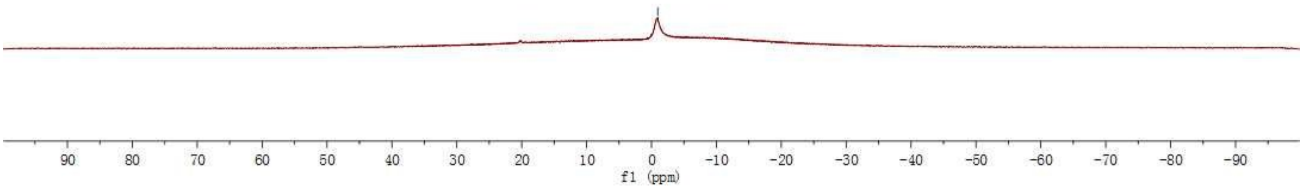

0.96
90 80 70 60 50 40 30 20 10 0 -10 -20 -30 -40 -50 -60 -70 -80 -90
f1 (ppm)

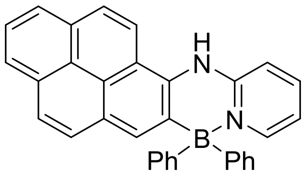

19

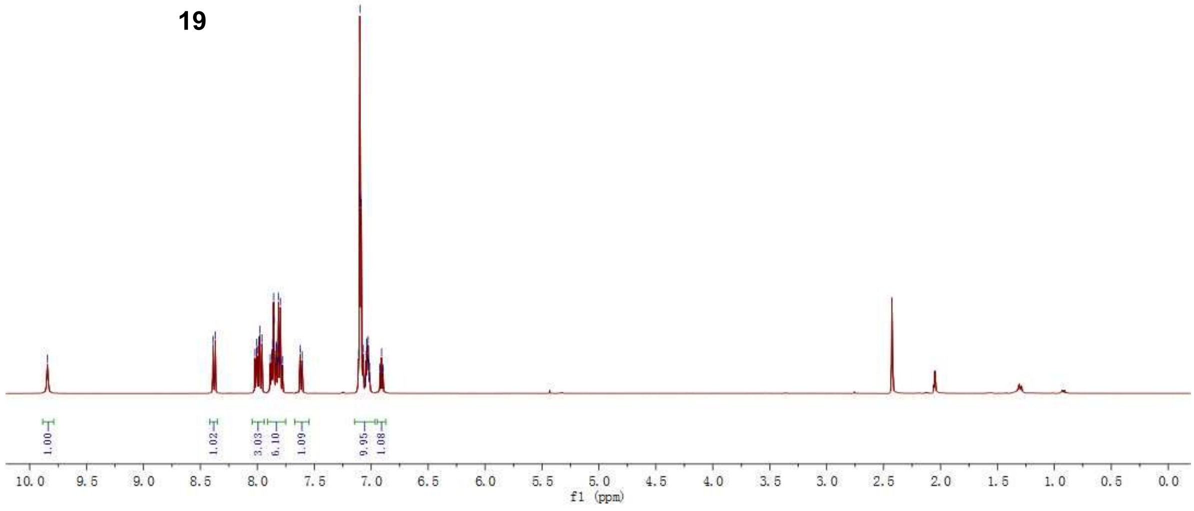

9.84
8.39 8.37
8.01 7.98 7.98 7.96 7.86 7.85 7.81 7.80 7.11 7.10 7.09 7.09 7.07 7.05 7.05 7.04 7.04 7.03 7.03 7.03 7.02 7.02 7.01 7.01 6.92 6.92 6.91 6.90 6.89
1.00 1.02 3.03 6.10 1.09 9.95 1.08
10.0 9.5 9.0 8.5 8.0 7.5 7.0 6.5 6.0 5.5 5.0 4.5 4.0 3.5 3.0 2.5 2.0 1.5 1.0 0.5 0.0
f1 (ppm)

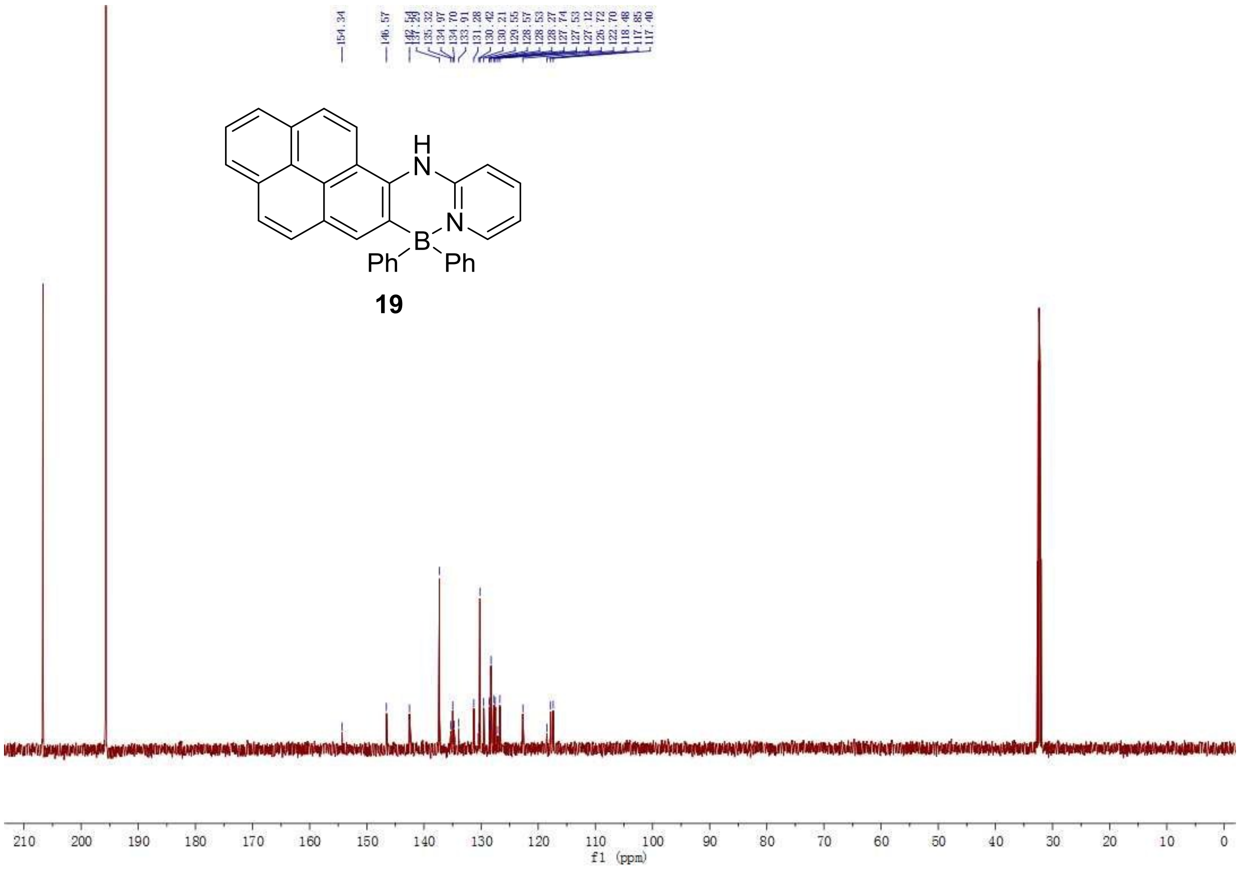

154.34
146.57
135.32
134.97
134.70
133.91
131.28
130.42
130.21
129.55
128.57
128.53
128.27
127.74
127.53
127.12
126.72
122.70
118.48
117.85
117.40
H
N
N
B
Ph
Ph
19
f1 (ppm)

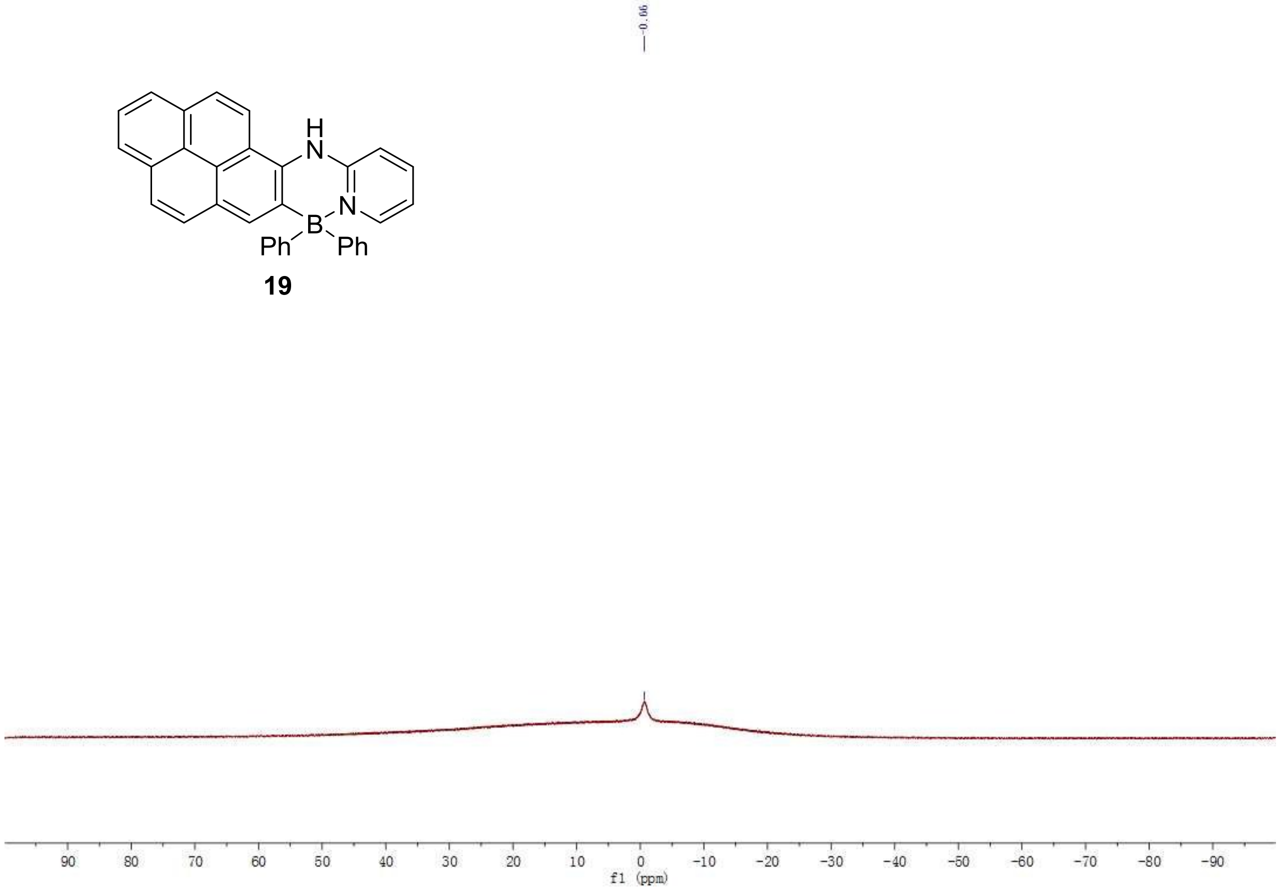

0.66
H
N
N
B
Ph
Ph
19
f1 (ppm)

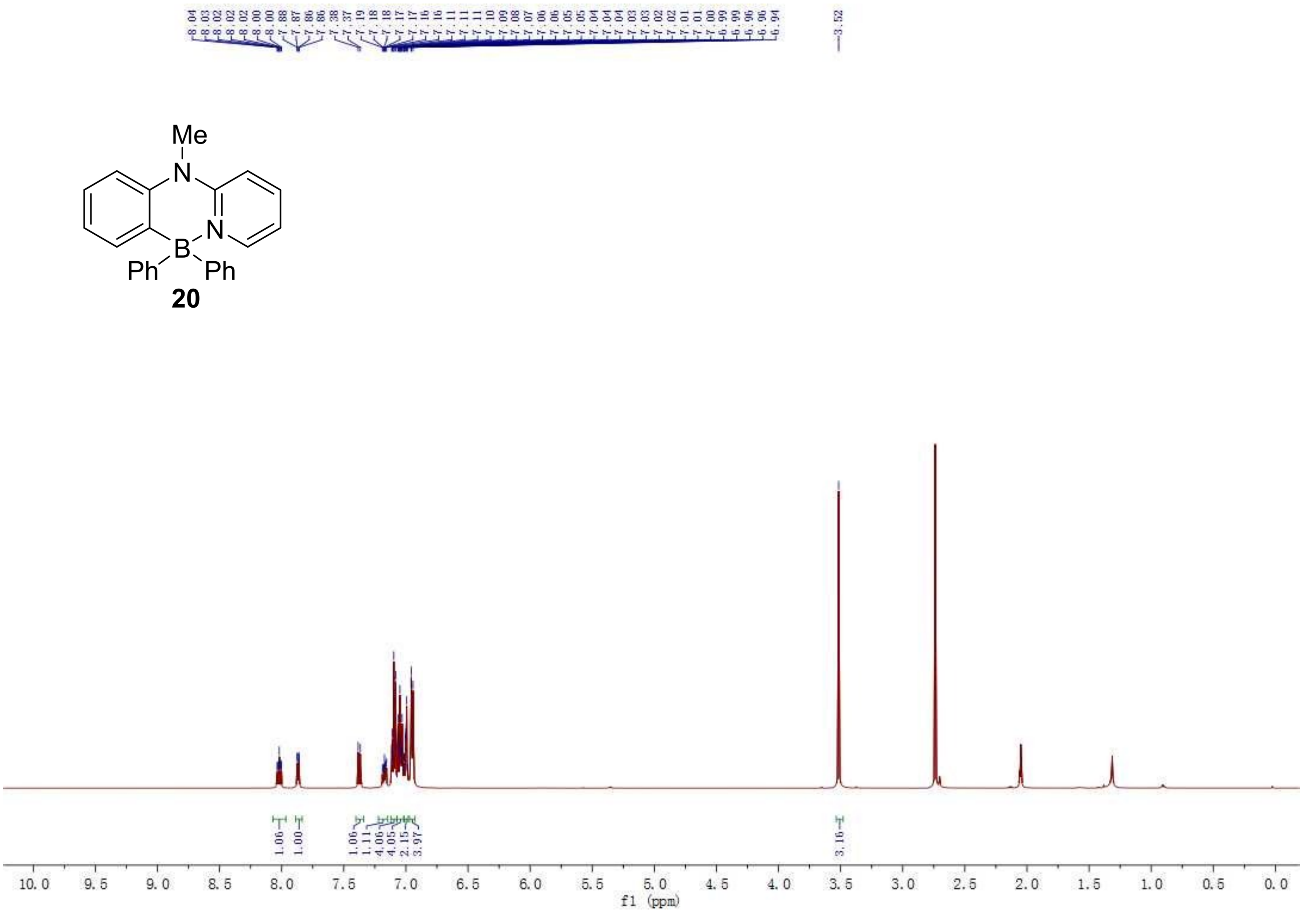

3.52
Me
N
N
B
Ph
Ph
20
1.06
1.00
1.06
1.11
4.06
4.05
2.15
3.97
3.16
10.0 9.5 9.0 8.5 8.0 7.5 7.0 6.5 6.0 5.5 5.0 4.5 4.0 3.5 3.0 2.5 2.0 1.5 1.0 0.5 0.0
f1 (ppm)

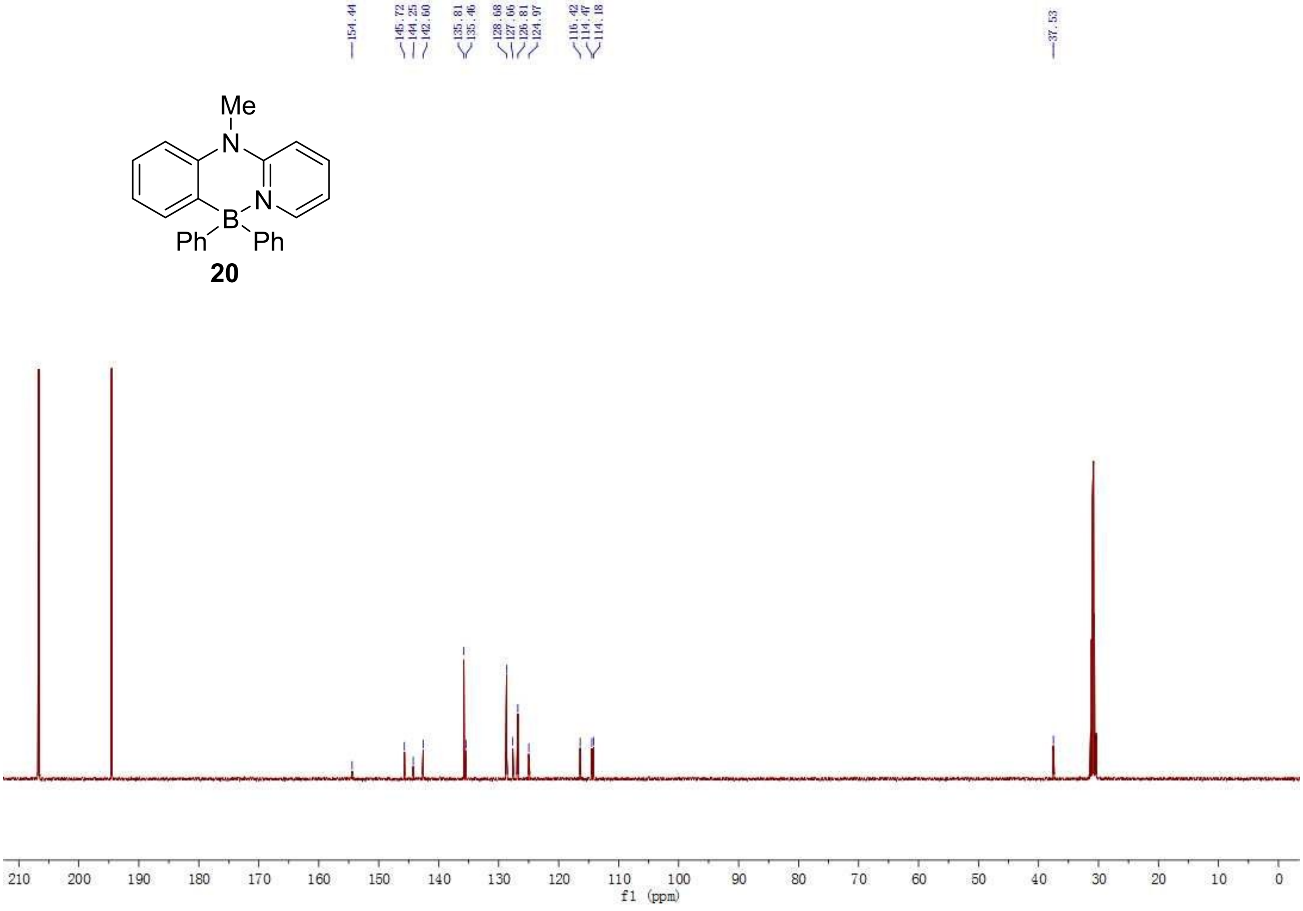

154.44
145.72
144.25
142.60
135.81
135.46
128.68
127.66
126.81
124.97
116.42
114.47
114.18
37.53
Me
N
N
B
Ph
Ph
20
210 200 190 180 170 160 150 140 130 120 110 100 90 80 70 60 50 40 30 20 10 0
f1 (ppm)

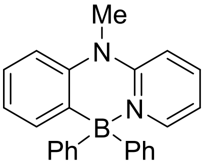
Me
N
N
B
Ph
Ph

20

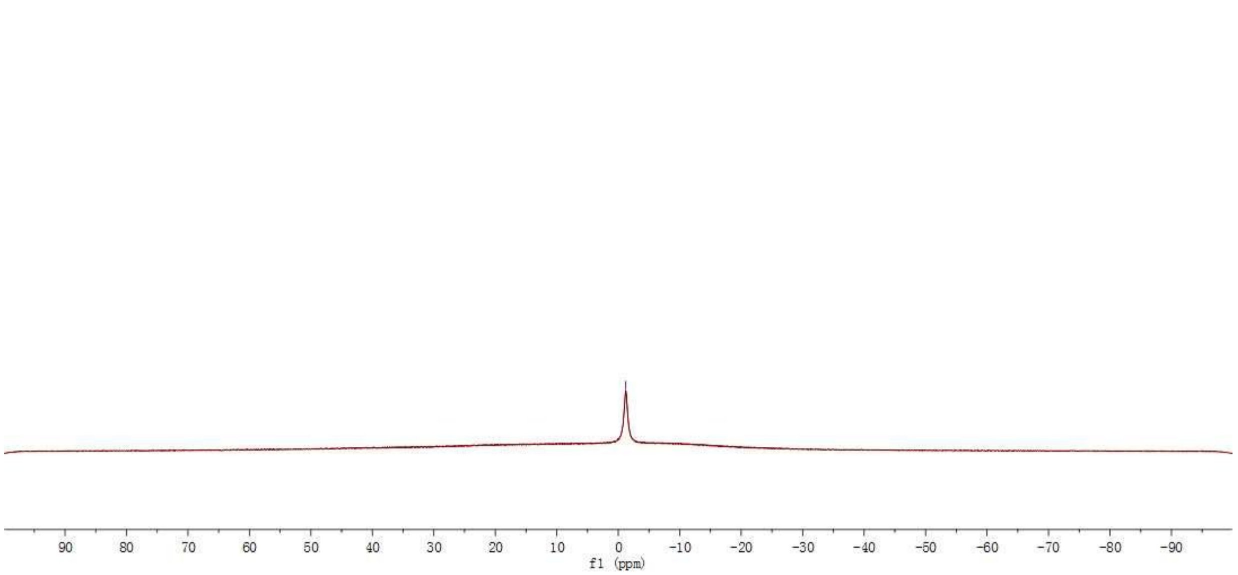
1.23
f1 (ppm)

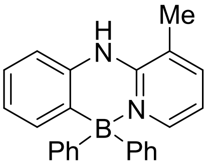
H
N
Me
N
B
Ph
Ph

21

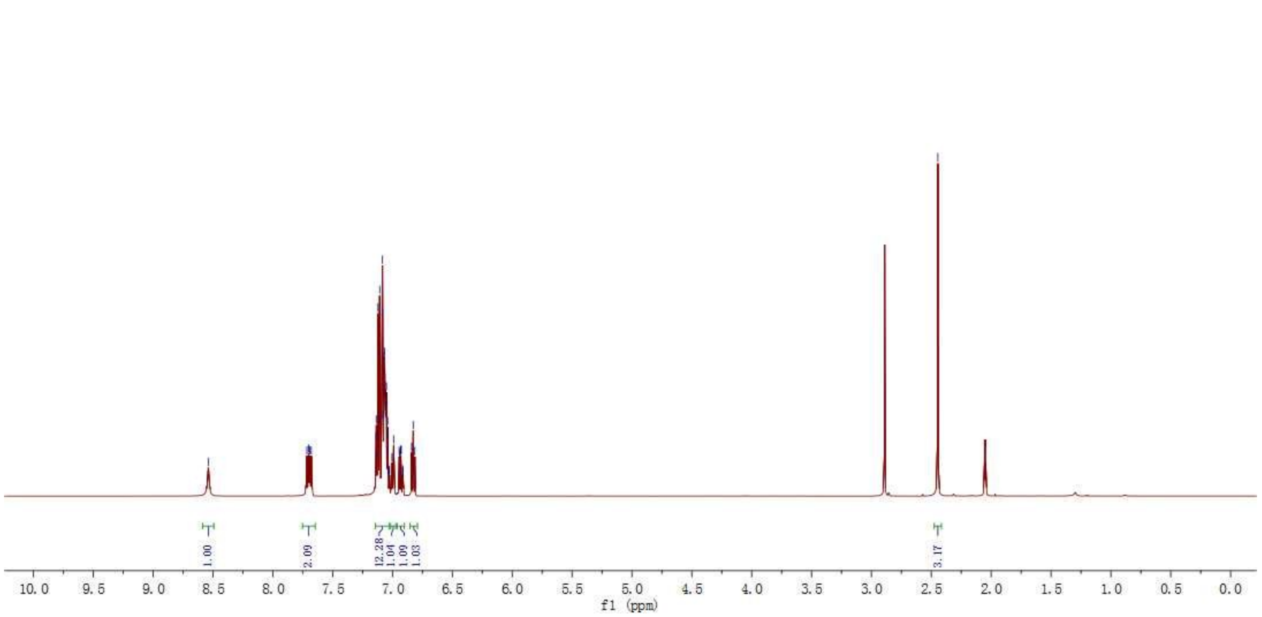
8.54
2.44
1.00
2.09
12.28
1.04
1.09
1.03
3.17
f1 (ppm)

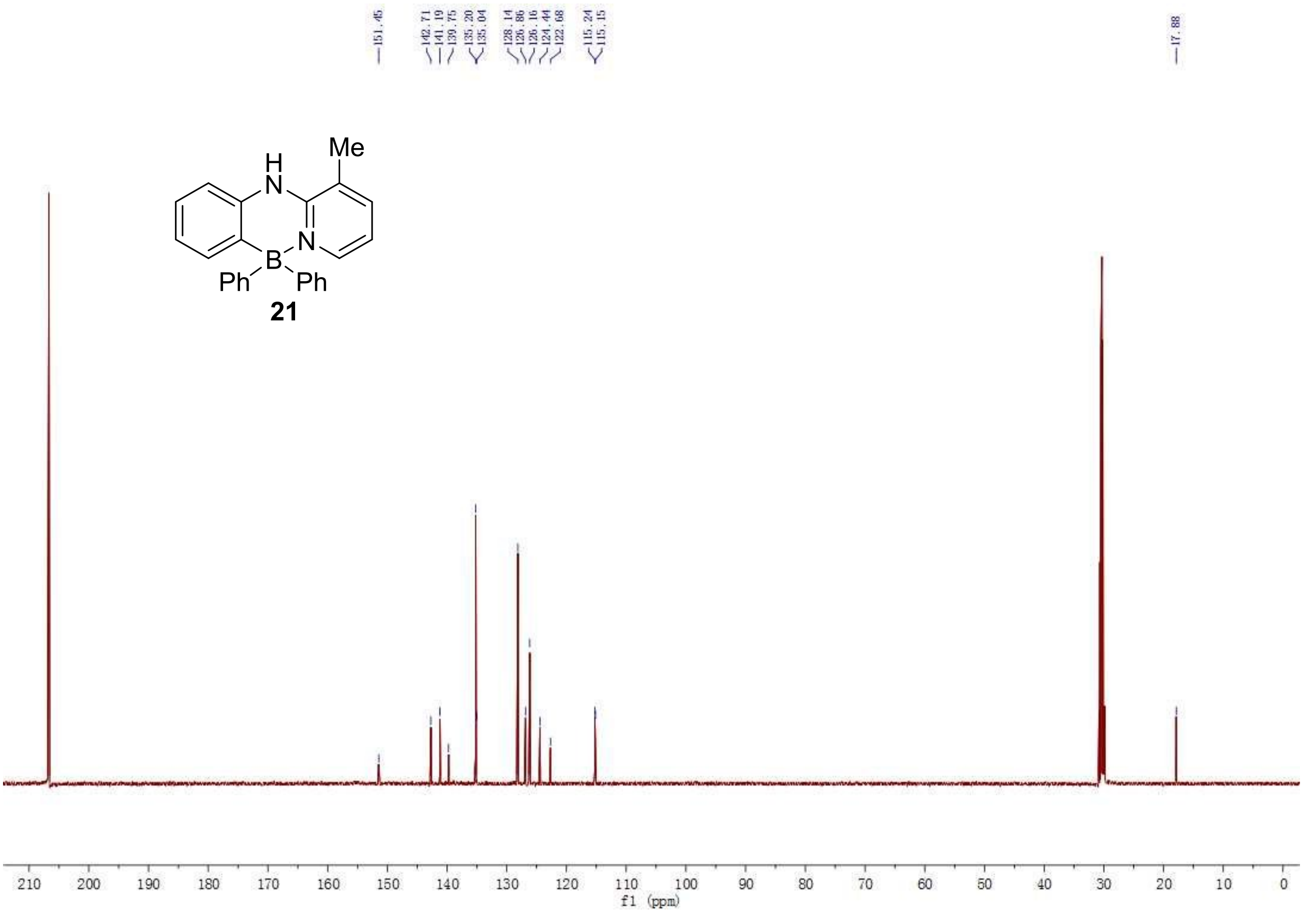

21

151.45
142.71
141.19
139.75
135.20
135.04
128.14
126.88
126.16
124.44
122.68
115.24
115.15
17.88
210 200 190 180 170 160 150 140 130 120 110 100 90 80 70 60 50 40 30 20 10 0
f1 (ppm)

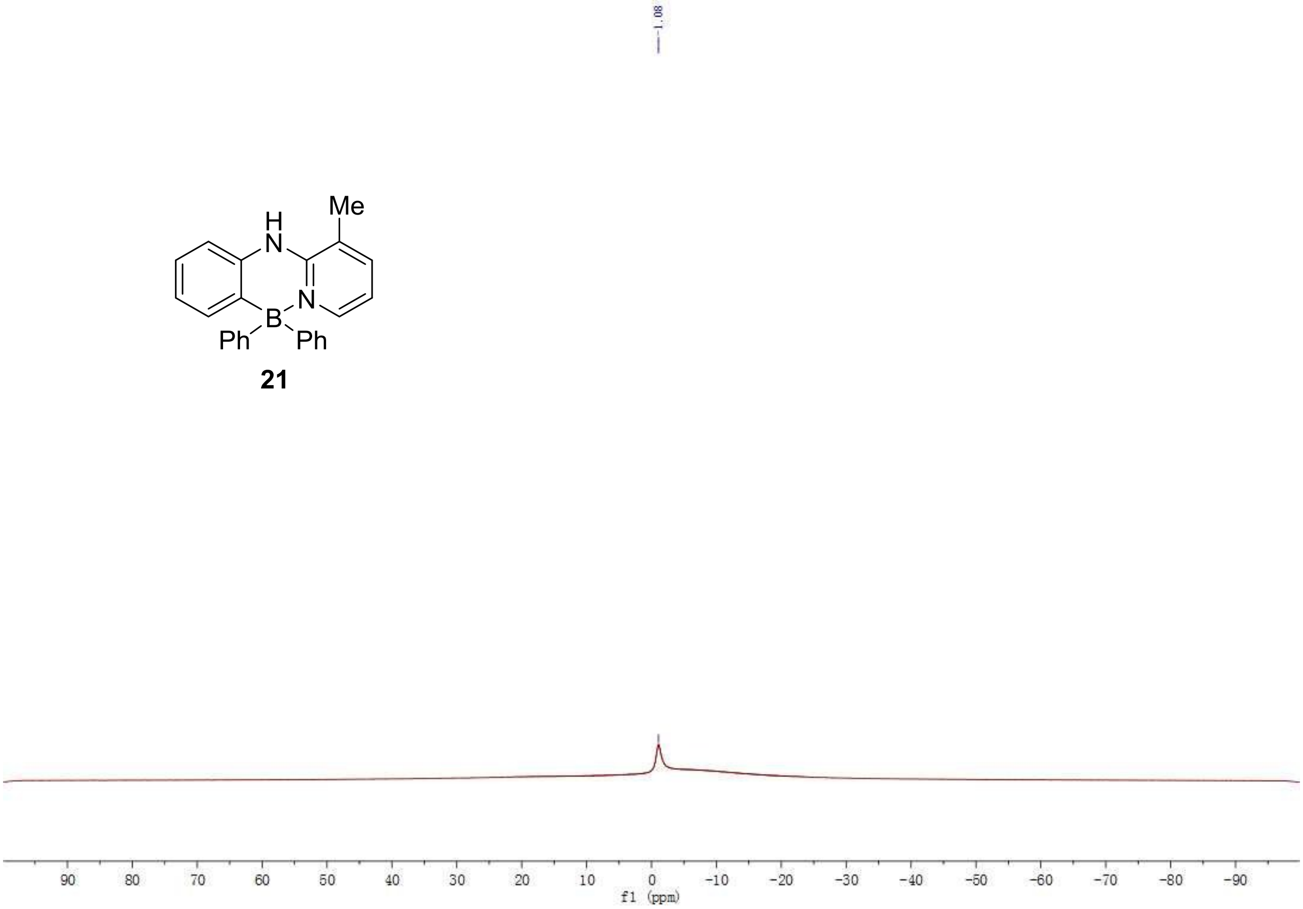

21

1.08
90 80 70 60 50 40 30 20 10 0 -10 -20 -30 -40 -50 -60 -70 -80 -90
f1 (ppm)

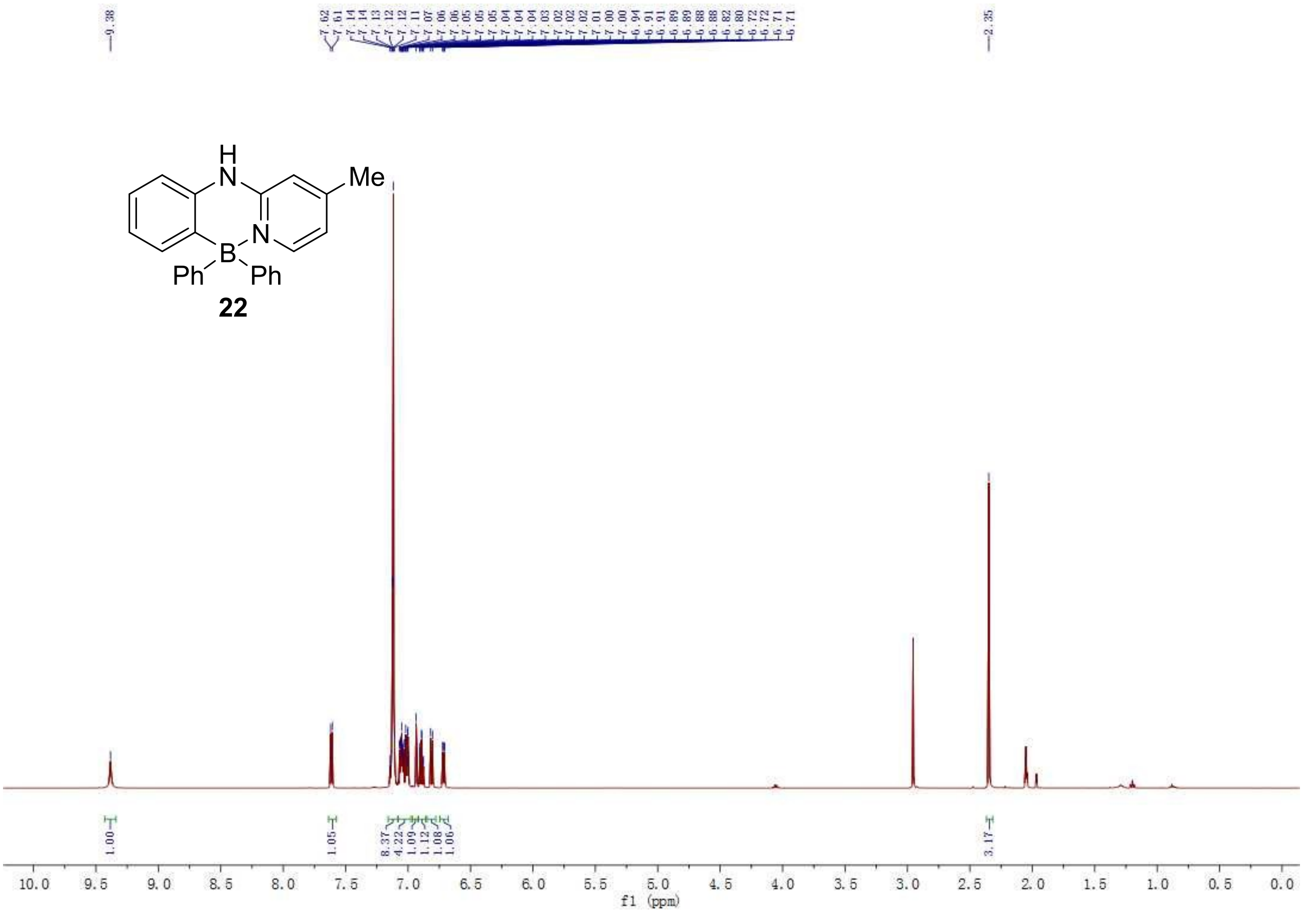

22

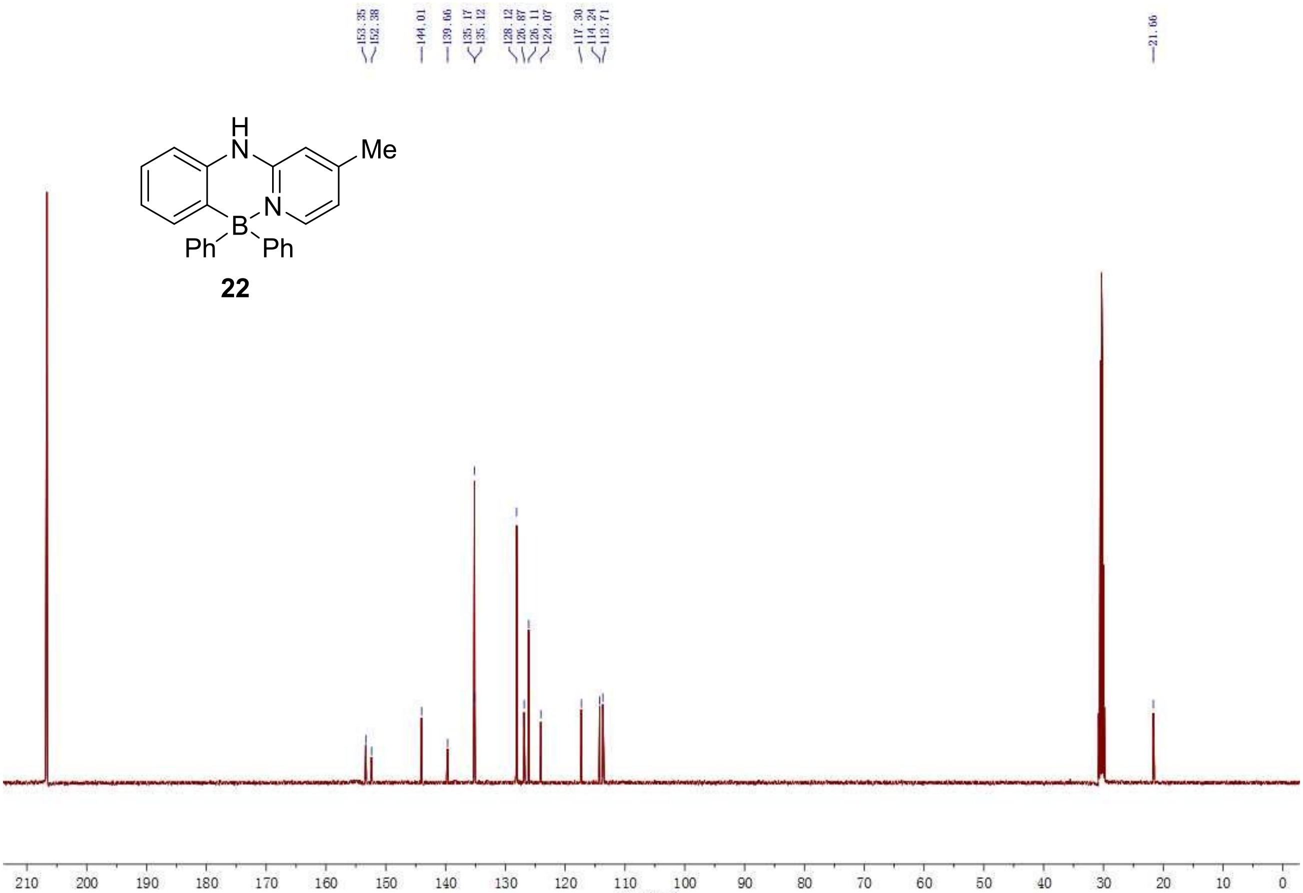

22

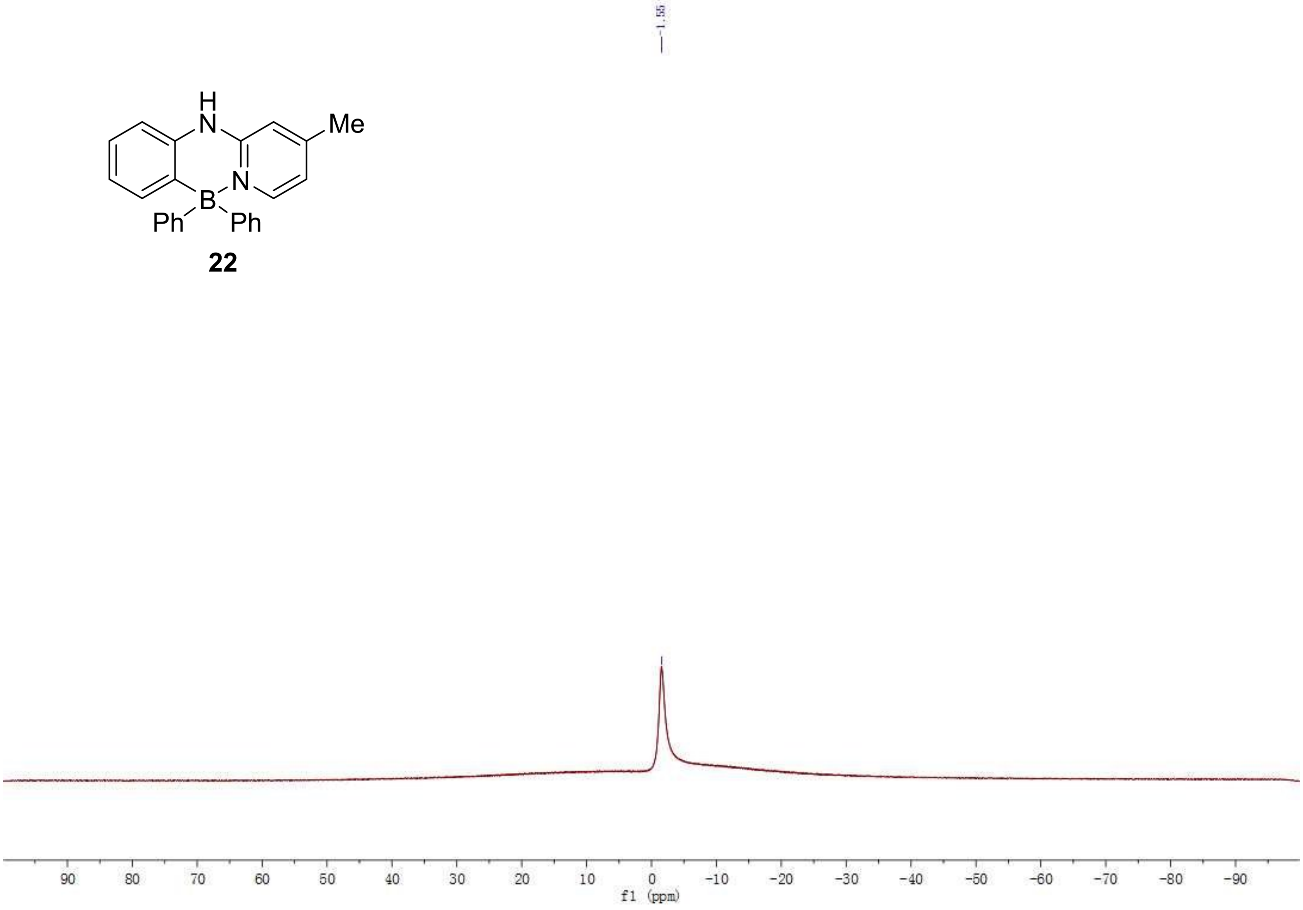

-1.55
H
N
Me
N
B
Ph
Ph
90 80 70 60 50 40 30 20 10 0 -10 -20 -30 -40 -50 -60 -70 -80 -90
f1 (ppm)

22

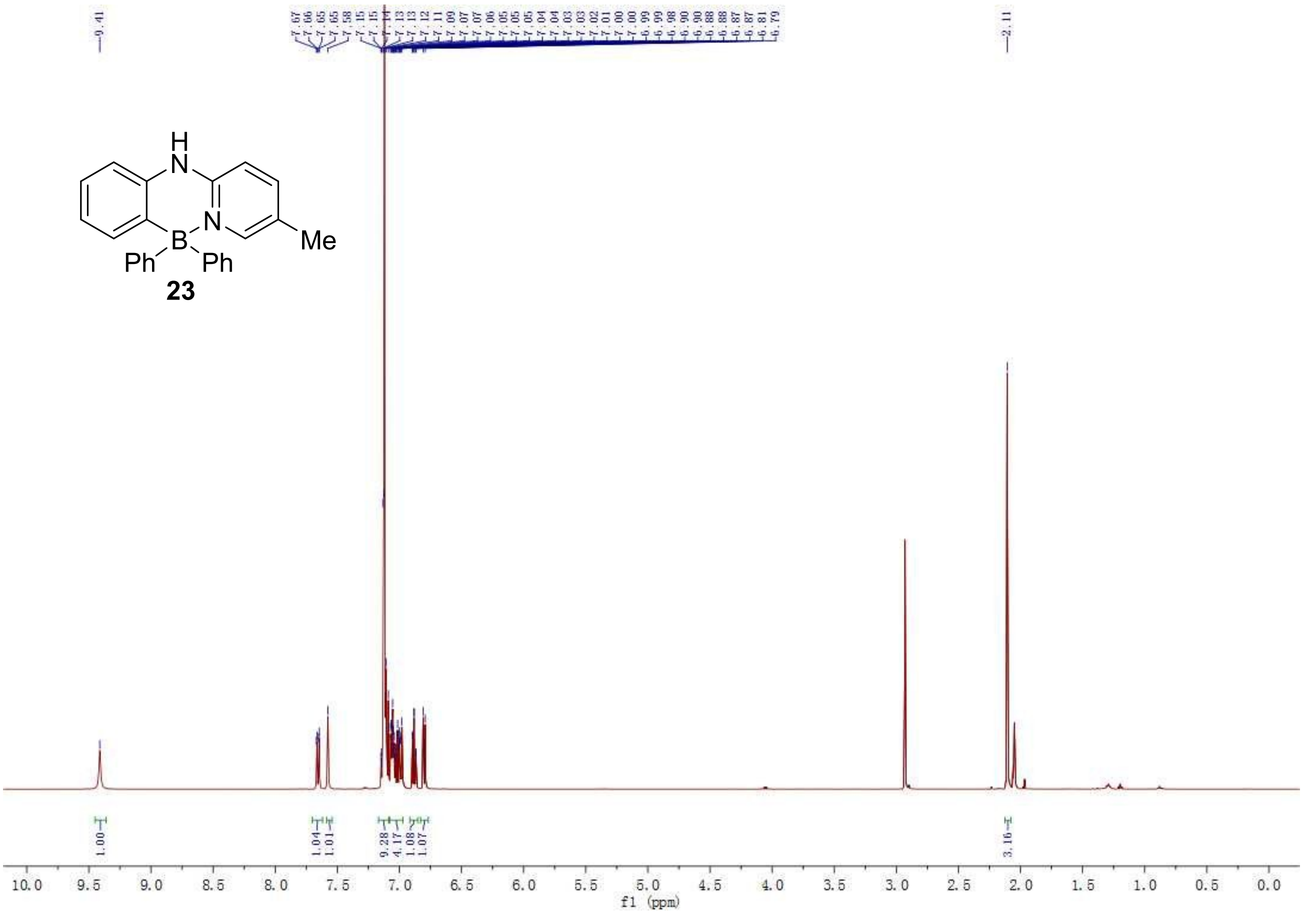

9.41
2.11
H
N
N
B
Me
Ph
Ph
1.00
1.04
1.01
9.28
4.17
1.08
1.07
3.16
10.0 9.5 9.0 8.5 8.0 7.5 7.0 6.5 6.0 5.5 5.0 4.5 4.0 3.5 3.0 2.5 2.0 1.5 1.0 0.5 0.0
f1 (ppm)

23

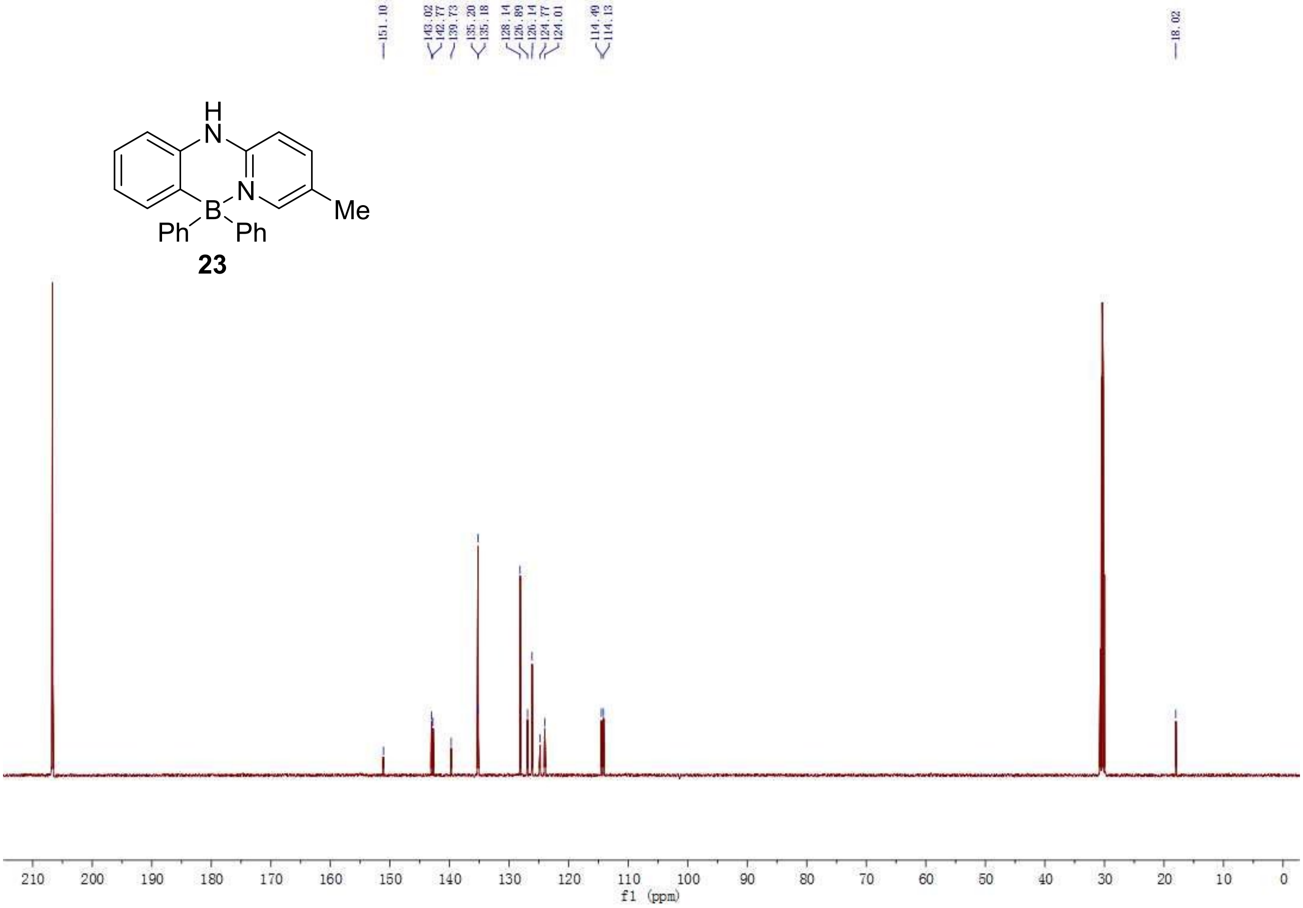
151.10
143.02
142.77
139.73
135.20
135.18
128.14
126.89
126.14
124.77
124.01
114.49
114.13
18.02
H
N
N
B
Me
Ph
Ph
23
210 200 190 180 170 160 150 140 130 120 110 100 90 80 70 60 50 40 30 20 10 0
f1 (ppm)

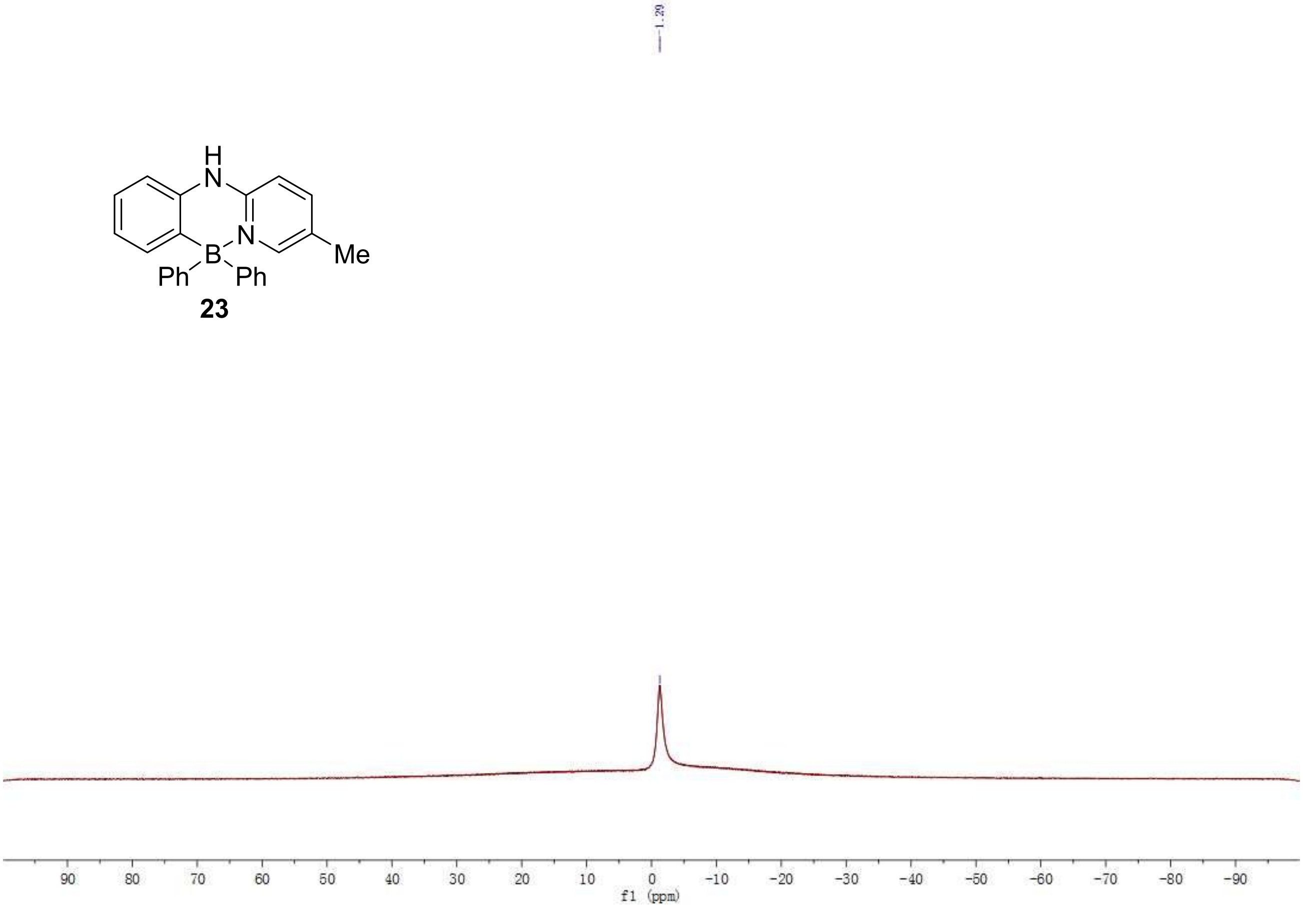
1.29
H
N
N
B
Me
Ph
Ph
23
90 80 70 60 50 40 30 20 10 0 -10 -20 -30 -40 -50 -60 -70 -80 -90
f1 (ppm)

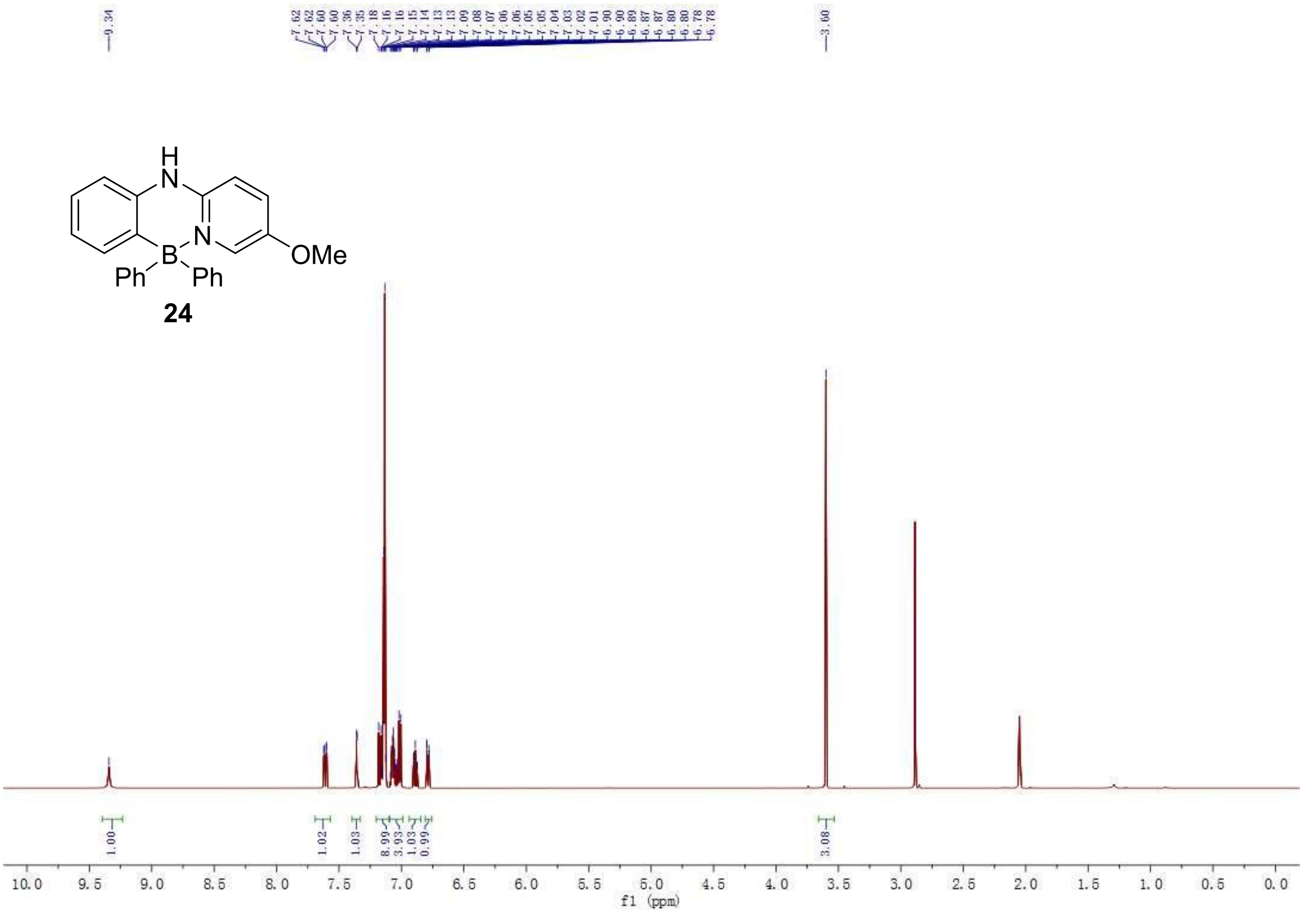

9.34
7.62
7.62
7.60
7.36
7.35
7.18
7.16
7.16
7.15
7.14
7.13
7.09
7.08
7.07
7.06
7.06
7.05
7.05
7.04
7.03
7.02
7.01
6.90
6.89
6.87
6.80
6.80
6.78
3.60
H
N
N
B
Ph
Ph
OMe
24
1.00
1.02
1.03
8.99
3.93
1.03
0.99
3.08
10.0 9.5 9.0 8.5 8.0 7.5 7.0 6.5 6.0 5.5 5.0 4.5 4.0 3.5 3.0 2.5 2.0 1.5 1.0 0.5 0.0
f1 (ppm)

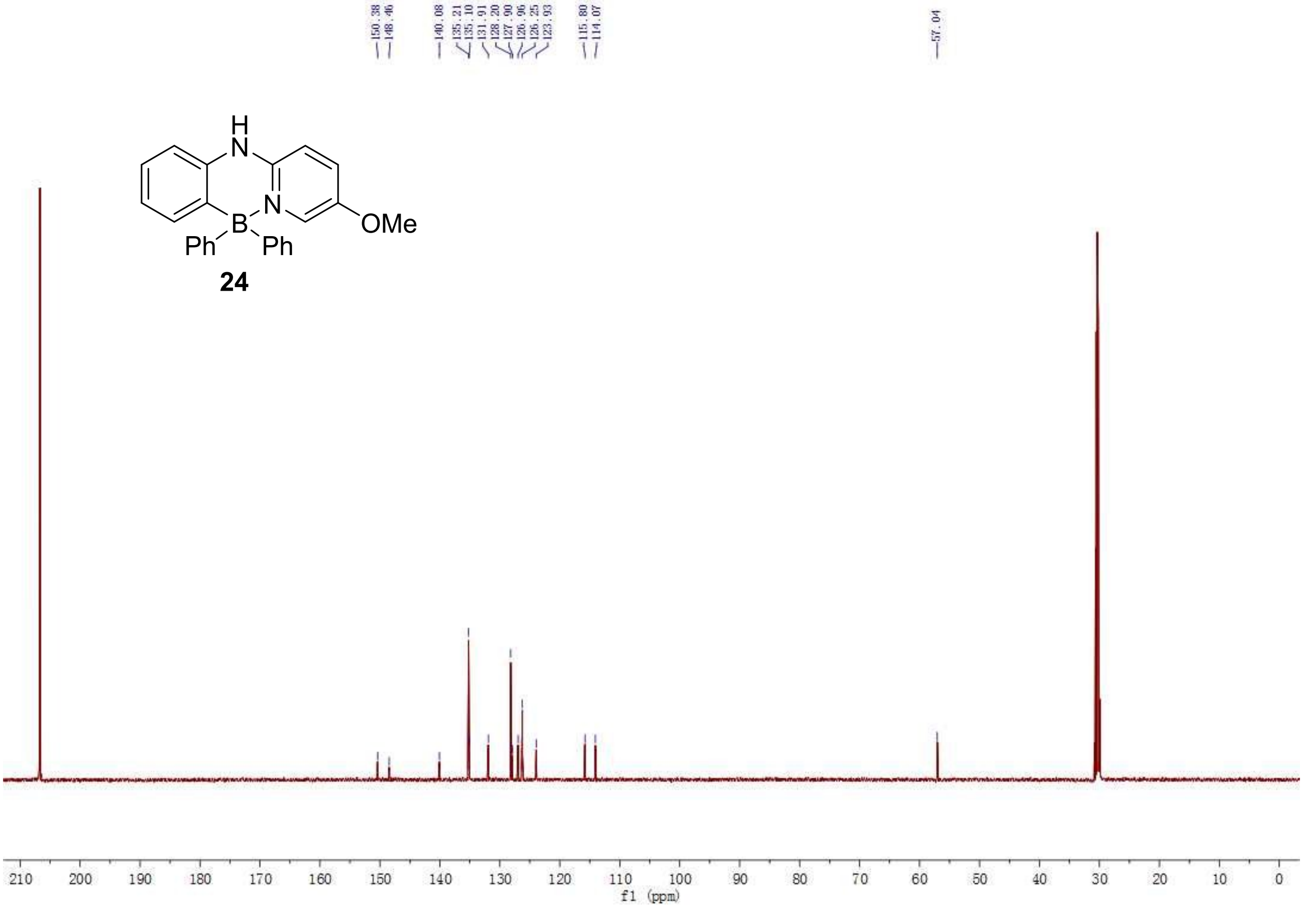

150.38
148.46
140.08
135.21
135.10
131.91
128.20
127.90
126.96
126.25
123.93
115.80
114.07
57.04
H
N
N
B
Ph
Ph
OMe
24
210 200 190 180 170 160 150 140 130 120 110 100 90 80 70 60 50 40 30 20 10 0
f1 (ppm)

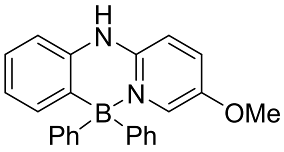

24

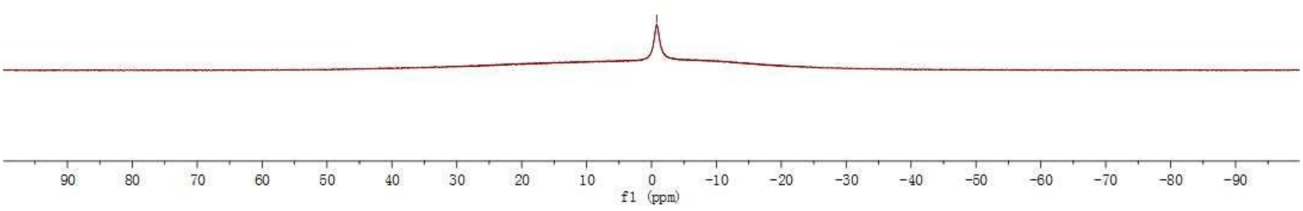

-0.80
f1 (ppm)

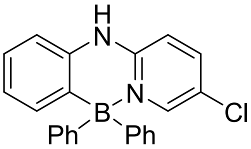

25

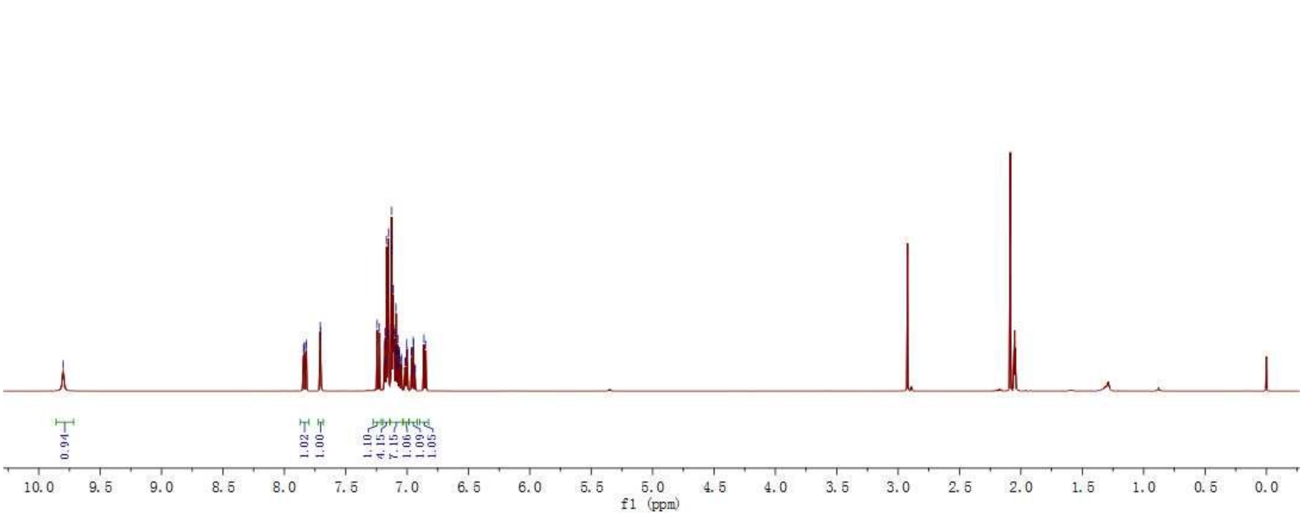

9.80
7.84 7.84 7.83 7.82 7.71 7.70 7.24 7.23 7.18 7.18 7.17 7.16 7.15 7.13 7.13 7.12 7.11 7.10 7.10 7.09 7.09 7.08 7.08 7.08 7.07 7.06 7.06 7.05 7.05 7.02 7.01 7.00 6.96 6.95 6.95 6.94 6.93 6.86 6.85
0.94
1.02
1.00
1.10
4.15
7.15
1.06
1.09
1.05
f1 (ppm)

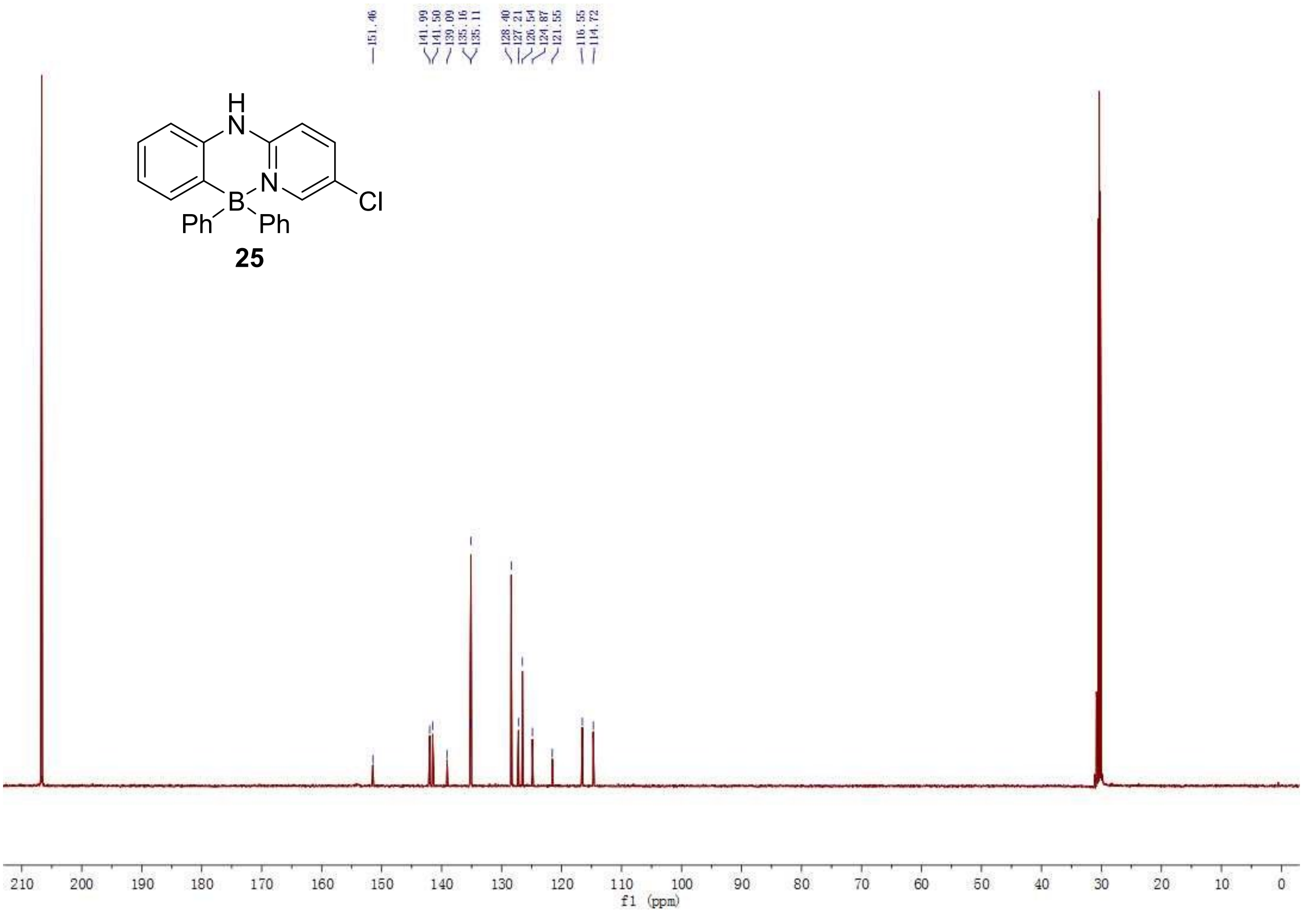
151.46
141.99
141.50
139.09
135.16
135.11
128.40
127.21
126.54
124.87
121.55
116.55
114.72
H
N
N
B
Cl
Ph
Ph
25
210 200 190 180 170 160 150 140 130 120 110 100 90 80 70 60 50 40 30 20 10 0
f1 (ppm)

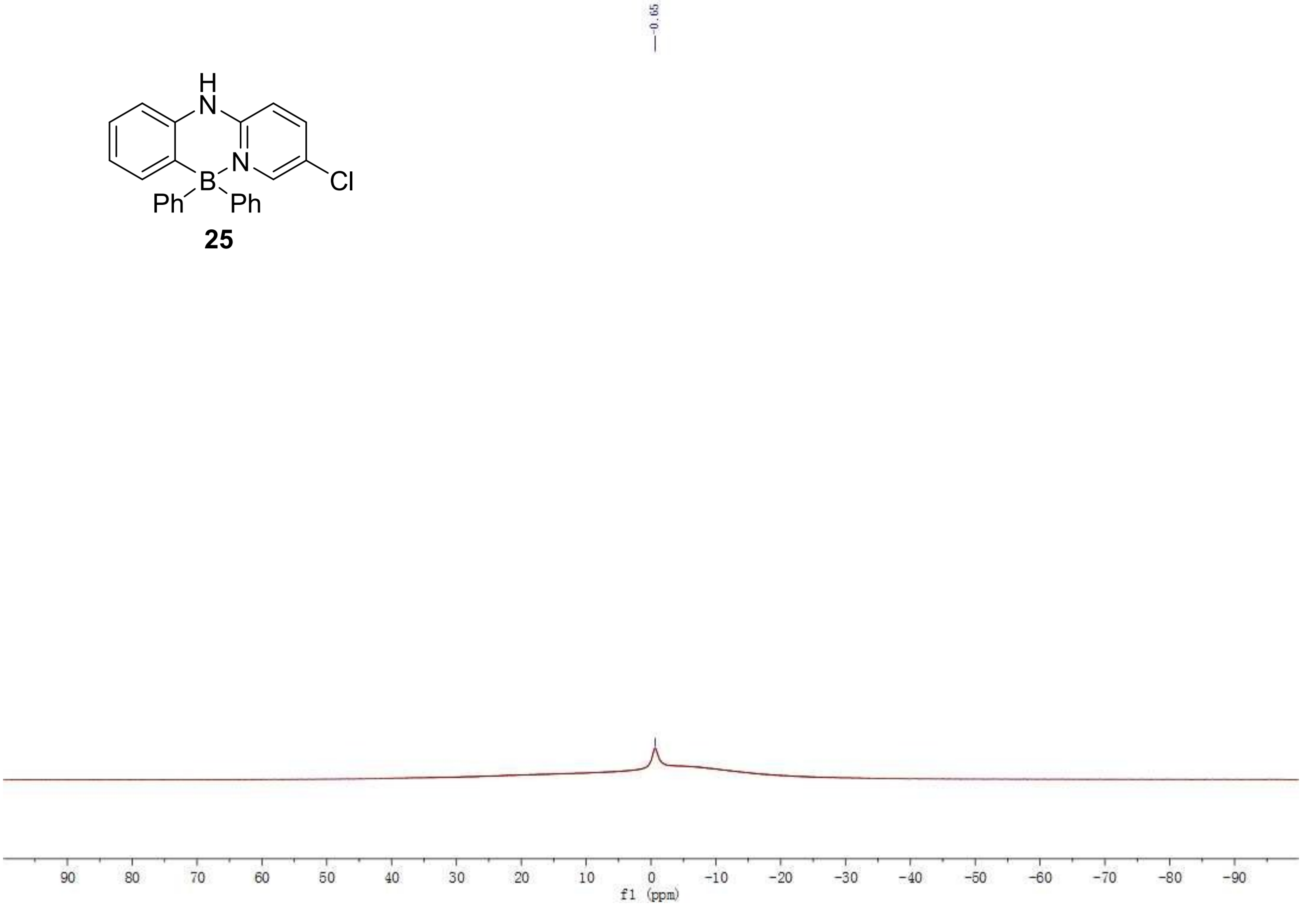
0.65
H
N
N
B
Cl
Ph
Ph
25
90 80 70 60 50 40 30 20 10 0 -10 -20 -30 -40 -50 -60 -70 -80 -90
f1 (ppm)

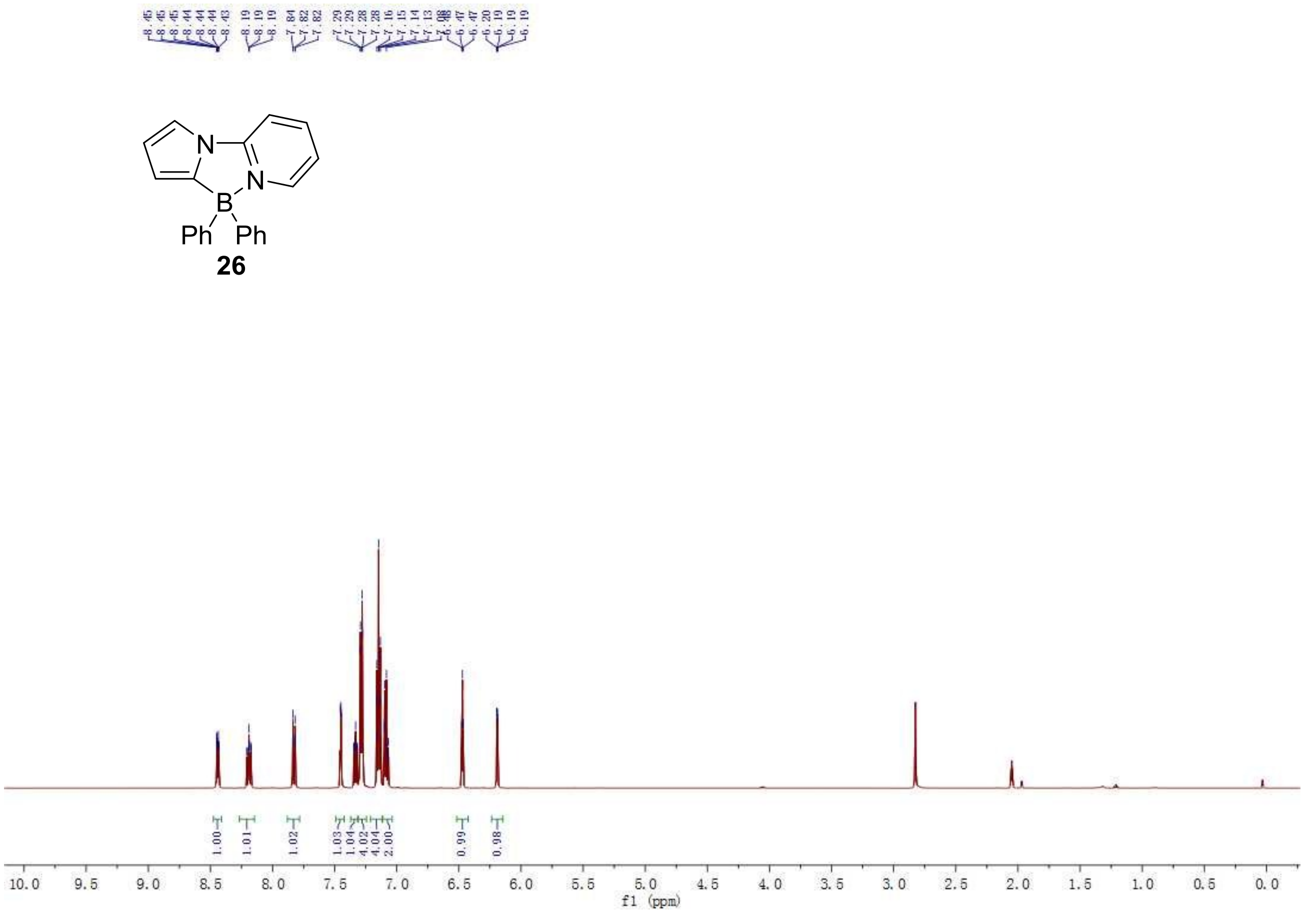
8.45 8.45 8.45 8.44 8.44 8.44 8.43
8.19 8.19 8.19
7.84 7.82 7.82
7.29 7.29 7.28 7.28 7.16 7.15 7.14 7.13
6.47 6.47
6.20 6.19 6.19 6.19
N
N
B
Ph Ph
26
1.00 1.01 1.02 1.03 1.04 4.02 4.04 2.00 0.99 0.98
10.0 9.5 9.0 8.5 8.0 7.5 7.0 6.5 6.0 5.5 5.0 4.5 4.0 3.5 3.0 2.5 2.0 1.5 1.0 0.5 0.0
f1 (ppm)

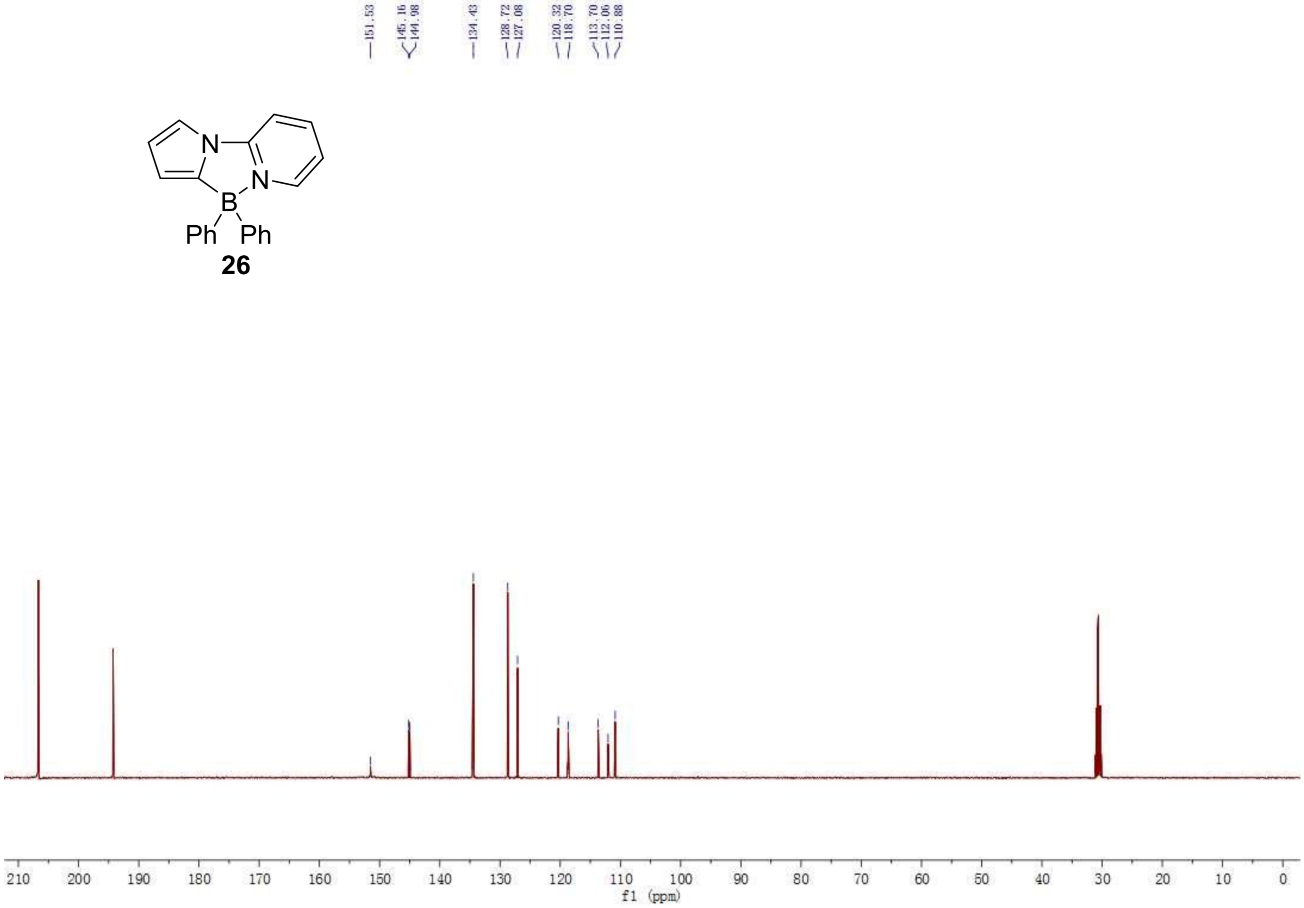
151.53
145.16 144.98
134.43
128.72 127.08
120.32 118.70
113.70 112.06 110.88
N
N
B
Ph Ph
26
210 200 190 180 170 160 150 140 130 120 110 100 90 80 70 60 50 40 30 20 10 0
f1 (ppm)

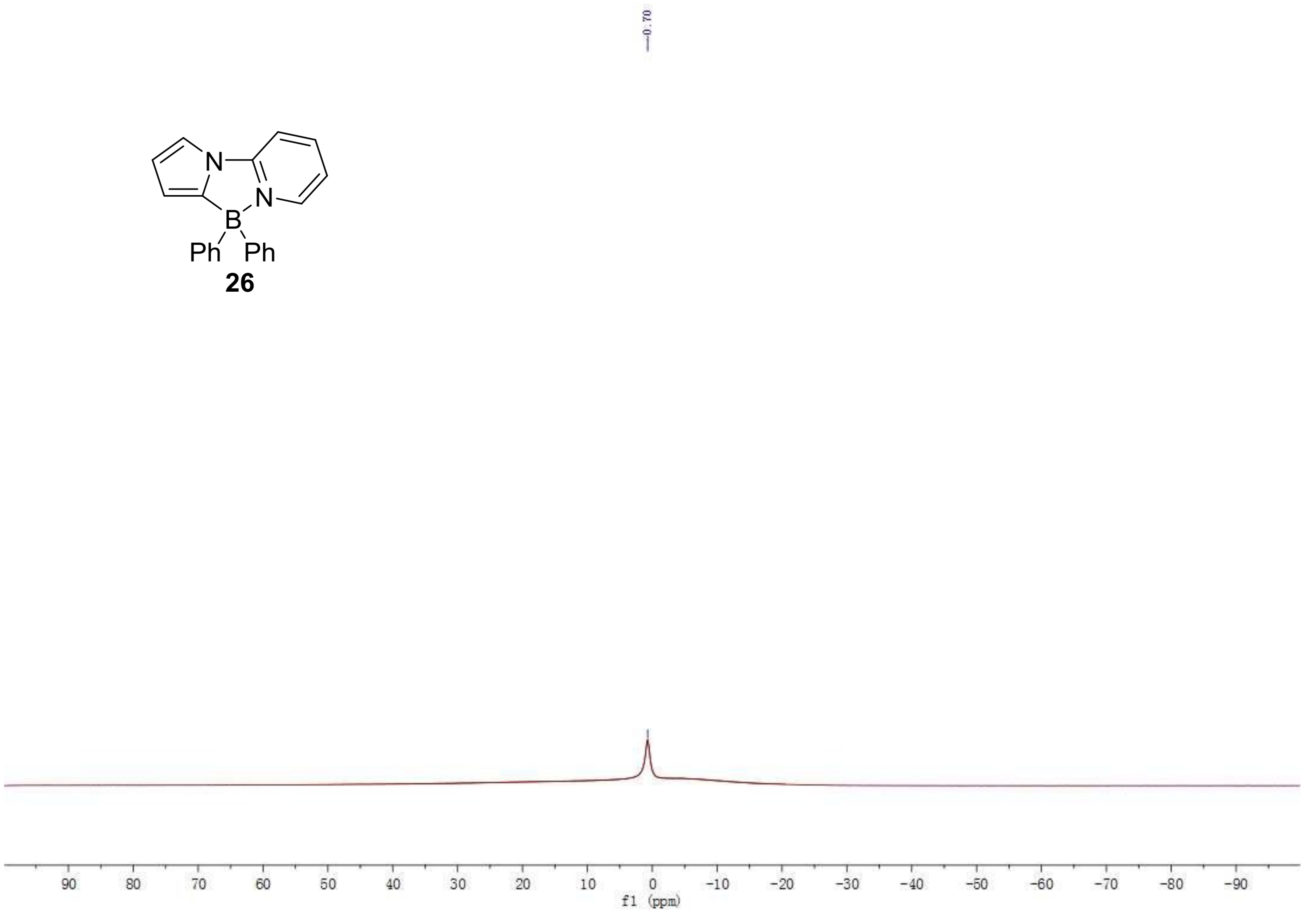

0.70
N
N
B
Ph
Ph
26
f1 (ppm)

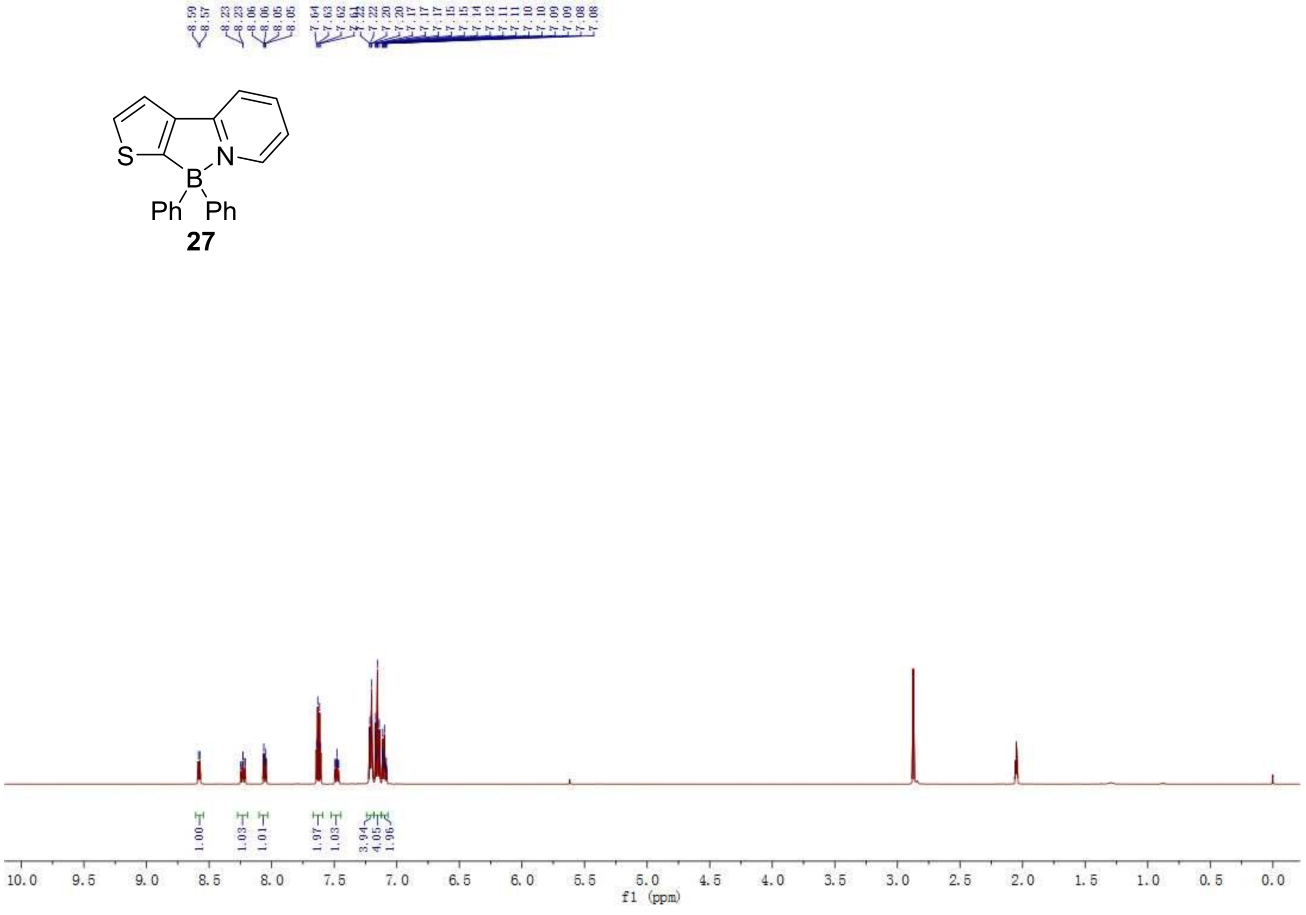

S
N
B
Ph
Ph
27
f1 (ppm)

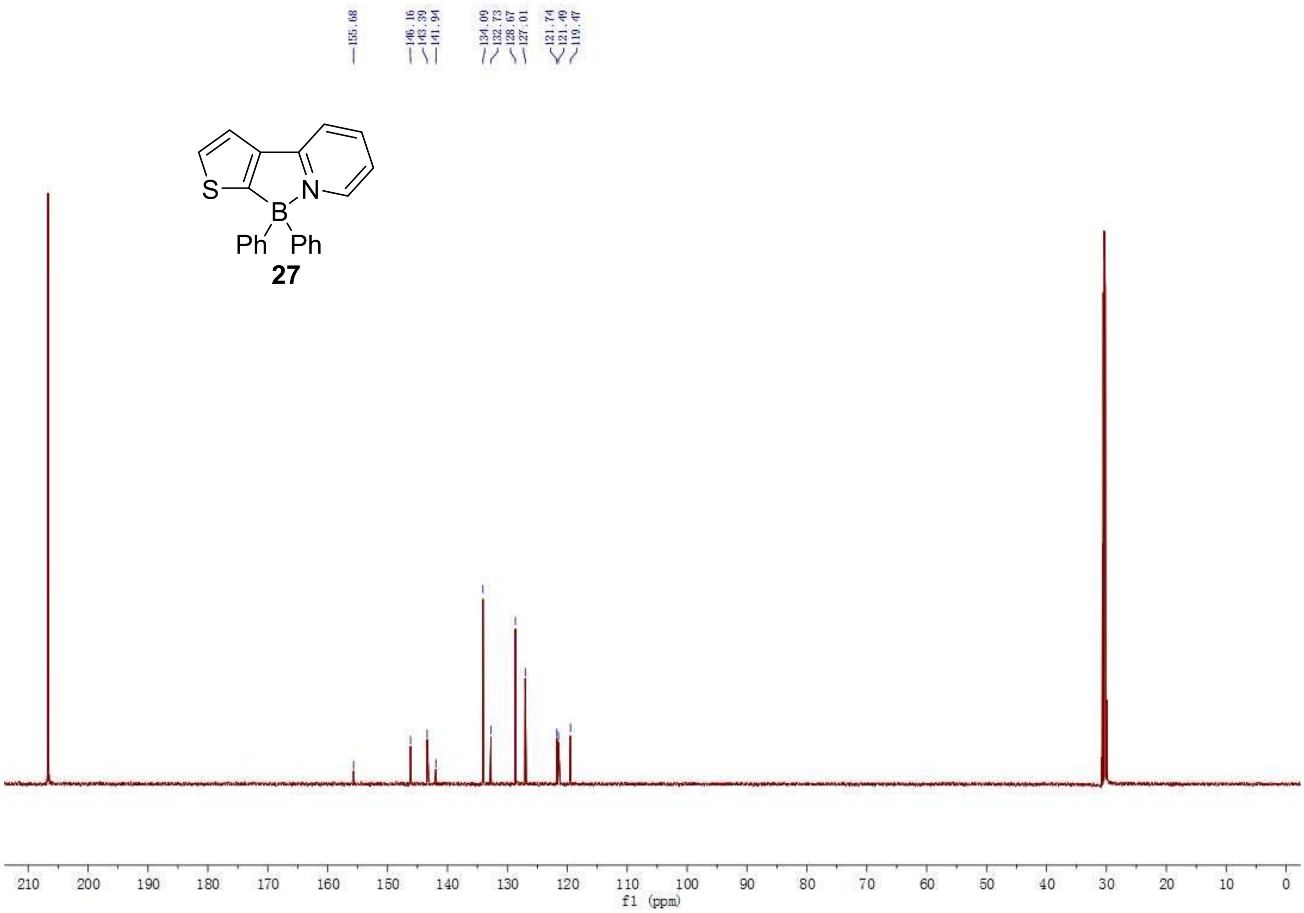
155.68
146.16
143.39
141.94
134.09
132.73
128.67
127.01
121.74
121.49
119.47
210 200 190 180 170 160 150 140 130 120 110 100 90 80 70 60 50 40 30 20 10 0
f1 (ppm)
S
B
N
Ph
Ph

27

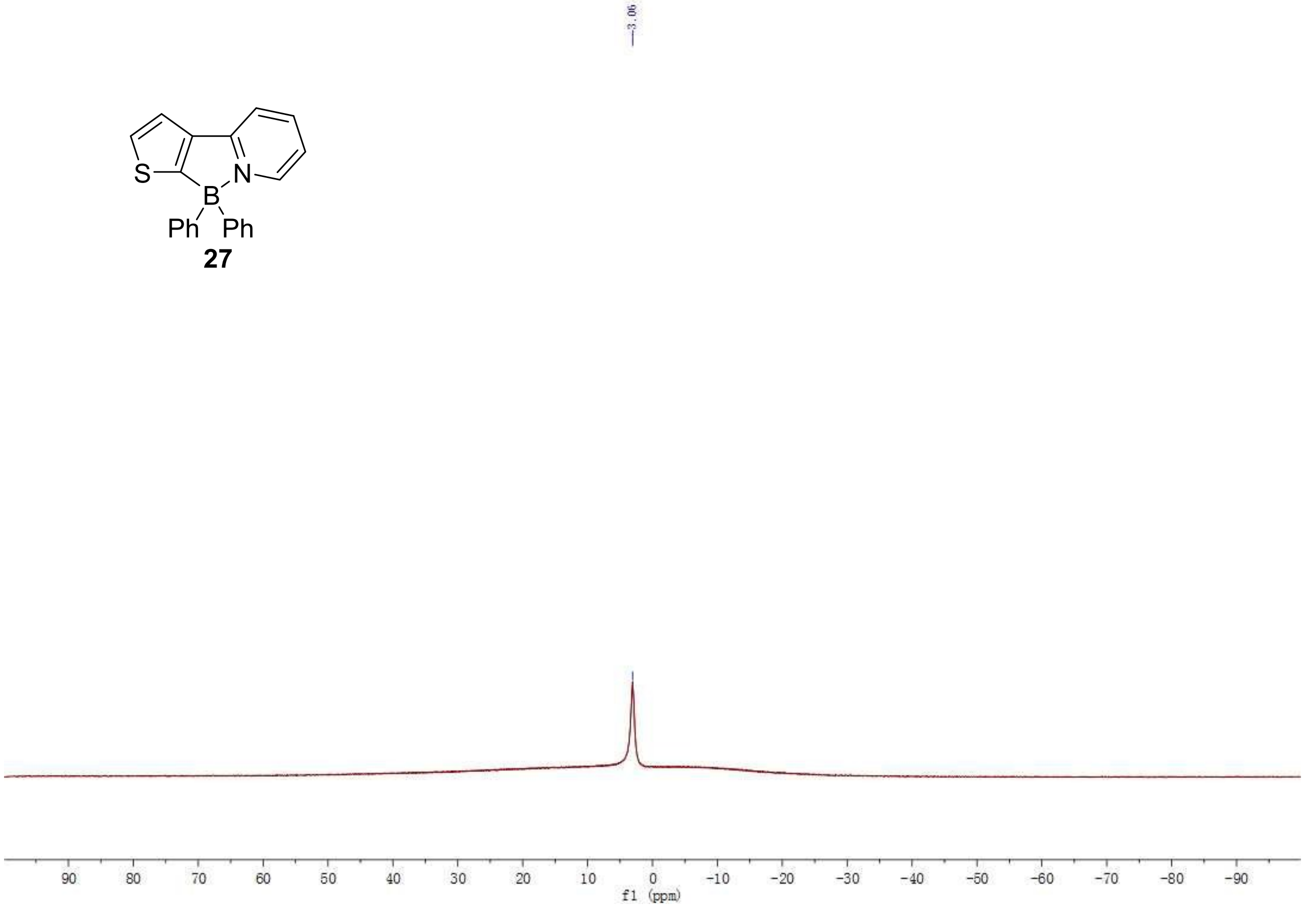
3.06
90 80 70 60 50 40 30 20 10 0 -10 -20 -30 -40 -50 -60 -70 -80 -90
f1 (ppm)
S
B
N
Ph
Ph

27

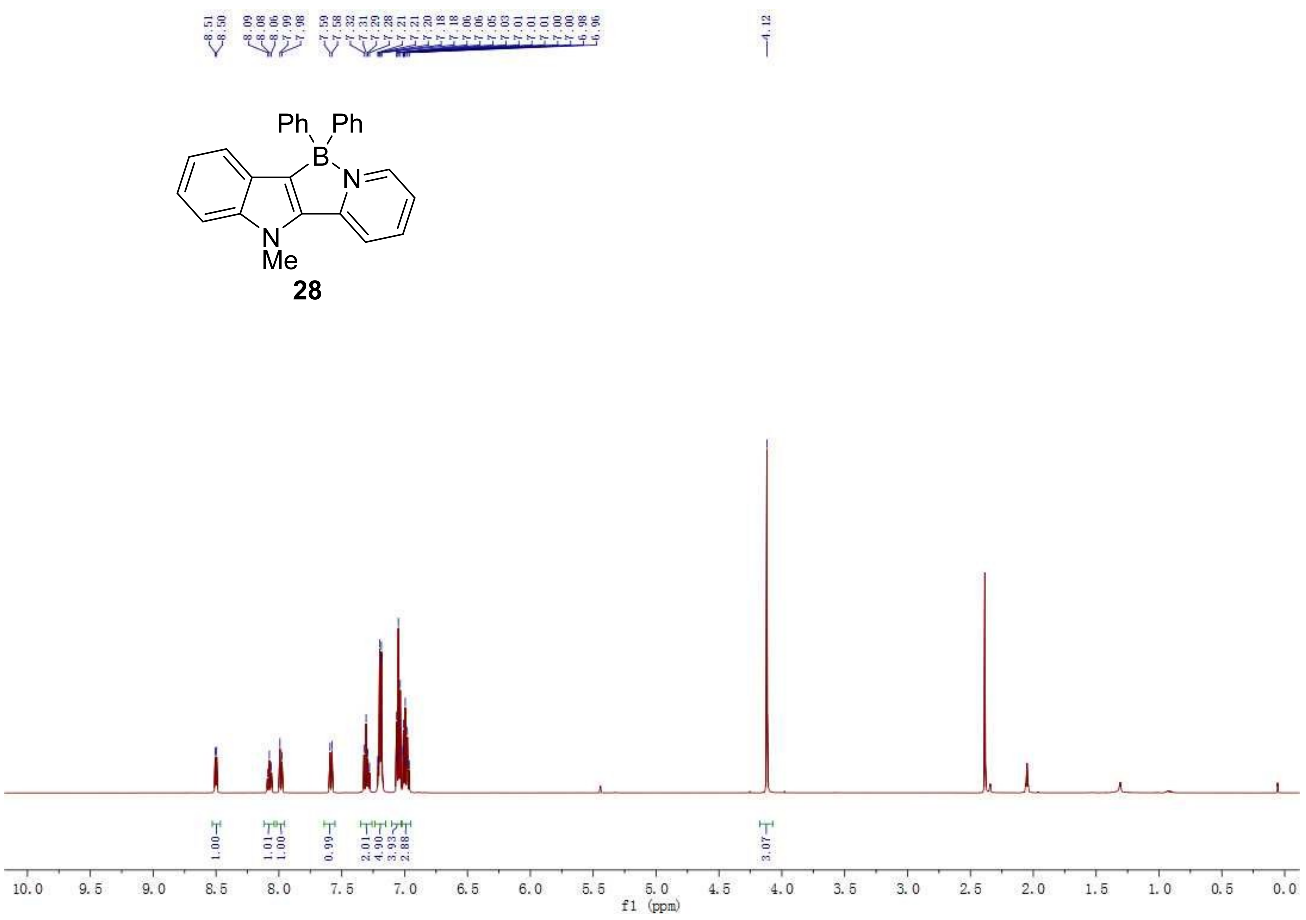
8.51
8.50
8.09
8.08
8.06
7.99
7.98
7.59
7.58
7.32
7.31
7.29
7.28
7.21
7.21
7.20
7.18
7.18
7.06
7.06
7.05
7.03
7.01
7.01
7.01
7.00
7.00
6.98
6.96
4.12
Ph
Ph
B
N
N
Me
28
1.00
1.01
1.00
0.99
2.01
4.90
3.93
2.88
3.07
f1 (ppm)

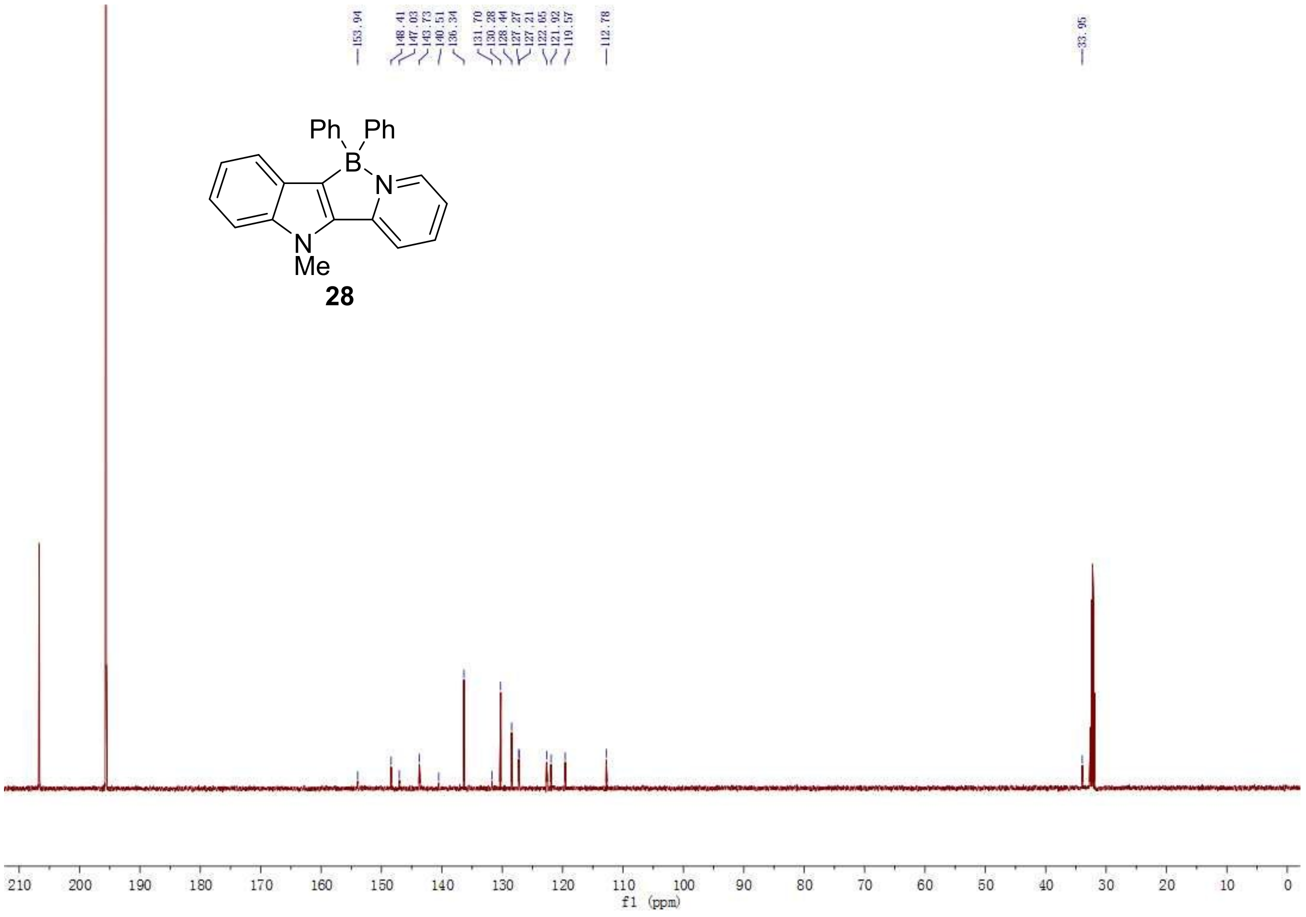
153.94
148.41
147.03
143.73
140.51
136.34
131.70
130.28
128.44
127.27
127.21
122.65
121.92
119.57
112.78
33.95
Ph
Ph
B
N
N
Me
28
f1 (ppm)

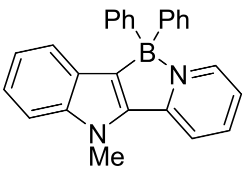

28

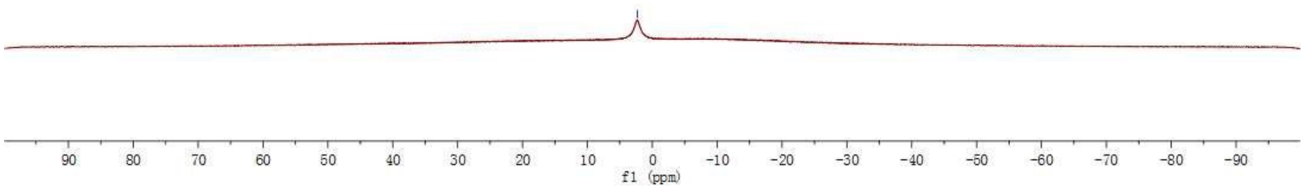

2.31
90 80 70 60 50 40 30 20 10 0 −10 −20 −30 −40 −50 −60 −70 −80 −90
f1 (ppm)

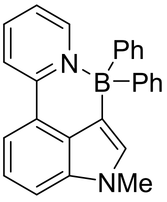

29

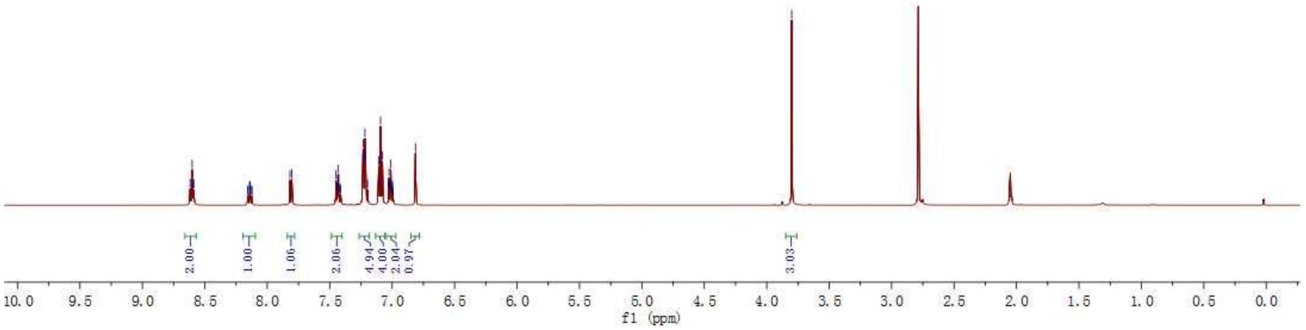

8.62 8.60 8.59 8.59 8.14 8.14 8.14 7.82 7.80 7.45 7.44 7.43 7.43 7.42 7.42 7.41 7.23 7.23 7.22 7.21 7.20 7.11 7.10 7.09 7.08 7.03 7.02 7.01 7.01 7.00 6.99 6.81
3.80
2.00 1.00 1.06 2.06 4.94 4.00 2.04 0.97
3.03
10.0 9.5 9.0 8.5 8.0 7.5 7.0 6.5 6.0 5.5 5.0 4.5 4.0 3.5 3.0 2.5 2.0 1.5 1.0 0.5 0.0
f1 (ppm)

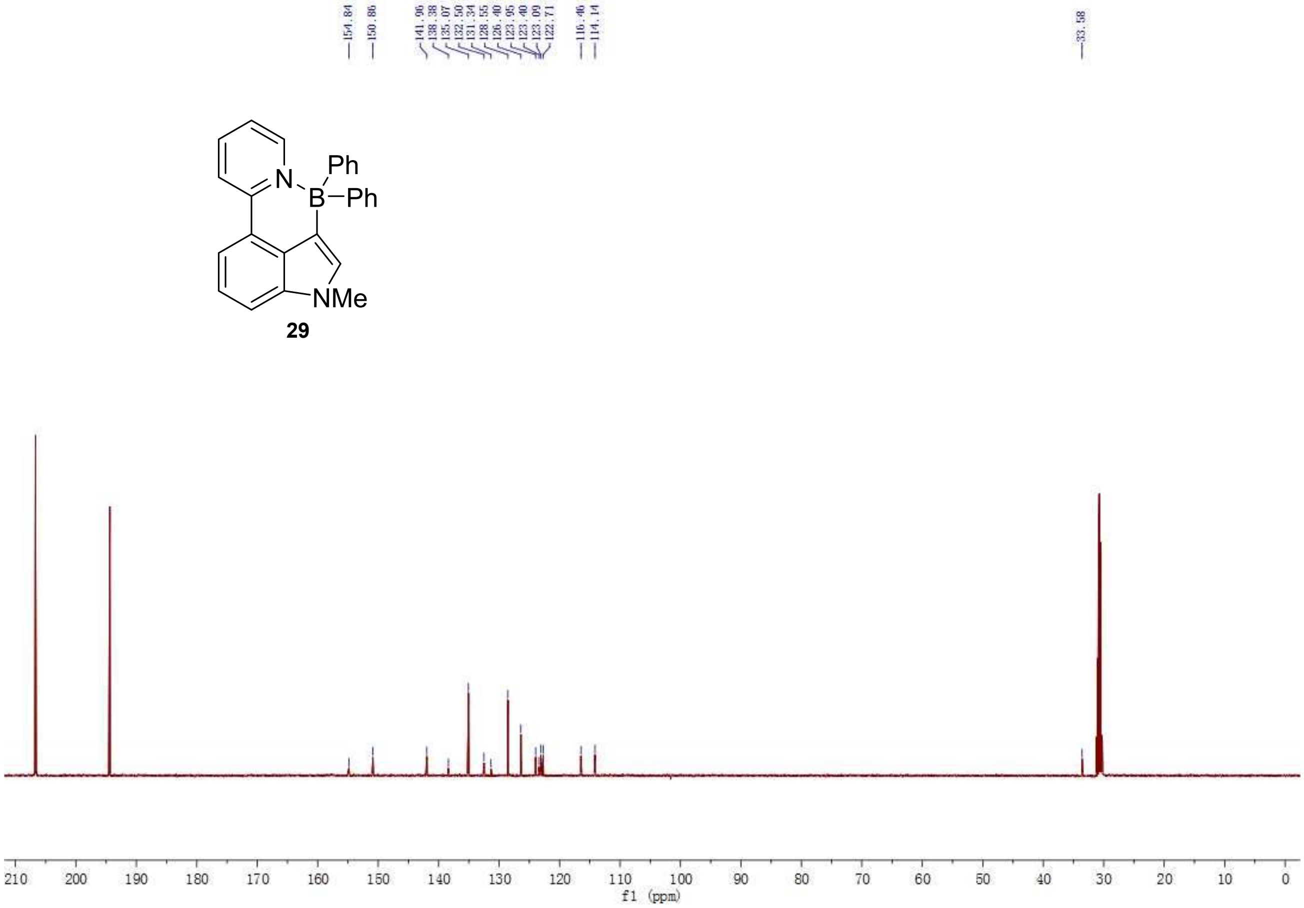
154.84
150.86
141.96
138.38
135.07
132.50
131.34
128.55
126.40
123.95
123.40
123.09
122.71
116.46
114.14
33.58
N
B
Ph
Ph
NMe
29
210 200 190 180 170 160 150 140 130 120 110 100 90 80 70 60 50 40 30 20 10 0
f1 (ppm)

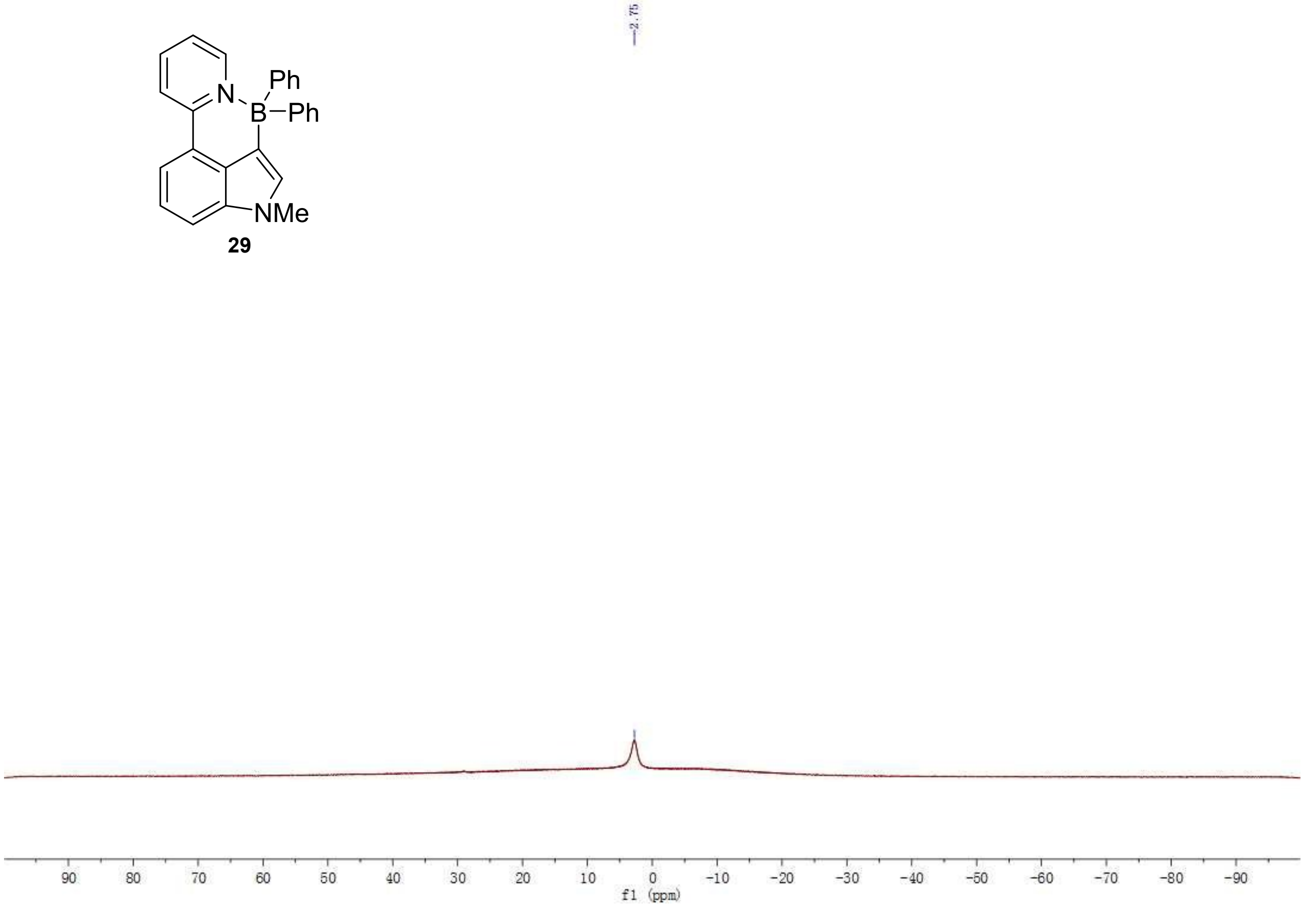
2.75
N
B
Ph
Ph
NMe
29
90 80 70 60 50 40 30 20 10 0 -10 -20 -30 -40 -50 -60 -70 -80 -90
f1 (ppm)

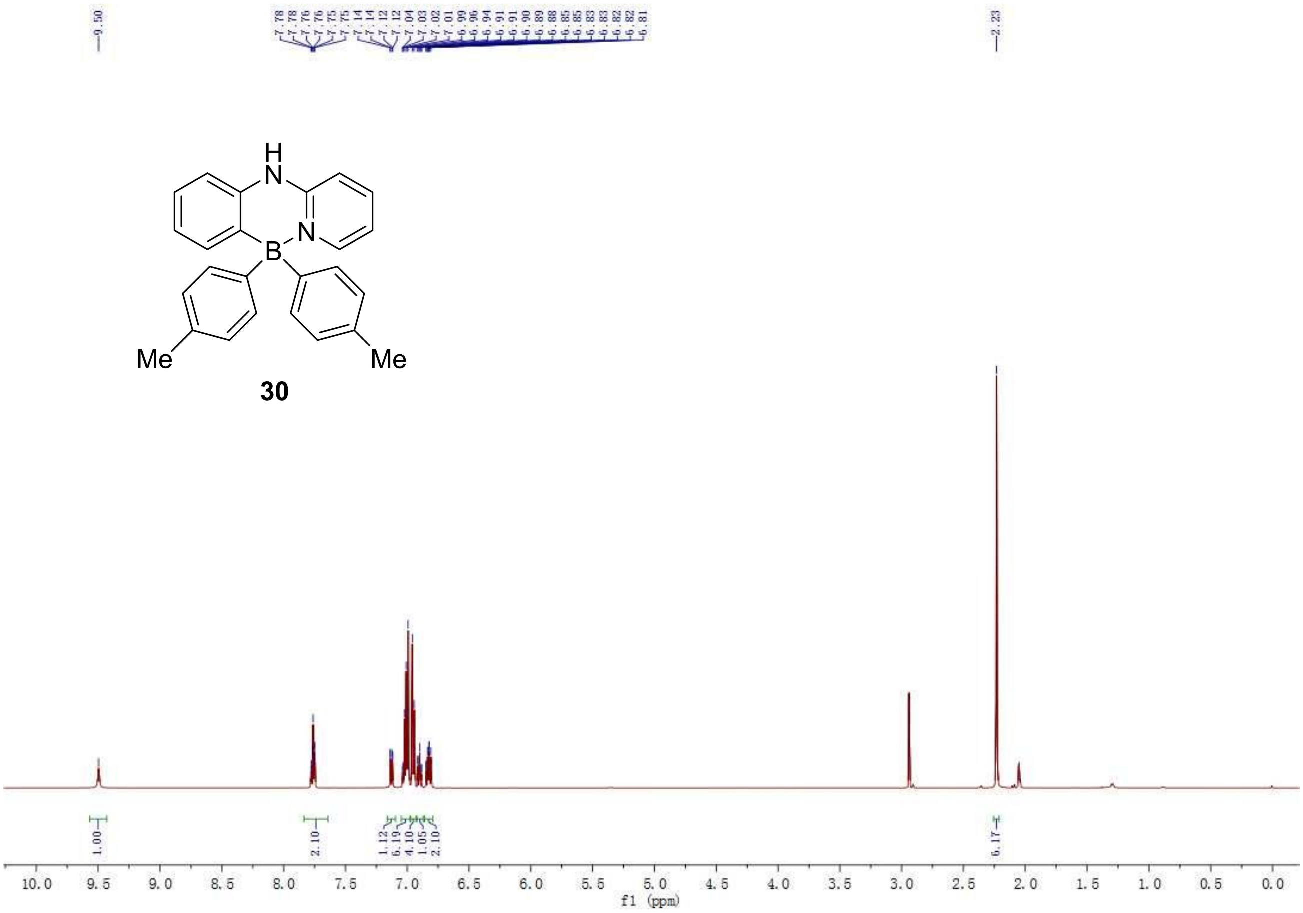

9.50
7.78
7.78
7.76
7.76
7.75
7.75
7.14
7.12
7.12
7.04
7.03
7.02
7.01
6.99
6.96
6.94
6.91
6.91
6.90
6.89
6.88
6.85
6.85
6.83
6.83
6.82
6.81
2.23
H
N
B
N
Me
Me
30
1.00
2.10
1.12
6.19
4.10
1.05
2.10
6.17
10.0 9.5 9.0 8.5 8.0 7.5 7.0 6.5 6.0 5.5 5.0 4.5 4.0 3.5 3.0 2.5 2.0 1.5 1.0 0.5 0.0
f1 (ppm)

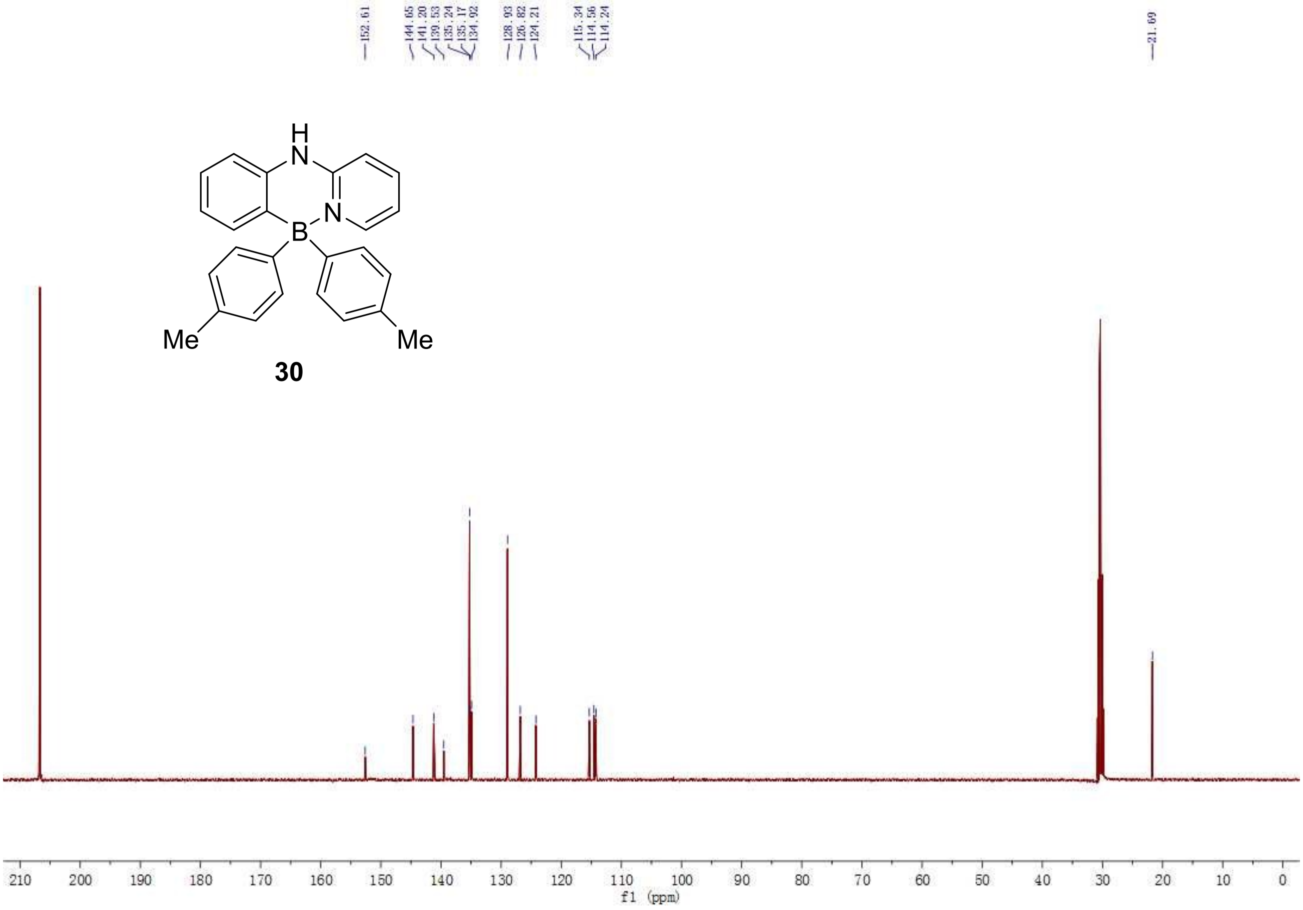

152.61
144.65
141.20
139.53
135.24
135.17
134.92
128.93
126.82
124.21
115.34
114.56
114.24
21.69
H
N
B
N
Me
Me
30
210 200 190 180 170 160 150 140 130 120 110 100 90 80 70 60 50 40 30 20 10 0
f1 (ppm)

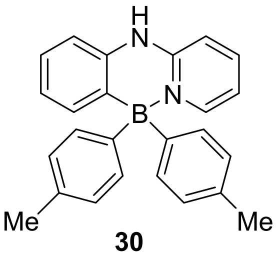

30

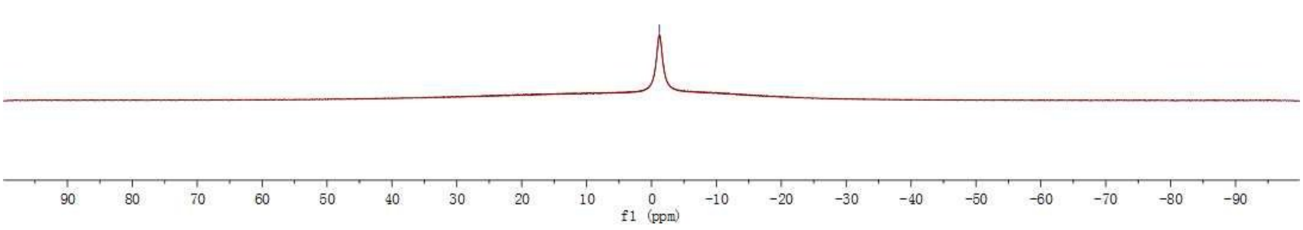

1.23
90 80 70 60 50 40 30 20 10 0 -10 -20 -30 -40 -50 -60 -70 -80 -90
f1 (ppm)

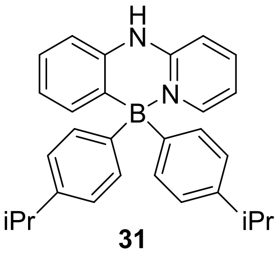

31

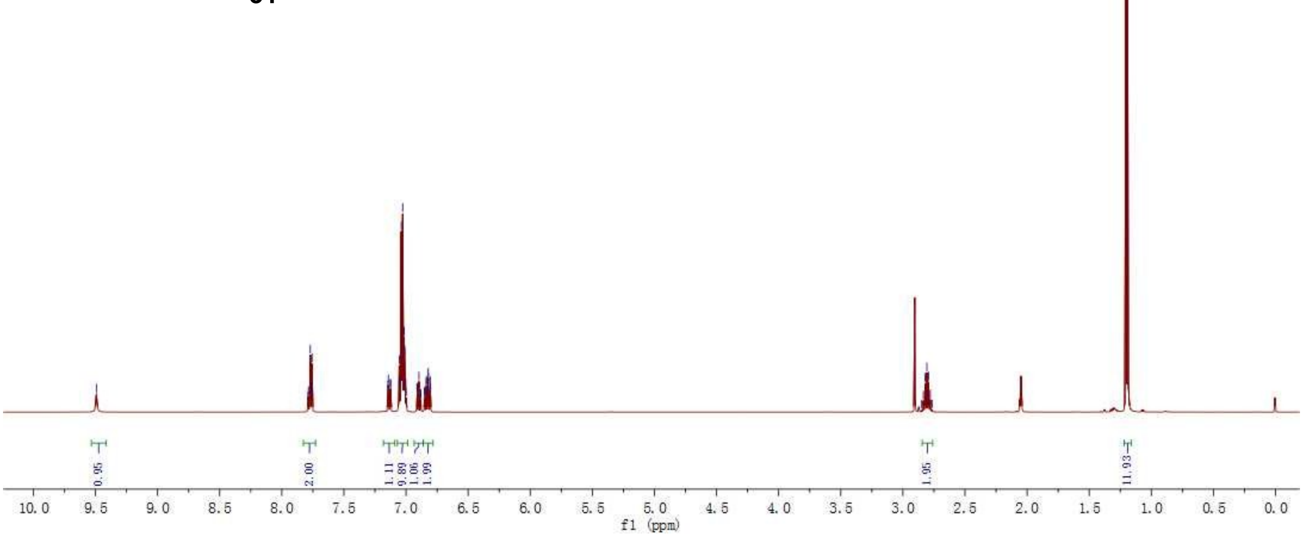

9.49
7.79 7.79 7.77 7.76 7.76 7.14 7.14 7.13 7.12 7.05 7.05 7.04 7.03 7.02 7.01 7.00 7.00 6.91 6.90 6.88 6.85 6.85 6.84 6.82 6.82 6.80 6.80
2.85 2.83 2.82 2.81 2.79 2.78 2.77
1.20 1.19
0.95
2.00
1.11 9.89 1.06 1.99
1.95
11.93
10.0 9.5 9.0 8.5 8.0 7.5 7.0 6.5 6.0 5.5 5.0 4.5 4.0 3.5 3.0 2.5 2.0 1.5 1.0 0.5 0.0
f1 (ppm)

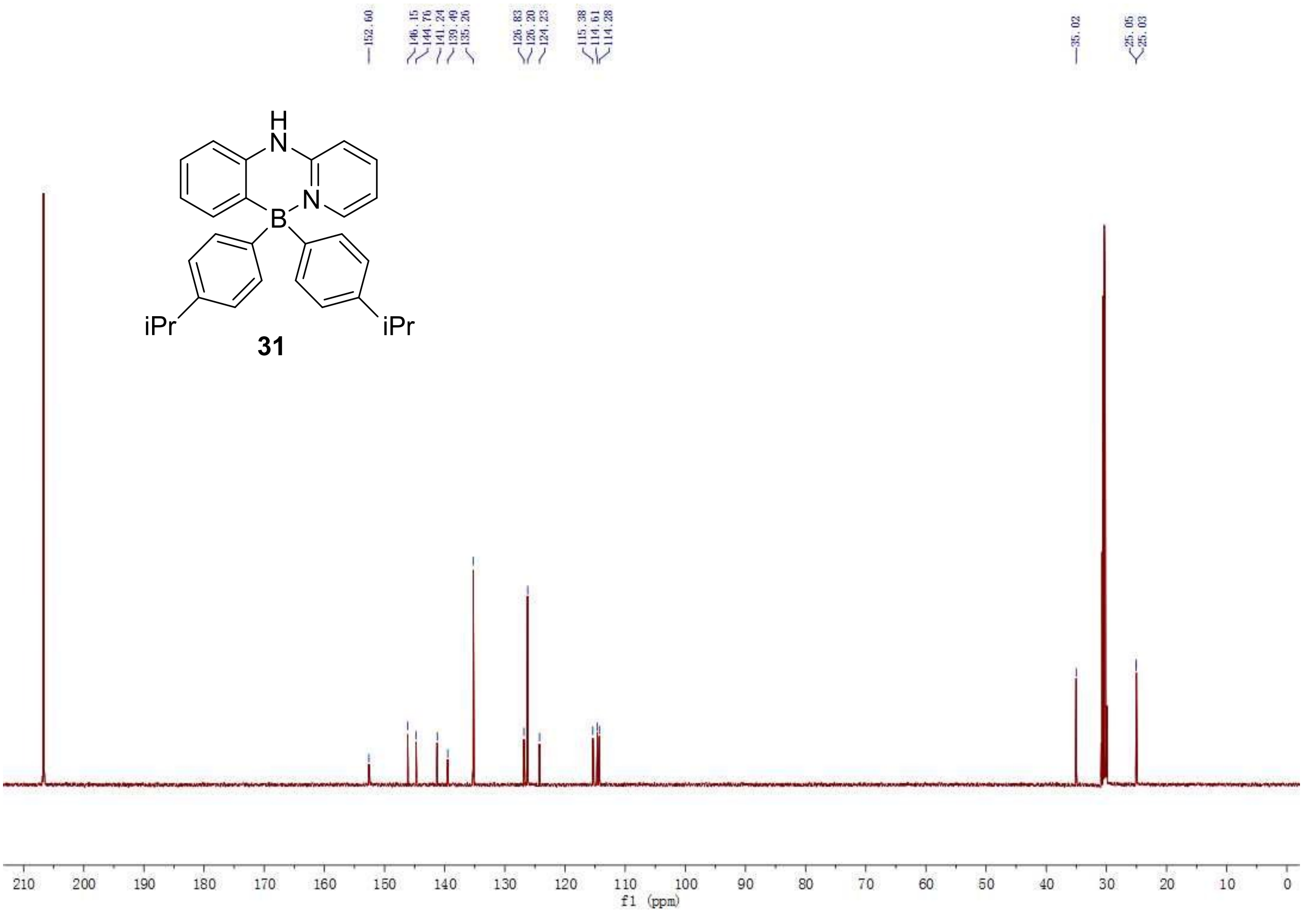
152.60
146.15
144.76
141.24
139.49
135.26
126.83
126.20
124.23
115.38
114.61
114.28
35.02
25.05
25.03
H
N
N
B
iPr
iPr
31
210 200 190 180 170 160 150 140 130 120 110 100 90 80 70 60 50 40 30 20 10 0
f1 (ppm)

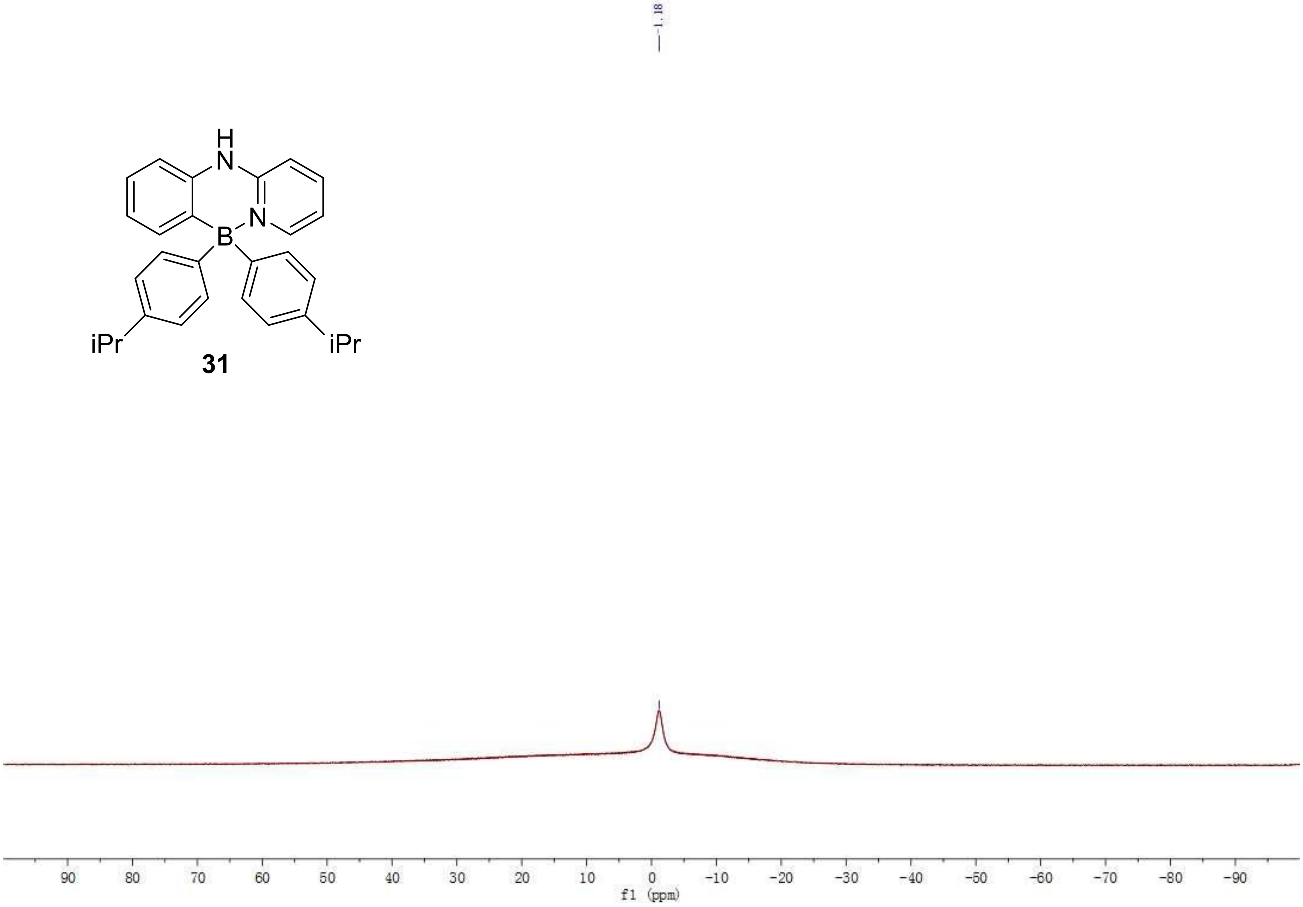
1.18
H
N
N
B
iPr
iPr
31
90 80 70 60 50 40 30 20 10 0 -10 -20 -30 -40 -50 -60 -70 -80 -90
f1 (ppm)

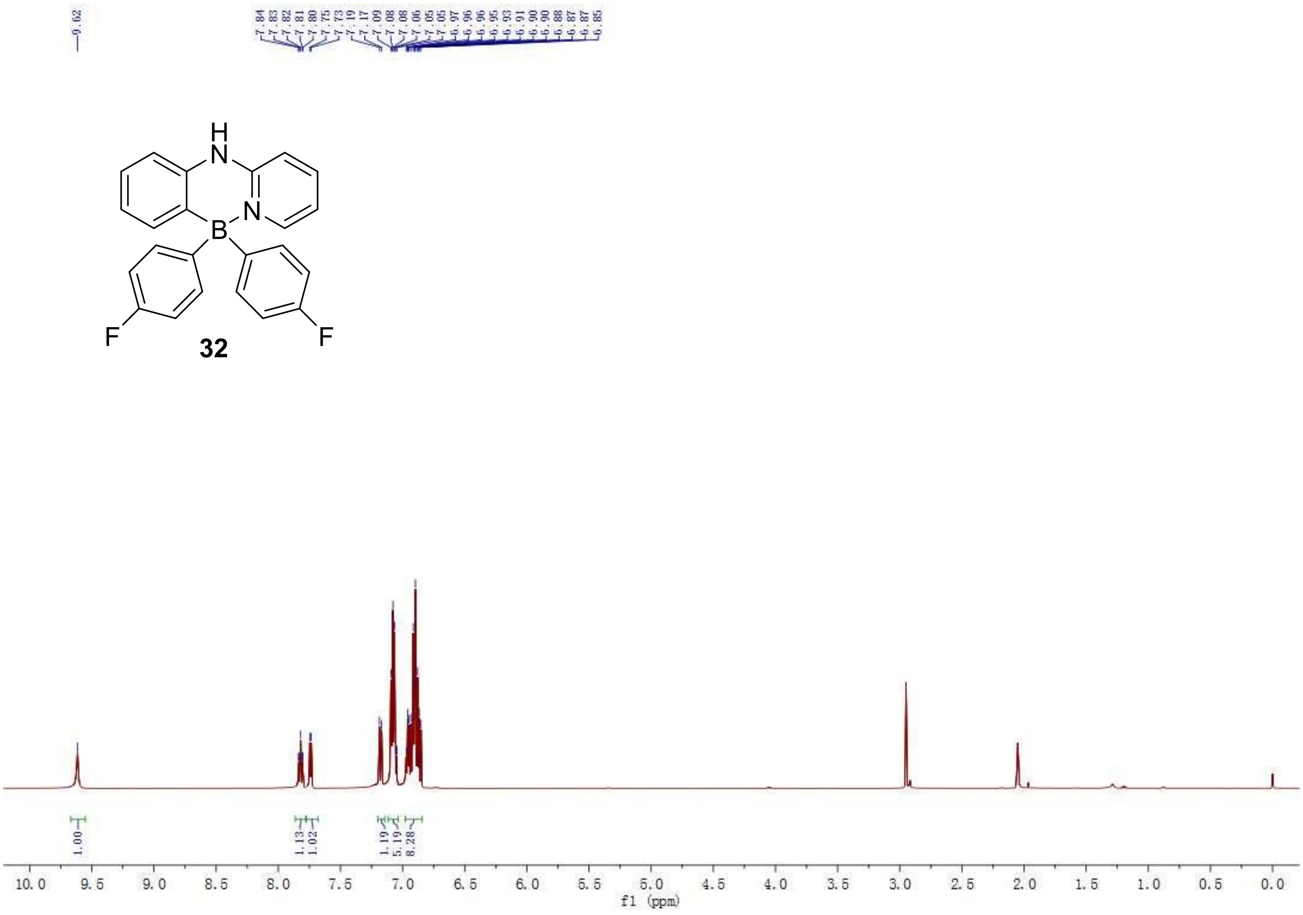
9.62
7.84 7.83 7.82 7.81 7.80 7.75 7.73 7.19 7.17 7.09 7.08 7.08 7.06 7.05 6.97 6.96 6.96 6.95 6.93 6.91 6.90 6.90 6.88 6.87 6.87 6.85
H
N
N
B
F
F
32
1.00
1.13 1.02
1.19 5.19 8.28
10.0 9.5 9.0 8.5 8.0 7.5 7.0 6.5 6.0 5.5 5.0 4.5 4.0 3.5 3.0 2.5 2.0 1.5 1.0 0.5 0.0
f1 (ppm)

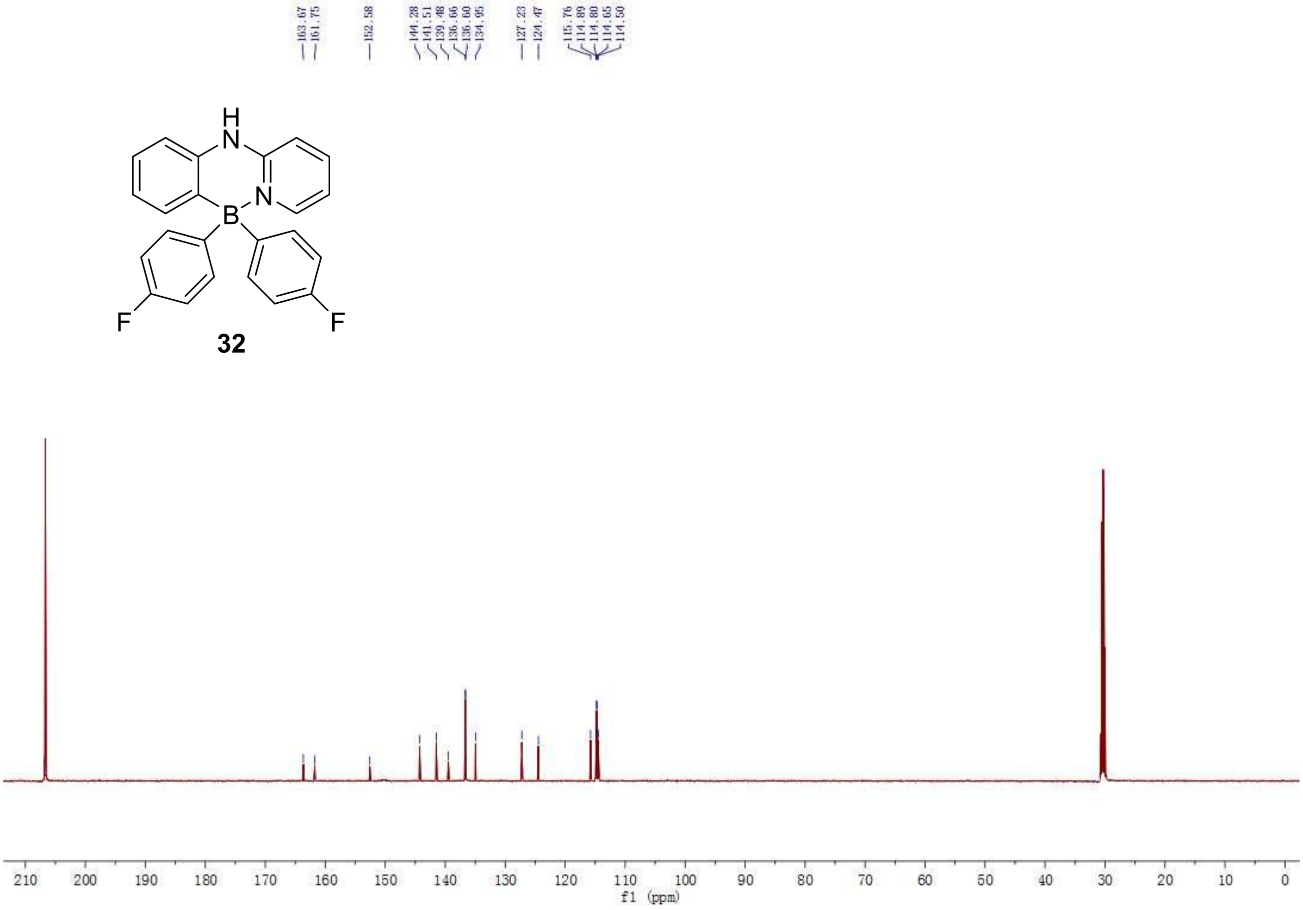
163.67 161.75
152.58
144.28 141.51 139.48 136.66 136.60 134.95
127.23 124.47
115.76 114.89 114.80 114.65 114.50
H
N
N
B
F
F
32
210 200 190 180 170 160 150 140 130 120 110 100 90 80 70 60 50 40 30 20 10 0
f1 (ppm)

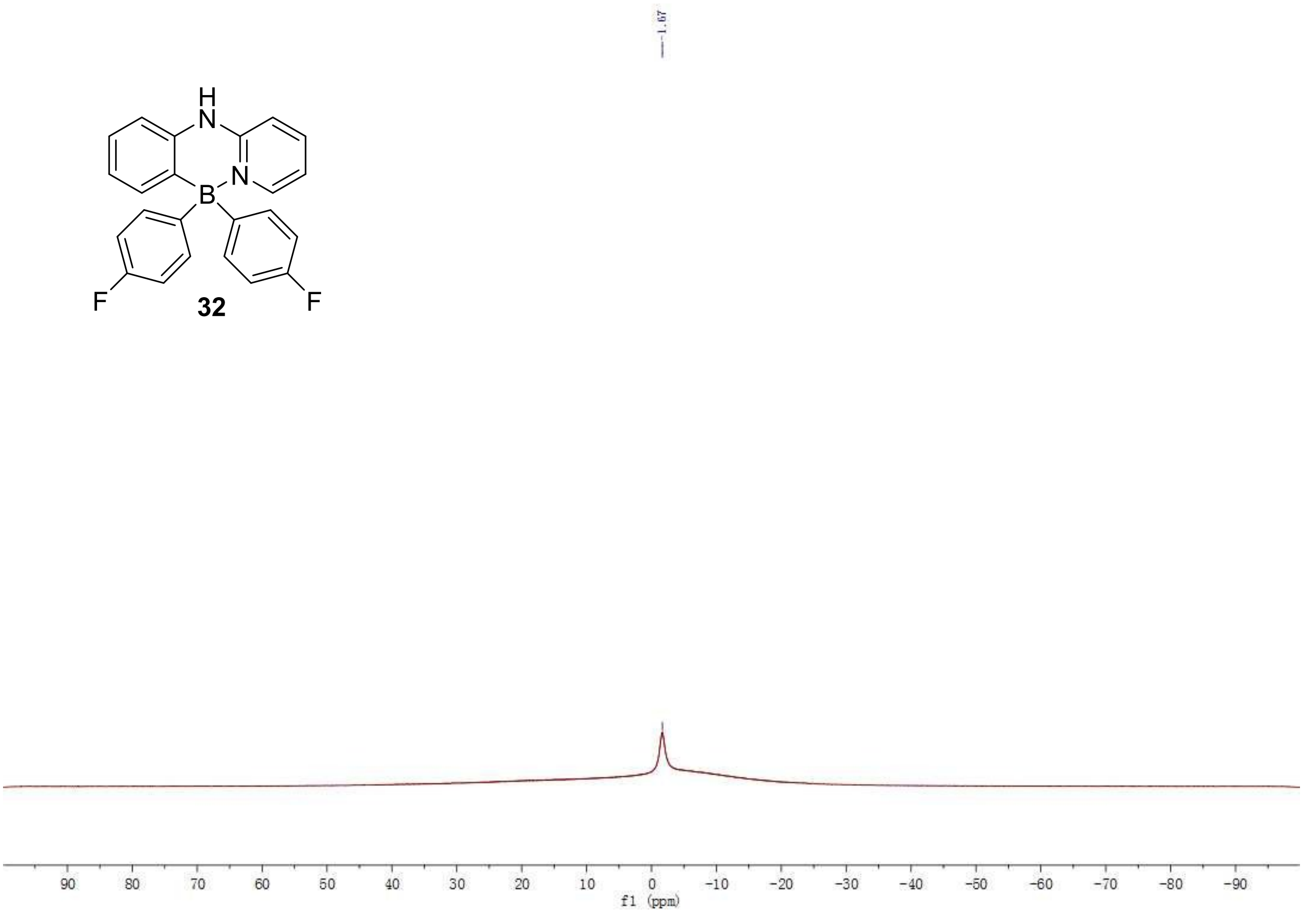

1.67
H
N
B
F
F
32
90 80 70 60 50 40 30 20 10 0 −10 −20 −30 −40 −50 −60 −70 −80 −90
f1 (ppm)

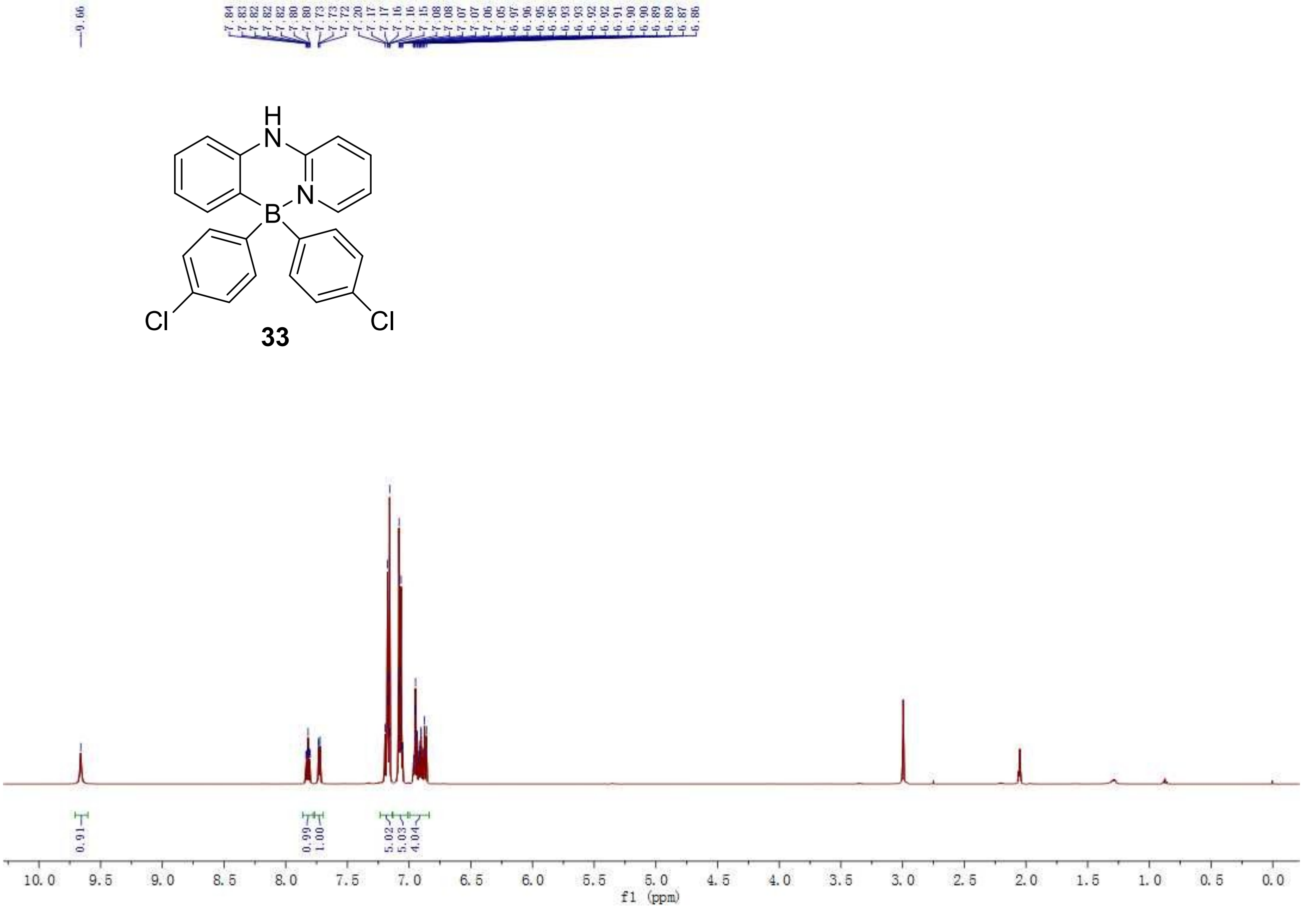

9.66
7.84 7.83 7.82 7.82 7.80 7.80 7.73 7.73 7.72 7.20 7.17 7.17 7.16 7.16 7.15 7.08 7.08 7.07 7.07 7.06 6.97 6.96 6.95 6.93 6.93 6.92 6.92 6.91 6.90 6.90 6.89 6.89 6.87 6.86
H
N
B
Cl
Cl
33
0.91
0.99
1.00
5.02
5.03
4.04
10.0 9.5 9.0 8.5 8.0 7.5 7.0 6.5 6.0 5.5 5.0 4.5 4.0 3.5 3.0 2.5 2.0 1.5 1.0 0.5 0.0
f1 (ppm)

152.51
144.17
141.57
139.38
136.69
134.83
131.97
128.20
127.34
124.54
115.83
114.94
114.54

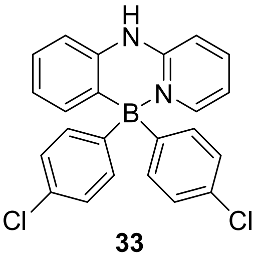

33

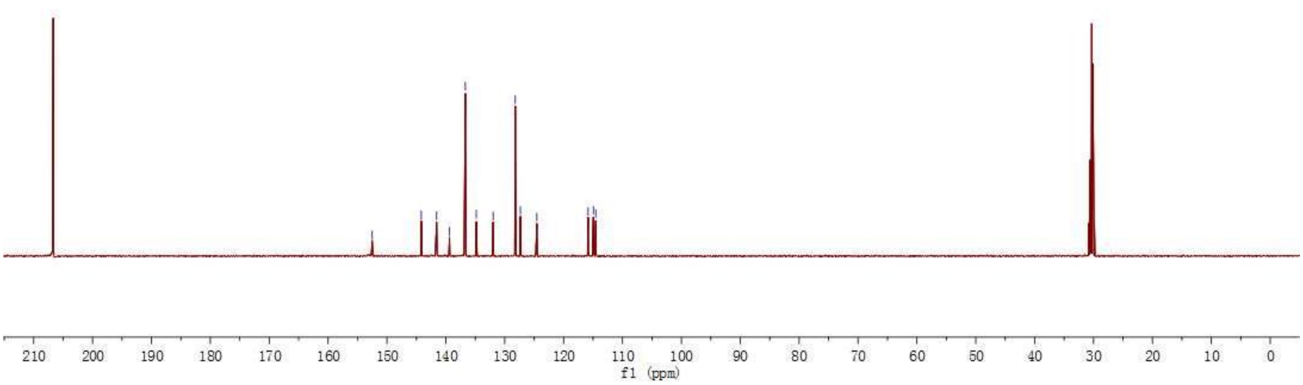

-1.74

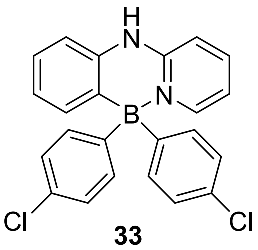

33

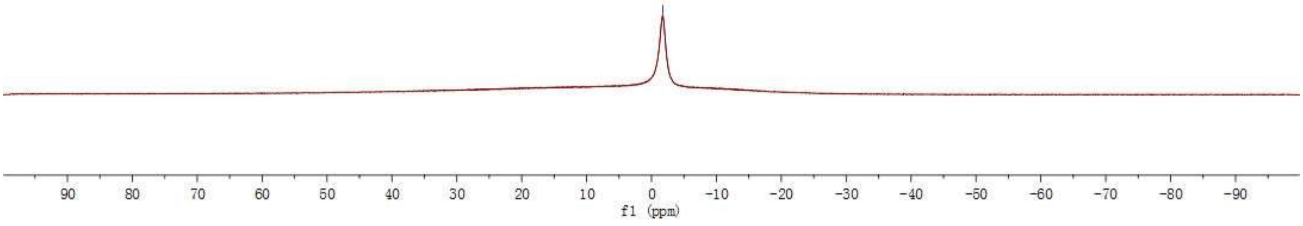

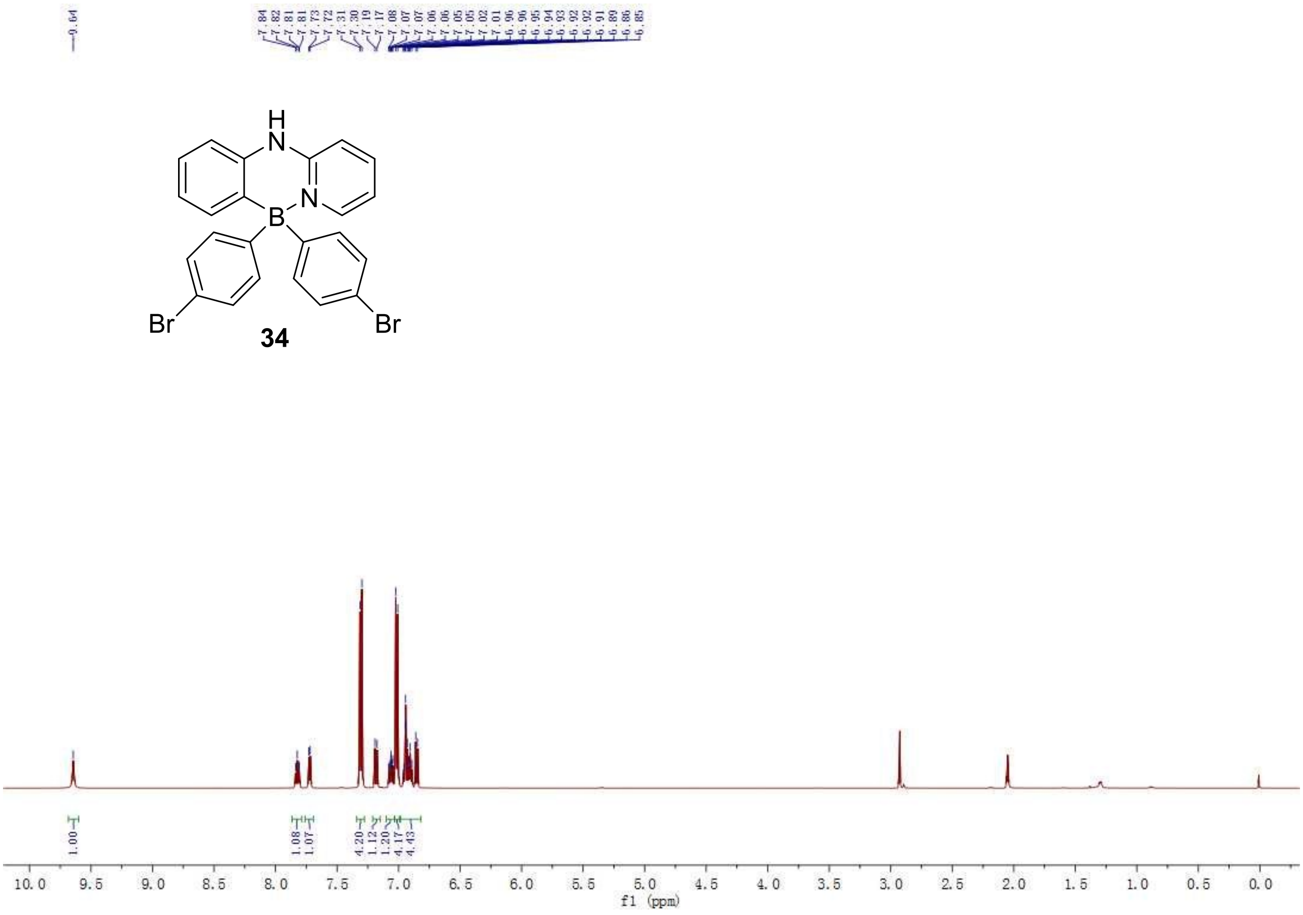

9.64
7.84 7.82 7.81 7.73 7.72 7.31 7.30 7.19 7.17 7.08 7.07 7.07 7.06 7.05 7.05 7.02 7.01 6.96 6.96 6.95 6.94 6.93 6.92 6.92 6.91 6.89 6.86 6.85
H
N
B
Br
Br
34
1.00
1.08 1.07
4.20 1.12 1.20 4.17 4.43
10.0 9.5 9.0 8.5 8.0 7.5 7.0 6.5 6.0 5.5 5.0 4.5 4.0 3.5 3.0 2.5 2.0 1.5 1.0 0.5 0.0
f1 (ppm)

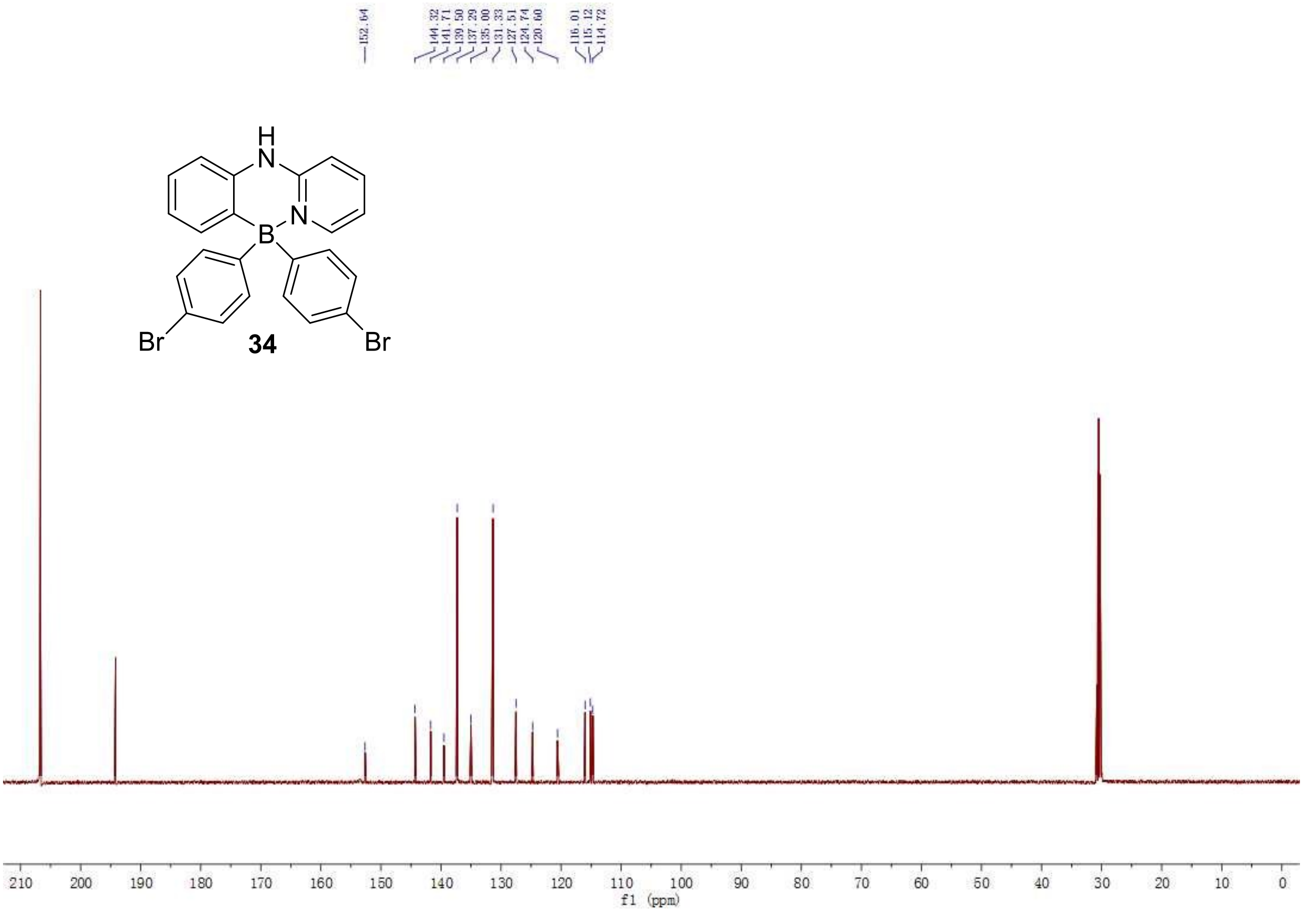

152.64
144.32 141.71 139.50 137.29 135.00 131.33 127.51 124.74 120.60
116.01 115.12 114.72
H
N
B
Br
Br
34
210 200 190 180 170 160 150 140 130 120 110 100 90 80 70 60 50 40 30 20 10 0
f1 (ppm)

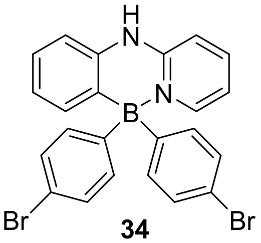

34

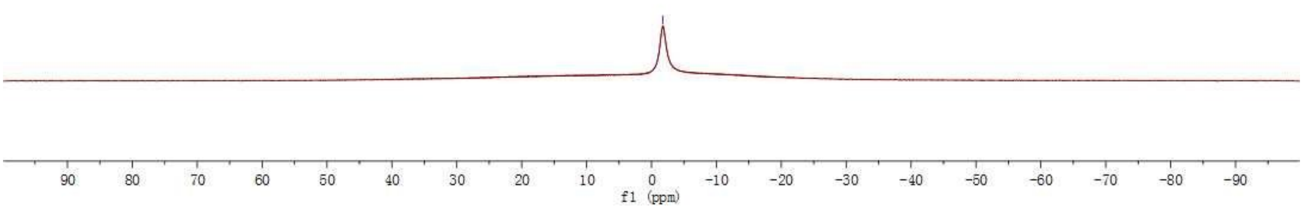

1.76
90 80 70 60 50 40 30 20 10 0 -10 -20 -30 -40 -50 -60 -70 -80 -90
f1 (ppm)

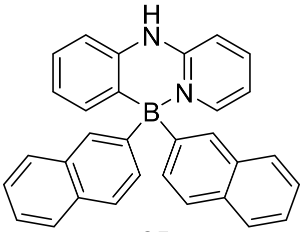

35

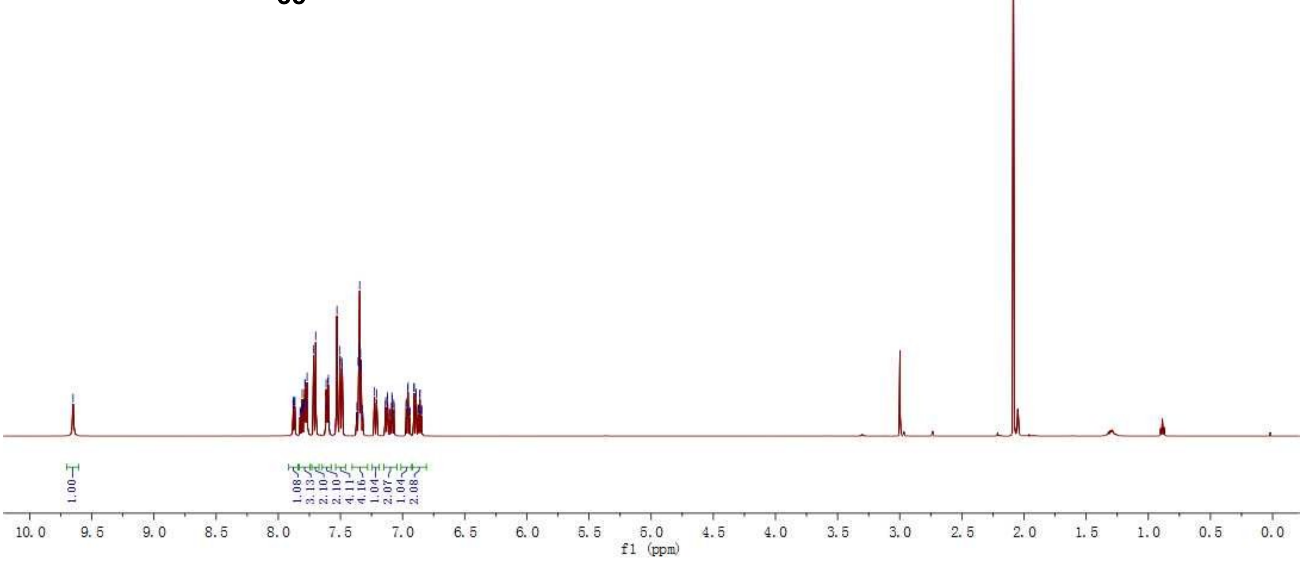

9.65 7.88 7.87 7.82 7.81 7.80 7.79 7.79 7.78 7.77 7.77 7.72 7.70 7.62 7.61 7.60 7.59 7.53 7.50 7.50 7.49 7.49 7.37 7.36 7.35 7.35 7.34 7.34 7.33 7.32 7.32 7.23 7.21 7.14 7.12 7.12 7.10 7.10 7.09 7.08 7.07 7.07 6.97 6.96 6.96 6.94 6.91 6.91 6.89 6.88 6.87 6.86 6.86 6.85
1.00
1.08 3.13 2.10 2.10 4.11 4.16 1.04 1.07 2.07 1.04 2.08
10.0 9.5 9.0 8.5 8.0 7.5 7.0 6.5 6.0 5.5 5.0 4.5 4.0 3.5 3.0 2.5 2.0 1.5 1.0 0.5 0.0
f1 (ppm)

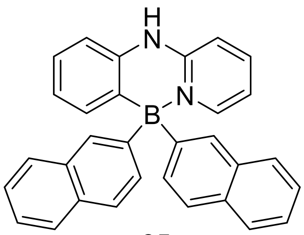

35

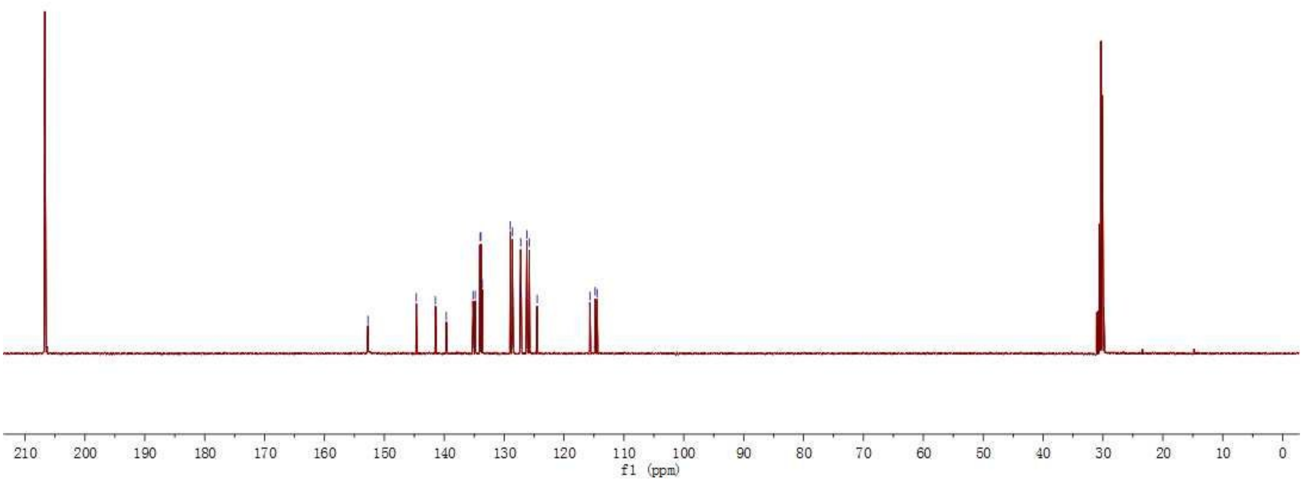
152.73
144.66
141.46
139.66
134.02
133.88
133.67
128.95
128.62
127.24
126.20
114.80
114.49
210 200 190 180 170 160 150 140 130 120 110 100 90 80 70 60 50 40 30 20 10 0
f1 (ppm)

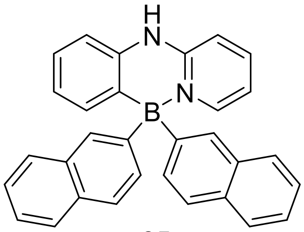

35

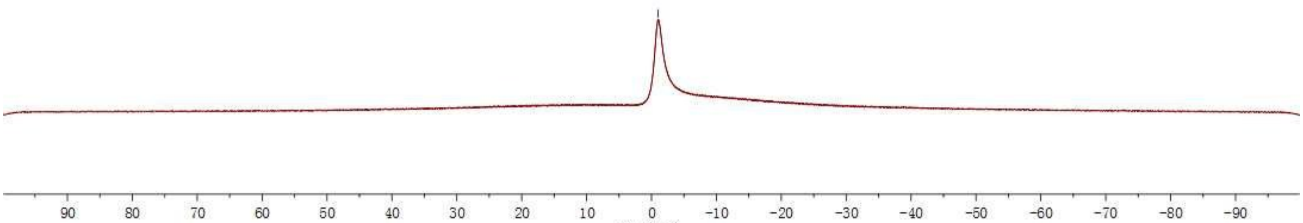
-1.03
90 80 70 60 50 40 30 20 10 0 -10 -20 -30 -40 -50 -60 -70 -80 -90
f1 (ppm)

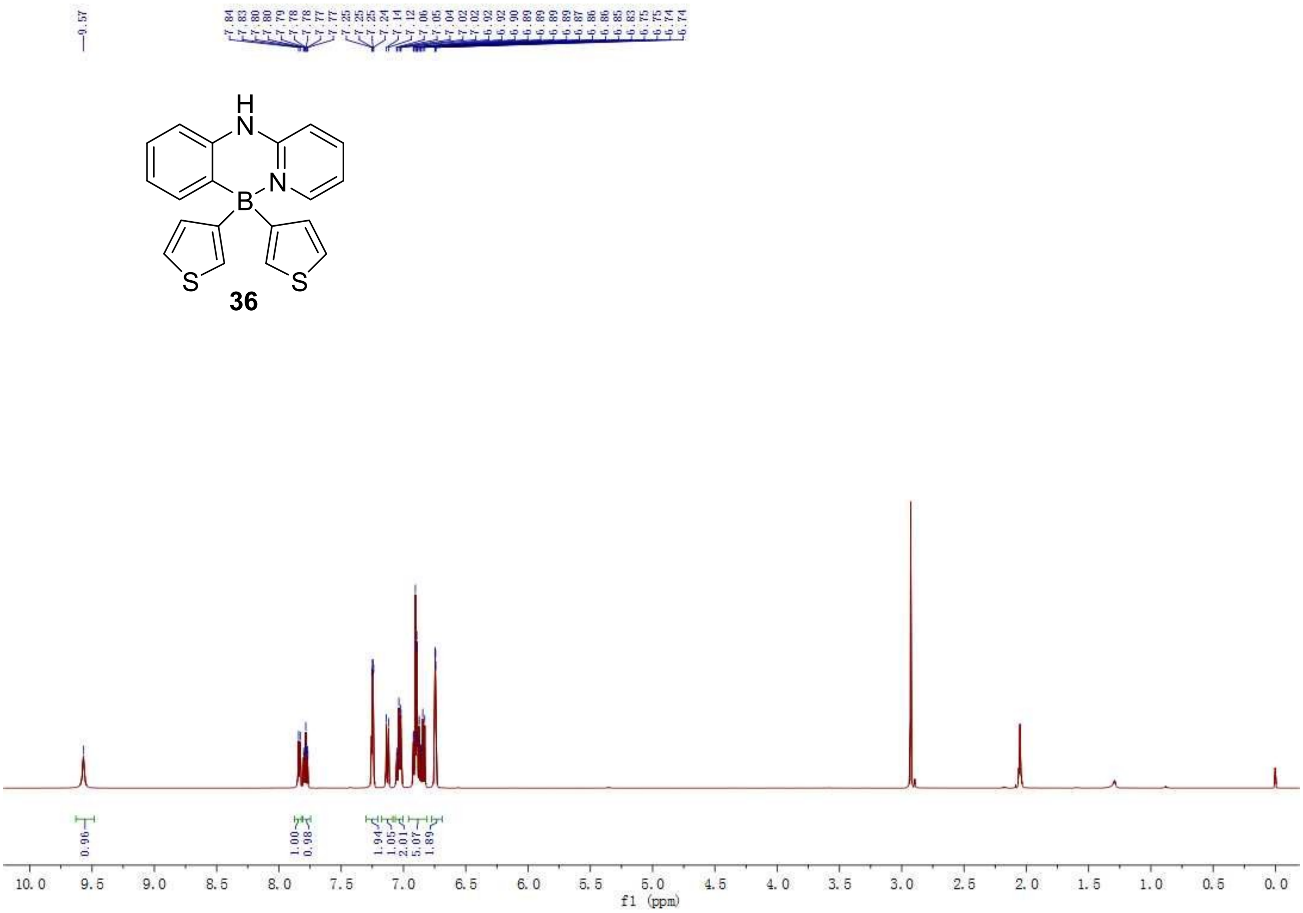
9.57
7.84 7.83 7.80 7.80 7.79 7.78 7.78 7.77 7.77 7.25 7.25 7.25 7.24 7.14 7.12 7.06 7.05 7.04 7.02 7.02 6.92 6.92 6.90 6.89 6.89 6.89 6.89 6.87 6.86 6.86 6.85 6.83 6.75 6.74 6.74
H
N
B
S
36
0.96
1.00
0.98
1.94
1.05
2.01
5.07
1.89
10.0 9.5 9.0 8.5 8.0 7.5 7.0 6.5 6.0 5.5 5.0 4.5 4.0 3.5 3.0 2.5 2.0 1.5 1.0 0.5 0.0
f1 (ppm)

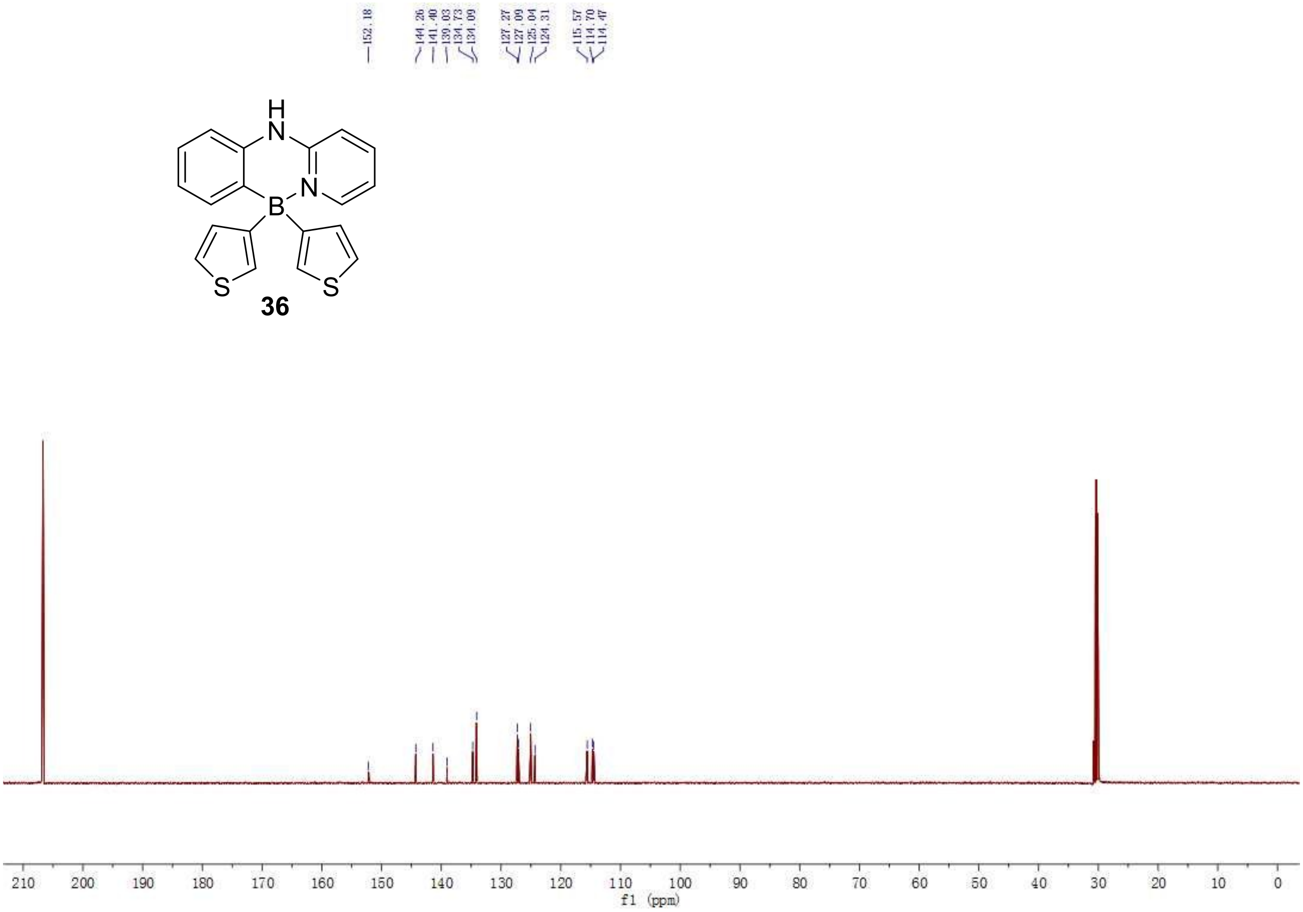
152.18
144.26 141.40 139.03 134.73 134.09
127.27 127.09 125.04 124.31
115.57 114.70 114.47
H
N
B
S
36
210 200 190 180 170 160 150 140 130 120 110 100 90 80 70 60 50 40 30 20 10 0
f1 (ppm)

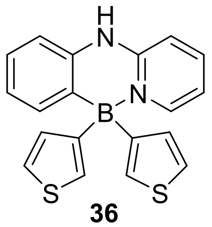
H
N
N
B
S
S

36

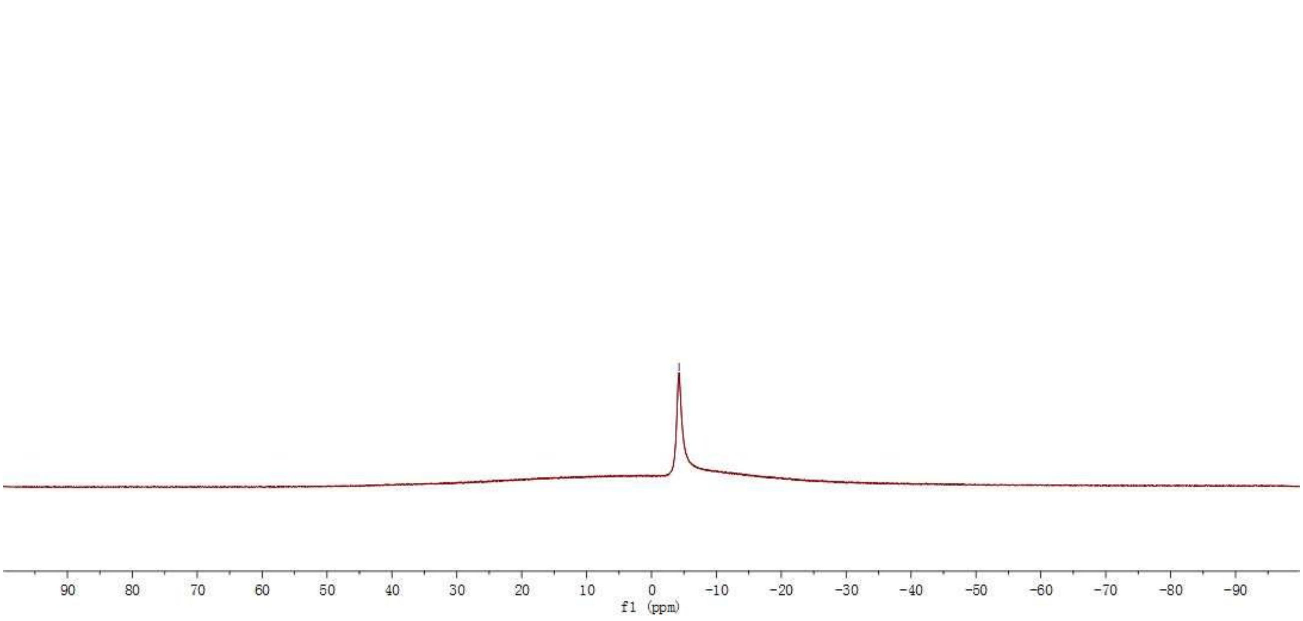
-4.24
90 80 70 60 50 40 30 20 10 0 -10 -20 -30 -40 -50 -60 -70 -80 -90
f1 (ppm)

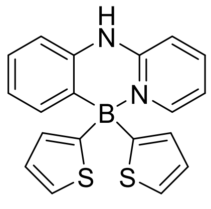
H
N
N
B
S
S

37

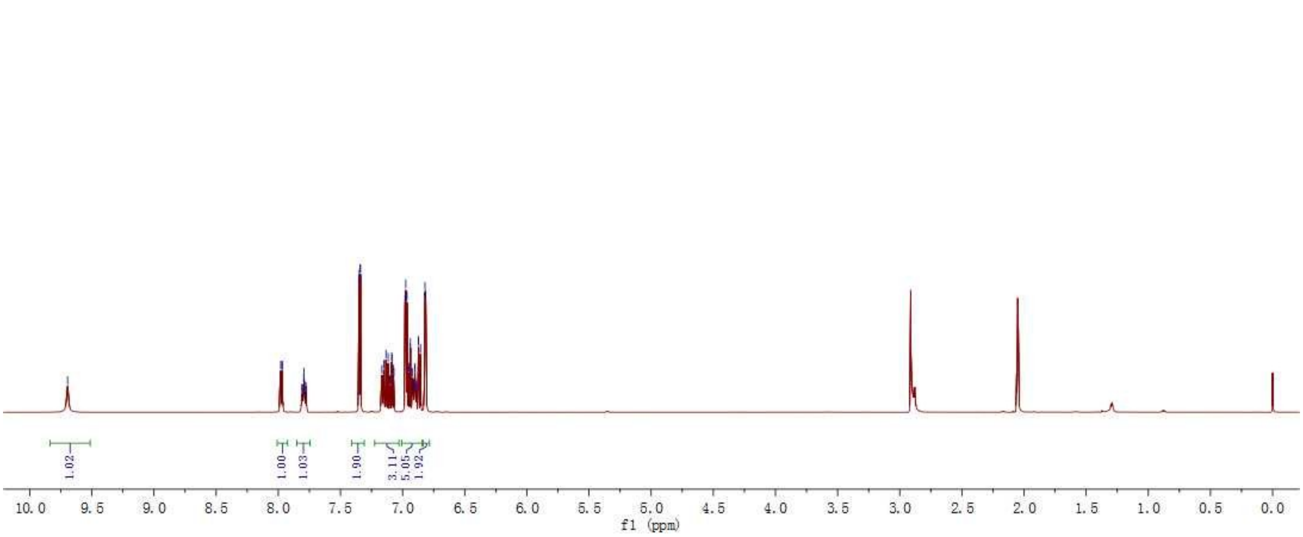
9.70
7.98 7.97 7.97 7.81 7.81 7.80 7.79 7.79 7.78 7.78 7.78 7.35 7.35 7.34 7.34 7.17 7.15 7.13 7.12 7.10 7.10 7.09 7.09 7.07 7.07 6.98 6.97 6.97 6.96 6.95 6.95 6.94 6.94 6.92 6.92 6.91 6.91 6.90 6.90 6.89 6.89 6.87 6.87 6.86 6.82 6.81
1.02
1.00
1.03
1.90
3.11
5.05
1.92
10.0 9.5 9.0 8.5 8.0 7.5 7.0 6.5 6.0 5.5 5.0 4.5 4.0 3.5 3.0 2.5 2.0 1.5 1.0 0.5 0.0
f1 (ppm)

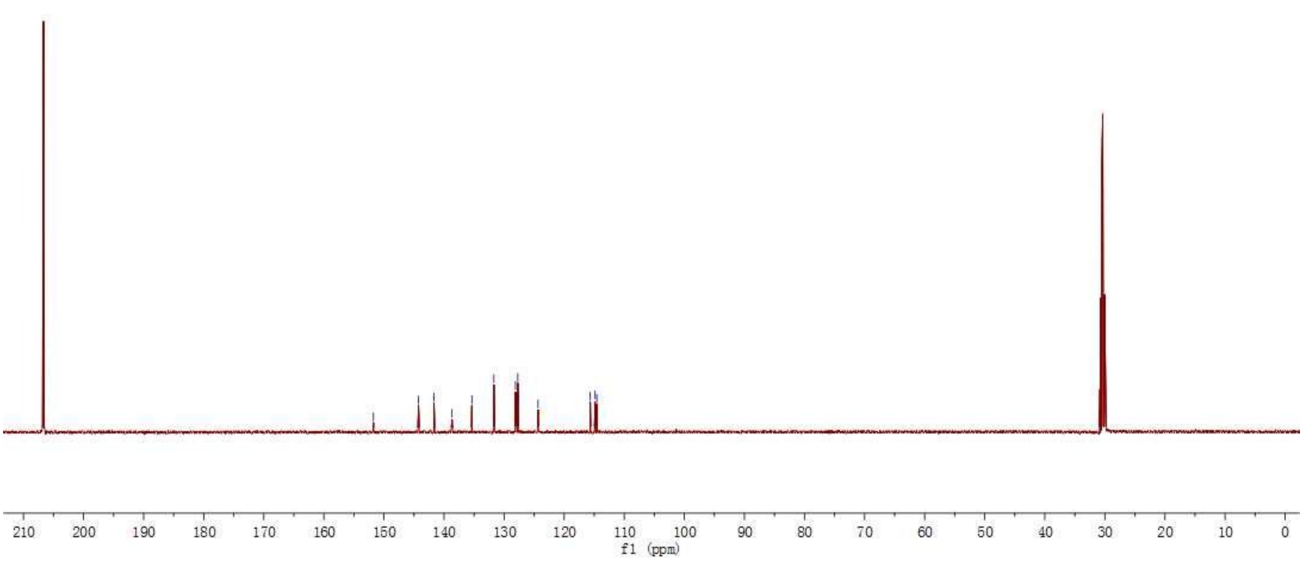

151.75
144.24
141.67
138.67
135.37
131.70
128.10
127.72
127.66
124.35
115.66
114.85
114.57
f1 (ppm)

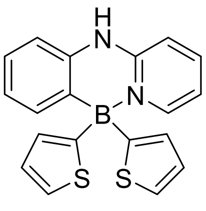

37

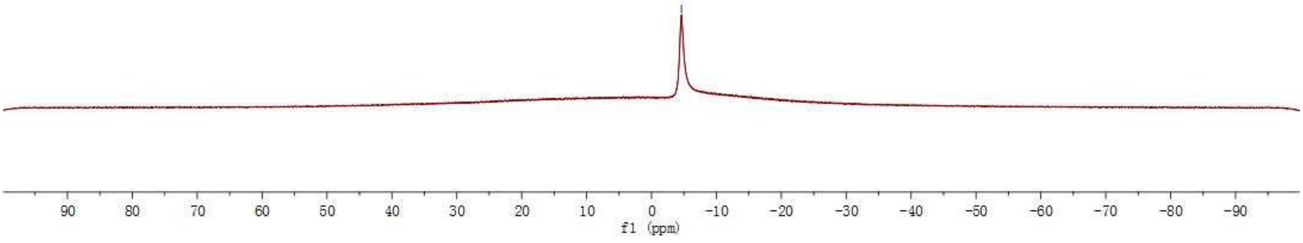

-4.61
f1 (ppm)

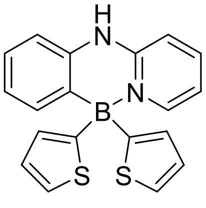

37

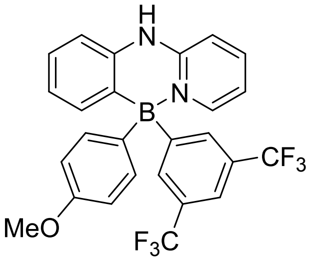
H
N
N
B
CF3
MeO
F3C

38

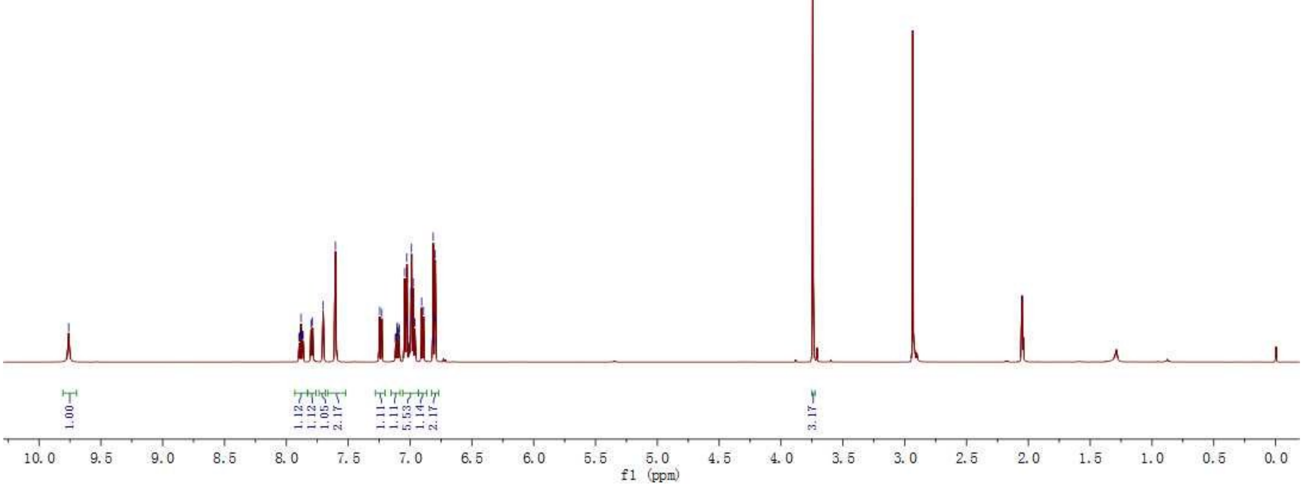
9.76
7.90 7.90 7.88 7.88 7.88 7.87 7.86 7.80 7.79 7.70 7.60 7.25 7.23 7.12 7.11 7.11 7.10 7.10 7.10 7.09 7.09 7.04 7.03 7.03 7.01 7.00 6.99 6.99 6.97 6.96 6.91 6.89 6.82 6.81 6.81 6.80 6.80 6.79
3.74
1.00
1.12 1.12 1.05 2.17
1.11 1.11 5.53 1.14 2.17
3.17
10.0 9.5 9.0 8.5 8.0 7.5 7.0 6.5 6.0 5.5 5.0 4.5 4.0 3.5 3.0 2.5 2.0 1.5 1.0 0.5 0.0
f1 (ppm)

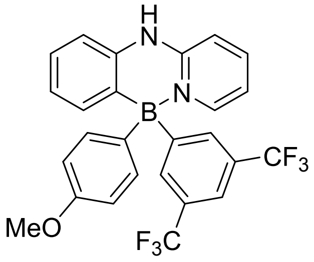
H
N
N
B
CF3
MeO
F3C

38

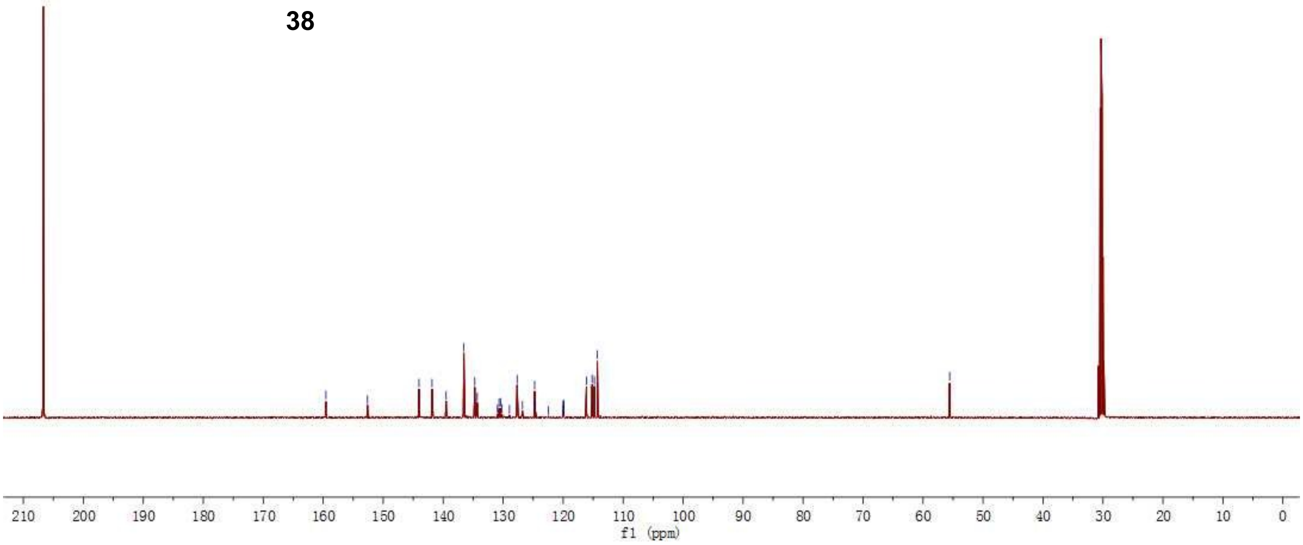
159.55
152.62 144.04 141.86 139.51 136.55 134.73 134.33 130.94 130.69 130.44 130.19 128.94 127.67 126.78 124.75 124.62 122.46 120.00 119.97 119.94 119.91 119.88 116.13 115.17 114.79 114.30
55.56
210 200 190 180 170 160 150 140 130 120 110 100 90 80 70 60 50 40 30 20 10 0
f1 (ppm)

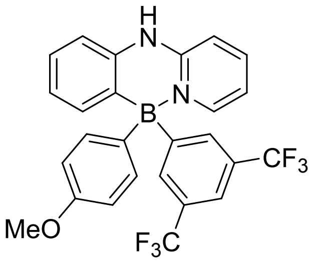

38

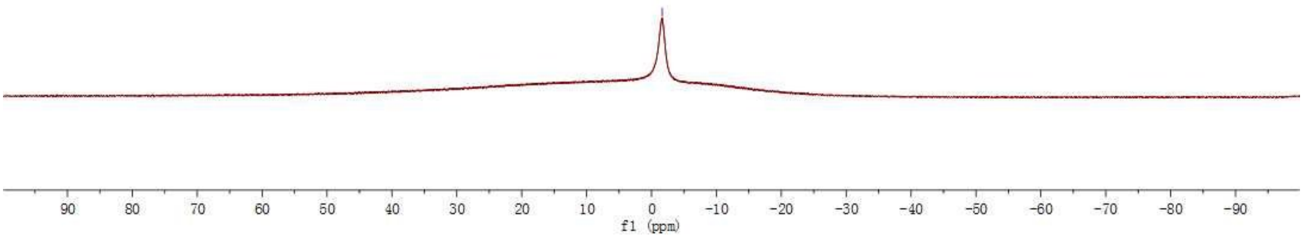

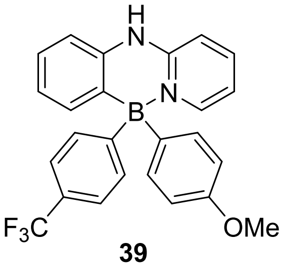

39

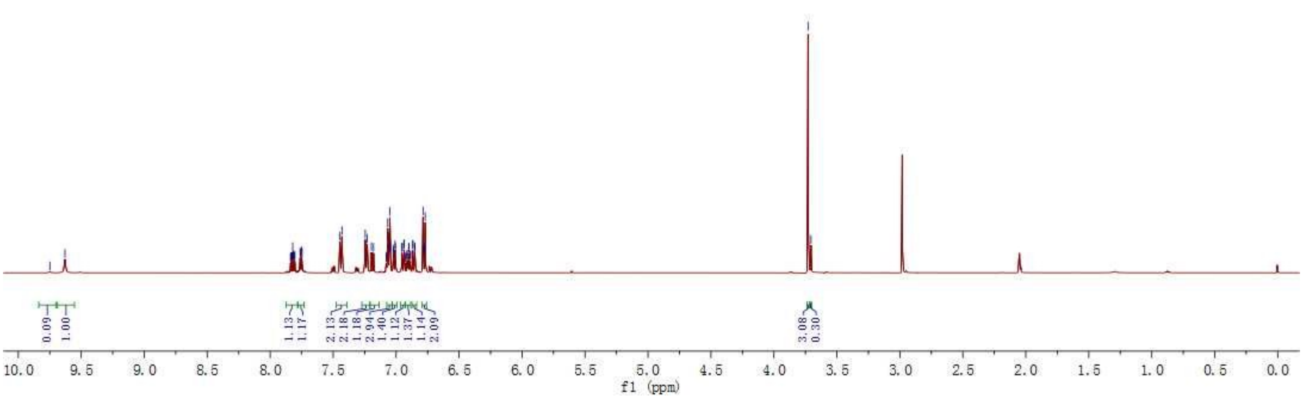

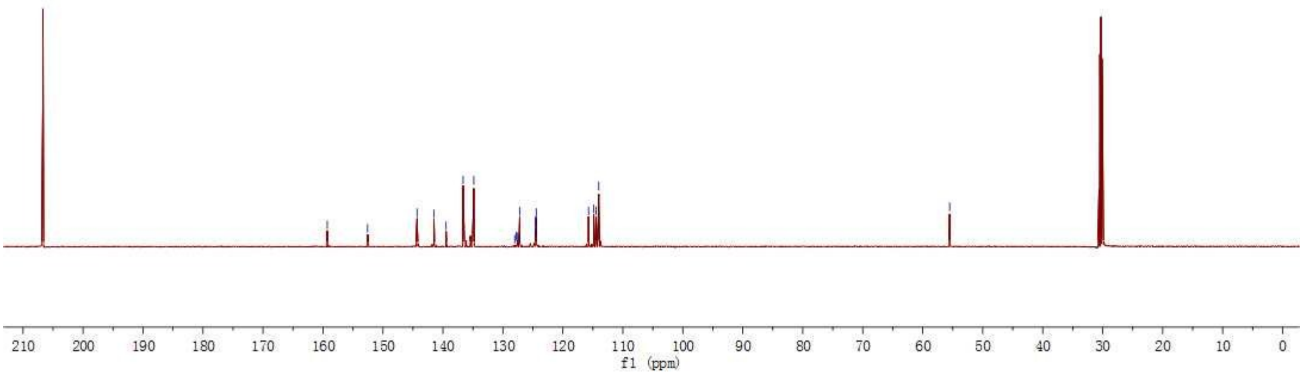

159.29
152.56
144.31
141.49
139.48
136.64
134.97
134.85
127.21
124.55
124.52
124.49
124.46
114.84
114.47
114.02
55.51
f1 (ppm)

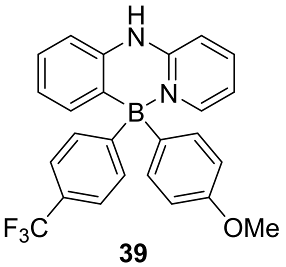

H
N
N
B
F3C
OMe

39

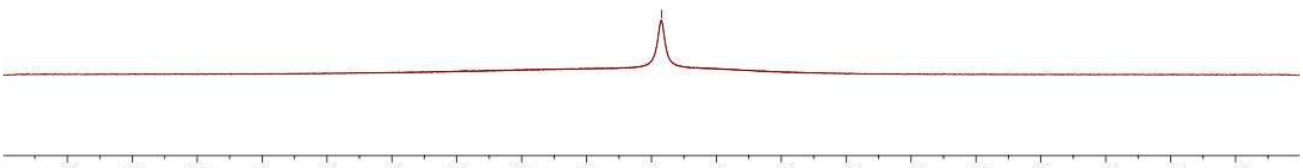

-1.53
f1 (ppm)

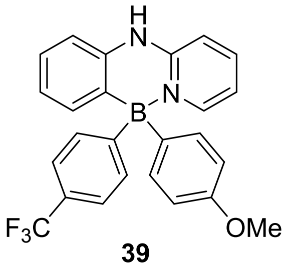

H
N
N
B
F3C
OMe

39

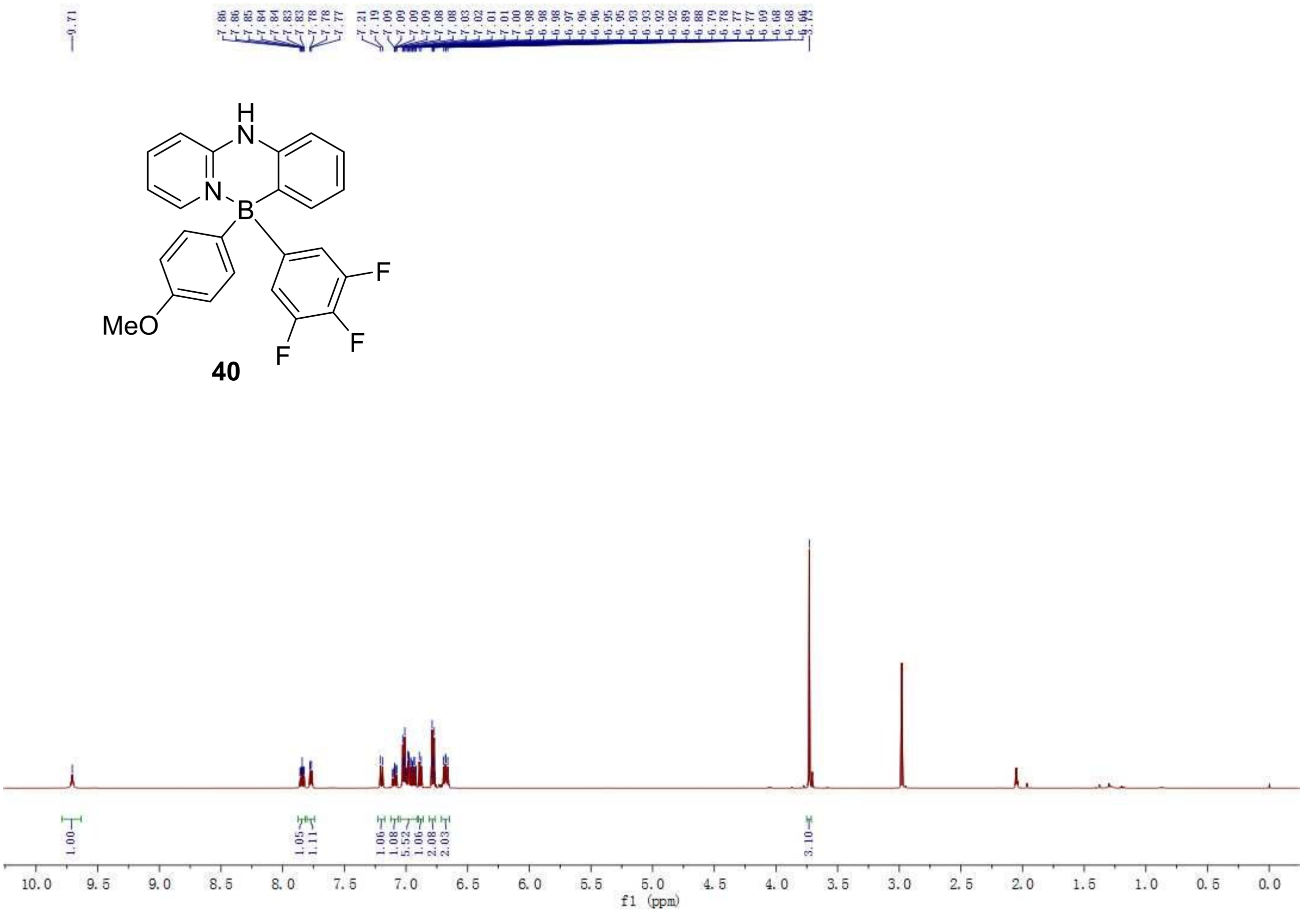
H
N
N
B
MeO
F
F
F
40
f1 (ppm)

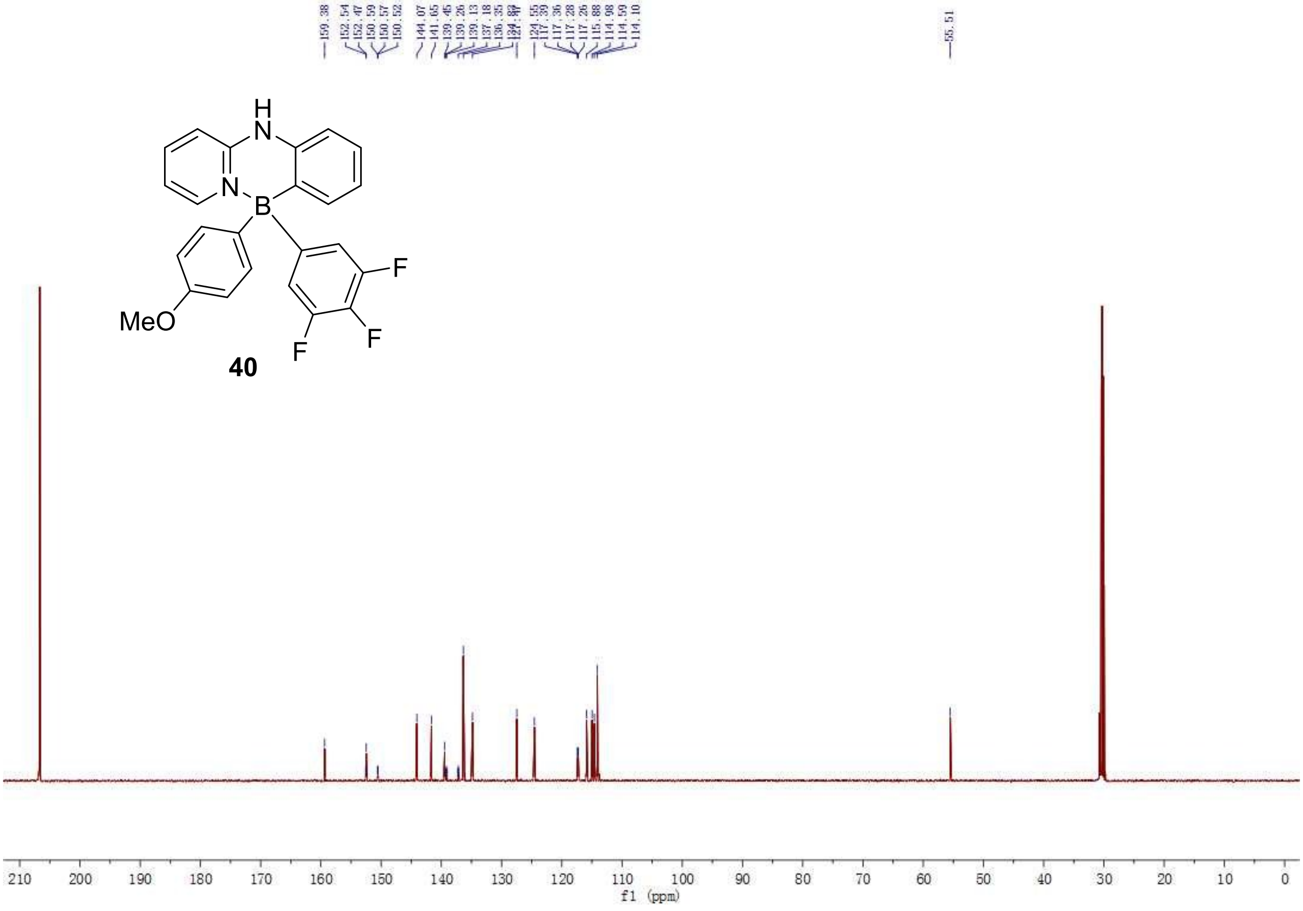
H
N
N
B
MeO
F
F
F
40
55.51
f1 (ppm)

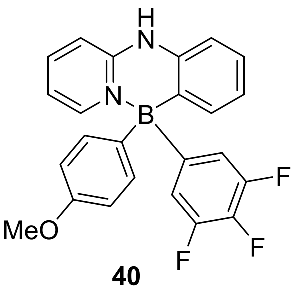

40

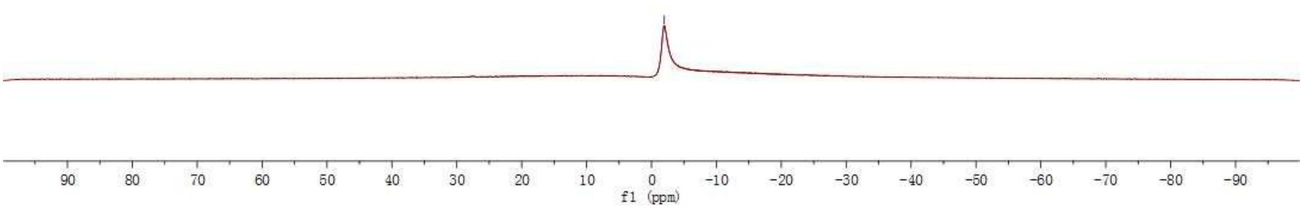

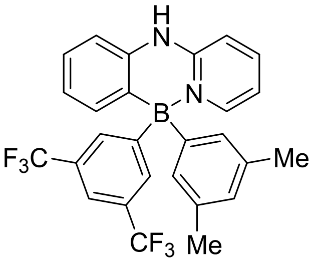

41

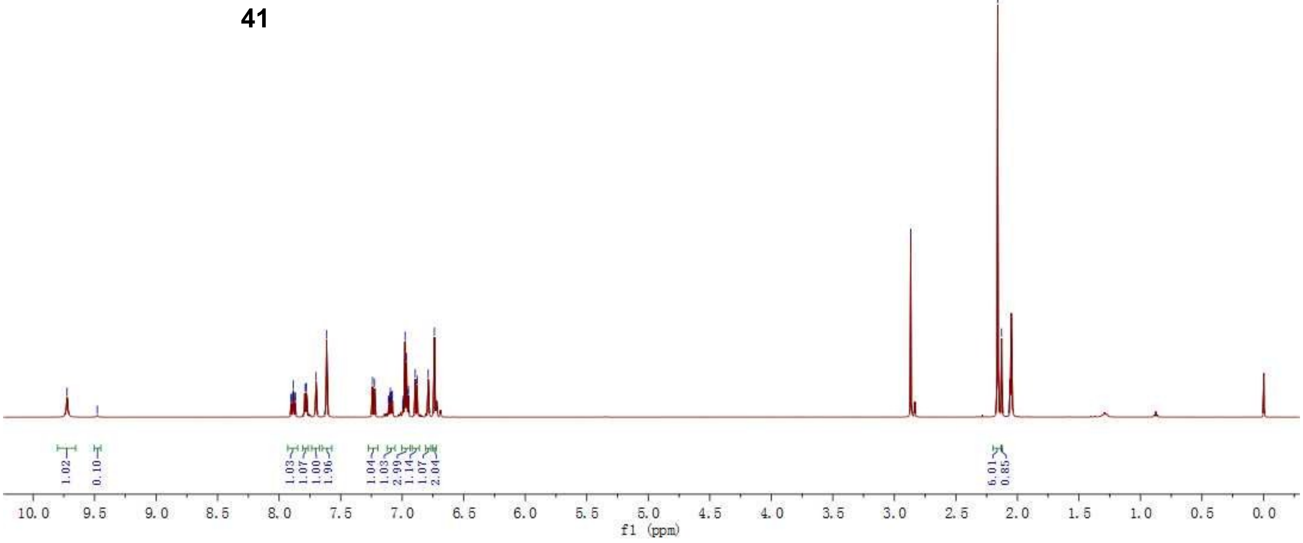

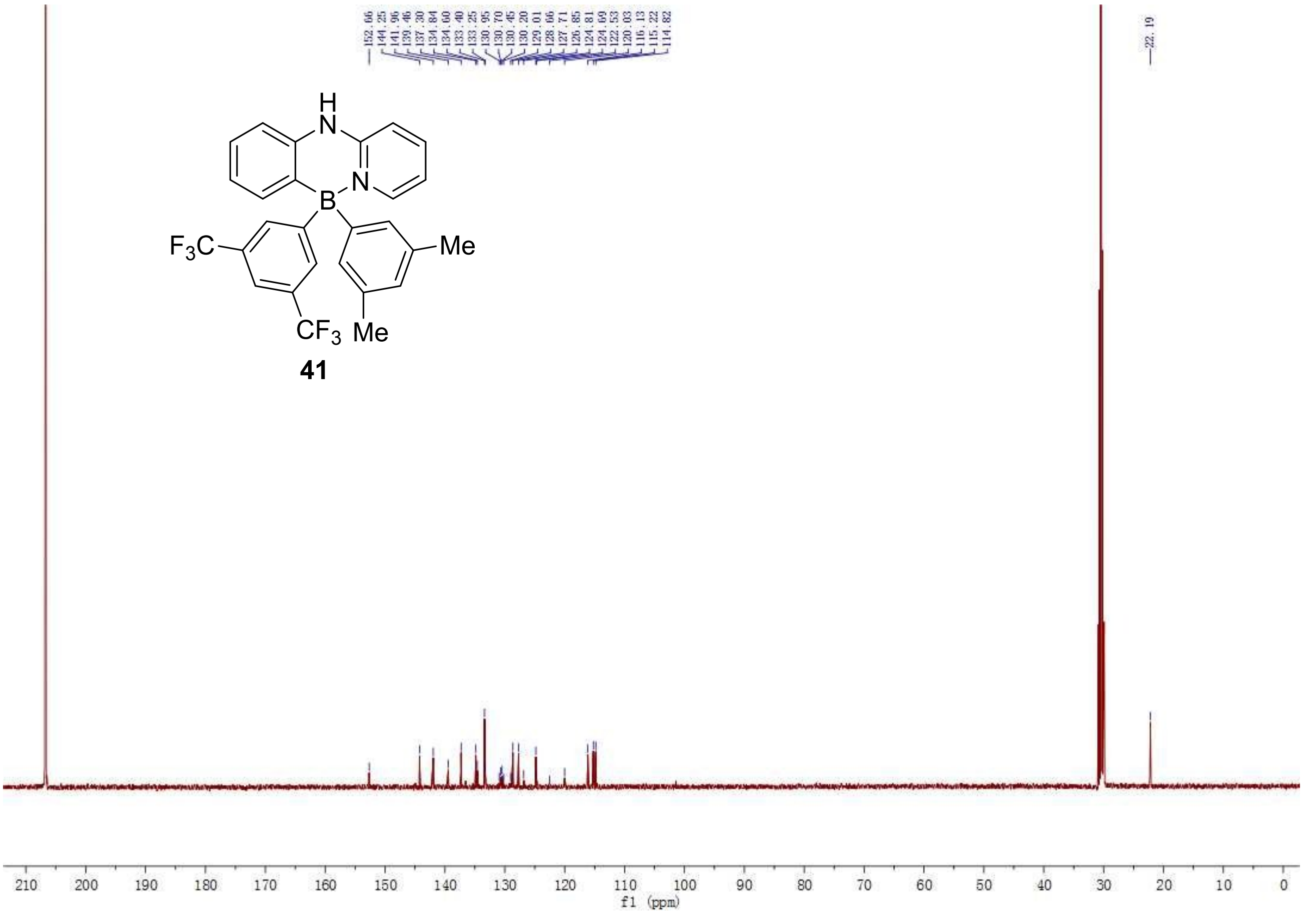
152.66
144.25
141.96
139.46
137.30
134.84
134.60
133.40
133.25
130.95
130.70
130.45
130.20
129.01
128.66
127.71
126.85
124.81
124.69
122.53
120.03
116.13
115.22
114.82
22.19
H
N
B
F3C
CF3
Me
41
f1 (ppm)

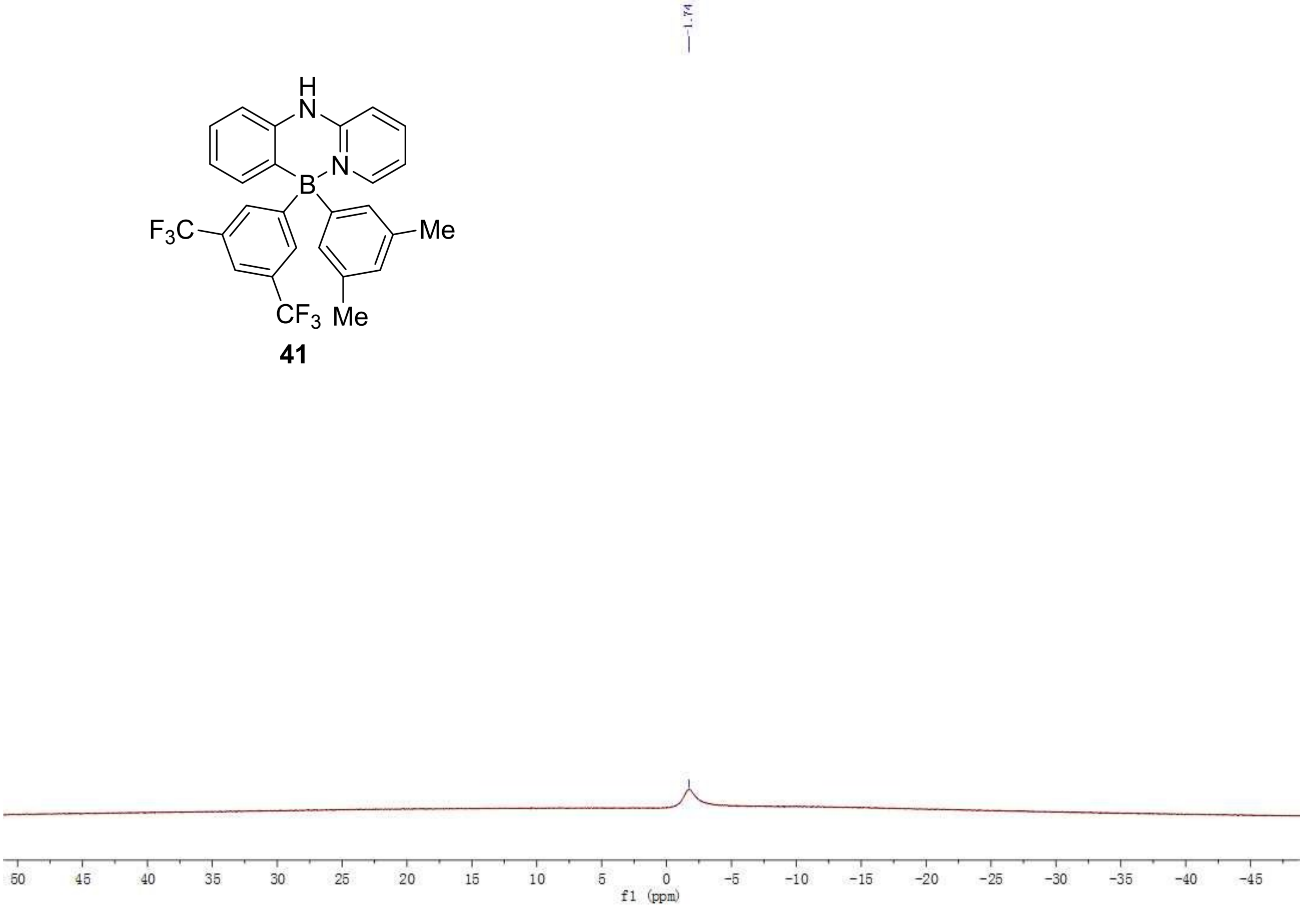
-1.74
H
N
B
F3C
CF3
Me
41
f1 (ppm)

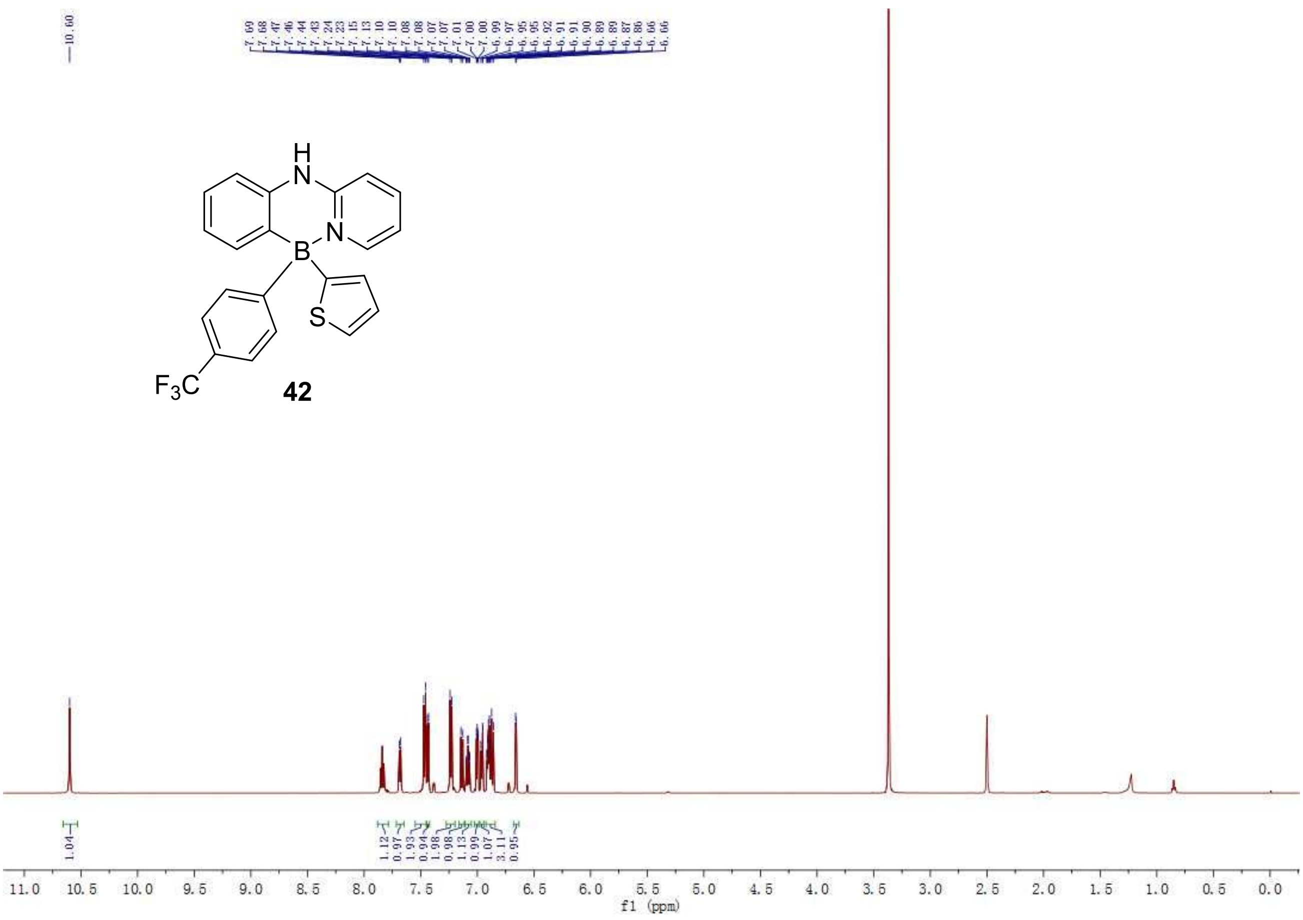
H
N
B
S
F3C
42
11.0 10.5 10.0 9.5 9.0 8.5 8.0 7.5 7.0 6.5 6.0 5.5 5.0 4.5 4.0 3.5 3.0 2.5 2.0 1.5 1.0 0.5 0.0
f1 (ppm)

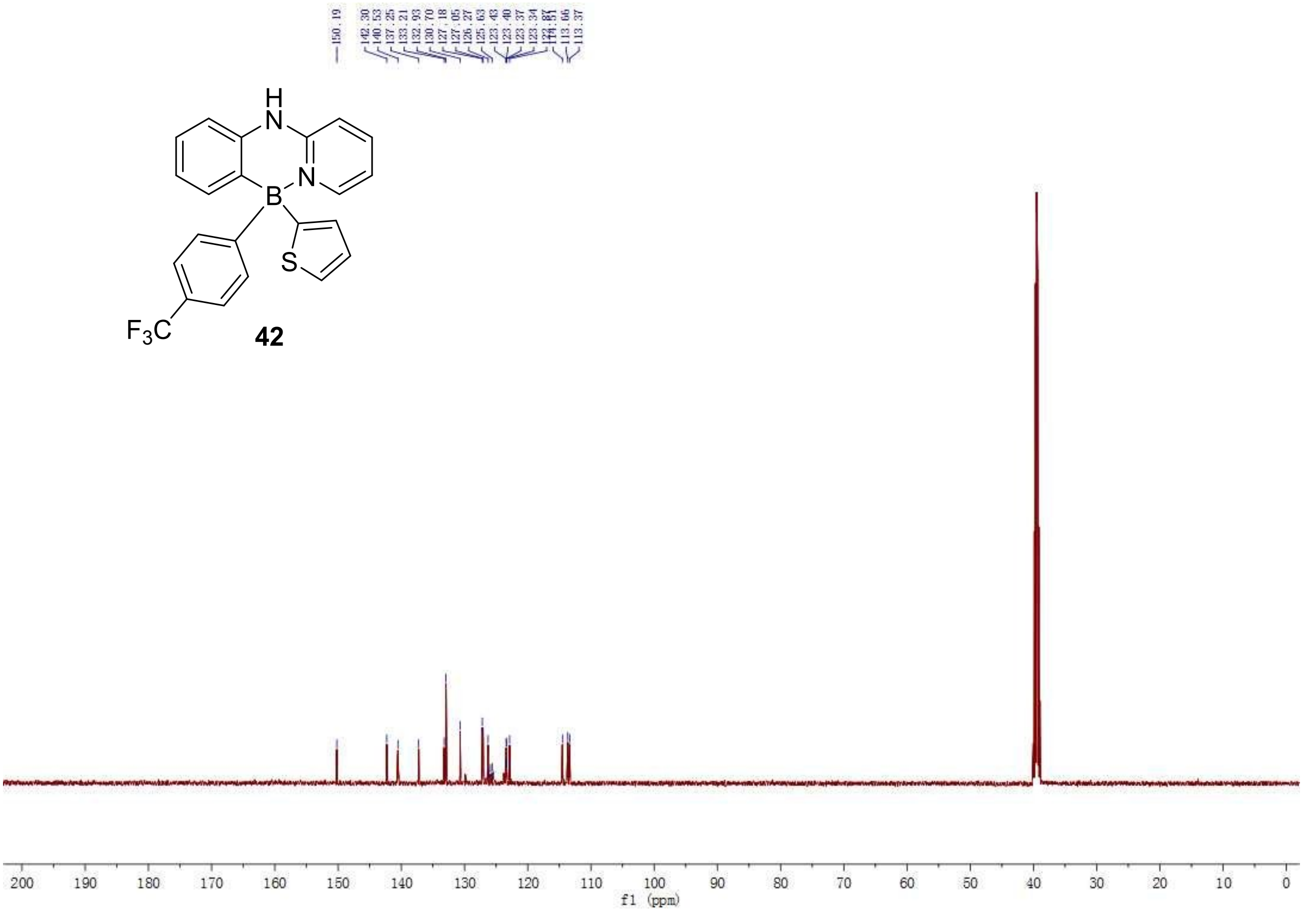
H
N
B
S
F3C
42
200 190 180 170 160 150 140 130 120 110 100 90 80 70 60 50 40 30 20 10 0
f1 (ppm)

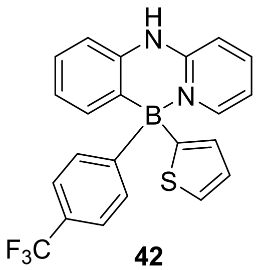

42

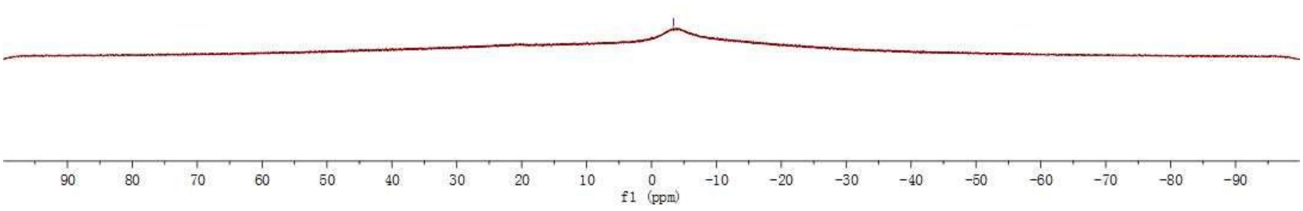

3.37
90 80 70 60 50 40 30 20 10 0 -10 -20 -30 -40 -50 -60 -70 -80 -90
f1 (ppm)

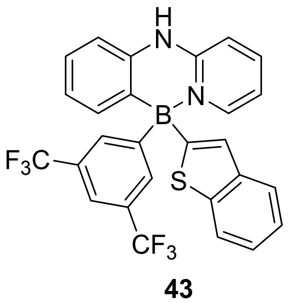

43

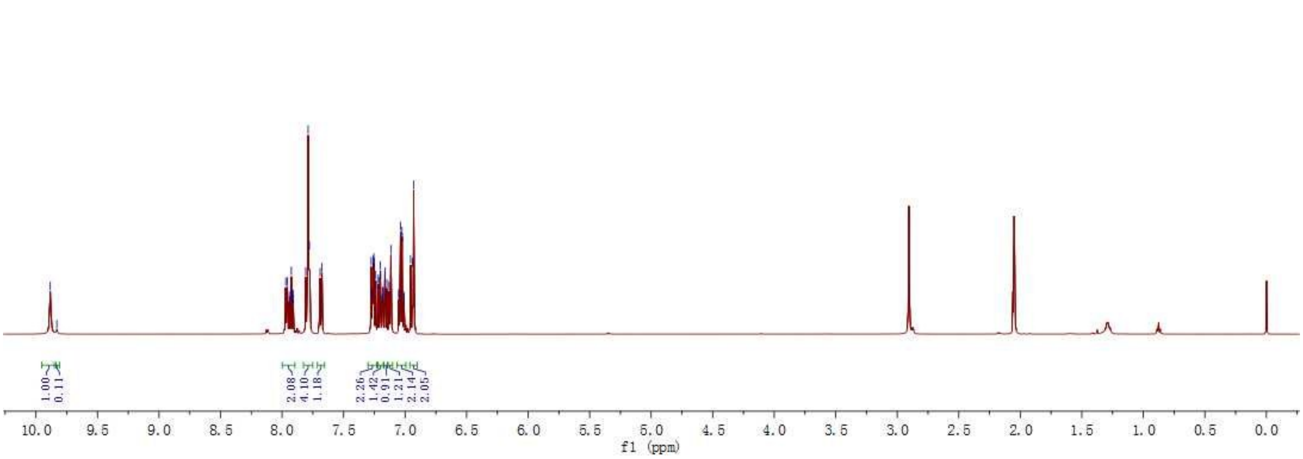

9.88
9.83
1.00
0.11
2.08
4.10
1.18
2.26
1.42
0.91
1.21
2.14
2.05
10.0 9.5 9.0 8.5 8.0 7.5 7.0 6.5 6.0 5.5 5.0 4.5 4.0 3.5 3.0 2.5 2.0 1.5 1.0 0.5 0.0
f1 (ppm)

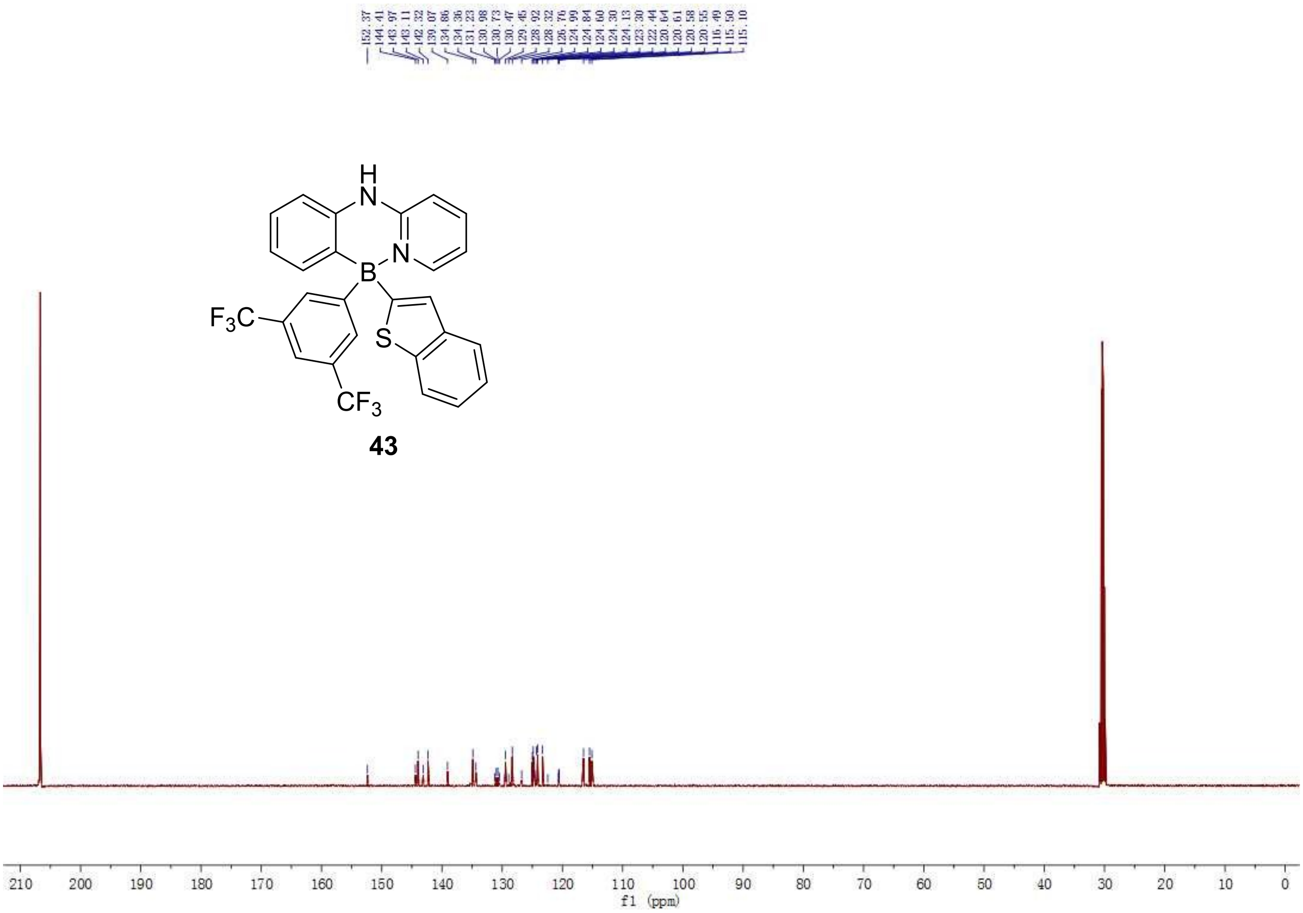
152.37
144.41
143.97
143.11
142.32
139.07
134.86
134.36
131.23
130.98
130.73
130.47
129.45
128.92
128.32
126.76
124.99
124.84
124.60
124.30
124.13
123.30
122.44
120.64
120.61
120.58
120.55
116.49
115.50
115.10
43
f1 (ppm)

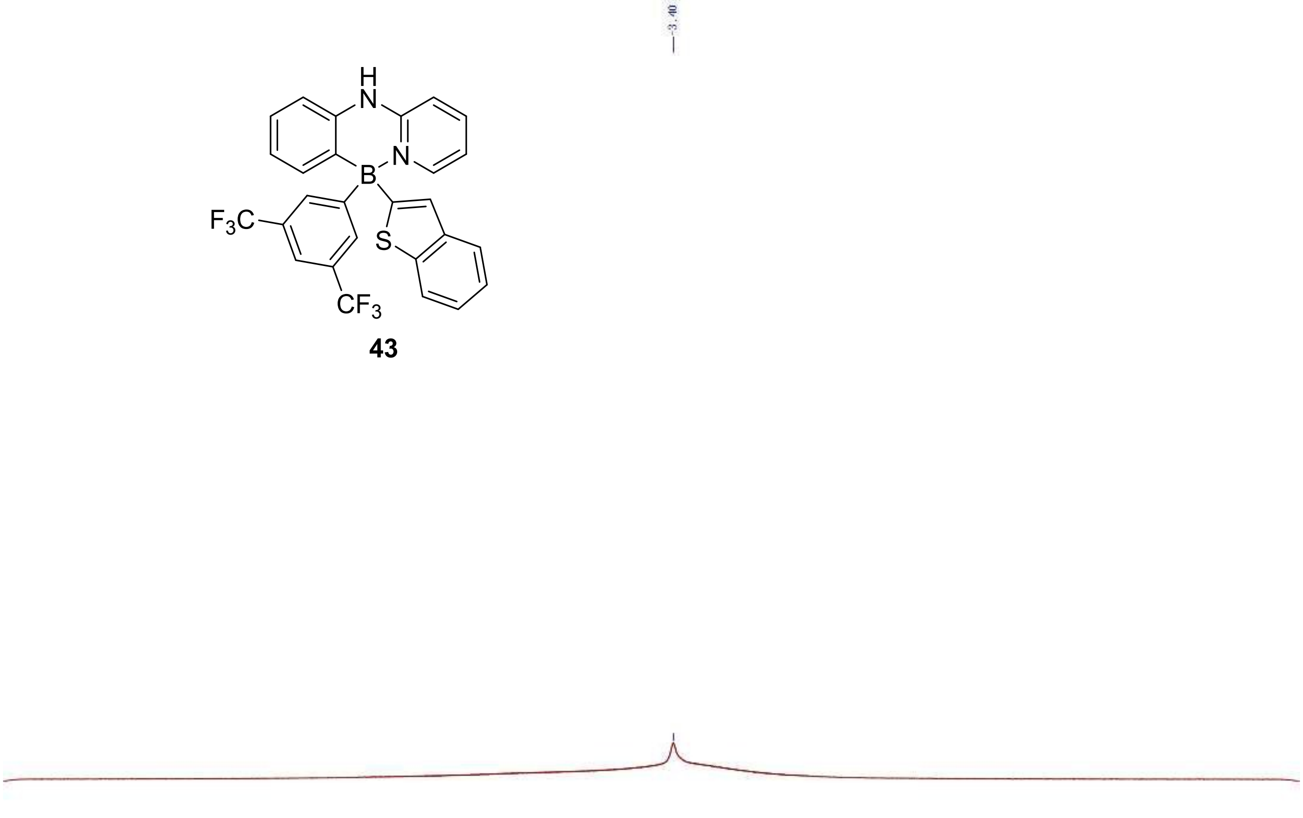
-3.40
43
f1 (ppm)

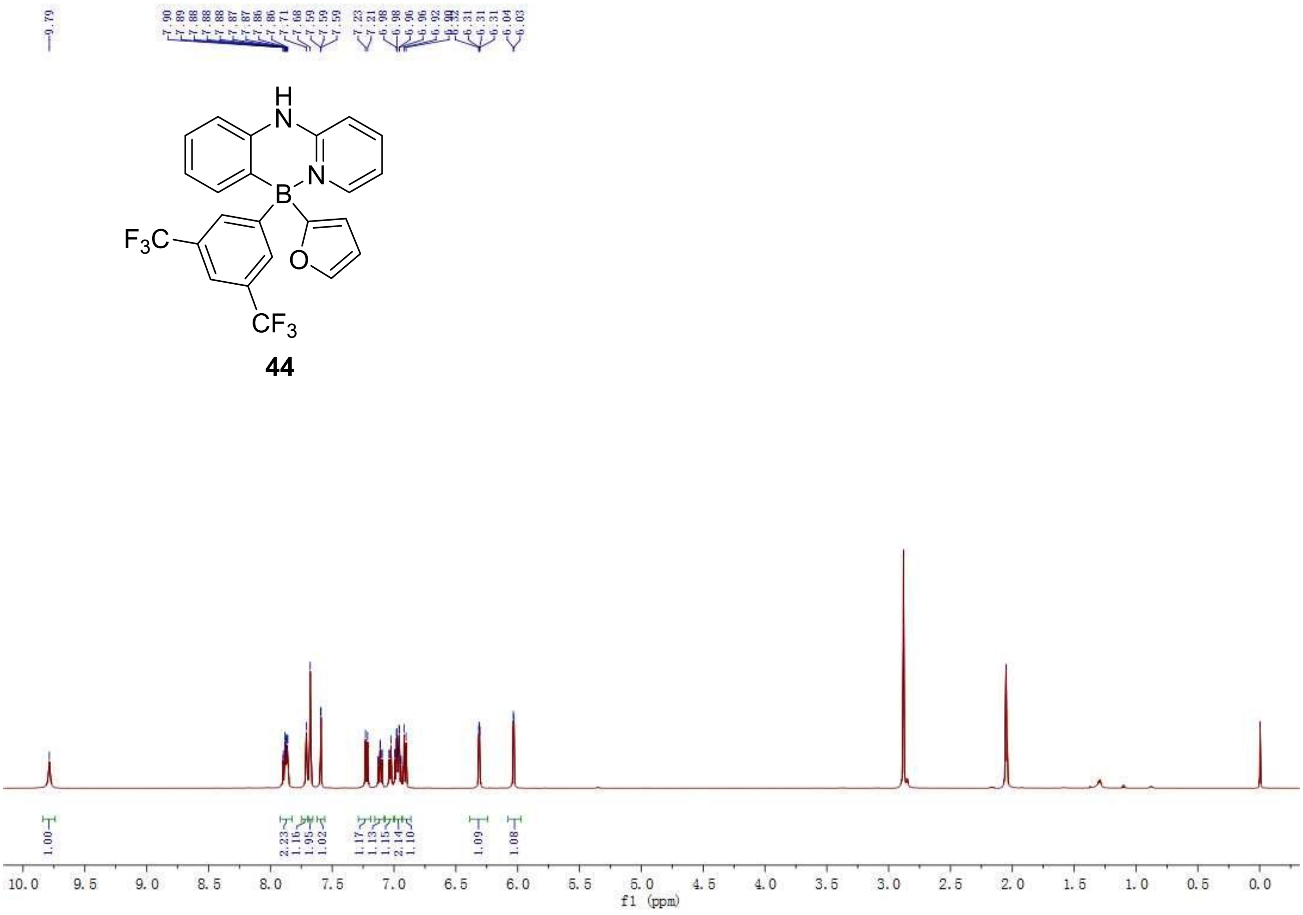
9.79
7.90 7.89 7.88 7.88 7.88 7.87 7.87 7.86 7.86 7.71 7.68 7.59 7.59 7.59
7.23 7.21 6.98 6.98 6.96 6.96 6.92
6.31 6.31 6.31 6.04 6.03
H
N
B
F3C
O
CF3
1.00
2.23 1.16 1.95 1.02
1.17 1.13 1.15 2.14 1.10
1.09
1.08
10.0 9.5 9.0 8.5 8.0 7.5 7.0 6.5 6.0 5.5 5.0 4.5 4.0 3.5 3.0 2.5 2.0 1.5 1.0 0.5 0.0
f1 (ppm)

44

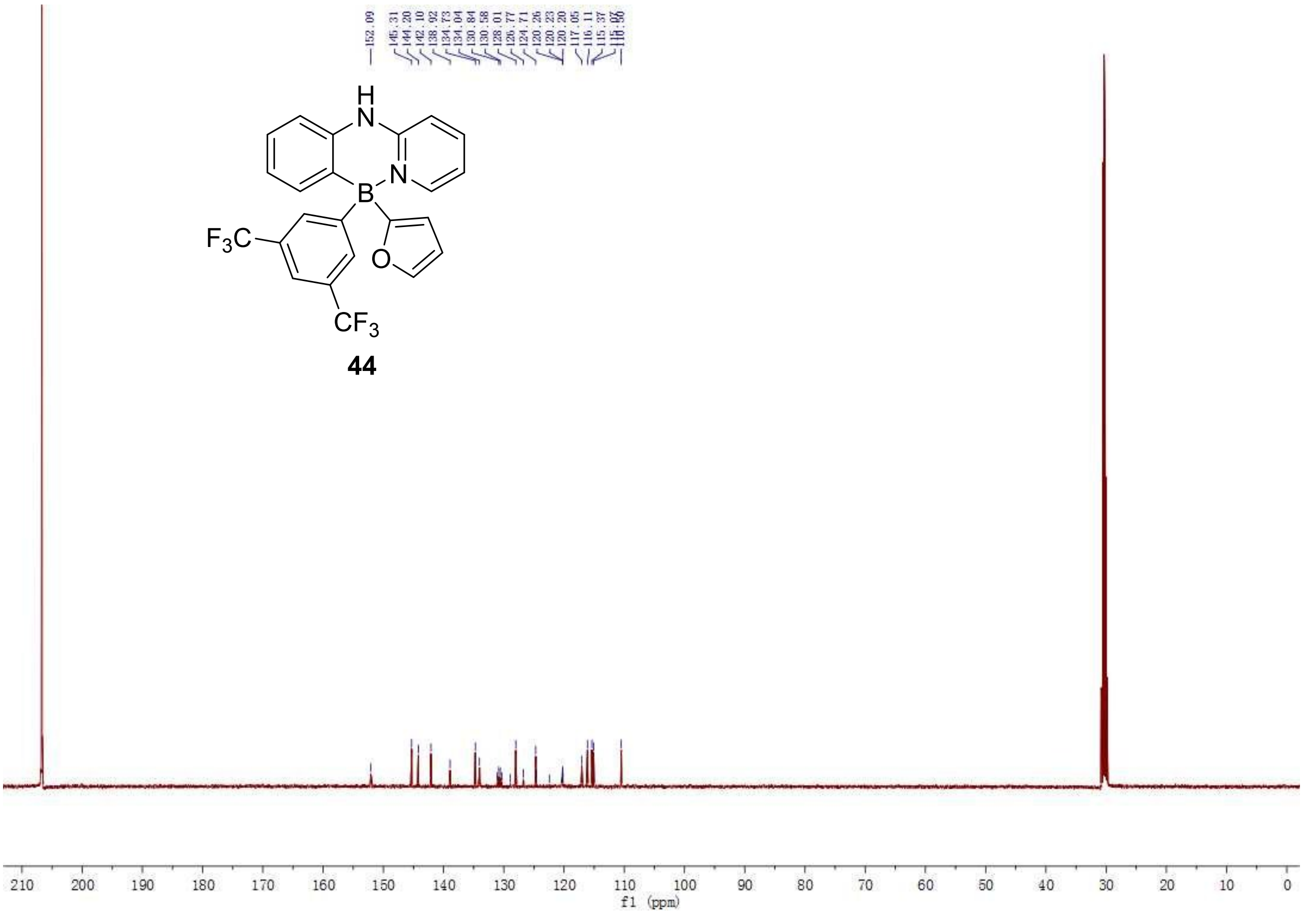
152.09
145.31 144.20 142.10 138.92 134.73 134.04 130.84 130.58 128.01 126.77 124.71 120.26 120.23 120.20 117.05 116.11 115.37
H
N
B
F3C
O
CF3
210 200 190 180 170 160 150 140 130 120 110 100 90 80 70 60 50 40 30 20 10 0
f1 (ppm)

44

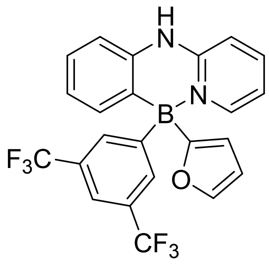

44

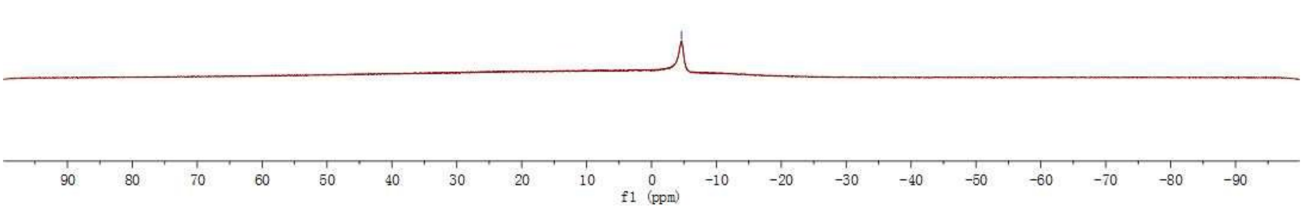

-4.61
90 80 70 60 50 40 30 20 10 0 -10 -20 -30 -40 -50 -60 -70 -80 -90
f1 (ppm)

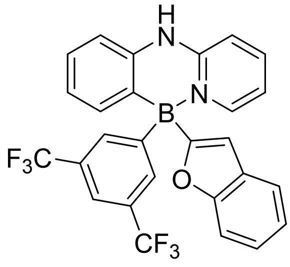

45

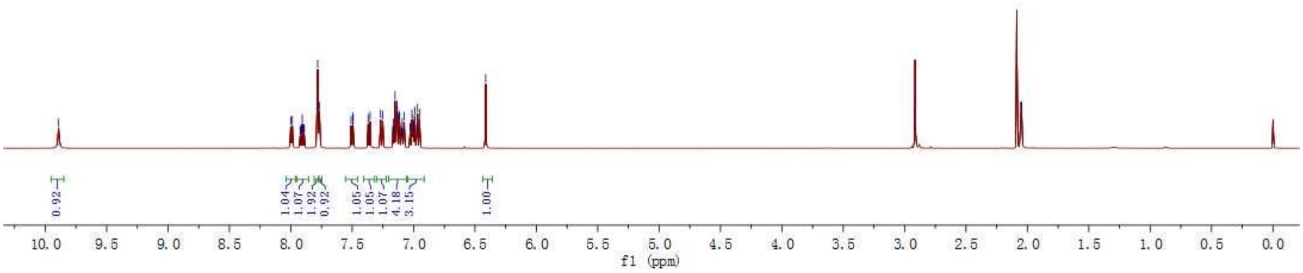

9.89
8.00 7.99 7.92 7.92 7.91 7.91 7.90 7.89 7.89 7.78 7.77
7.35 7.27 7.25 7.15 7.14 7.13 7.12 7.01 6.99 6.97 6.95 6.41
0.92
1.04 1.07 1.92 0.92
1.05 1.05 1.07 4.18 3.15
1.00
10.0 9.5 9.0 8.5 8.0 7.5 7.0 6.5 6.0 5.5 5.0 4.5 4.0 3.5 3.0 2.5 2.0 1.5 1.0 0.5 0.0
f1 (ppm)

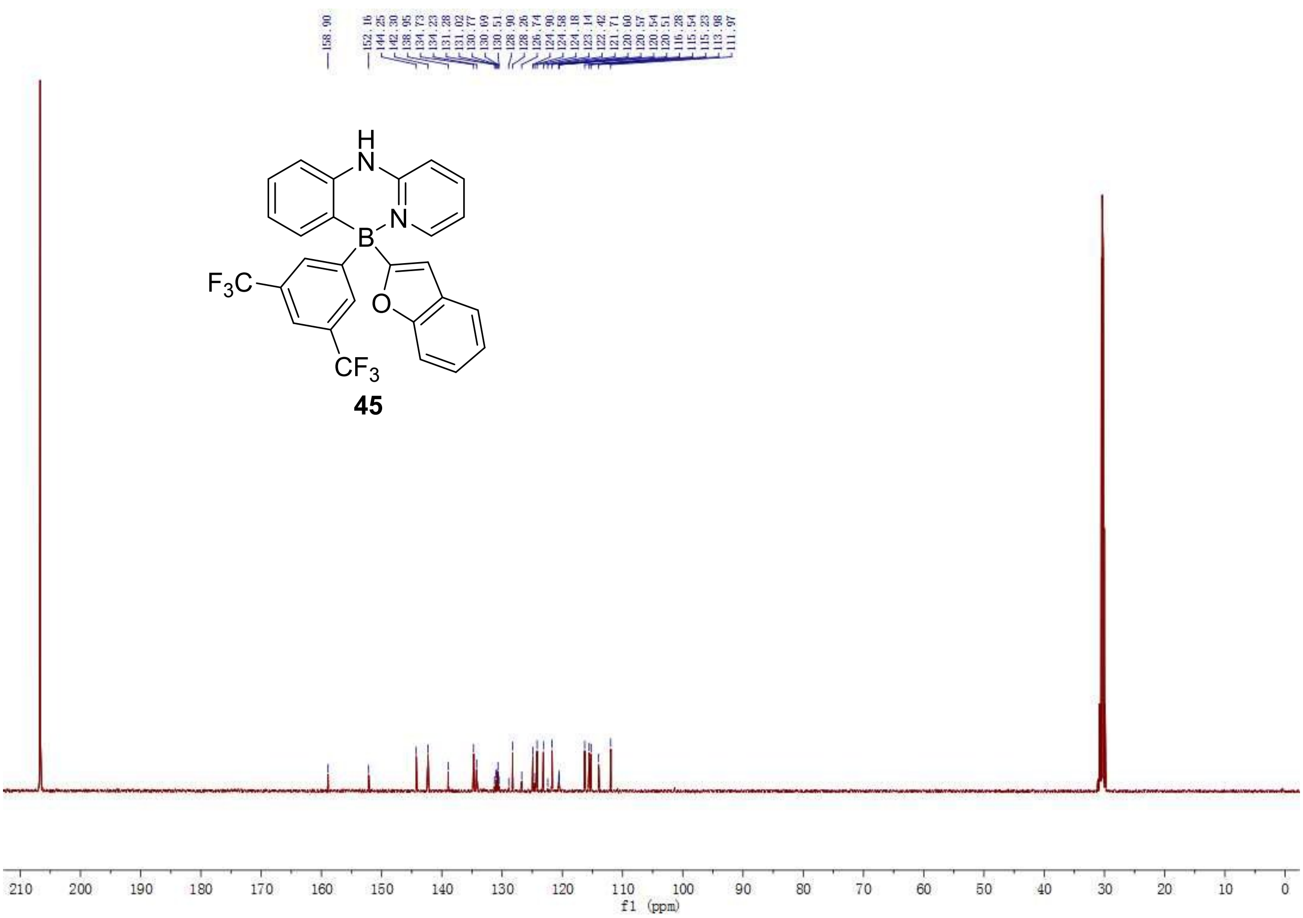
158.90
152.16
144.25
142.30
138.95
134.73
134.23
131.28
131.02
130.77
130.69
130.51
128.90
128.26
126.74
124.90
124.58
124.18
123.14
122.42
121.71
120.60
120.57
120.54
120.51
116.28
115.54
115.23
113.98
111.97
45
f1 (ppm)

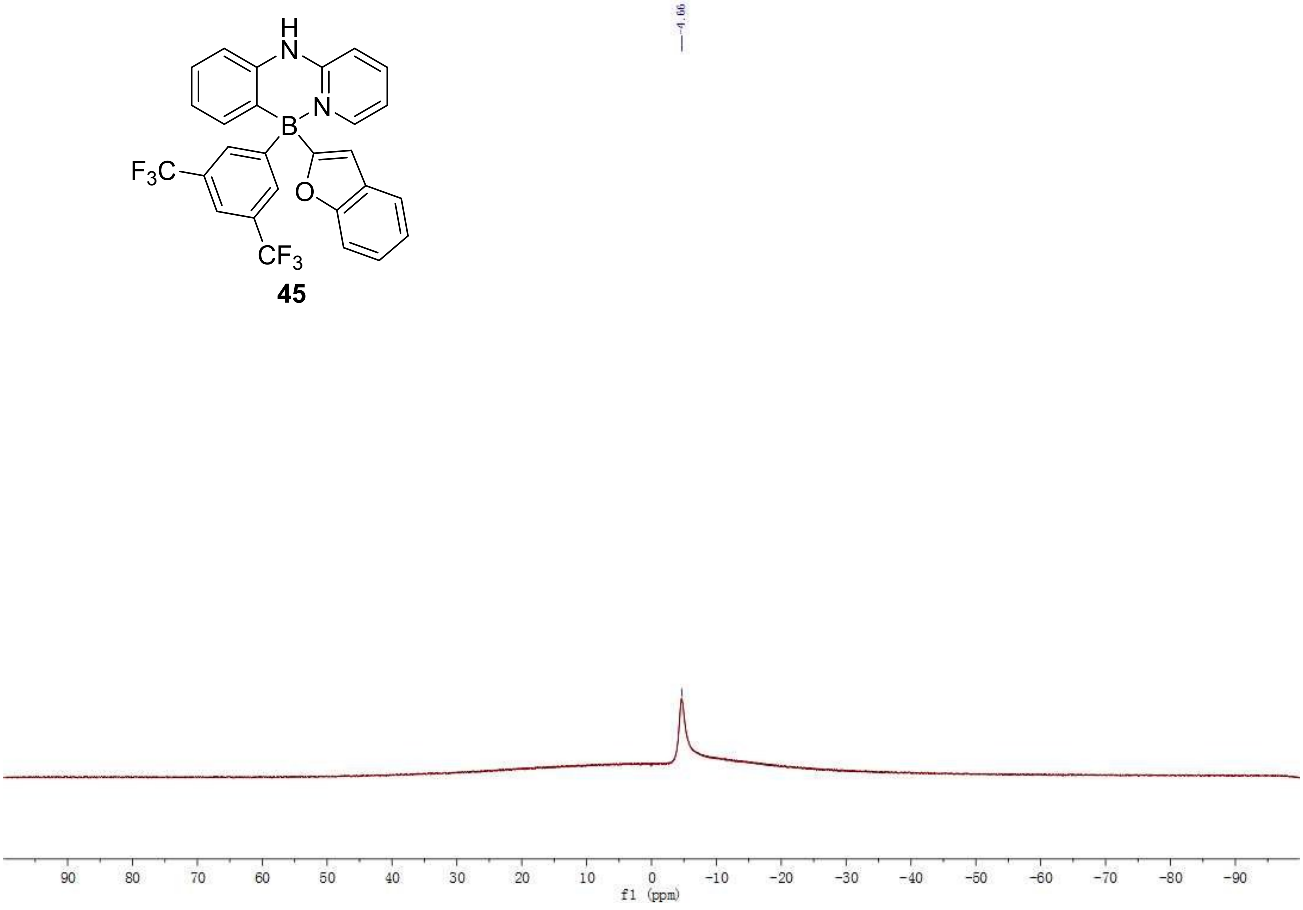
-4.66
45
f1 (ppm)

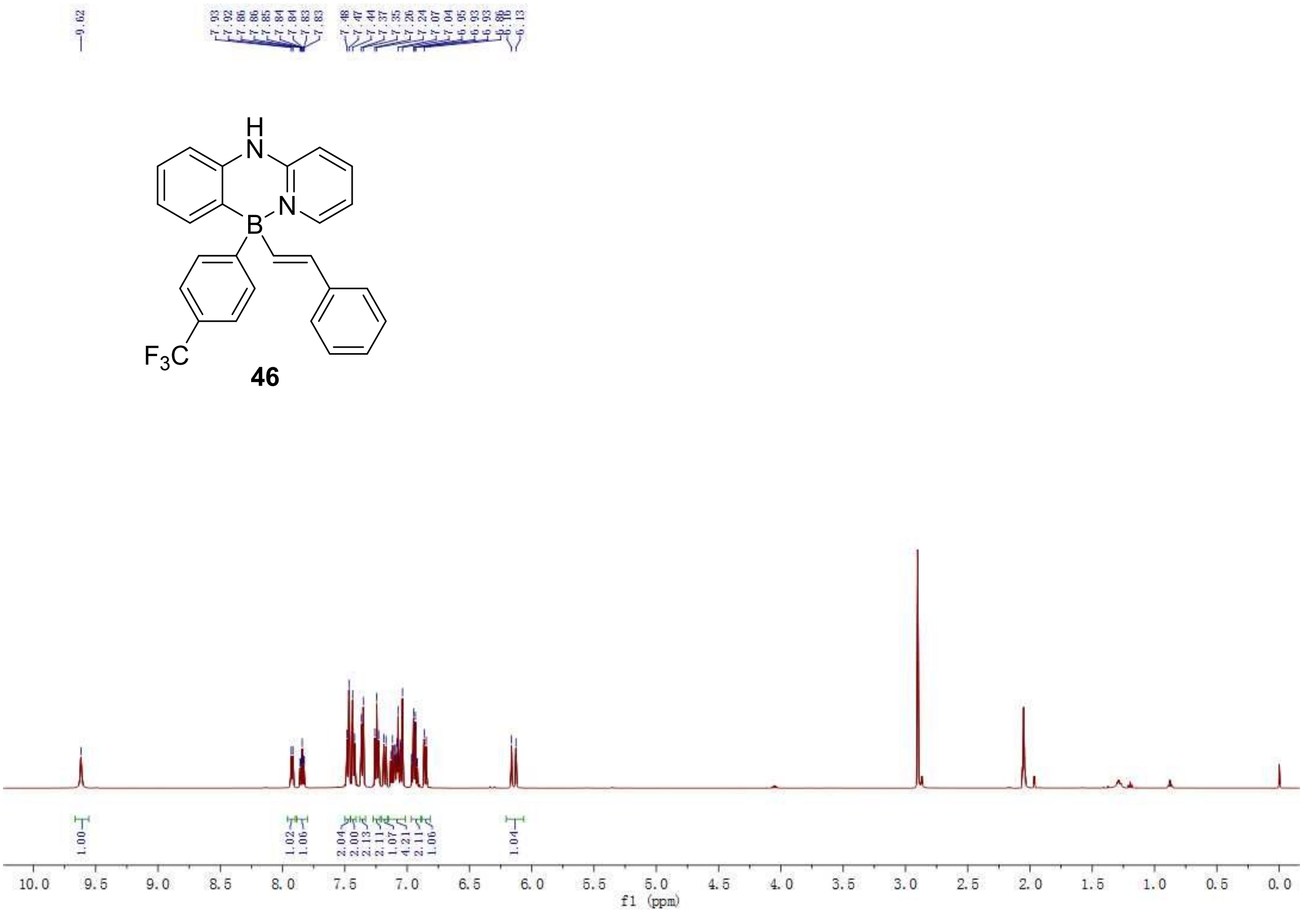
46
F3C
f1 (ppm)

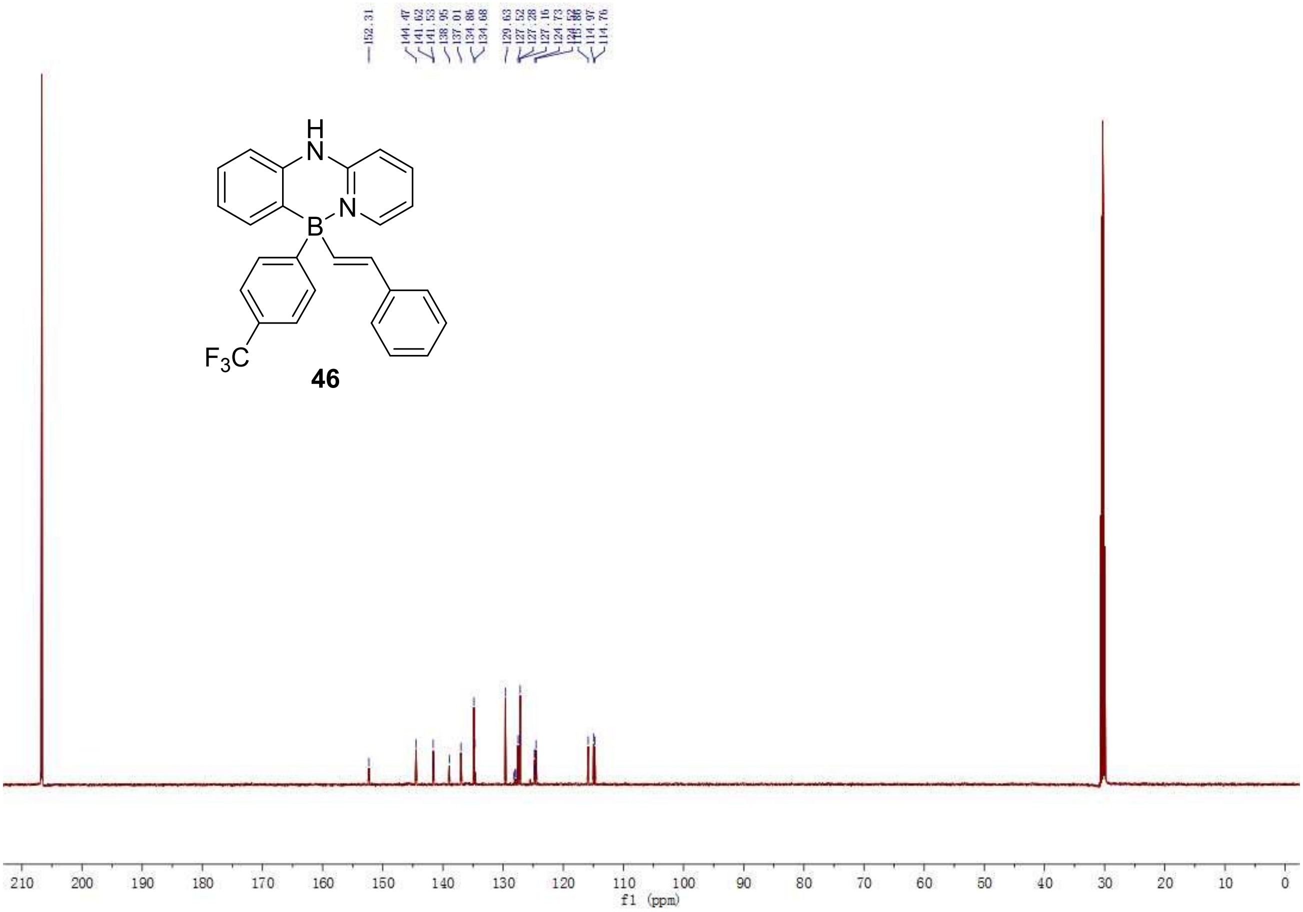
46
F3C
f1 (ppm)

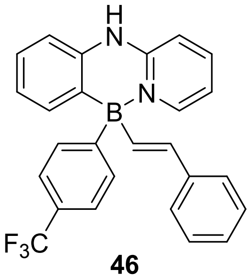

46

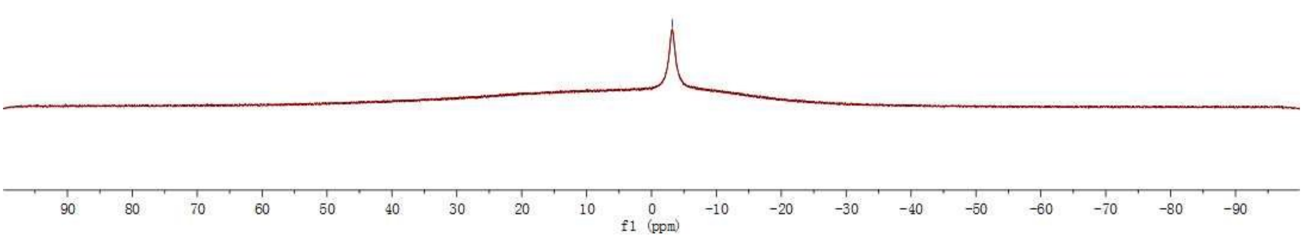

-3.23
90 80 70 60 50 40 30 20 10 0 -10 -20 -30 -40 -50 -60 -70 -80 -90
f1 (ppm)

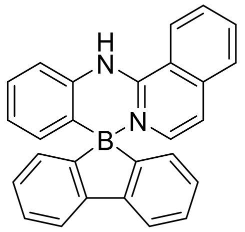

47

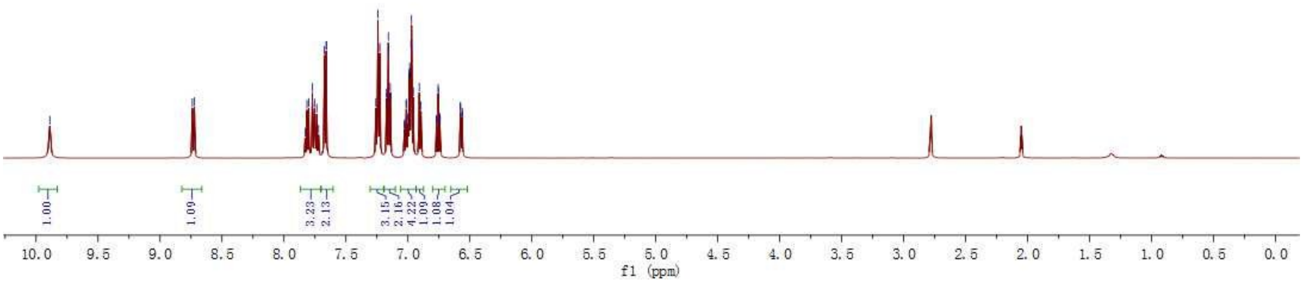

9.89
8.74 8.72
7.83 7.81 7.80 7.77 7.75 7.73 7.72 7.67 7.66 7.66 7.26 7.24 7.23 7.17 7.17 7.16 7.16 7.14 7.14 7.03 7.03 7.01 7.01 7.00 7.00 6.99 6.99 6.98 6.98 6.97 6.96 6.91 6.89 6.77 6.77 6.76 6.75 6.74 6.74 6.58 6.57 6.56 6.56
1.00 1.09 3.23 2.13 3.15 2.16 4.22 1.09 1.08 1.04
10.0 9.5 9.0 8.5 8.0 7.5 7.0 6.5 6.0 5.5 5.0 4.5 4.0 3.5 3.0 2.5 2.0 1.5 1.0 0.5 0.0
f1 (ppm)

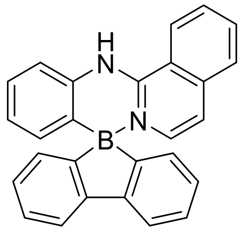

47

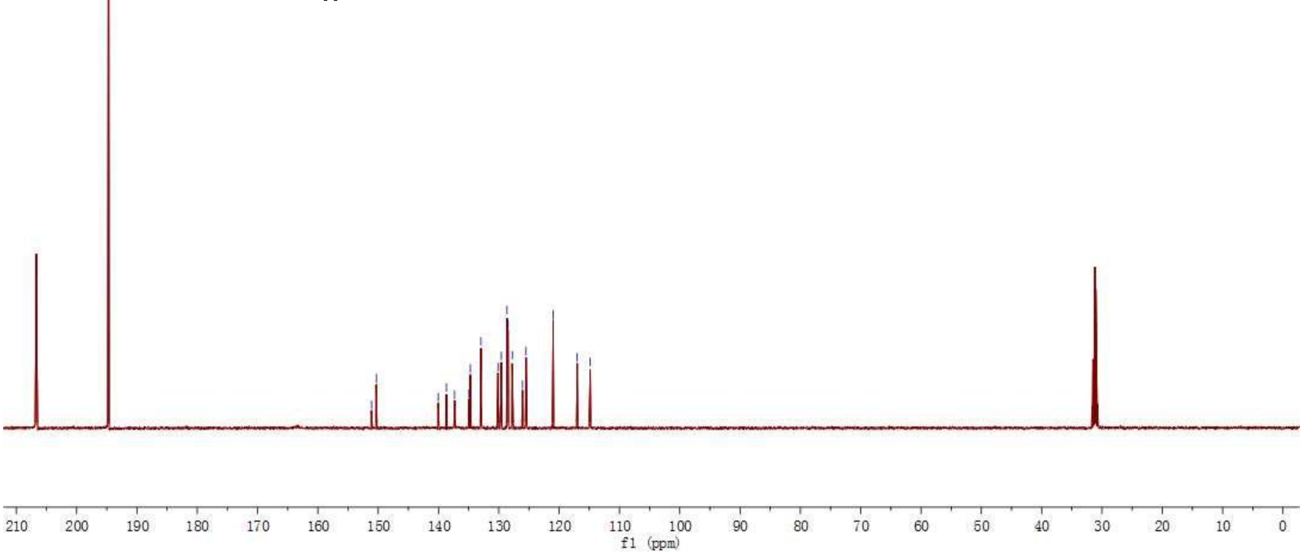

151.10
150.30
138.68
137.33
134.95
134.73
132.96
130.13
129.59
128.64
128.46
127.77
126.07
117.00
114.85
210 200 190 180 170 160 150 140 130 120 110 100 90 80 70 60 50 40 30 20 10 0
f1 (ppm)

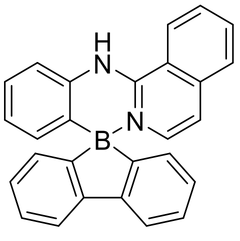

47

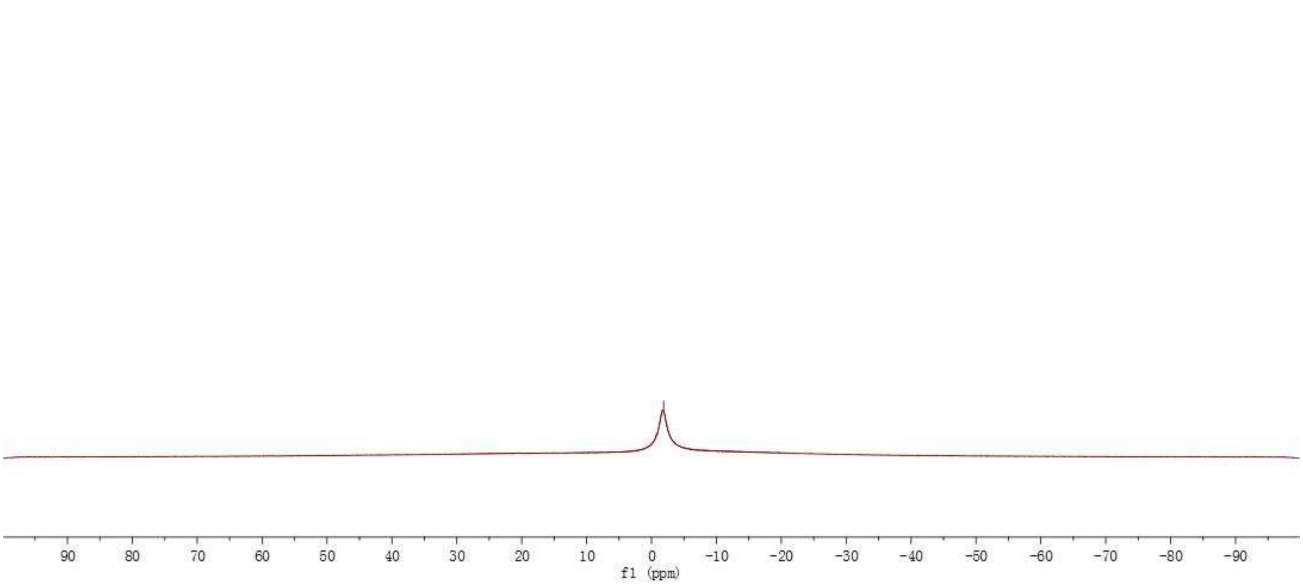

-1.87
90 80 70 60 50 40 30 20 10 0 -10 -20 -30 -40 -50 -60 -70 -80 -90
f1 (ppm)

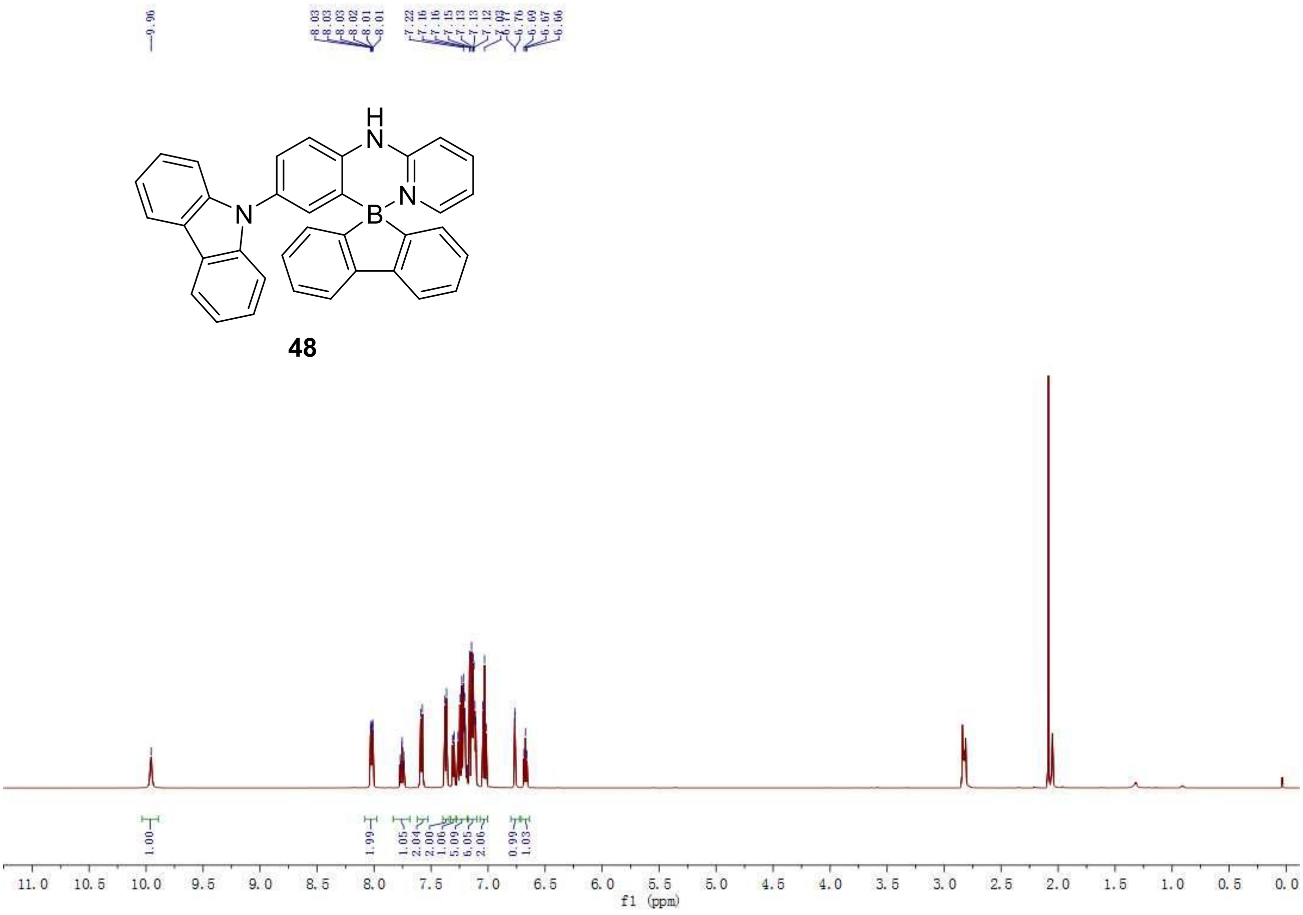

9.96
8.03 8.03 8.02 8.01 8.01
7.22 7.16 7.15 7.13 7.13 7.12 6.76 6.69 6.67
H
N
B
48
1.00
1.99
1.05 2.04 2.00 1.00 5.09 6.05 2.06
0.99 1.03
11.0 10.5 10.0 9.5 9.0 8.5 8.0 7.5 7.0 6.5 6.0 5.5 5.0 4.5 4.0 3.5 3.0 2.5 2.0 1.5 1.0 0.5 0.0
f1 (ppm)

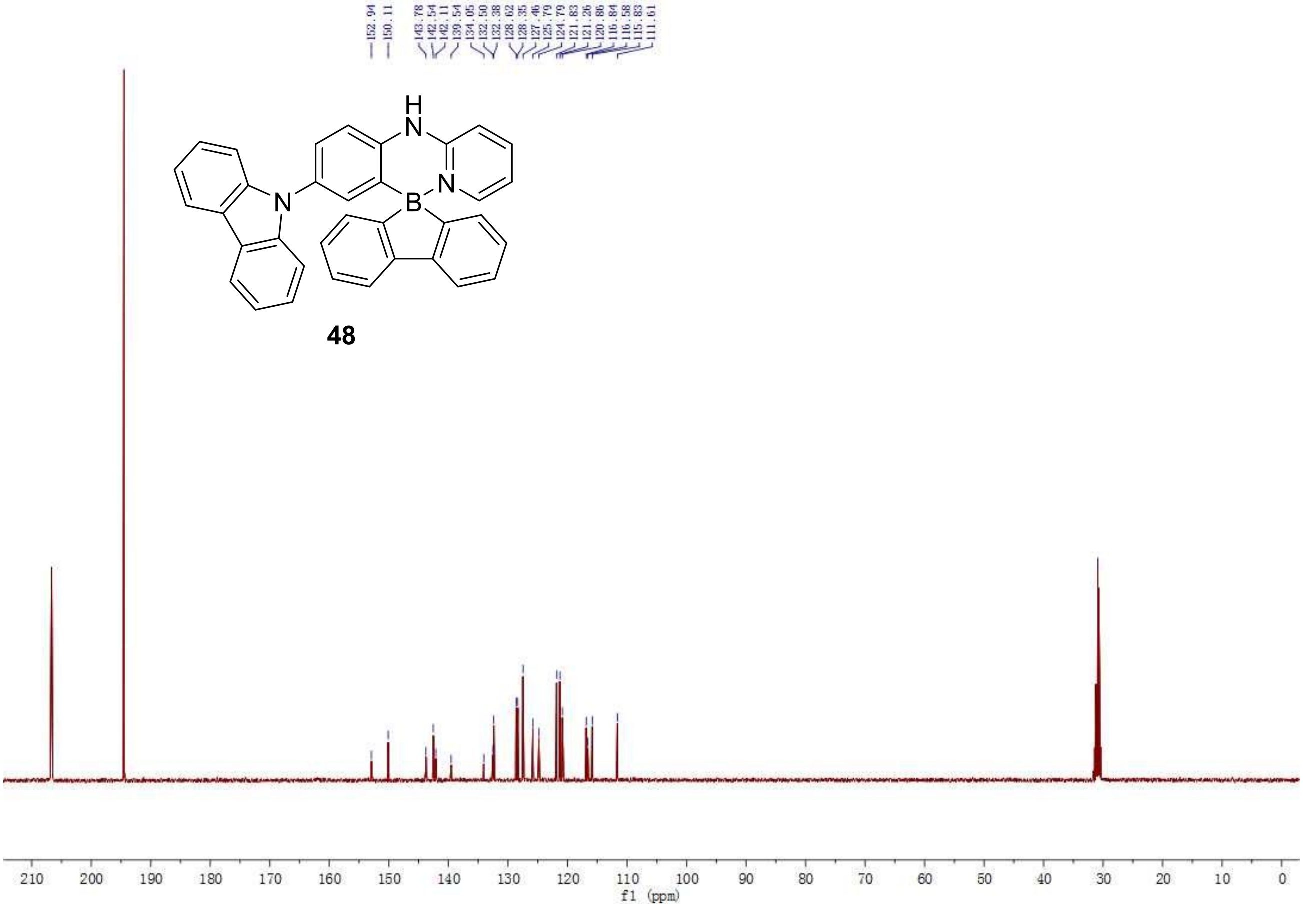

152.94
150.11
143.78 142.54 142.11 139.54 134.05 132.50 132.38 128.62 128.35 127.46 125.79 124.79 121.83 121.26 120.86 116.84 116.58 115.83 111.61
H
N
B
48
210 200 190 180 170 160 150 140 130 120 110 100 90 80 70 60 50 40 30 20 10 0
f1 (ppm)

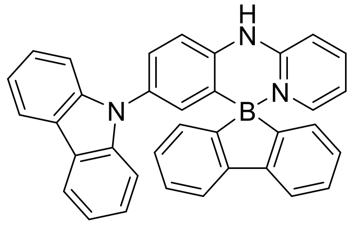

48

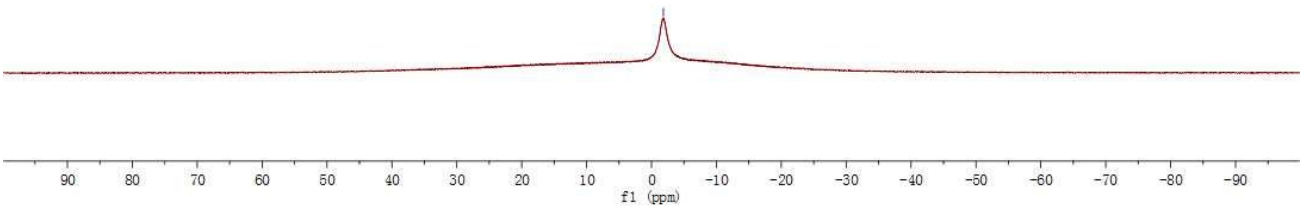

1.83
90 80 70 60 50 40 30 20 10 0 −10 −20 −30 −40 −50 −60 −70 −80 −90
f1 (ppm)

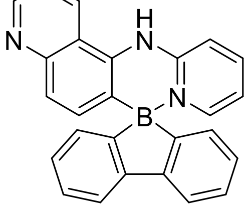

49

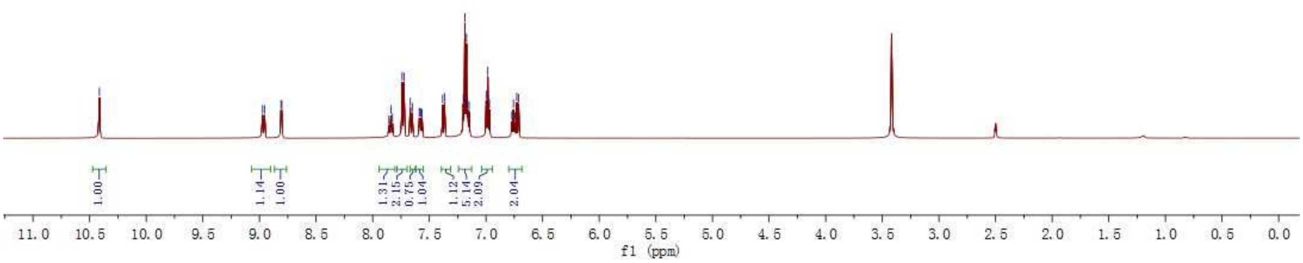

10.41
8.97 8.96 8.81 8.80 7.86 7.84 7.83 7.74 7.72 7.67 7.65 7.59 7.58 7.57 7.56 7.38 7.37 7.20 7.19 7.17 7.16 7.15 7.00 6.98 6.97 6.77 6.76 6.74 6.73 6.71
1.00 1.14 1.00 1.31 2.15 0.75 1.04 1.12 5.14 2.09 2.04
11.0 10.5 10.0 9.5 9.0 8.5 8.0 7.5 7.0 6.5 6.0 5.5 5.0 4.5 4.0 3.5 3.0 2.5 2.0 1.5 1.0 0.5 0.0
f1 (ppm)

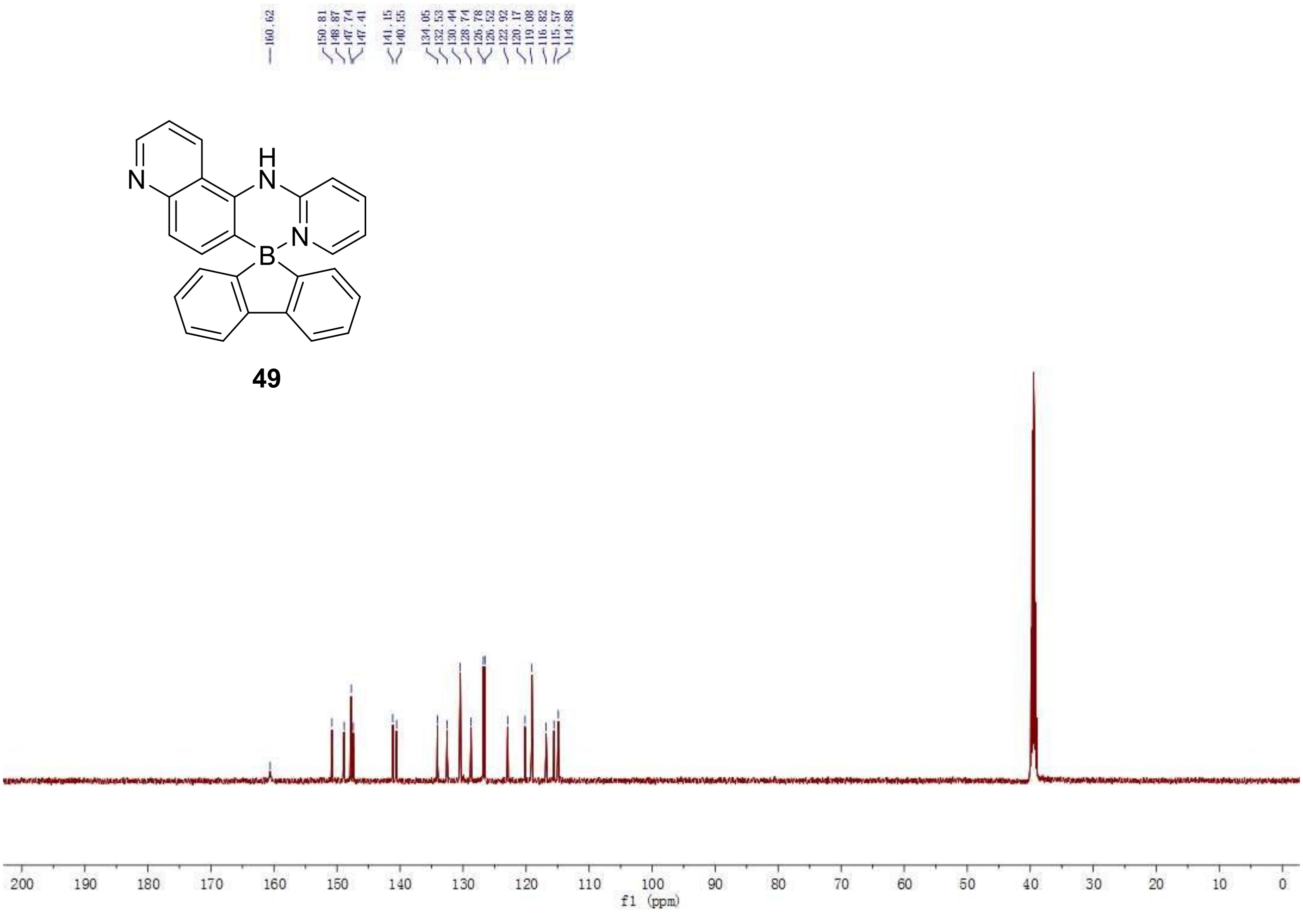
160.62
150.81
148.87
147.74
147.41
141.15
140.55
134.05
132.53
130.44
128.74
126.78
126.52
122.92
120.17
119.08
116.82
115.57
114.88
200 190 180 170 160 150 140 130 120 110 100 90 80 70 60 50 40 30 20 10 0
f1 (ppm)

49

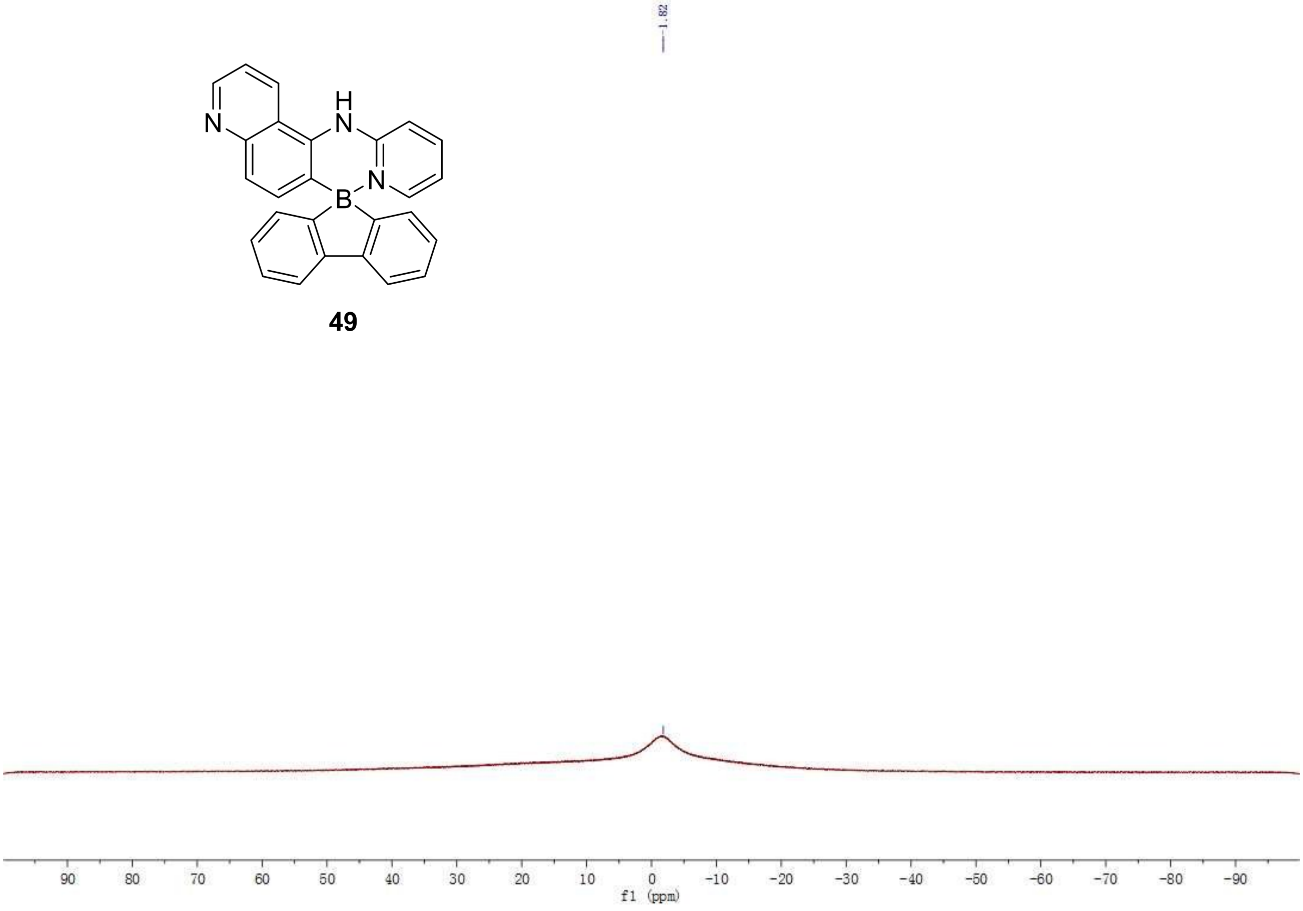
1.82
90 80 70 60 50 40 30 20 10 0 -10 -20 -30 -40 -50 -60 -70 -80 -90
f1 (ppm)

49

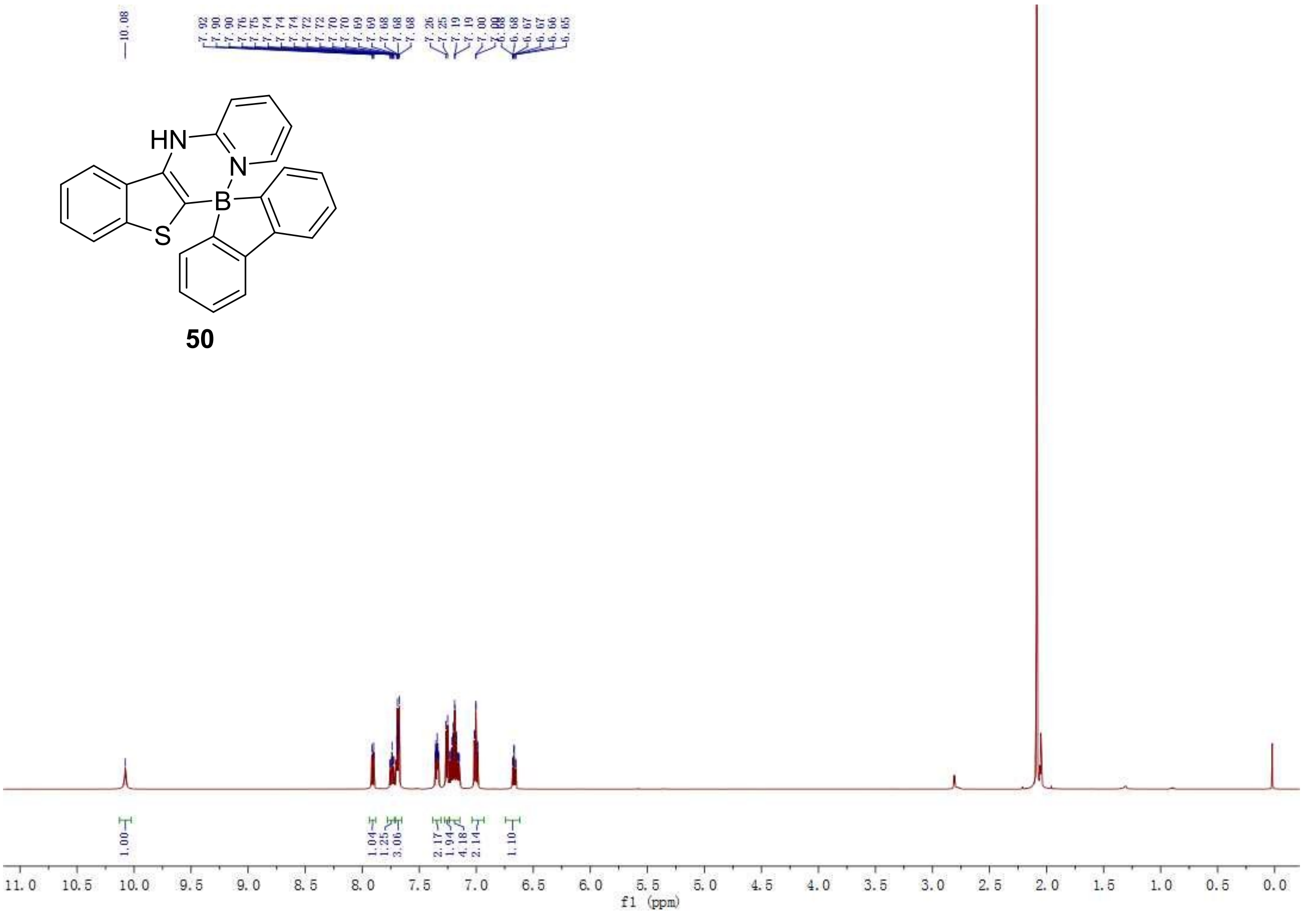

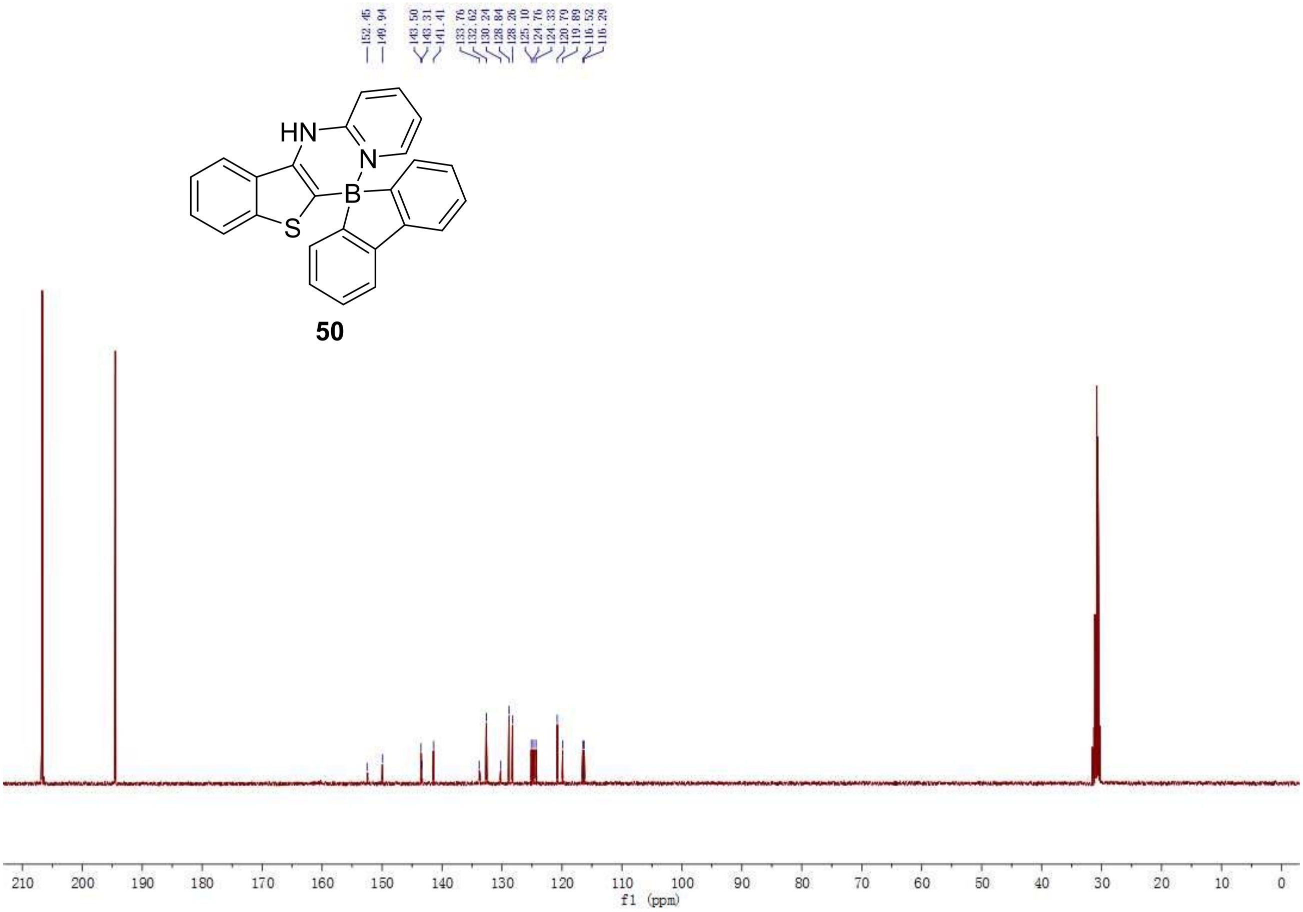

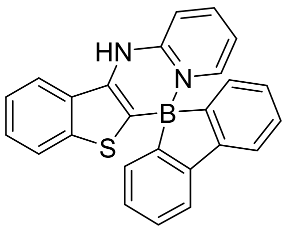

50

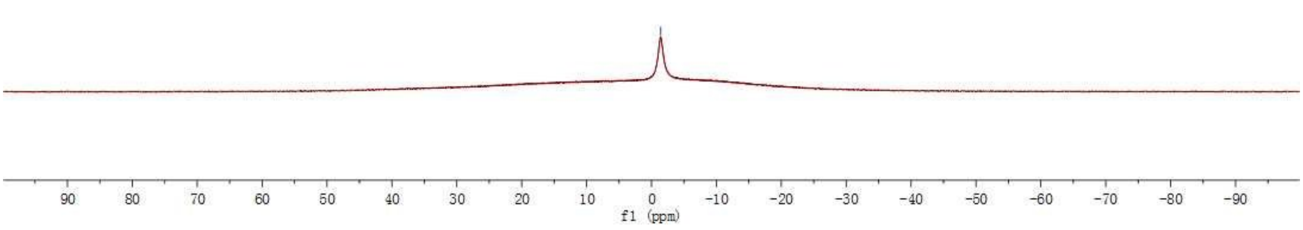

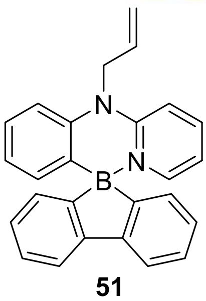

51

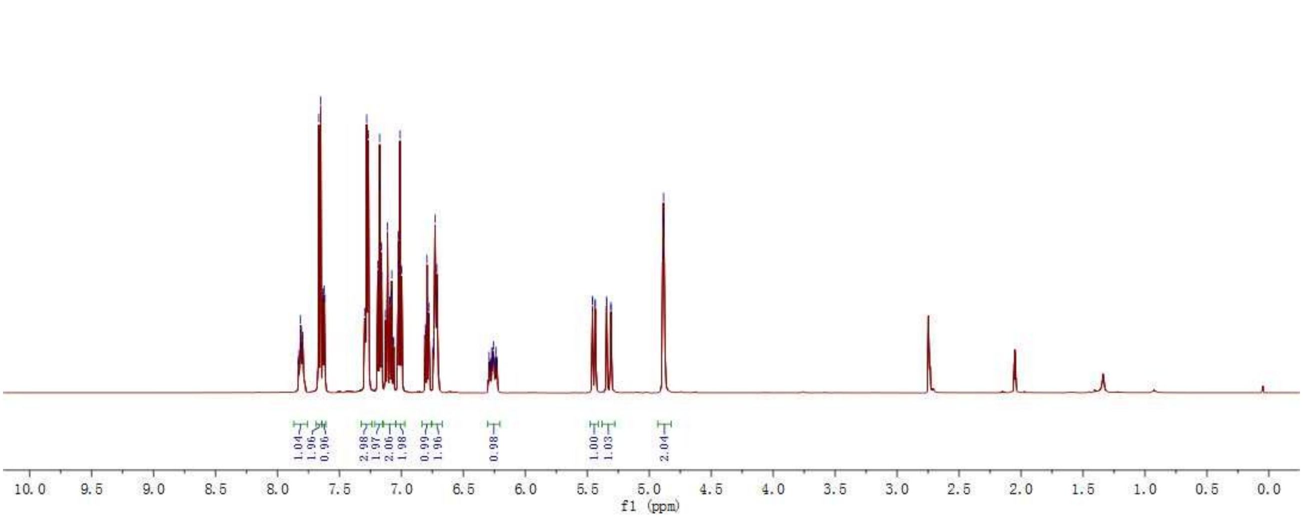

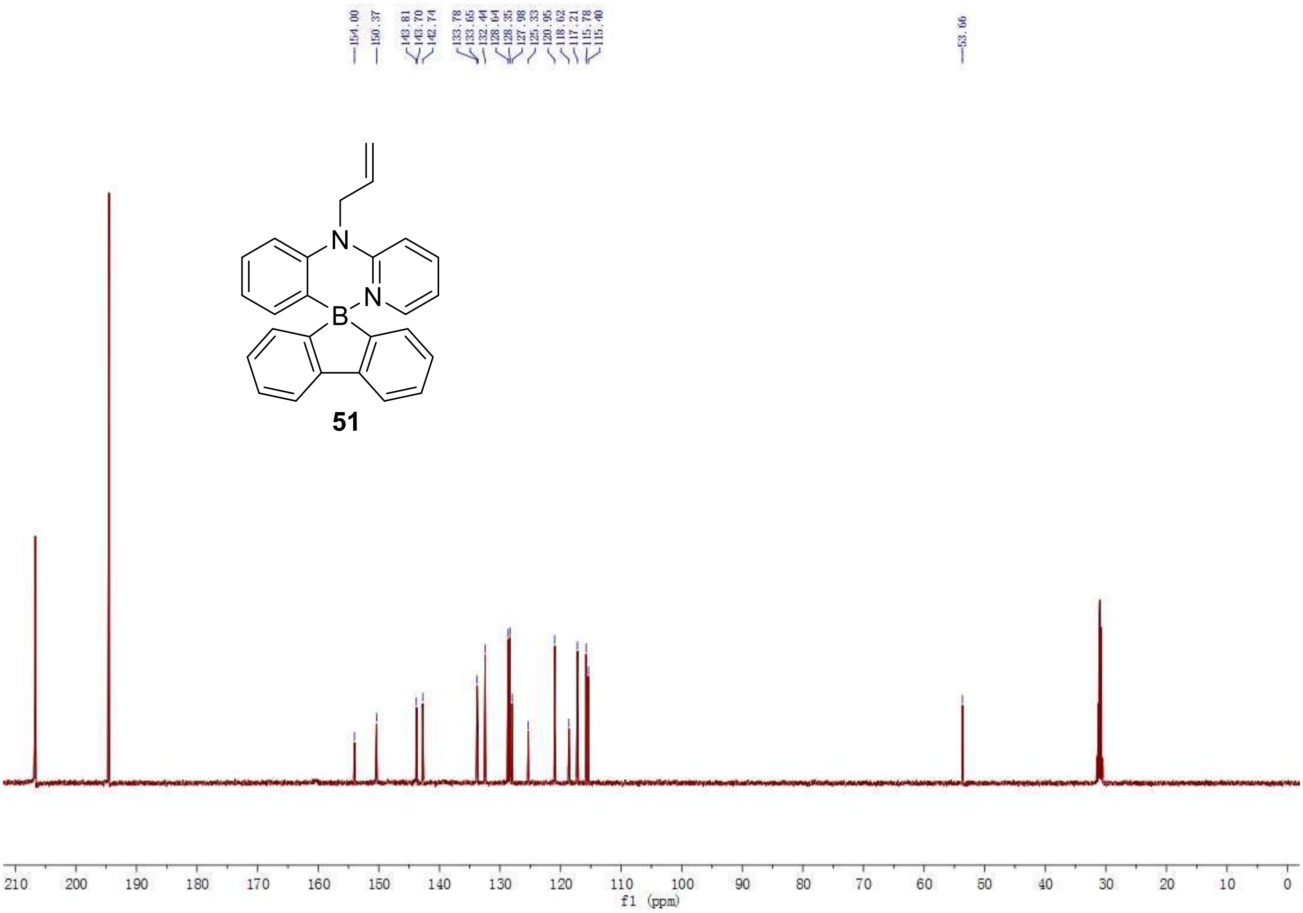
154.00
150.37
143.81
143.70
142.74
133.78
133.65
132.44
128.64
128.35
127.98
125.33
120.95
118.62
117.21
115.78
115.40
53.66
f1 (ppm)

51

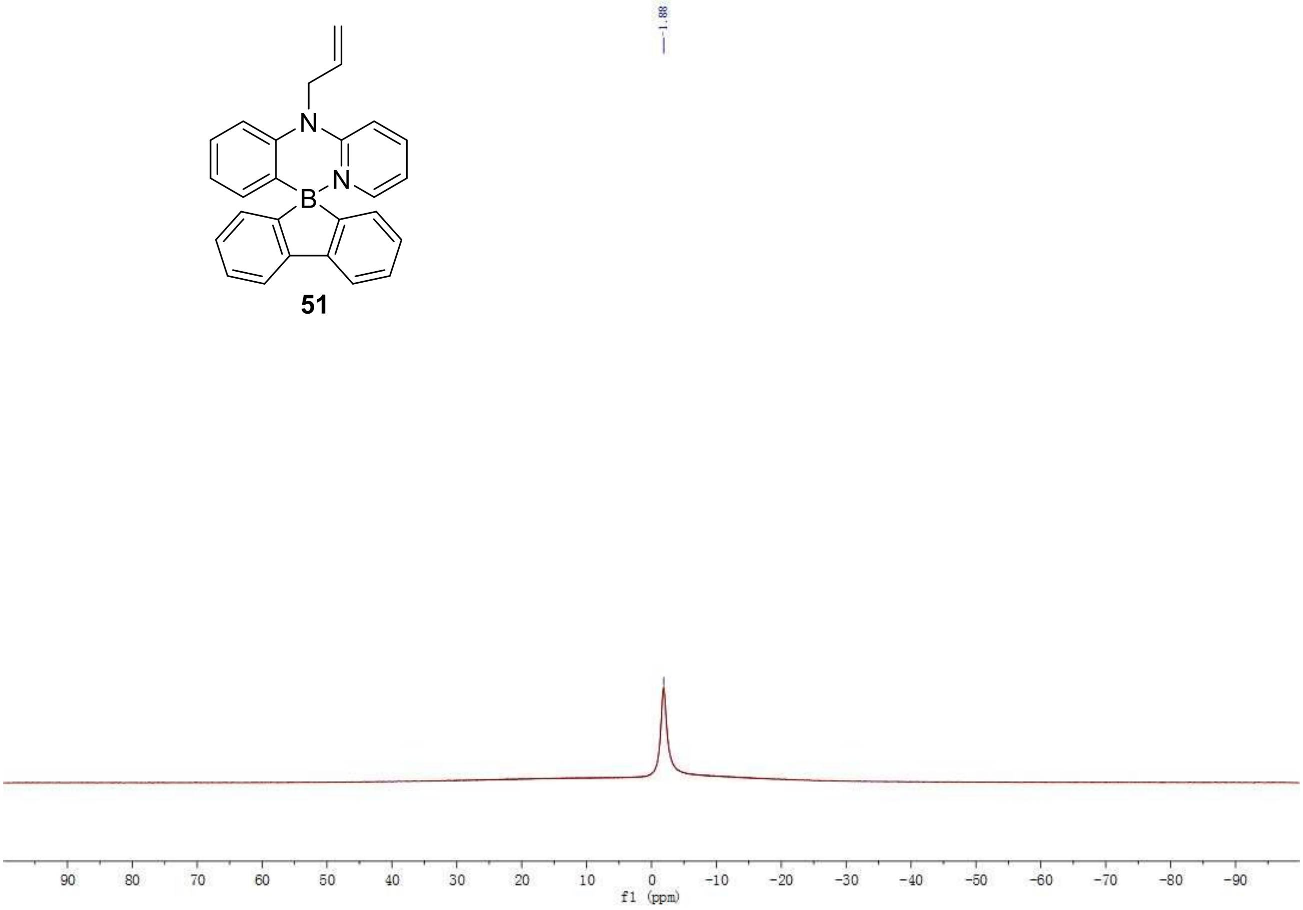
-1.88
f1 (ppm)

51

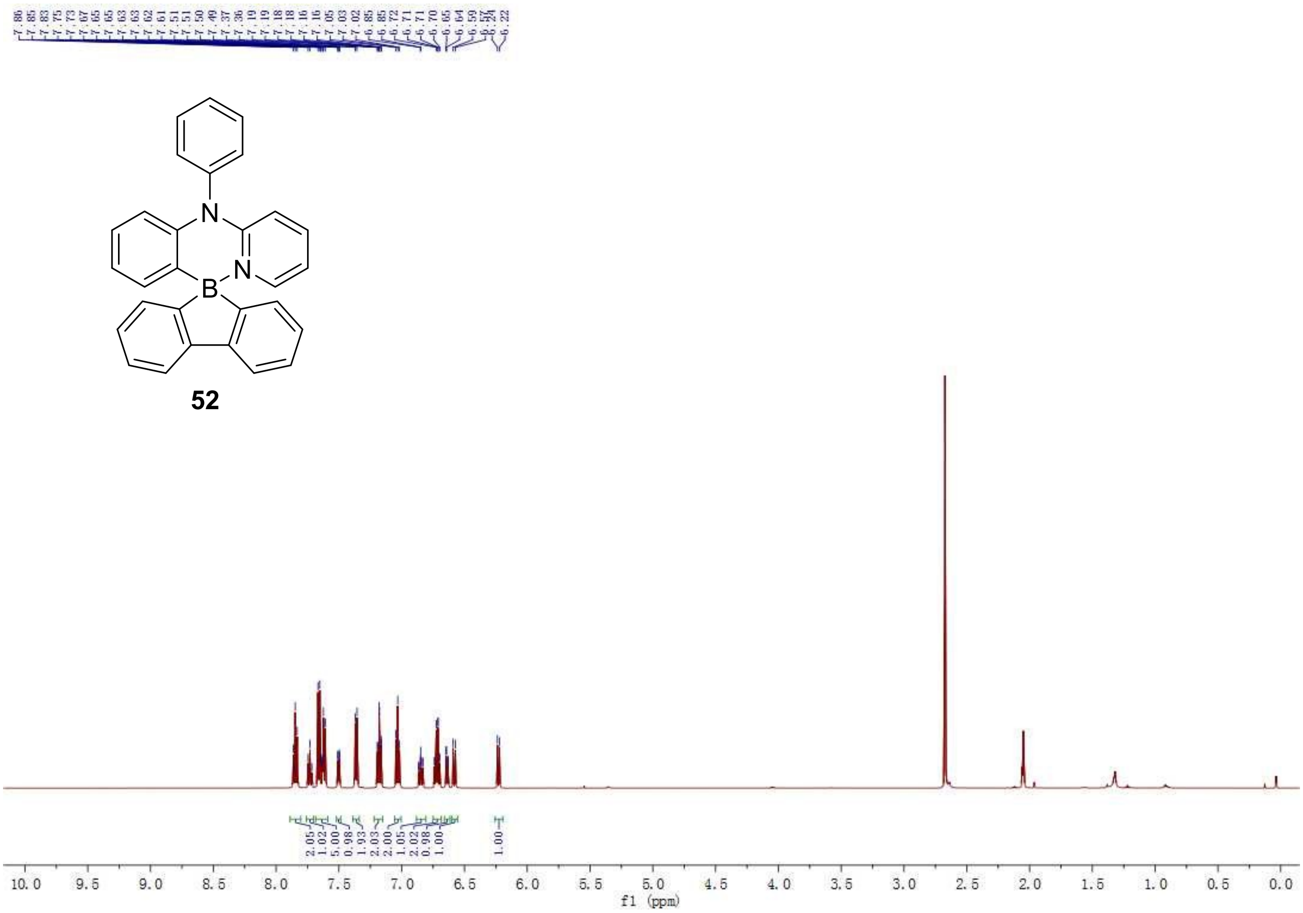
10.0 9.5 9.0 8.5 8.0 7.5 7.0 6.5 6.0 5.5 5.0 4.5 4.0 3.5 3.0 2.5 2.0 1.5 1.0 0.5 0.0
f1 (ppm)

52

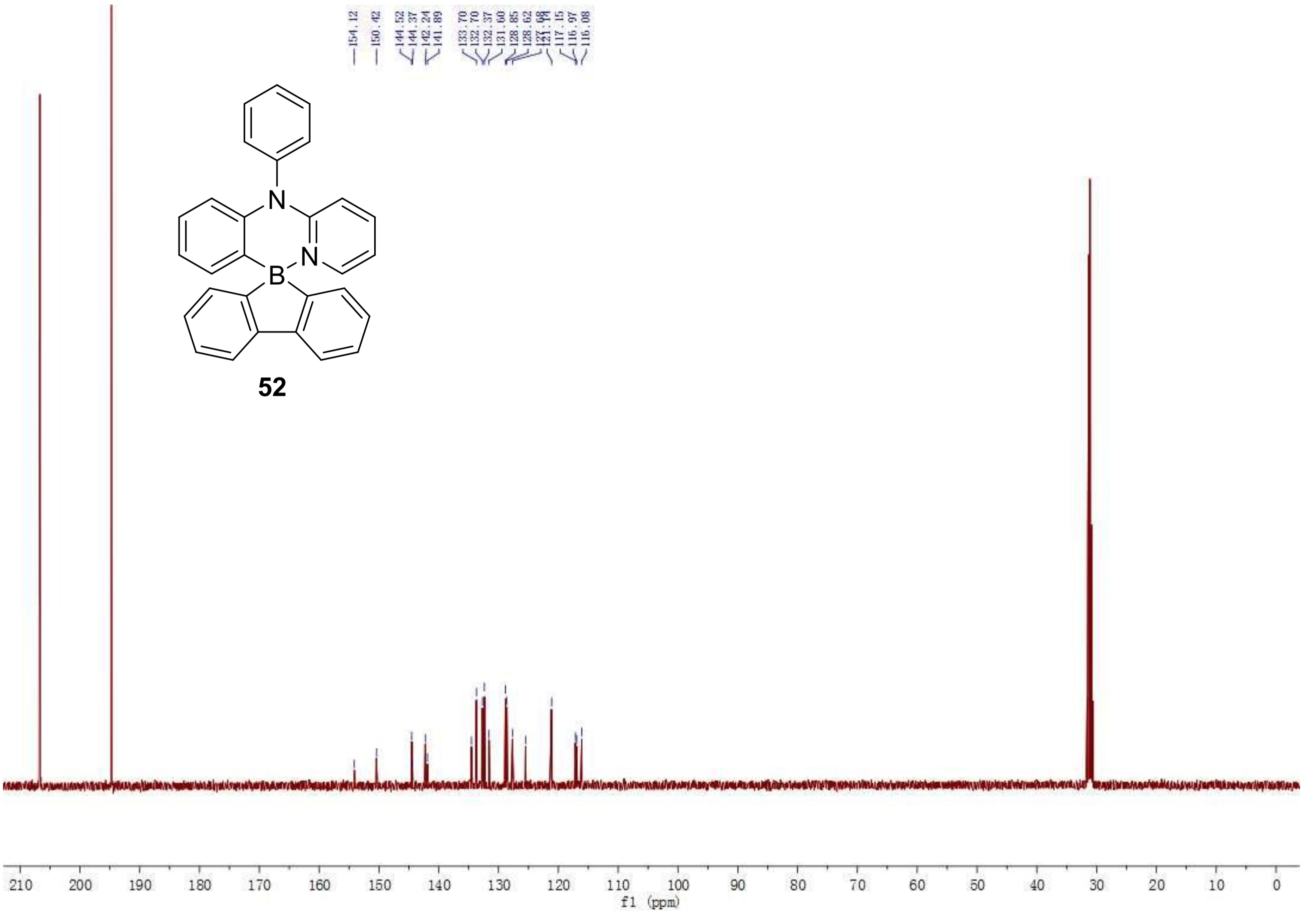
154.12
150.42
144.52
144.37
142.24
141.89
133.70
132.70
132.37
131.60
128.85
128.62
117.15
116.97
116.08
210 200 190 180 170 160 150 140 130 120 110 100 90 80 70 60 50 40 30 20 10 0
f1 (ppm)

52

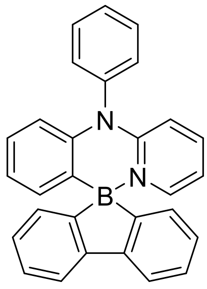

52

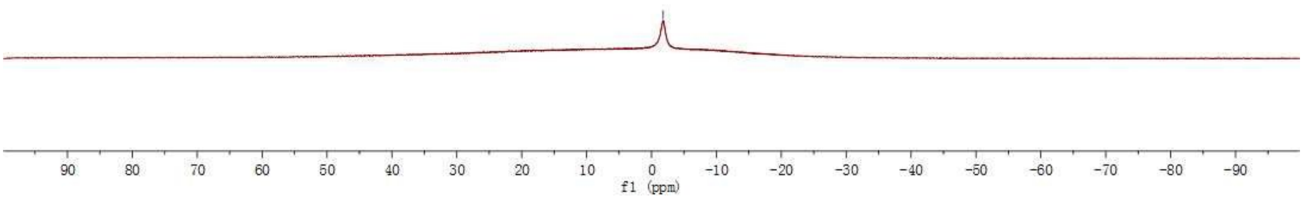

1.80
90 80 70 60 50 40 30 20 10 0 −10 −20 −30 −40 −50 −60 −70 −80 −90
f1 (ppm)

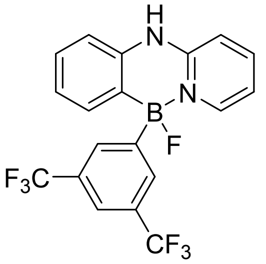

H
N
B
N
F
F_3C
CF_3

54

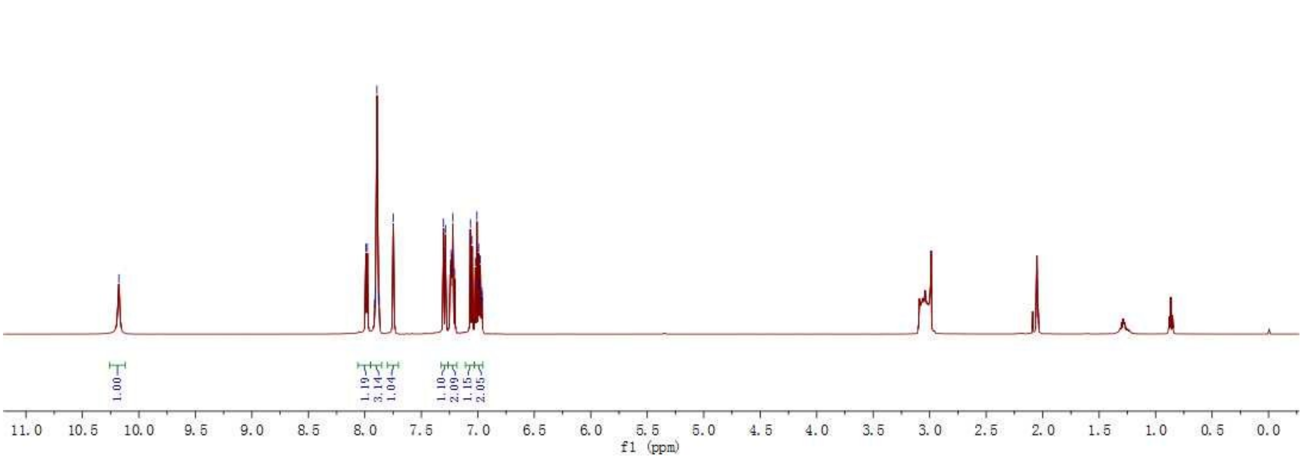

10.18
7.99 7.98 7.91 7.91 7.89 7.87 7.87 7.75 7.30 7.29 7.24 7.24 7.23 7.22 7.21 7.05 7.02 7.01 6.99 6.99 6.98 6.97 6.96 6.96
1.00
1.19 3.14 1.04
1.10 2.09 1.15 2.05
11.0 10.5 10.0 9.5 9.0 8.5 8.0 7.5 7.0 6.5 6.0 5.5 5.0 4.5 4.0 3.5 3.0 2.5 2.0 1.5 1.0 0.5 0.0
f1 (ppm)

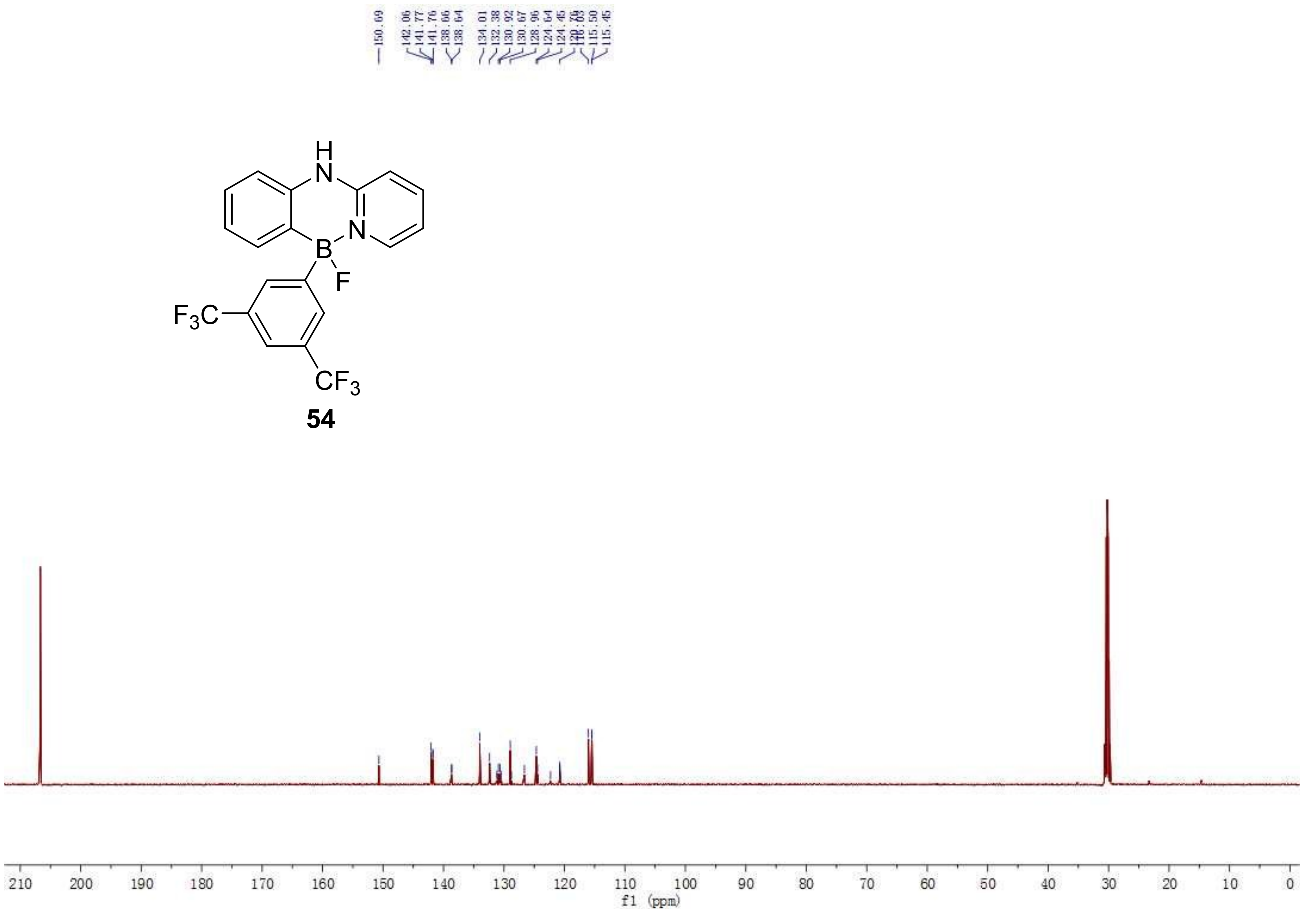
150.69
142.06
141.77
141.76
138.66
138.64
134.01
132.38
130.92
130.67
128.96
124.64
124.45
115.50
115.45
H
N
B
F
F3C
CF3
54
f1 (ppm)

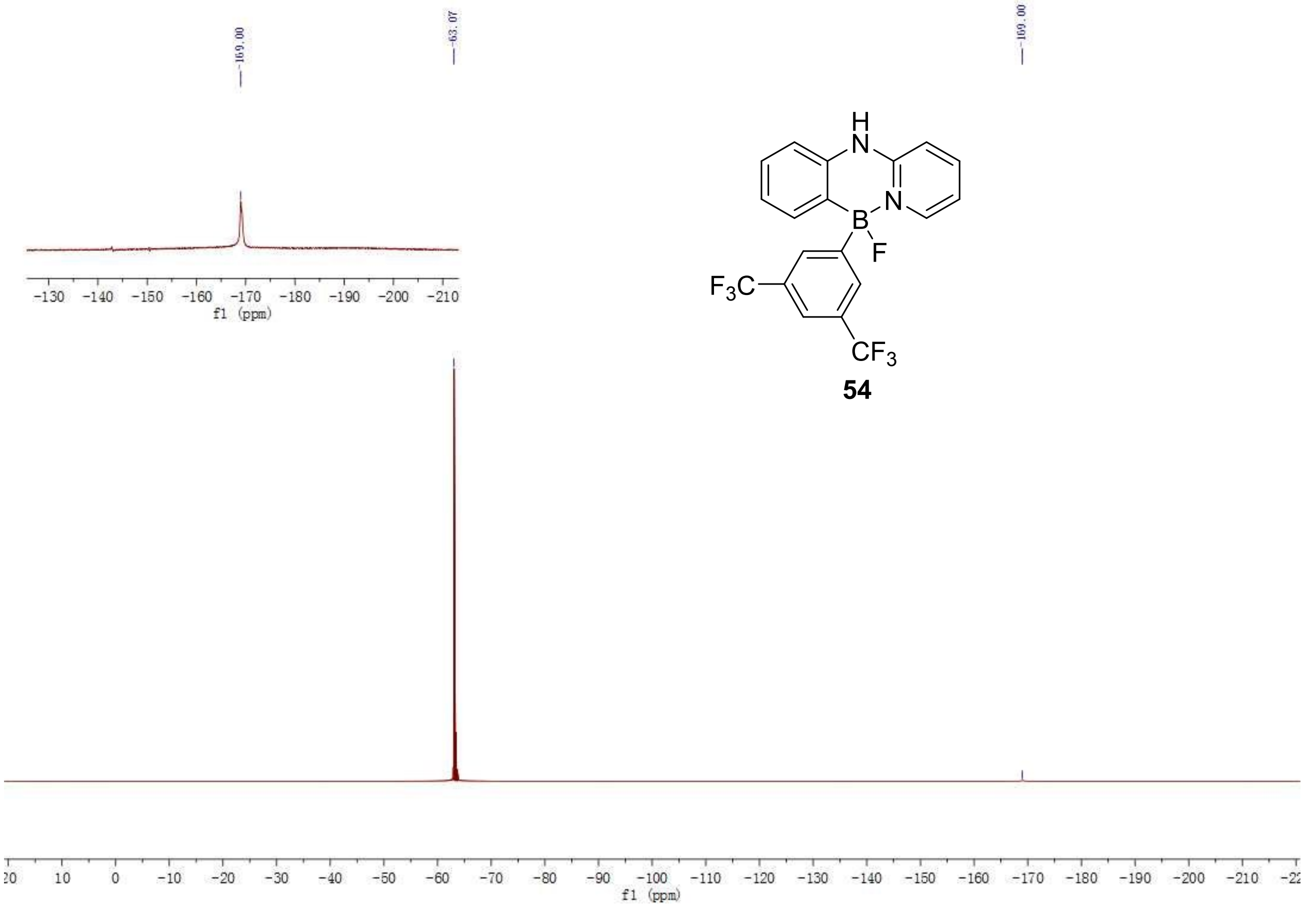
-169.00
-63.07
-169.00
f1 (ppm)
H
N
B
F
F3C
CF3
54
f1 (ppm)

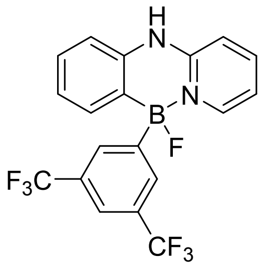
H
N
N
B
F
F3C
CF3

54

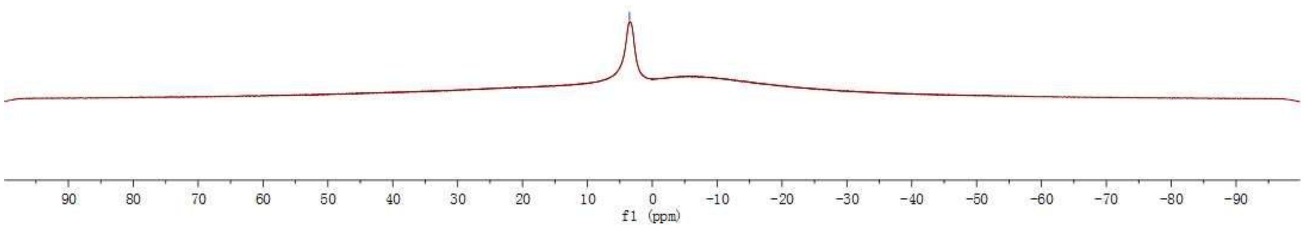
3.51
90
80
70
60
50
40
30
20
10
0
-10
-20
-30
-40
-50
-60
-70
-80
-90
f1 (ppm)
